# Minimal information for studies of extracellular vesicles (MISEV2023): From basic to advanced approaches

**DOI:** 10.1002/jev2.12404

**Published:** 2024-02-07

**Authors:** Joshua A. Welsh, Deborah C. I. Goberdhan, Lorraine O'Driscoll, Edit I. Buzas, Cherie Blenkiron, Benedetta Bussolati, Houjian Cai, Dolores Di Vizio, Tom A. P. Driedonks, Uta Erdbrügger, Juan M. Falcon‐Perez, Qing‐Ling Fu, Andrew F. Hill, Metka Lenassi, Sai Kiang Lim, Mỹ G. Mahoney, Sujata Mohanty, Andreas Möller, Rienk Nieuwland, Takahiro Ochiya, Susmita Sahoo, Ana C. Torrecilhas, Lei Zheng, Andries Zijlstra, Sarah Abuelreich, Reem Bagabas, Paolo Bergese, Esther M. Bridges, Marco Brucale, Dylan Burger, Randy P. Carney, Emanuele Cocucci, Federico Colombo, Rossella Crescitelli, Edveena Hanser, Adrian L. Harris, Norman J. Haughey, An Hendrix, Alexander R. Ivanov, Tijana Jovanovic‐Talisman, Nicole A. Kruh‐Garcia, Vroniqa Ku'ulei‐Lyn Faustino, Diego Kyburz, Cecilia Lässer, Kathleen M. Lennon, Jan Lötvall, Adam L. Maddox, Elena S. Martens‐Uzunova, Rachel R. Mizenko, Lauren A. Newman, Andrea Ridolfi, Eva Rohde, Tatu Rojalin, Andrew Rowland, Andras Saftics, Ursula S. Sandau, Julie A. Saugstad, Faezeh Shekari, Simon Swift, Dmitry Ter‐Ovanesyan, Juan P. Tosar, Zivile Useckaite, Francesco Valle, Zoltan Varga, Edwin van der Pol, Martijn J. C. van Herwijnen, Marca H. M. Wauben, Ann M. Wehman, Sarah Williams, Andrea Zendrini, Alan J. Zimmerman, MISEV Consortium, Clotilde Théry, Kenneth W. Witwer

**Affiliations:** ^1^ Translational Nanobiology Section, Laboratory of Pathology National Cancer Institute, National Institutes of Health Bethesda Maryland USA; ^2^ Nuffield Department of Women's and Reproductive Health University of Oxford, Women's Centre, John Radcliffe Hospital Oxford UK; ^3^ School of Pharmacy and Pharmaceutical Sciences Trinity College Dublin Dublin Ireland; ^4^ Trinity Biomedical Sciences Institute Trinity College Dublin Dublin Ireland; ^5^ Trinity St. James's Cancer Institute Trinity College Dublin Dublin Ireland; ^6^ Department of Genetics, Cell‐ and Immunobiology Semmelweis University Budapest Hungary; ^7^ HCEMM‐SU Extracellular Vesicle Research Group Semmelweis University Budapest Hungary; ^8^ HUN‐REN‐SU Translational Extracellular Vesicle Research Group Semmelweis University Budapest Hungary; ^9^ Faculty of Medical and Health Sciences The University of Auckland Auckland New Zealand; ^10^ Department of Molecular Biotechnology and Health Sciences University of Turin Turin Italy; ^11^ University of Georgia Athens Georgia USA; ^12^ Department of Surgery, Division of Cancer Biology and Therapeutics Cedars‐Sinai Medical Center Los Angeles California USA; ^13^ Department CDL Research University Medical Center Utrecht Utrecht The Netherlands; ^14^ University of Virginia Health System Charlottesville Virginia USA; ^15^ Exosomes Laboratory, Center for Cooperative Research in Biosciences Basque Research and Technology Alliance Derio Spain; ^16^ Metabolomics Platform, Center for Cooperative Research in Biosciences Basque Research and Technology Alliance Derio Spain; ^17^ IKERBASQUE, Basque Foundation for Science Bilbao Spain; ^18^ Otorhinolaryngology Hospital, The First Affiliated Hospital Sun Yat‐sen University Guangzhou China; ^19^ Extracellular Vesicle Research and Clinical Translational Center The First Affiliated Hospital, Sun Yat‐sen University Guangzhou China; ^20^ Institute for Health and Sport Victoria University Melbourne Australia; ^21^ Faculty of Medicine University of Ljubljana Ljubljana Slovenia; ^22^ Institute of Molecular and Cell Biology (IMCB) Agency for Science, Technology and Research (A*STAR) Singapore Singapore; ^23^ Paracrine Therapeutics Pte. Ltd. Singapore Singapore; ^24^ Department of Surgery, YLL School of Medicine National University Singapore Singapore Singapore; ^25^ Thomas Jefferson University Philadelphia Pennsylvania USA; ^26^ Stem Cell Facility All India Institute of Medical Sciences New Delhi India; ^27^ Chinese University of Hong Kong Hong Kong Hong Kong S.A.R.; ^28^ QIMR Berghofer Medical Research Institute Brisbane Australia; ^29^ Laboratory of Experimental Clinical Chemistry, Amsterdam University Medical Centers, Location AMC University of Amsterdam Amsterdam The Netherlands; ^30^ Amsterdam Vesicle Center, Amsterdam University Medical Centers, Location AMC University of Amsterdam Amsterdam The Netherlands; ^31^ Tokyo Medical University Tokyo Japan; ^32^ Icahn School of Medicine at Mount Sinai New York New York USA; ^33^ Laboratório de Imunologia Celular e Bioquímica de Fungos e Protozoários, Departamento de Ciências Farmacêuticas, Instituto de Ciências Ambientais, Químicas e Farmacêuticas Universidade Federal de São Paulo (UNIFESP) Campus Diadema Diadema Brazil; ^34^ Department of Laboratory Medicine, Nanfang Hospital Southern Medical University Guangzhou China; ^35^ Department of Pathology Vanderbilt University Medical Center Nashville Tennessee USA; ^36^ Genentech South San Francisco California USA; ^37^ Department of Molecular Medicine, Beckman Research Institute City of Hope Comprehensive Cancer Center Duarte California USA; ^38^ Department of Molecular and Translational Medicine University of Brescia Brescia Italy; ^39^ Center for Colloid and Surface Science (CSGI) Florence Italy; ^40^ National Center for Gene Therapy and Drugs based on RNA Technology Padua Italy; ^41^ Weatherall Institute of Molecular Medicine University of Oxford Oxford UK; ^42^ Consiglio Nazionale delle Ricerche ‐ Istituto per lo Studio dei Materiali Nanostrutturati Bologna Italy; ^43^ Consorzio Interuniversitario per lo Sviluppo dei Sistemi a Grande Interfase Florence Italy; ^44^ Kidney Research Centre Ottawa Hopsital Research Institute Ottawa Canada; ^45^ Department of Cellular and Molecular Medicine University of Ottawa Ottawa Canada; ^46^ School of Pharmaceutical Sciences University of Ottawa Ottawa Canada; ^47^ Department of Biomedical Engineering University of California Davis California USA; ^48^ Division of Pharmaceutics and Pharmacology, College of Pharmacy The Ohio State University Columbus Ohio USA; ^49^ Comprehensive Cancer Center The Ohio State University Columbus Ohio USA; ^50^ Sahlgrenska Center for Cancer Research, Department of Surgery, Institute of Clinical Sciences Sahlgrenska Academy, University of Gothenburg Gothenburg Sweden; ^51^ Wallenberg Centre for Molecular and Translational Medicine, Institute of Clinical Sciences Sahlgrenska Academy, University of Gothenburg Gothenburg Sweden; ^52^ Department of Biomedicine University Hospital Basel Basel Switzerland; ^53^ Department of Biomedicine University of Basel Basel Switzerland; ^54^ Department of Oncology University of Oxford Oxford UK; ^55^ Departments of Neurology and Psychiatry Johns Hopkins University School of Medicine Baltimore Maryland USA; ^56^ Laboratory of Experimental Cancer Research, Department of Human Structure and Repair Ghent University Ghent Belgium; ^57^ Cancer Research Institute Ghent Ghent Belgium; ^58^ Barnett Institute of Chemical and Biological Analysis, Department of Chemistry and Chemical Biology Northeastern University Boston Massachusetts USA; ^59^ Department of Cancer Biology and Molecular Medicine, Beckman Research Institute City of Hope Comprehensive Cancer Center Duarte California USA; ^60^ Bio‐pharmaceutical Manufacturing and Academic Resource Center (BioMARC) Infectious Disease Research Center, Colorado State University Fort Collins Colorado USA; ^61^ Department of Rheumatology University Hospital Basel Basel Switzerland; ^62^ Krefting Research Centre, Department of Internal Medicine and Clinical Nutrition Institute of Medicine at Sahlgrenska Academy, University of Gothenburg Gothenburg Sweden; ^63^ Krefting Research Centre, Institute of Medicine at Sahlgrenska Academy University of Gothenburg Gothenburg Sweden; ^64^ Erasmus MC Cancer Institute University Medical Center Rotterdam, Department of Urology Rotterdam The Netherlands; ^65^ College of Medicine and Public Health Flinders University Adelaide Australia; ^66^ Department of Physics and Astronomy, and LaserLaB Amsterdam Vrije Universiteit Amsterdam Amsterdam The Netherlands; ^67^ Department of Transfusion Medicine, University Hospital Salzburger Landeskliniken GmbH of Paracelsus Medical University Salzburg Austria; ^68^ GMP Unit, Paracelsus Medical University Salzburg Austria; ^69^ Transfer Centre for Extracellular Vesicle Theralytic Technologies, EV‐TT Salzburg Austria; ^70^ Expansion Therapeutics, Structural Biology and Biophysics Jupiter Florida USA; ^71^ Department of Anesthesiology & Perioperative Medicine Oregon Health & Science University Portland Oregon USA; ^72^ Department of Stem Cells and Developmental Biology, Cell Science Research Center Royan Institute for Stem Cell Biology and Technology, ACECR Tehran Iran; ^73^ Celer Diagnostics Toronto Canada; ^74^ Waipapa Taumata Rau University of Auckland Auckland New Zealand; ^75^ Wyss Institute for Biologically Inspired Engineering Harvard University Boston Massachusetts USA; ^76^ Universidad de la República Montevideo Uruguay; ^77^ Institut Pasteur de Montevideo Montevideo Uruguay; ^78^ Biological Nanochemistry Research Group Institute of Materials and Environmental Chemistry, Research Centre for Natural Sciences Budapest Hungary; ^79^ Department of Biophysics and Radiation Biology Semmelweis University Budapest Hungary; ^80^ Biomedical Engineering and Physics, Amsterdam UMC, location AMC University of Amsterdam Amsterdam The Netherlands; ^81^ Laboratory of Experimental Clinical Chemistry, Amsterdam UMC, location AMC University of Amsterdam Amsterdam The Netherlands; ^82^ Department of Biomolecular Health Sciences, Faculty of Veterinary Medicine Utrecht University Utrecht The Netherlands; ^83^ University of Denver Denver USA; ^84^ International Society for Extracellular Vesicles; ^85^ Institut Curie, INSERM U932 PSL University Paris France; ^86^ CurieCoreTech Extracellular Vesicles, Institut Curie Paris France; ^87^ Department of Molecular and Comparative Pathobiology Johns Hopkins University School of Medicine Baltimore Maryland USA; ^88^ EV Core Facility “EXCEL”, Institute for Basic Biomedical Sciences Johns Hopkins University School of Medicine Baltimore Maryland USA; ^89^ The Richman Family Precision Medicine Center of Excellence in Alzheimer's Disease Johns Hopkins University School of Medicine Baltimore Maryland USA

**Keywords:** ectosomes, exosomes, extracellular vesicles, extracellular particles, guidelines, microparticles, microvesicles, minimal information requirements, MISEV, reproducibility, rigor, standardisation

## Abstract

Extracellular vesicles (EVs), through their complex cargo, can reflect the state of their cell of origin and change the functions and phenotypes of other cells. These features indicate strong biomarker and therapeutic potential and have generated broad interest, as evidenced by the steady year‐on‐year increase in the numbers of scientific publications about EVs. Important advances have been made in EV metrology and in understanding and applying EV biology. However, hurdles remain to realising the potential of EVs in domains ranging from basic biology to clinical applications due to challenges in EV nomenclature, separation from non‐vesicular extracellular particles, characterisation and functional studies. To address the challenges and opportunities in this rapidly evolving field, the International Society for Extracellular Vesicles (ISEV) updates its ‘Minimal Information for Studies of Extracellular Vesicles’, which was first published in 2014 and then in 2018 as MISEV2014 and MISEV2018, respectively. The goal of the current document, MISEV2023, is to provide researchers with an updated snapshot of available approaches and their advantages and limitations for production, separation and characterisation of EVs from multiple sources, including cell culture, body fluids and solid tissues. In addition to presenting the latest state of the art in basic principles of EV research, this document also covers advanced techniques and approaches that are currently expanding the boundaries of the field. MISEV2023 also includes new sections on EV release and uptake and a brief discussion of in vivo approaches to study EVs. Compiling feedback from ISEV expert task forces and more than 1000 researchers, this document conveys the current state of EV research to facilitate robust scientific discoveries and move the field forward even more rapidly.

## 1 AN INTRODUCTION TO ISEV AND MISEV

### 1.1 Extracellular vesicles and MISEV

Extracellular vesicles (EVs) serve diverse and important roles in most biological systems, arising in part from their compositional complexity. EVs are lipid bilayer membrane‐delimited, nano‐ to micro‐sized particles that appear to be released by all cell types. The molecular and structural heterogeneity of EVs mean that many discoveries remain to be made in fundamental biology and development of biomarker and therapeutic applications, yet this same complexity also poses challenges at every stage of EV studies. From definition and categorisation to separation, characterisation, engineering and clinical applications, the ‘Minimum Information for Studies of Extracellular Vesicles’ (MISEV) aims to help all practitioners of EV research and application to follow best practices for each specific question and indication.

Now in its third iteration, MISEV2023, as a field consensus document seeks to provide recommendations and guidance on EV‐related studies that encourage enhanced research design and reporting of experimental details, building on the criteria and guidelines set out in the previous two iterations. MISEV is produced by the International Society for Extracellular Vesicles (ISEV) (https://www.isev.org). Founded in 2011 with the mission to enhance EV research globally, ISEV is the leading professional society for scientists and clinicians involved in the study and use of extracellular vesicles. ISEV engages a diverse group of researchers across the world through its annual meeting, thematic workshops and other meetings (in‐person and virtual), peer‐reviewed journals, online learning platforms, and partnerships with other societies. ISEV is thus uniquely positioned to shepherd the development and dissemination of expert consensus on best‐practice guidelines and scientific considerations.

MISEV2014 (Lotvall et al., 2014) was the first EV position paper produced by ISEV and designed to give robustness to EV analysis. MISEV2018 (Thery et al., 2018) gave a more in‐depth and critical assessment of the approaches and methods used to move the field forward, much of which still holds today. MISEV2018 also includes suggested experimental approaches to address some of the remaining challenges and to provide robust EV characterisation. The earlier MISEV recommendations remain largely or entirely valid, and MISEV2023 should be read in the context of the previous documents.

Like the iterations before it, MISEV2023 provides succinct recommendations and guidance for EV researchers, with refinement of points raised in MISEV2018 and addition of recommendations and guidance for newer areas of development. MISEV2023 broadly covers the nomenclature, pre‐processing variables, separation and characterisation of EVs, as well as in vitro and in vivo analysis of EV release, uptake and functions.

In addition to previous MISEV guidelines (Lotvall et al., 2014; Thery et al., 2018), ISEV has prompted and coordinated development and dissemination of expert consensus on best‐practice guidelines and scientific considerations including inter‐society position papers (Welsh, Van Der Pol, Arkesteijn, et al., 2020), and focused recommendations of topic‐specific experts (Erdbrügger et al., 2021; Hill et al., 2013; Lener et al., 2015; Mateescu et al., 2017; Russell et al., 2019; Verweij et al., 2021; Witwer et al., 2013) (Table 1). More recently, the ISEV Rigor and Standardisation Subcommittee oversees appointment and activities of thematic task forces and special interest groups on specific sources of EVs and other EV‐related topics. ISEV also recommends adoption of other reporting and atlas tools, such as the ‘Minimum Information for the Publication of Quantitative Real‐Time PCR Experiments’ (MIQE) for real‐time reverse transcriptase‐quantitative polymerase chain reaction (qPCR) analyses (Bustin et al., 2009) and EV‐TRACK (EV‐TRACK Consortium et al., 2017; Roux et al., 2020). Overall, the activities and recommendations of ISEV share the aim of increasing rigor, reproducibility and transparency in EV research. The goal of this MISEV document is to help practitioners in all areas of EV research and application to implement or develop best practices for each individual EV source, type, research question or application.

**TABLE 1:** Journal of Extracellular Vesicles: ISEV position papers and statements.

Title	Year	Ref.
Standardization of sample collection, isolation and analysis methods in extracellular vesicle research	2013	(Witwer et al., 2013)
ISEV position paper: extracellular vesicle RNA analysis and bioinformatics	2013	(Hill et al., 2013)
Minimal experimental requirements for definition of extracellular vesicles and their functions: a position statement from the International Society for Extracellular Vesicles	2014	(Lotvall et al., 2014)
Applying extracellular vesicles‐based therapeutics in clinical trials—an ISEV position paper	2015	(Lener et al., 2015)
Obstacles and opportunities in the functional analysis of extracellular vesicle RNA—an ISEV position paper	2017	(Mateescu et al., 2017)
Minimal information for studies of extracellular vesicles 2018 (MISEV2018): a position statement of the International Society for Extracellular Vesicles and update of the MISEV2014 guidelines	2018	(Thery et al., 2018)
Biological membranes in EV biogenesis, stability, uptake, and cargo transfer: an ISEV position paper arising from the ISEV membranes and EVs workshop	2019	(Russell et al., 2019)
MIFlowCyt‐EV: a framework for standardized reporting of extracellular vesicle flow cytometry experiments	2020	(Welsh, Van Der Pol, Arkesteijn, et al., 2020)
Urinary extracellular vesicles: A position paper by the Urine Task Force of the International Society for Extracellular Vesicles	2021	(Erdbrügger et al., 2021)

### 1.2 What MISEV IS and IS NOT

Since MISEV2018 appeared, there has been much discussion of what the guidelines mean and how they should or should not be applied. Informed by that discussion, what MISEV IS, and IS NOT, is summarised below.

MISEV IS:

1. An introduction to EV research.
2. A set of recommendations that are meant to increase rigor, reproducibility, and transparency during EV study design, execution and reporting.
3. A tool to assist reviewers and editors, using their own expert knowledge, in assessing the strengths and weaknesses of EV‐related proposals, funding applications, abstracts and manuscripts.
4. A non‐exhaustive set of examples of various useful EV techniques and platforms.
5. A rigor and standardisation framework that supports innovative EV research and applications and parties ranging from product developers to regulators.
6. An indication of current, broad consensus in the EV field as well as some areas of uncertainty and growth.
7. Relevant to translational and clinical research and applications, including production and initial evaluation of therapeutic EVs.
8. Applicable to all sorts of EV research and applications, not just those involving mammalian EVs. Although examples provided in MISEV may be specific to mammalian EVs, the basic principles are most likely applicable to all EV sources. These include informative nomenclature, definition of sources, description of separation/concentration techniques, and characterisation of EVs, properly controlled functional studies, and comprehensive reporting.

By contrast, MISEV IS NOT:

1. A one‐size‐fits‐all blueprint, a comprehensive checklist of ‘dos and don'ts’, or a substitute for careful expert judgement. There is no technique or platform that is absolutely required or prohibited by MISEV. Similarly, MISEV does not mandate use of any particular marker or markers, enriched or depleted. Chosen techniques and targets should be fit for purpose, appropriate for the experimental system, contributing to overall MISEV compliance, and properly reported. Importantly, no research group has access to all techniques and platforms.
2. A barrier to innovation. When introducing a new technique or new application of EVs, it is possible that some aspects of the approach do not fit perfectly into the existing MISEV framework, or more likely, into a reviewer's interpretation of it. See above on absolute mandates and invoke the exceptions if you must. MISEV should not stifle innovation, but rather inform how innovative or new techniques are presented and validated.
3. A means to prevent publication or funding of a particular project. Just as MISEV should not stifle innovation, it should not be used to prevent research from being shared with the community. For example, an ‘exosome’ or ‘ectosome’ study that does not prove biogenesis can be presented instead as about EVs, or an ‘EV’ report without full characterisation as a broader extracellular particle study. Proper controls might be needed to prove the contribution of EVs to an effect, but if they cannot be done, it might suffice to acknowledge the caveats.
4. A comprehensive collection of citations, each of which entirely embodies the recommendations of MISEV. The MISEV document is not a literature review or compendium. Only a small percentage of the EV literature is cited here, and each citation is made for a specific purpose. Citation in MISEV does not imply endorsement of a report, author team, journal or publisher by ISEV, nor does it suggest primacy or perfection of the cited study. Some cited studies may contain aspects that are inconsistent with MISEV recommendations. Also, many excellent studies are not cited in this document.

In summary, the spirit of MISEV is embodied in just a handful of questions:

1. What terms do you use, and what do they mean?
2. From what/where did you obtain your EVs?
3. How did you separate, concentrate, characterise and store them?
4. How confidently can you attribute a function or biomarker to EVs versus other components?
5. Have you shared data and reported methods in sufficient detail to enable others to replicate or reproduce your results?

### 1.3 How to use MISEV2023

MISEV2023 is intended to aid any and all EV researchers: from those just starting their EV journey to more established investigators who wish to understand the current state of the art and/or cutting‐edge problems faced by the EV community. However, the result is a large document that may require some help to navigate.

Nomenclature (Section 2) is applicable to all EV studies. Clear and consistent language will help to ensure that results are understandable and comparable.

For those who are newer to EV research, we consider Sections 3, 4, 5 to be vital, covering minimum considerations for sample collection/processing, EV separation methods and EV characterisation, respectively.

Sections 6, 7, 8, 9 provide further technique‐specific guidance for EV characterisation, approaches to modulate EV release and uptake, EV functional studies, and the EV analysis in vivo. These sections provide the reader with up‐to‐date information to support informed decisions, but, for the most part, do not give specific recommendations.

The information and guidelines presented in MISEV2023 thus promote rigor, reproducibility and transparency in EV science, with the goal to ensure that conclusions are supported by the experiments performed and the information reported.

Consensus: 89.3% (891) of MISEV2023 survey respondents agreed “completely,” and 10.7% (107) agreed “mostly” with Section 1: An introduction to ISEV and MISEV. No respondents disagreed (“mostly” or “completely”), and no respondents stated that they had no opinion and/or expertise.

## 2 NOMENCLATURE

### 2.1 EV definition and EV subtypes

Definition: The term ‘extracellular vesicles’ (EVs) refers to particles that are released from cells, are delimited by a lipid bilayer, and cannot replicate on their own (i.e., do not contain a functional nucleus). The current definition of EV is retained from MISEV2018, except that the 2018 use of the word ‘naturally’ (as in ‘naturally released’) has been removed to avoid unintended exclusion of engineered EVs or EVs produced under various cell culture conditions. In general, ISEV recommends use of the generic term ‘EV’ and operational extensions of this term instead of inconsistently defined and sometimes misleading terms such as ‘exosomes’ and ‘ectosomes’ that are associated with biogenesis pathways that are difficult to establish.

Regarding ‘operational terms’ that can be added as a prefix to ‘EV’ (Table 2), their use continues to be encouraged *with caution* if one or more EV subtypes are separated on the basis of characteristics such as size, density, molecular composition or cellular origin. We urge careful and clear definition of these operational terms. For example, terms such as ‘small’ and ‘large’ have been commonly used to denote EV populations over the last few years, usually after presumed size‐based populations of EVs have been separated with methods such as filtration or differential ultracentrifugation (differential UC, dUC). However, although ‘small’ might generally refer to EVs <200 nm in diameter, there is no strict consensus on upper and lower size cut‐offs, and it has also become clear that many separation methods, such as dUC, yield EV populations with overlapping size profiles. Thus, while such terminology may still be used, researchers should be aware of its limitations and strive to define terms as clearly as possible.

**TABLE 2:** Quick‐reference card on EV nomenclature and related terms.

Term	Definition	Usage
Extracellular vesicles (EVs)	Particles that are released from cells, are delimited by a lipid bilayer, and cannot replicate on their own.	Recommended
Non‐vesicular extracellular particles (NVEPs)	Multimolecular assemblies that are released from cells and do not have a lipid bilayer (non‐vesicular extracellular particle fraction).	Recommended
Extracellular particles (EPs)	Umbrella term for all particles outside the cell, including EVs and NVEPs.	Recommended
EV mimetic	EV‐like particles that are produced through direct artificial manipulation. This term is preferred over ‘exosome‐like vesicles’ and similar terms that imply specific biogenesis‐related properties.	Recommended
Artificial cell‐derived vesicles (ACDVs)	EV mimetics that are produced in the laboratory under conditions of induced cell disruption, such as extrusion.	Recommended
Synthetic vesicles (SVs)	EV mimetics that are synthesized de novo from molecular components or made as hybrid entities, e.g., fusions between liposomes and native EVs.	Recommended
Small EVs (operational term)	Based on the diameter of the separated particles, small EVs are often described as <200 nm in diameter. However, measured diameter is related to the specific characterization method.	Recommended, but caution required
Large EVs (operational term)	Based on the diameter of the separated particles, large EVs are often described as >200 nm in diameter. However, measured diameter is related to the specific characterization method.	Recommended, but caution required
Other ‘operational terms’	Physical characteristics: e.g., diameter: small extracellular vesicles (sEVs), large EVs (lEVs), density: low, medium, high (defined ranges). Biochemical composition: e.g., contains a specific (macro)molecule, such as a protein. Cellular origin and/or conditions under which EVs were generated: terms that highlight specific aspects of biogenesis such as molecular mechanisms, energy‐dependence (or lack thereof) and functional state of the parent cell related to stress or death.	Recommended, but caution required
Exosome	Biogenesis‐related term indicating origin from the endosomal system. Unless subcellular origin can be demonstrated, it is likely that a broad population of EVs is being studied, not exosomes specifically. Exosomes represent a subtype of small EVs: the diameter of intraluminal vesicles of endosomes is generally smaller than 200 nm.	Discouraged unless subcellular origin can be demonstrated
Ectosome	Biogenesis‐related term indicating origin from the plasma membrane. Unless subcellular origin can be demonstrated it is likely that a broad population of EVs is being studied, not ectosomes specifically. Ectosomes can have a wide range of sizes, including sizes similar to those of exosomes.	Discouraged unless subcellular origin can be demonstrated
Microvesicle	Biogenesis‐related term indicating origin from the plasma membrane. However, historically, the term has often been used to designate large EVs or all EVs, whatever their subcellular origin. This term can therefore lead to confusion.	Discouraged
Exosome‐like vesicles	As ‘exosome’ is a biogenesis‐related term indicating origin from the endosomal system, this and similar terms are discouraged for synthesized EV mimetics.	Discouraged

As mentioned above, terms related to presumed biogenesis pathways should be used only with caution and strong evidence. The term ‘exosome’ refers to EVs from internal compartments of the cell that are released via the multivesicular body (MVB), while the term ‘ectosome’ (a.k.a., microvesicle, microparticle) refers to EVs from the cell surface. Numerous specialised terms have also been used to denote EVs that arise during specific cellular processes such as cell migration (‘migrasomes’) or programmed cell death (‘apoptotic bodies’). In some cases, biogenesis or release of specific EV subtypes may be inhibited or stimulated by pharmacological or genetic intervention (see also 7.1). Unfortunately, most EV separation techniques do not enrich for EVs produced by different mechanisms, and definitive characterisation of biogenesis‐based subtypes is also difficult, with no universal molecular markers of ectosomes, exosomes or other EV subtypes. Therefore, ISEV discourages the use of biogenesis‐based terms unless such an EV population is specifically separated and characterised. Of note, ‘sEV’ (for small EV) and ‘exosome’ are not synonymous: small EV populations include both small ectosomes and exosomes. For the reasons above, most of the existing ‘exosome’ and ‘ectosome/microvesicle’ literature refers to a broad population of EVs, and not to EVs that are released via specific biogenesis pathways. Some EV‐like particles may not fully meet the definition of EVs as given above. For example, if a cell is extruded, the resulting particles have not been strictly ‘released’ from the cell.

### 2.2 EV mimetics

A term such as ‘EV mimetics’ (EVMs) can be used to denote EV‐like particles that are produced through direct disruption of cells, by de novo synthesis from molecular components, or by fusion of native EVs with, for example, liposomes. Whatever nomenclature is used for such particles, it will ideally indicate the general production process, differentiate the particles from native EVs, and not claim resemblance to EVs from a specific biogenesis pathway. That is, avoid ‘exosome‐like vesicles’ and similar terms that incorrectly imply specific biogenesis‐related properties. Some examples of possible terms, but without strict endorsement, are artificial cell‐derived vesicles (ACDVs) for vesicles from extruded cells and synthetic vesicles (SVs) for EV mimetics that are synthesised de novo from molecular components or made as hybrid entities, for example, fusions between liposomes and native EVs (Table 2).

### 2.3 How to approach non‐vesicular extracellular particles (NVEPs)

**FIGURE 1:**
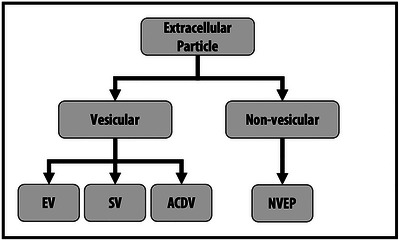
Hierarchy of EP nomenclature. Extracellular particles include vesicular and non‐vesicular particles. This figure presents several distinctions that can be made between classes of EPs, as well as examples of possible nomenclature. EP: extracellular particle; EV: extracellular vesicle; SV: synthetic vesicle; ACDV: artificial cell‐derived vesicle; NVEP: non‐vesicular extracellular particle. See also Section 2 and Table 2.

There is a growing awareness of a wide diversity of non‐vesicular extracellular particles that often co‐separate with EVs, and the ISEV community specifically requested guidance in the run‐up to MISEV2023 on how to handle and name these particles. Since ISEV is a society of EV experts, we cannot presume to establish a nomenclature for other types of extracellular particles, such as lipoprotein particles (LPPs), ribonucleoprotein particles (RNPs), viruses, or various newly proposed particle types like exomeres and supermeres. Nevertheless, how EVs relate to other particles—and how they can be separated from them and characterised along with them in complex mixtures—is of great relevance to the EV field. Therefore, MISEV2023 provides the following nomenclature proposals while recognising that other terms may be required for increased clarity (Figure 1, Table 2).

**Extracellular particles (EPs)** is the preferred overarching term for cell‐derived multimolecular assemblies in the nanometre to micron size range, including both EVs and non‐vesicular entities:

**Non‐vesicular extracellular particles (NVEPs)** are all non‐EV particles made from cell‐derived components of one or more molecular classes (e.g., proteins, nucleic acids); lipids, if present, do not form a delimiting bilayer membrane. NVEPs and EVs may have overlapping physicochemical properties, and NVEPs may greatly outnumber EVs in biological matrices. As a result, most EP separation methods result in NVEP/EV co‐isolation. Similarly, many EP characterisation methods do not identify EVs specifically. NVEPs that are smaller than EVs may not be detected by some EV characterisation methods, thus their quantity in an EP preparation may remain unknown. Therefore, when EVs and NVEPs cannot be fully distinguished from each other, the term ‘EP’ may be appropriate, or the use of ‘EV preparation’ or ‘EV‐containing preparation’.

Table 2 is a quick‐reference card of recommended nomenclature.

**Recommendations**:
  - **‘Extracellular vesicles’** is the term for particles that are delimited by a lipid bilayer and cannot replicate on their own (vesicular component of extracellular particles).
  - Operational terms are encouraged, but with caution, as these can be influenced by separation methods.
  - Biogenesis terms are discouraged unless subcellular origin can be demonstrated for the specific EV source and condition. With few exceptions, a broad population of EVs is studied, not ectosomes or exosomes specifically.
  - **‘Extracellular particles’** is the overarching term for cell‐derived multimolecular assemblies in the nanometer to micron size range, including both vesicular and non‐vesicular entities.
  - **‘Non‐vesicular extracellular particles’** is an accurate term for cell‐derived multimolecular assemblies that are non‐vesicular in nature (i.e., the non‐vesicular fraction of extracellular particles).
    Consensus: 79.5% (793) of MISEV2023 survey respondents agreed “completely,” and 19.9% (199) agreed “mostly” with Section 2: Nomenclature. 0.4% (4) “mostly” disagreed, and 0.2% (2) stated that they had no opinion and/or expertise. No respondents disagreed “completely.”

## 3 COLLECTION AND PRE‐PROCESSING: PRE‐ANALYTICAL VARIABLES THROUGH TO STORAGE

An array of factors in sample collection, pre‐processing (i.e., before specific EV separation/concentration steps), and storage of EV‐containing sources and their derivatives may affect EVs quantitatively and qualitatively. Some considerations related to these factors are common between many EV source materials, such as how to maximise (and measure) the quality of starting material; reporting all relevant donor characteristics for biofluid/solid tissue samples; measures of the quantity and quality of the source material as the baseline for the data collected during EV characterisation; and standardising and reporting pre‐processing variables. In contrast, other recommendations may be specific to the starting source, such as approaches to remove source‐specific contaminants/co‐isolates and to confirm their removal.

### 3.1 Common recommendations

- Describe the source of EV‐containing materials. For materials from human and non‐human animal donors, report relevant donor characteristics, including but not limited to age, biological sex, substance exposures (medications, substance use) and disease.
- Report the quantity (e.g., sample volume, mass) and quality of source material.
- Provide all methodologic details of sample collection.
- Consider how pre‐separation storage may influence the EVs that are eventually separated. Where relevant, avoid repeated freeze‐thaw cycles or assess effects of freeze‐thaw.
- Report all storage parameters pre‐ and post‐EV separation (including use of preservatives or cryoprotectants, temperature, time, freezing procedure, storage vessel, number of freeze‐thaw cycles and thawing method).
- Remove cells from all EV source materials as early as possible in pre‐processing. Cell disruption can form particles resembling native EVs, and post‐collection cellular processes like activation and death can alter EV composition and function.
- Assess and report the degree of depletion of cells and source‐specific, common EV co‐isolates during pre‐processing and, later, after EV separation/concentration.
- Implement quality control measures throughout the sample collection, pre‐processing and EV separation.
- If samples must be pooled to obtain sufficient EVs for study, report the number of individual samples in a pool, the donor demographics contributing to the pool, the quantity (e.g., volume) of each individual sample and final quantity. Where possible, follow up with individual samples.
- In studies that seek to determine if EVs or EV cargo can serve as biomarkers of a disease or condition, also test whether non‐enriched materials, for example, NVEPs or whole biofluid, may have similar associations.
- For those EV sources for which ISEV has a Task Force (isev.org/taskforces), we recommend that researchers keep themselves updated and informed on outputs of that Task Force. See also the next sections with some specific recommendations.

### 3.2 Cell culture‐conditioned medium

All types of cells cultured in vitro release EVs and other factors into their culture medium, thus creating cell culture‐conditioned medium [CCM; Shekari et al., 2023]. This includes eukaryotic cells from multi‐ and unicellular organisms and prokaryotic cells including gram‐positive and ‐negative bacteria and *Mycobacteria*. Most recommendations in this section apply to CCM from all cell types; additional and more specific details on bacterial EVs are provided in Section 3.3.

Cell culture parameters for both eukaryotic and prokaryotic cells include the producing cells (e.g., name, viability, passage number and seeding and harvest density); medium components (e.g., basal medium, complex additives such as serum, nutrients, micronutrients, antibiotics/mycotics and any other additives); culture conditions, including 2D/3D/suspension culture, temperature, pH, gas concentrations and any physical stimuli; duration of conditioning; harvesting approaches; and any detected contaminations or infections. Cell culture conditions directly and indirectly affect EV yield, composition, and function. Culture media components can contain EVs or may be taken up by cells and repackaged into EVs (Lehrich et al., 2021; Palviainen et al., 2019). Complex supplements such as blood serum [e.g., foetal bovine/calf serum (FBS/FCS)] and platelet lysate (PL) are often used in mammalian cell culture, but they are rich in EVs, NVEPs, and various, often undefined entities, including DNA fragments and micronutrients (Arigony et al., 2013; Lehrich et al., 2021). Depleting EVs from these supplements can be difficult to accomplish and verify (Erdbrügger et al., 2021; Lehrich et al., 2018), and depletion of complex supplements, for example, by ultracentrifugation, may depend on degree of dilution. Commercial ‘EV‐free’ products should also not be assumed to be devoid of EVs without verification. Use of both EV‐depleted medium and ‘defined’ (serum/PL‐free) media may alter cell physiology and EV production (Lehrich et al., 2021). Since viable and dying cells may release different subtypes of EVs (Crescitelli et al., 2013; Shlomovitz et al., 2021), and since EVs produced by only a few percent of dying cells may outnumber EVs generated by healthy cells, the proportion of live and dying cells in a culture affects proportions of EV subtypes and EV quantity. Unwanted microbial contamination (common: *Mycoplasma*), should be checked and reported. These microbes affect many characteristics of producing cells (Zhang et al., 2000); they or their constituents may be repackaged into EVs of the host culture (Yang et al., 2012); and some may also release their own EVs (Gaurivaud et al., 2018).

**Recommendations**:
  - CCM recommendations made in MISEV2018 (Thery et al., 2018) are still relevant. These include, but are not limited to, reporting medium composition and preparation; characteristics of producing cells including identity, seeding and harvest density and viability at harvest; culture conditions including vessel/system, surface coating (if any), temperature, and gas concentrations; physical or chemical stimulants/treatments, if any; frequency, intervals and method of CCM harvest; and any CCM storage before EV separation. If cells are from a primary source, rather than an established cell line, report harvesting and pre‐culturing conditions such as enzymatic digestion.
  - If serum, PL or other complex additives are used, report the source and the percent of the total medium. If EV depletion of such additives is done, report method and degree of depletion (including dilution, which may be necessary prior to depletion methods involving centrifugation) using the same methods used to characterise released EVs. Vendors of EV‐depleted supplements are also encouraged to report method and degree of EV depletion.
  - Non‐conditioned medium controls should be processed and characterised to assess the contribution of the medium itself to putative EV measurements.

### 3.3 Bacteria

The diversity of bacteria, bacterial EVs and source material characteristics makes it difficult to issue universal recommendations on sample type, pre‐processing, separation, collection and characterisation. Bacterial EVs arise from outer and inner membranes of gram‐negative bacteria and cytoplasmic membranes of gram‐positive bacteria through blebbing and lytic biogenesis pathways (Toyofuku et al., 2023). Different species, strains (Bitto, Cheng, et al., 2021; Bitto, Zavan, et al. 2021; McMillan & Kuehn, 2023; Zavan et al., 2023), and growth conditions (Hong et al., 2019; Keenan & Allardyce, 2000) affect EV heterogeneity on multiple levels, including function (Turner et al., 2018). Bacterial EVs can be harvested from mono‐ or polymicrobial culture in vitro, in vivo/ex vivo sources such as body fluids or faeces, and environmental samples ranging from soil to seawater. Despite this diversity, some recommendations are possible.

For most bacterial species, studies of the influence of culture conditions on the yield and composition of bacterial EVs are in their infancy, but most considerations for culture‐derived eukaryotic EVs also apply to bacterial EVs (Bose et al., 2020; Brown et al., 2015). These include effects of media composition, oxygenation/aeration and culture format (for bacteria: standing, shaking, roller bottle, bioreactor, planktonic cell or biofilm) and growth phase (Bitto, Cheng, et al., 2021; Bitto, Zavan, et al. 2021; Kuehn & Kesty, 2005; Mehanny et al., 2022; Zavan et al., 2019). Thus, culture details should be reported.

Following sample collection, as for eukaryotic EVs, all methodologic details of separation/concentration should be reported. Non‐specific methods like precipitation and ultracentrifugation may co‐isolate and/or aggregate unwanted non‐EV materials. For bacteria, these may include pili, flagellae, phage, and protein, lipoprotein, and nucleoprotein complexes. Filtration and chromatography methods are gentler alternatives (Bitto & Kaparakis‐Liaskos, 2022; Liangsupree et al., 2021). In density gradient ultracentrifugation, densities of EV‐rich fractions should be determined for each bacterium and growth condition, with clear reporting of fractions (Bitto & Kaparakis‐Liaskos, 2022; Singorenko et al., 2017). Consider that different separation methods may enrich or deplete subtypes of bacterial EVs.

Detailed characterisation of bacterial EV preparations beyond core measurements of size distribution and macromolecular content is limited by the availability of validated, commercially available affinity reagents to bacterial markers for only a limited number of species. In many cases, markers of co‐isolating materials require further definition. Lipopolysaccharide [LPS, gram‐negative bacteria, Tulkens et al., 2020], lipotechoic acid [LTA, gram‐positive bacteria, Champagne‐Jorgensen et al., 2021] and mycobacterial lipids (Prados‐Rosales et al., 2011) are universal markers for these broad classes of bacterial EVs. LPS and LTA have the advantage of commercially available antibodies. However, LPS can be present in NVEPs including LPS micelles and complexes with LPS binding protein that may be present in in vivo samples (Page et al., 2022), so appropriate controls should be included. Finally, for functional assays, normalisation methods for bacterial EV input should be accurately reported, e.g., different protein assay types can return different values (Bitto, Zavan, et al., 2021).

**Recommendations**:
  - In addition to other culture parameters, report bacterial growth phase at harvest.
  - Limit storage prior to EV separation/concentration, especially if samples are left unfiltered.
  - When obtaining bacterial EVs from in vivo and environmental sources, consider that host EVs or EVs from non‐target species are likely present.
  - LPS and LTA are broad markers of gram‐negative and ‐positive bacteria, respectively, with well‐characterised, commercially‐available affinity reagents. In many species, specific markers of EVs and non‐EV materials remain unavailable.
  - Non‐vesicular co‐isolates of bacterial EVs may include pili, flagellae, phage and protein, lipoprotein and nucleoprotein complexes.

### 3.4 Blood

Blood is the most studied biofluid in EV research, and most studies involve human blood. Previous MISEV guidelines, ISEV position papers and other publications (Clayton et al., 2019; Coumans et al., 2017; Thery et al., 2018; Witwer et al., 2013) highlighted the importance of standardisation and reporting of (i) donor variables, for example, age, biological sex, circadian rhythm, diet, exercise level and medication, and (ii) pre‐analytical processing variables such as blood collection, preparation, handling, storage, anticoagulants, centrifugation protocols and handling time (Buntsma et al., 2022; Dhondt et al., 2020, 2023; Lacroix et al., 2012; López‐Guerrero et al., 2023; Gyorgy et al., 2014; Palviainen et al., 2020), which remain valid. Here, we focus on the complexity of blood, which contains cells, lipoproteins, proteins and other factors that may be retained in EV preparations and confound downstream analysis. The degree to which blood samples are processed and EVs are separated from common co‐isolates depends on the study aim and the downstream analysis. The MIBlood‐EV was developed by the ISEV Blood Task Force to enable scientists to report the traceability of blood‐derived samples used for EV studies (Lucien et al., 2023). The MIBlood‐EV is divided into categories of: (a) general study information, (b) blood collection, processing, storage, (c) qualitative and quantitative evaluation of haemolysis, platelets and lipoproteins, three major confounding factors in blood EV research.

Blood cells account for about 45% of the blood volume, so removal of cells before any cell‐disruptive processing such as freeze/thawing (which forms EV‐like cell fragments) and avoidance of cell activation (and thus release of EVs post‐collection) is particularly important. Red blood cells are dense and thus relatively easy to separate from EVs by low‐speed centrifugation. However, red blood cells may lyse (‘haemolysis’) during blood collection and processing, releasing internal contents such as haemoglobin, which turns the plasma or serum a reddish instead of yellow colour. Most other blood cells can also be efficiently removed by centrifugation. In contrast, 1–3 μm platelets are derived from megakaryocytes, highly abundant in blood, and overlap in size range and/or density with EVs. The presence of even a few platelets may affect downstream EV analysis, and activated platelets will release large numbers of EVs. Although various centrifugation protocols are used to deplete platelets from plasma and serum (Bracht et al., 2023; Karimi et al., 2022), these protocols incompletely separate platelets from EVs, and extent of platelet depletion is typically unreported. Additional depletion of residual platelets from plasma and serum can be achieved by filtration (Bettin et al., 2022; Bracht et al., 2023).

Lipoproteins are another main confounding class of NVEPs, including high‐density, low‐density, intermediate‐density, and very low‐density lipoproteins (HDL, LDL, IDL, VLDL) as well as larger chylomicrons. They overlap in size (all but HDL), density (HDL), and/or molecular composition with blood EVs, and some lipoprotein subtypes outnumber blood EVs by orders of magnitude (Johnsen et al., 2019; Simonsen, 2017). Because neither density‐ nor size‐based separation can separate all lipoproteins from EVs, a combination of methods that exploit different physical and biochemical properties (here reported in Chapter 4) is recommended when more pure EV populations are required (Karimi et al., 2018; Ter‐Ovanesyan et al., 2023; Van Deun et al., 2020; Vergauwen et al., 2021; Zhang, Borg, et al., 2020).

Blood also contains high concentrations of free, ‘soluble’ proteins such as serum albumin, immunoglobulins and fibrinogen, as well as protein and ribonucleoprotein (RNP) aggregates, that may co‐isolate with EVs and affect downstream analysis. These proteins are generally smaller and denser than EVs, allowing separation from EVs by size exclusion chromatography (SEC), density gradient centrifugation, or combinations thereof.

Of note, the surface of EVs, especially in complex environments such as blood, is covered with a biomolecular corona of various molecules and particles [(Palviainen et al., 2020; Tóth et al., 2021; Wolf et al., 2022; Yerneni et al., 2022) and see also Section 4.7]. Hence, some blood proteins and lipoproteins, previously defined as contaminants of the EV preparation, may be truly associated with EVs and remain even after the EVs have been rigorously but gently separated from blood.

Recommendations:

- Effects of donor characteristics on blood and EV properties are better studied for blood than for many other EV sources and are thus especially important to consider and report. For example, large lipoprotein particles such as chylomicrons have elevated concentrations after dietary intake, so to minimise their influence, collect blood from overnight‐fasted donors.
- When blood is collected by venipuncture, use the largest feasible needle gauge to minimise platelet activation and haemolysis. To minimise bacterial and skin cell contamination and to avoid tissue factor‐mediated platelet activation, it may also be a good practice to discard a small volume of the blood draw (e.g., for human blood draws, the first 2–3 mL).
- Select blood collection tubes/anticoagulants that are compatible with downstream analyses.
- Following collection, minimise platelet activation and EV release by avoiding excessive agitation and low temperatures and processing to plasma or serum as quickly as possible.
- Use a plasma or serum preparation protocol that efficiently removes platelets but not EVs. If centrifugation is used, draw supernatant from the top down with a pipette, leaving a specified amount of plasma or serum on top of the pellet to avoid disturbing the pellet and releasing platelets.
- Major contaminants/co‐isolates of blood EVs are platelets, lipoproteins, haemolysis products, and a host of soluble/aggregated proteins including RNPs. Determine and report relative enrichment of EVs over whichever of these materials is important in a given study.
- Complete the MIBlood‐EV reporting tool and attach it as supplementary material for any manuscript with research using blood specimens. The completed document should also be added to the MIBlood‐EV shared folder (details at: https://www.isev.org/rigor‐standardization)

### 3.5 Urine

Urine is the second most‐analysed biofluid after blood and can be obtained non‐invasively, serially and in large quantities. Urinary EV (uEVs) and their contents are promising biomarkers and bio‐regulators in health and disease of the kidney, the urogenital tract and possibly other organs and systems (Burger et al., 2014; Carreras‐Planella et al., 2021; Erdbrügger et al., 2021; Morikawa et al., 2019; Ramirez‐Garrastacho et al., 2022). Challenges in uEV studies arise from the diverse cellular origin of uEVs and the dynamic composition of urine, which varies by fluid intake, time of collection, diet, exercise, age, biological sex, medication and health and disease status. Please refer to previous, specific recommendations of the Urine Task Force of ISEV for all stages of uEV research: a position paper (Erdbrügger et al., 2021) and a ‘Quick Reference Card’ (van Royen et al., 2023).

Here, we focus on considerations specific for urine as an EV source. For urine collection and storage, many biobanked urine samples have not been processed to remove cells prior to storage, so uEV‐specific biobanks or new collections may be needed. For any urine sample, urine proteins are the most common co‐isolates/contaminants of uEV preparations (Dhondt et al., 2020). Protein abundance in urine spans five orders of magnitude. Amongst the highest‐abundance urinary proteins (Tamm‐Horsfall protein (THP), albumin and 20 other serum‐filtered proteins) THP can not only co‐isolate with uEVs, reducing uEV purity, but also polymerise into lattice‐like networks that trap uEVs, reducing uEV yield. THP can be depolymerised and reduced by changing urine ionic strength or pH or by treating with reducing reagents (Correll et al., 2022; Liu, Cauvi, et al., 2018; Pisitkun et al., 2004). Removal of THP may be needed for downstream characterisation procedures such as mass spectrometry, but it is less necessary for other approaches (e.g., single‐EV analysis by immunolabelling).

uEV studies in particular require careful normalisation approaches because of the magnitude of inter‐ and intra‐individual variation in urine concentrations (i.e., of solutes in the urine; specific gravity), resulting from changes in the external environment, water and salt homeostasis, and circadian patterns. Because uEV levels may vary with urine concentration, normalisation between samples is necessary to counterbalance data variance. Unfortunately, there is no consensus method or marker(s) accounting for excretion rate and uEV processing that can be used for the robust normalisation of uEV quantity and/or content. Currently, normalisation for excretion rate is done based on absolute (total protein, uEV number, uEV biomarker) or relative (time collection, relative to urinary creatinine, osmolality) measures. In studies of organ‐specific uEVs, organ‐specific markers can be used; for example, prostate‐specific antigen (PSA) concentrations can account for the proportion of prostate fluid in urine.

**Recommendations**:
  - Follow previously published ISEV recommendations (Erdbrügger et al., 2021; van Royen et al., 2023).
  - Perform uEV research using cell‐free urine and cell‐free urine biobanks.
  - Where appropriate, report methodology and outcome of uEV co‐isolate/contaminant depletion (THP, albumin and other serum‐filtered proteins).
  - For normalisation, collect data both on uEVs and non‐EV urine parameters (e.g., creatinine, PSA or others as applicable) to estimate absolute or relative excretion rates.

### 3.6 Cerebrospinal fluid

Cerebrospinal fluid (CSF) bathes the central nervous system (CNS) and contains biomarkers of CNS health and disease (Gaetani et al., 2019; Hühmer et al., 2006; Jack et al., 2018; Rao et al., 2013). Several CSF‐ specific factors must be considered in CSF EV studies. CSF is produced in the brain ventricles and circulates through the brain and spinal cord in a continuous flow (Czarniak et al., 2023). This flow establishes a rostro‐caudal gradient, with lower levels of some brain proteins (e.g., S‐100β, total or phosphorylated Tau), but higher levels of others (e.g., neurofilament, amyloid‐β40 or β42) in the lumbar region relative to the brain (Jingami et al., 2019; Rostgaard et al., 2023). Hence, collection site (e.g., lumbar/spinal canal vs. brain) and volume may affect CSF composition (Cameron et al., 2019; Teunissen et al., 2009). Common confounders of CSF studies include residual cells and blood contamination, since protein concentrations in blood are 200−400 times greater than in CSF (You et al., 2005). Useful measurements of contaminants include cell counts (e.g., CSF samples that contain >500 erythrocytes/μL might be excluded (Teunissen et al., 2009) and protein assays for hemoglobin, catalase, peroxiredoxin, carbonic anhydrase I, apolipoprotein B‐100, IgM, apolipoprotein B‐100, fibrinogen or haptoglobin (Aasebø et al., 2014; You et al., 2005). Human donor characteristics reported to affect CSF biomarkers (Klener et al., 2014; Lewczuk et al., 2006; Mattsson et al., 2011) include sex (Li et al., 2017), ethnicity (Howell et al., 2017), disease‐relevant genotypes (Li et al., 2017), medications (Riekse et al., 2006; Wong, 2007) and substance use (Liu et al., 2020; Wang et al., 2021). Age (Shah et al., 2011; Wong et al., 2000; Zhang et al., 2005) may be particularly important for cohort design and normalisation considerations, since human CSF protein concentrations are high in neonates, decline through childhood and increase from adolescence through adulthood (Howell et al., 2017; Shah et al., 2011; Zhang et al., 2005). For biomarkers that cycle with circadian rhythm, the time of day for collection is important (Lucey et al., 2017). However, these effects of pre‐analytical variables may or may not affect EVs.

CSF EV studies are also challenged by very low concentration of EVs in CSF and the precious nature of CSF samples. Since CSF collection is relatively invasive, total CSF volume is limited for most patients, and sampling is usually done only for specific disease indications, the total number of samples and their volumes are small. For example, most established human CSF biorepositories are able to share 1.0 mL or less of each sample. As a result, high‐yield separation approaches and high‐sensitivity characterisation assays are especially needed for CSF EV studies (Krušić Alić et al., 2022; Sandau et al., 2020; Ter‐Ovanesyan et al., 2021). Pooling samples from multiple donors may be an option to optimise new protocols or to perform omics characterisation, with or without follow‐up with higher‐sensitivity specific molecular assays for individual samples.

**Recommendations**:
  - Report anatomic collection site and volume of CSF drawn because of possible influence of the rostral‐caudal CSF gradient.
  - Measure levels of specific co‐isolates/contaminants, such as blood cells and blood proteins, and establish exclusion criteria where appropriate, for example, >500 erythrocytes/μL from biomarker studies.
  - High‐yield separations and high‐sensitivity characterisation methods are especially important for studying CSF EVs, and sample pooling may be needed.

### 3.7 Saliva

Healthy adult humans produce 500–1500 mL saliva per day, varying with pathological and physiological conditions (Chiappin et al., 2007). Saliva is non‐invasively accessed, making it an attractive source of biomarkers, EV‐associated or not, especially for oral and periodontal conditions (Nonaka & Wong, 2022; Ogawa et al., 2008). In saliva EV studies, common co‐isolates include salivary components such as eukaryotic cells and subcellular structures, proteins such as enzymes and antibodies, electrolytes, food debris, bacterial cells and bacterial EVs (Aps & Martens, 2005; Chiappin et al., 2007; Han, Bartold, et al., 2021; Kaczor‐Urbanowicz et al., 2019; Ngamchuea et al., 2017; Ogawa et al., 2008). The overall composition of saliva depends on the relative activity and contributions of the three major pairs of salivary glands—parotid, submandibular and sublingual—as well as 300–750 minor salivary glands (Aps & Martens, 2005; Khurshid et al., 2016), which may secrete different amounts of salivary enzymes and mucins.

Parameters to report in saliva studies are whether whole saliva or saliva from one type of gland only is collected; the method of saliva collection (Beale et al., 2016; Khurshid et al., 2016; Navazesh, 1993); salivation stimulus, if any (Gomar‐Vercher et al., 2018). Recency of food and drink intake may have outsized effects on saliva quantity and quality and should be standardised if possible or assessed at collection. From studies of whole saliva, age (Xu et al., 2019), biological sex (Li‐Hui et al., 2016), smoking (Rad et al., 2010), stress (Keremi et al., 2017), exercise (Ligtenberg et al., 2016), oral hygiene, medical conditions and medications, and mental health status (Aps & Martens, 2005; Bhattarai et al., 2018) may be associated with differences in one or more of viscosity, pH, concentrations of different proteins, and saliva flow rate. However, it is not known if these factors affect or are associated with the concentration and composition of saliva EVs, so additional studies are needed.

**Recommendations**:
  - Report the source of saliva clearly (whole or from a specific gland), the method used for collection, and any stimulus used.
  - Standardise allowed food and drink intake prior to collection or, at minimum, assess these factors at collection.

### 3.8 Synovial fluid

Synovial fluid (SF) is a viscous fluid within the spaces of joints. SF EVs have potential as biomarkers and therapeutic agents for joint disorders (Boere et al., 2019) since SF is in direct contact with affected tissues (Michael et al., 2019). The viscosity of SF is due to large amounts of protein and the glycosaminoglycan hyaluronic acid (HA). This viscosity poses several hurdles to reproducible SF EV studies, for example, making it challenging to pellet cells/debris prior to freezing and hampering EV recovery. Indeed, most reported samples have been frozen and thawed before EV separation and characterisation, with inconsistent pre‐freezing removal of cells and debris (Gao et al., 2020; Rüwald et al., 2020). Hyaluronidase treatment of SF is required for accurate detection of inflammatory cells and soluble mediators (Boere et al., 2019). Most research groups use hyaluronidase to decrease SF viscosity before EV separation, but others do not (Mustonen et al., 2021). SEC may outperform UC in removal of proteins such as albumin, fibronectin and apolipoprotein A‐I (Foers et al., 2018). Donor characteristics that may associate with differences in SF variables and possibly EVs include biological sex (Kolhe et al., 2020) and disease identity and stage (Foers et al., 2020; Schioppo et al., 2021).

**Recommendations**:
  - Consider the use of hyaluronidase to reduce viscosity and obtain homogenised synovial fluid before EV separation and characterisation.

### 3.9 Milk

Milk is a rich and complex source of nutritional and immunological components, which include cells, milk fat globules (MFGs), casein micelles, soluble molecules and EVs (Ballard & Morrow, 2013). EVs separated from milk of at least 16 different species have thus far been reported, chiefly human and bovine. To allow separation of relatively pure EVs, milk components that share EV characteristics such as density and size [MFGs and cellular debris (Busatto et al., 2019)] should be removed, for example, by centrifugation), and milk should be kept at body temperature for short‐term storage (Zonneveld et al., 2014). Casein micelles, which overlap in size with EVs, are the biggest challenge, especially for milk of ruminant species. Casein micelles can be precipitated by pelleting after acidifying milk to pH 4.6 (Mukhopadhya, Santoro, Moran, et al., 2021; Rahman et al., 2019; Santoro et al., 2023; Somiya et al., 2018), aggregated by enzymatic treatment (Gao et al., 2019), or dissociated by sequestering calcium with EDTA (Gao et al., 2019) or sodium citrate (Benmoussa et al., 2020). Currently, there is no preferred method, but acidification and EDTA are used most often. Following pre‐processing, cleared milk supernatant can be stored until EV separation. Methods such as UC, dgUC and SEC may be combined for higher purity, since single‐step approaches will yield a low purity. Colloidal properties and acceptable storage times until processing may be different for raw, homogenised, pasteurised, ultra‐high temperature‐treated, and dried/powdered milk (Mukhopadhya, Santoro, & O'Driscoll, 2021). Furthermore, the effects of storage length and temperatures have yet to be comprehensively determined.

**Recommendations**:
  - Keep milk at body temperature for short‐term storage prior to storage or EV separation.
  - Common EV co‐isolates include cells/components, milk fat globules, and casein micelles. These should be removed (and/or, in the case of micelles, disrupted), and their presence tracked through the EV separation process.

### 3.10 Solid tissue

Cell‐EV interactions in solid tissues may primarily involve EVs that are released near the site of action. It is thus important to study EVs in tissue. However, greatly complicating the study of tissue EVs is the interrelated diversity of tissue harvesting and storage methods, cellular and extracellular matrix composition, and physical properties. Despite these challenges, two basic approaches to tissue EV studies have been developed and applied mostly to brain or tumor tissues.

Tissues can be used for EV studies by keeping tissues/cells ‘alive’ in culture after harvesting or by harvesting EVs directly from tissue before or after storage. Some tissues can be cultured ex vivo over several days and culture medium harvested for EV separation (Lunavat et al., 2017; Jeurissen et al., 2017; Jingushi et al., 2018). EV preparations may include tissue EVs present in the original tissue, EVs released during culture (perhaps with different properties from the native EVs), and products of cell death in culture like apoptotic bodies (Carrel & Burrows, 1911). Keeping tissue under conditions as close to their in situ environment as possible may be very important, such as maintaining tissue hydrated prior to culturing and avoiding high oxygen concentrations, although limited evidence has been gathered for the influence of these factors on collected EVs. Alternatively, tissue is processed immediately after resection (Cianciaruso et al., 2019; Crescitelli et al., 2020; Crescitelli et al., 2021; Gallart‐Palau et al., 2016; Huang et al., 2020; Jang et al., 2019; Jeppesen et al., 2019; Perez‐Gonzalez et al., 2012; Steenbeek et al., 2018) or after prior storage, usually freezing (Hurwitz et al., 2018; Hurwitz et al., 2019; Huang et al., 2020; Perez‐Gonzalez et al., 2012; Vella et al., 2017; Yelamanchili et al., 2015). A preliminary study found no major differences in EV composition in fresh versus frozen tissues (Shen et al., 2023). Tissues are typically divided into small pieces [using tissue homogenizers (Gallart‐Palau et al., 2016; Hurwitz et al., 2018; Hurwitz et al., 2019; Yelamanchili et al., 2015), vortexing (Banigan et al., 2013) or slicing (Huang et al., 2020; Jeppesen et al., 2019; Polanco et al., 2016; Vella et al., 2017)], followed by enzymatic treatment to disrupt the extracellular matrix (ECM) (Jingushi et al., 2018). These methods result in different degrees of cell damage, potentially introducing EV‐like artefacts.

**Recommendations**:
  - For ex vivo culturing approaches, keep the tissue as close as possible to its ‘native’ conditions, including maintaining hydration and nutrition. Consider also the influence of cell death processes on the EV preparation.
  - For separating EVs directly from tissue (without ex vivo culturing), establish or follow best practices for the specific tissue in harvesting (e.g., perfusion or not of an animal model to minimise effects of blood); storage (does freezing affect outcome?); physical and enzymatic tissue separation (if done) and influence of specific EV separation/concentration methods.
  - Tissue EV characterisation should focus in particular on tracing the presence of cellular components that may be expected to be depleted in EVs, since cells and cellular artefacts may be the key contaminants of tissue EV preparations.

### 3.11 Other sources

Not all sources of EVs are covered above; only those for which ISEV recently had or currently has a Task Force. ISEV members are welcome to propose formation of new task forces where no ISEV task force yet exists. These, in turn, may help to inform best practice.

### 3.12 Pre‐separation and post‐separation storage

Storage conditions of both pre‐separation sources and post‐separation EVs may also affect EV yields, contents, functionality, and the ratio of single particles and aggregates. For most EV sources, pre‐processing is advisable prior to pre‐separation storage to remove potentially interfering entities such as cells. However, stringent pre‐processing is not always possible. Details of whatever steps are performed should be reported, and an explanation given if pre‐processing cannot be done. Acceptable storage prior to EV separation varies by source. Storage conditions, including any additives [e.g., bactericidal agents (Lucas et al., 2021)], should be fully reported and the influence on EV quantity and quality investigated if not already known.

Following separation of EVs, EVs should be studied in as native a form as possible. However, for most studies, stored EVs are used. Here, several considerations apply. All storage vessels and their materials should be reported, as EVs can be lost by attaching to surfaces (Evtushenko et al., 2020). Separated EVs may be stable without freezing for some time, but this may vary by EV composition and source and of course information on storage of EVs from some matrices is more comprehensive to date compared to information on EVs from other matrices. Long‐term storage is typically at −80°C, although other temperatures have been examined. For example, saliva EVs were reportedly stable at 4°C for up to 20 months, retaining membrane integrity and protein content (Kumeda et al., 2017). Urinary EVs have reportedly been stored at −20°C for up to four years (Barreiro et al., 2021). Lyophilisation of EVs is also possible (Trenkenschuh et al., 2022). There is conflicting evidence on the effects of freeze‐thaw cycles on EV properties. A study of saliva EVs found minimal effects of freeze‐thawing on membrane integrity (defined as dipeptidyl peptidase IV activity) (Kumeda et al., 2017). However, studies of various sources of EVs have reported particle concentration and other changes with freeze‐thawing (Gelibter et al., 2022; Görgens et al., 2022). Cryoprotectants may reduce effects of freeze‐thaw (Le Saux et al., 2020; Lőrincz Á et al., 2014); for example, supplementing phosphate buffered saline (PBS) with human albumin and trehalose (PBS‐HAT) reportedly improved short‐ and long‐term stability for EVs stored at −80°C and through several freeze‐thaw cycles (Görgens et al., 2022). Since optimal storage conditions may vary by EV composition and source, the freezing method (e.g., snap‐freezing in liquid nitrogen, gradual freezing), suspension buffer (including cryoprotectants and other additives), temperature, duration of storage until use, thawing method (speed, temperature) and number of freeze‐thaw cycles should be reported. Freeze‐thaw cycles should be minimised, for example, by a careful aliquoting strategy, and samples with different numbers of freeze cycles may not be directly comparable.

Consensus: 70.4% (703) of MISEV2023 survey respondents agreed “completely,” and 28.5% (284) agreed “mostly” with Section 3: Collection and pre‐processing: pre‐analytical variables through to storage. 0.1% (1) “mostly” disagreed, and 1.0% (10) stated that they had no opinion and/or expertise. No respondents disagreed “completely.”

## 4 EV SEPARATION AND CONCENTRATION

**FIGURE 2:**
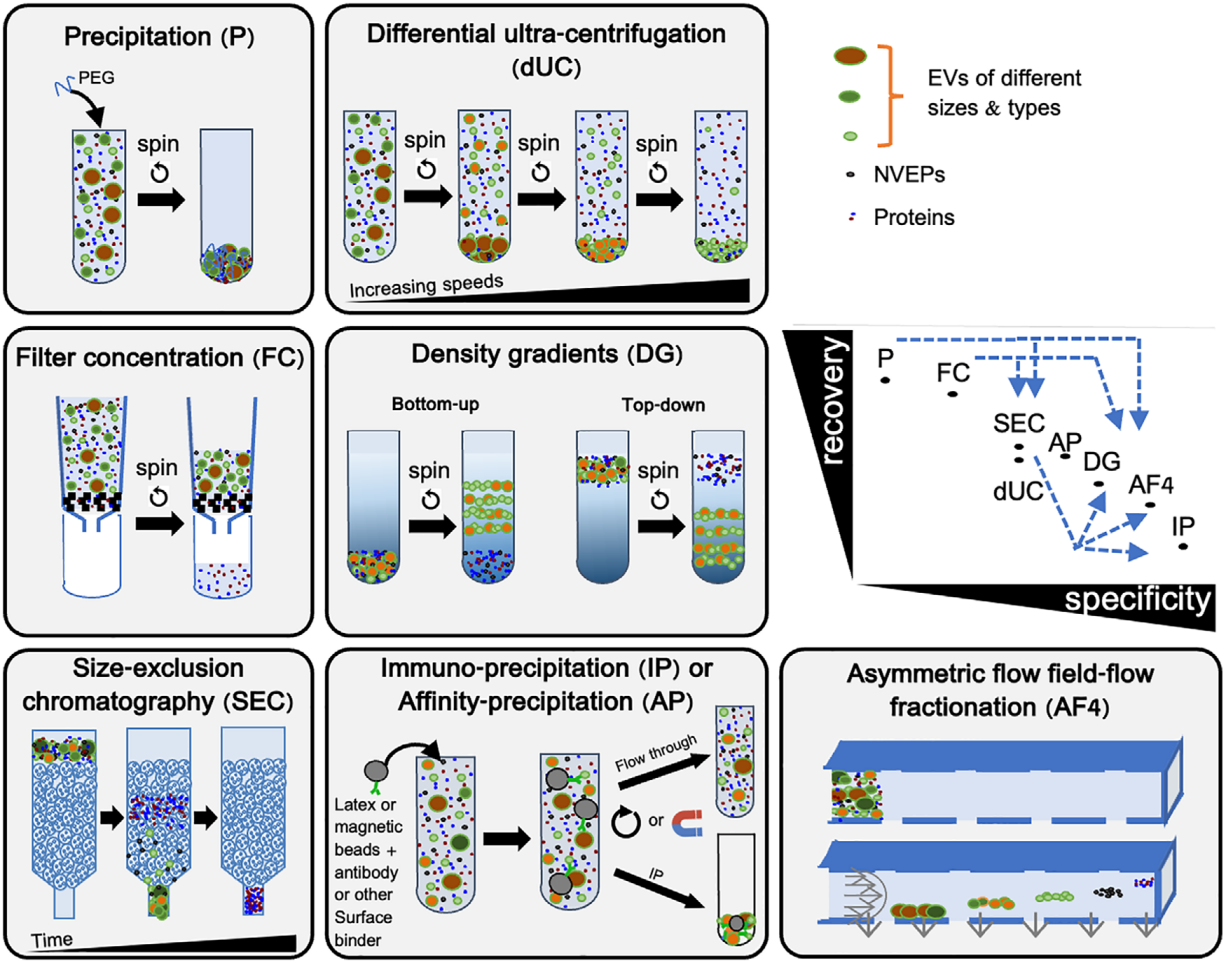
Position of some EV separation and concentration methods on a recovery (yield) versus specificity grid. Dashed blue arrows indicate combinations of methods resulting in increased specificity. Specificity can be of different types: Size exclusion chromatography (SEC) separates EVs by size from many (but not all) NVEPs, but all EV types are recovered together, while differential ultracentrifugation (dUC) separates EV subtypes based on their size/weight, but also co‐isolates NVEPs at high speeds. Note that many ‘exosome purification’ kits use precipitation (P), thus do not isolate pure exosomes or even EVs but a mixture of EPs, while some use affinity precipitation (AP), which may be more specific to EVs but not exosomes. Those who develop new methods should consider positioning their EV outcomes on such a graph.

EVs are typically characterised and used after one or more separation or concentration procedures. Trends in these approaches have been previously assessed by ISEV (Gardiner et al., 2016; Royo et al., 2020). Separation/concentration can be performed according to the EV biophysical characteristics of size, density, charge and surface composition (specific surface molecules). Other terms that are sometimes used for these procedures include ‘enrichment’, ‘purification’ and ‘isolation’. The material captured after separation/concentration is an ‘EV‐containing preparation’ or ‘EV preparation’ that may require storage prior to analysis or use. Any separation method should be chosen based on the known properties of the specific EV sources and the desired EV yield and specificity. When separating complex biofluids, quantification of yield and specificity for total EVs will likely be estimates, since particle number quantification is not always EV‐specific and/or typically relies on surrogates of EV abundance such as spike‐in populations or measurement of detectable subpopulations. Figure 2 shows the position of some commonly used methods for EV preparation on a yield (recovery) versus specificity grid. This section provides information and suggestions on some of these methods. More detailed information can be found in the literature (Hendrix et al., 2023).

EVs can sometimes be studied or used directly and immediately in the source matrix. In biomarker studies, for example, there may be no need to separate or concentrate EVs from a biological matrix if sufficient specificity and sensitivity are reached with the unfractionated sample. In some cases, EVs can also be analysed specifically and directly in a biological fluid (Duijvesz et al., 2015; Woud et al., 2022). However, to show exclusive EV association of a proposed biomarker or function, separation may be required in the first instance, and further guidance on this is provided here.

### 4.1 EV concentration

Concentration in EV studies is the act of increasing the particle number:sample volume ratio. Concentration may be needed in various settings. Large volumes of source materials like CCM, urine, milk may require concentration before EVs can be separated from other EPs. For example, chromatography columns may have a maximum loading volume, while some separation methods may be more efficient if material is first concentrated (e.g., some immunoisolation procedures). Concentration methods may, but do not necessarily, also achieve some degree of separation of particle types.

Concentration can be done by several approaches. Polymer‐based methods of precipitation reduce the availability of biomolecules to solvent, ‘crowding out’ water molecules. This allows suspended/dissolved materials including EPs to be pelleted by low‐speed centrifugation. Some commercial kits that are described as ‘exosome isolation’ kits in fact rely on such polymer precipitation and do not strictly ‘isolate’ EVs, much less subtypes of EVs. Precipitation methods may not achieve any appreciable separation of EPs (Gámez‐Valero et al., 2016; Karttunen et al., 2019; Lobb et al., 2015; Paolini et al., 2016).

In filtration, a suspension passes through a filter by, for example, gravity, centrifugation or vacuum: water and molecules smaller than the molecular weight cut‐off of the filter pass through, while EPs larger than the cut‐off are recovered in the concentrated fluid compartment of the filter. A variety of filter cut‐offs are available, including 3, 10, 100 and 1000 kDa, allowing filtration to achieve some degree of size separation, not just concentration. A cut‐off of 100 kDa retains EVs while removing many proteins, while a cut‐off of 1000 kDa may allow passage of some smaller EVs. However, another consideration is recovery, since different filters/tubes may allow different levels of EV ‘sticking’ and thus recovery (Vergauwen et al., 2017). Please note that filtration may also be performed to retain microbes (‘sterilization’) or large EVs/EPs in the pre‐filter compartment; although care should be taken to avoid extrusion. Tangential flow filtration (TFF, also called cross‐flow filtration) is a filter‐based concentration method in which liquid and molecules smaller than the pores pass through the filter perpendicularly to the flow applied to the EV‐containing fluid. This allows continuous flow and repeated passages of the fluid unless and until the filter is clogged, and thus allows processing of large volumes of fluid. As for other filtration methods, size‐based separation can be achieved based on the molecular weight cut‐off of the filter. TFF has been successfully and reproducibly used for large‐scale EV production, for example, for therapeutic applications (Busatto et al., 2018; Lamparski et al., 2002). Finally, concentration can also be obtained by (ultra)centrifugation, for which parameters are described in the next section.

**Summary: Concentration**

- Can be done by polymer‐based precipitation, filtration including tangential flow filtration, and (ultra)centrifugation.
- Leads to EV‐containing preparations containing variable amounts of NVEPs and proteins, depending on the exact method and variables such as filter cut‐off (size or molecular weight).

**Reporting recommendations**: for concentration, report the following:

- nature of the material used for concentration;
- initial and final volumes of biofluid;
- time of processing (incubation with polymer, centrifugation through filters or directly);
- flow rate (for TFF);
- size or molecular weight cut‐off (for filtration/concentration);
- temperature during concentration.

### 4.2 Differential (ultra)centrifugation

The principle of differential ultracentrifugation (dUC) is to apply increasing relative centrifugal forces (RCF = g‐force) to the EV‐containing fluid, from which intact donor cells or tissues have first been eliminated by one or more low speed centrifugations. The aim is to pellet sequentially EPs of decreasing sedimentation coefficients. Since the sedimentation velocity of a sphere is proportional to its diameter squared and to the density contrast between the particle and the medium (Stokes’ law equation), the largest and/or densest EPs tend to be pelleted in the first (medium speed/short time) steps, while the smallest and/or least dense are recovered predominantly after higher speed/longer centrifugation. However, in practice, perfect EV separations are not achieved by this method, and pellets from different centrifugation speed have overlapping properties and variable biochemical and physical parameters.

Whatever the centrifugation steps used, as detailed in MISEV2018, report speed in rpm and rotor type (to allow calculation of adjusted k‐factor), time of centrifugation (to allow calculation of the sedimentation coefficient of the pelleted particles), and temperature. Instrument acceleration and deceleration settings should also be reported. In typical dUC workflows reported in the literature, a maximal force of around 10,000 to 20,000 × *g* is applied for between 10 and 90 min to enrich putatively larger/denser EVs, while a maximal force of around 100,000 to 200,000 × *g* is applied for 45 to 150 min to pellet putatively smaller/lighter EVs. These figures can be used to calculate the sedimentation coefficient (S) of the particles recovered by these different protocols: S = adjusted K factor of the rotor/Time of centrifugation. Theoretically, particles with S coefficients in the range of 15–150 are recovered by the ‘larger EV’ centrifugation conditions, and those in the range of 2 to 5 by the ‘smaller EV’ conditions. Particles with smaller S can be recovered by extending the speed and time of centrifugation, at the cost of increasing NVEP/free protein co‐isolation. Depending on the centrifugation parameters, the resulting pellets may be enriched for large/dense or for small/light EVs, but complete separation of these populations is not achieved. Yield of smaller EVs may also be low, especially when suspended in protein‐rich fluids such as blood products and complex culture medium components, and this problem may not be resolved by simply increasing centrifugation time or speed (Driedonks et al., 2019; Zhang, Borg, et al., 2020). Examples of dUC protocols (with or without density gradient, see next section) and downstream comparison of EVs include (Jeppesen et al., 2019; Kowal et al., 2016; Lischnig et al., 2022; Martin‐Jaular et al., 2021).

The majority of published studies have focused on smaller EVs and thus discard and/or do not analyse the pellet(s) obtained with lower‐speed centrifugation. To allow comparison between studies and to avoid pelleting larger particles and potentially introducing artefacts, however, it is recommended to perform these first centrifugations. The strong g‐force of high‐speed UC has also been shown to induce aggregation of EVs (Linares et al., 2015), but this may not be observed for all sources of EVs. When analysing a new source of EVs, retain the intermediate centrifugation pellets and analyse them side‐by‐side at least once with the final, highest‐speed pellet to determine whether the molecules or activity of interest are specifically enriched in small EVs or are also present in other subtypes.

**Summary: dUC**

- Enriches for EV subtypes that are separated according to their sedimentation coefficient, proportional to their diameter and density.
- Co‐isolates NVEPs that have the same sedimentation coefficient as EVs, especially after high‐speed and lengthy ultracentrifugation.
- May induce aggregation of EVs.

**Reporting recommendations**: for differential (ultra)centrifugation, report the following:

- speed, rotor type, and time of centrifugation, to allow calculation of the adjusted k‐factor (to apply to other rotors) and the sedimentation coefficient of the pelleted EPs;
- tube type and sample volume in the tube;
- temperature during centrifugation;
- acceleration and deceleration (brake) settings.

### 4.3 Density gradient/cushion

Density gradients or cushions can be used to separate certain NVEPs and proteins from EVs based on the characteristic densities of different classes of EPs (Raposo et al., 1996). Gradients are prepared of layers consisting of different ratios of a selected dense medium (like sucrose, iodixanol or iohexol) and aqueous buffers, with density decreasing from bottom to top of the gradient, whereas cushions consist of a homogeneous layer of dense material below an aqueous column. EV‐containing materials can be loaded beneath a gradient (‘bottom‐up’) or onto the top of a gradient or cushion (‘top‐down’) and then ultracentrifuged. In the bottom‐up approach, the EV‐containing preparation is mixed with high‐density medium, loaded at the bottom of a centrifuge tube, and overlayed with layers of decreasing density; the preparation may also be underlaid under a prepared gradient. As ultracentrifugation proceeds, particles that are less dense than the surrounding medium float upwards. With sufficient time, particles will ultimately reach a density fraction corresponding to their buoyant density. Since smaller EVs travel at a relatively slower rate than larger EVs, especially in viscous media, the bottom‐up approach in velocity sucrose density gradient UC can also be used to separate EVs according to size (Aalberts et al., 2012). In top‐down settings, the EV‐containing preparation in a low‐density medium is loaded onto the top of a gradient or cushion: for gradients, particles travel into the gradient at a rate corresponding to their density and size until their equilibrium buoyant density is reached; for cushions, particles that reach the cushion remain at the interface if less dense than the cushion material but continue into and through the cushion if they are denser. The cushion approach is thus easier to implement but separates EPs by a threshold of density. Importantly, for gradients, lengthy ultracentrifugation may be needed for optimal separation [e.g., longer than 48 h (Aalberts et al., 2012; Palma et al., 2012)], but shorter spins may suffice for some applications [e.g., 1–2 h (Kowal et al., 2016), 16 h (Aalberts et al., 2012; Liao et al., 2019)].

Following separation by gradient, fractions must be collected carefully to avoid disrupting the gradient. It is good practice to confirm density of final fractions, for example, by weighing given volumes or measuring refractive index. Before performing most downstream assays, the density medium must be removed. This can be done, for example, by diluting the fractions with buffer and ultracentrifuging, or by using SEC. Recovery after density gradient and fraction washing is relatively low.

**Summary: density gradients and cushions**

- Can be implemented in different settings (top‐down, bottom‐up) depending on the aim, that is, to separate EVs from proteins, or from NVEPs, or to separate EV subtypes.
- Leads to low recovery of high‐purity material (based on density).

**Reporting recommendations**. For density gradients and cushions, report the following:

- density material, buffer composition, and exact method of gradient/cushion preparation;
- volume and concentration of material loaded, as well as method of loading onto or at the bottom of the column;
- exhaustive description of centrifugation parameters (same as for dUC);
- details of collection procedure, final densities of fractions (where relevant), and washing.

### 4.4 Size exclusion chromatography

SEC separates nanoparticles including EVs based on size (Boing et al., 2014; Karimi et al., 2018). In SEC, a sample is placed onto the top of a column loaded with a matrix that contains passages with defined pore size. Driven by gravity or by pressure from a pump, larger particles pass through the matrix quickly, without entering the pores, and can be collected as early fractions, while smaller particles (smaller than the matrix pore size) are retained longer and elute predominantly in later fractions. Certain SEC matrices allow separation of EV‐sized particles (EVs, viruses, larger lipoprotein particles) from small NVEPs and free proteins.

Variables that affect the degree of separation by SEC include the matrix composition and pore size, column packing method, the ratio of column length to diameter (or volume), flow rate (gravity vs. defined pressure), and applied sample concentration and volume. Size exclusion columns can be home‐made or purchased. Commercial columns are often packed under strictly controlled conditions and may allow more reproducible results than home‐made columns. Abundance and purity of EVs and other NVEPs in collected fractions must be established through careful characterisation, as for all other methods. SEC dilutes the sample, increasing volume compared with the input material, so concentration of a sample before or after SEC may thus be needed. SEC size separation can be combined with affinity methods by modifying the matrix. The related method of bind‐elute chromatography combines size‐based separation with selection by charge or molecular affinity and permits a single elution (with retention of unwanted materials) that may be amenable to high‐throughput separations, for example, in multi‐well plates. In some cases, SEC matrices can be reused after thorough cleaning.

**Summary: SEC**

- SEC is an easily accessible technique for size‐based separation of particles.
- Columns can be packed with a variety of matrices and at different scales, depending on desired capacity and resolution of separation.

**Reporting recommendations**. For SEC, report the following:

- type of matrix and pore size; height and diameter (or volume) of matrix‐containing column;
- method of column packing (or source of commercially‐available columns);
- source, volume and particle concentration of pre‐SEC sample, including any prior separation/concentration steps;
- buffer composition;
- specify gravity flow or pressure. If pressure, indicate pump system and pressure parameters;
- void volume and numbers and volume of fractions collected;
- any post‐SEC concentration methods;
- if columns are re‐used, method of column regeneration and number of times the column has been used.

### 4.5 Fluid flow‐based separation

Fluid flow‐based techniques separate EVs and other particles based on one or more particle properties, but without relying on a ‘matrix’ or stationary phase. The two main categories of flow‐based techniques currently used in EV studies are field‐flow fractionation (FFF, Giddings et al., 1976) and free‐flow electrophoresis (FFE, Preußer et al., 2022). These techniques can be applied to highly heterogeneous input materials, and the absence of a solid phase allows high particle recovery. They are also among the gentlest of separation methods and may thus be used to study molecules that are loosely associated with EVs.

The most prevalent FFF approach in EV studies is asymmetric flow FFF (AF4, Sitar et al., 2015), in which particles in a sample are transported by fluid flow with a parabolic pattern through a long, thin channel, while a field perpendicular to the direction of transport tends to concentrate particles against the bottom of the channel. Smaller particles, diffusing more rapidly, are more likely to enter the higher‐velocity flow regions in the middle of the channel, and particles are thus separated by hydrodynamic size. Some degree of purification may also be achieved by a channel bottom consisting of a molecular weight cut‐off filter. Although AF4 can precisely separate particle populations with small differences in size (Hood et al., 2014; Zhang et al., 2018), it is not specific to EVs in its standard configuration. AF4 is also not a preparative technique. In contrast, FFE combines flow with electrophoresis, adding separation by, for example, isoelectric point (Preußer et al., 2022). Introducing separation buffers with different pH or other characteristics across the separation channel allows high‐resolution separation of different EV and other EP populations. FFE can be done at various scales.

**Summary**:

- Fluid flow‐based separations such as AF4 can achieve size‐based separations with high resolution.
- Lacking a solid phase and with limited applied forces, flow is gentler than most EV separation techniques.
- Size‐based separation can be combined with separation by other principles by applying different types of fields.
- Preparative scales can be reached with flow techniques such as free‐flow electrophoresis.

**Reporting recommendations**. For flow‐based separations, report the following:

- all instrumentation, including pumps and collection devices;
- composition of all buffers and their filtration. Especially in FFE: how properties of the buffers were confirmed;
- all field characteristics such as flow rates and pressures, pH gradients, electric field;
- dimensions and composition of separation chambers, including any molecular weight cutoff plates;
- all relevant details of fraction collection.

### 4.6 Charge and molecular recognition‐based separations

The common principle of all affinity methods is to capture EVs based on their surface charge or molecular composition. Ion‐exchange chromatography takes advantage of a very simple affinity of particles/surfaces of opposite charge: EVs and/or NVEPs have affinity for a matrix based on negative (anion‐exchange) or positive (cation‐exchange) surface charge (Saari et al., 2023). In contrast, the term ‘affinity separation’ as commonly used in molecular biology refers to methods that harness the specific recognition of one macromolecular complex for another. In this context, affinity probes include heparin and various lectins (which bind glycans, Balaj et al., 2015); specific full‐length proteins with affinity for a particular lipid or protein (e.g., Tim4 for phosphatidylserine, (Nakai et al., 2016); peptides that bind specific EV surface proteins (Bai et al., 2014; Gao et al., 2018; Gobbo et al., 2016; Joy et al., 2018; Liu et al., 2019; Pham et al., 2021; Suwatthanarak et al., 2021) or the membrane more generally (Gori et al., 2020; Ishida et al., 2020; Yang et al., 2022), including curvature‐sensing peptides that select for EVs in certain size ranges (Saludes et al., 2012); aptamers [short single‐stranded DNA or RNA molecules that are developed to bind specific targets (Zhang, Yue et al., 2019)]. Antibodies that are raised to recognise specific EV surface molecules are the most commonly used affinity reagents, and their most‐used targets are the tetraspanins (Kowal et al., 2016; Mathieu et al., 2021).

In molecular recognition‐based affinity approaches, the EV‐containing fluid (which may have first been concentrated according to Section 4.1) is introduced to affinity probes before or after the latter are bound to a matrix, such as a membrane or beads. Beads, in turn, can be placed in a column or tube to facilitate binding and washing. Molecular target‐displaying materials are bound by affinity probes to the matrix (‘pull‐down’), while unbound material flows away (‘flow‐through’). Non‐specifically bound materials may be removed by one or more washes. If EVs are the intended target, detergent should not be present in the dilution and washing buffers unless at very small concentrations (0.001% or less) to minimise non‐specific binding to the capturing matrix or between EVs. To evaluate the efficiency and specificity of recovery of the targeted EVs, it is recommended, at least during protocol development, to compare side‐by‐side the flow‐through and the pull‐down by biochemical analyses, measuring the affinity motif and a few EV markers (see Section 5).

Bound EVs can be dissociated from the matrix and recovered by a variety of techniques, ranging from changing the properties of the buffer, to adding an excess of target molecules (e.g., sugars or lipids), to eliminating factors required for efficient binding (e.g., using EDTA to chelate calcium). However, some affinity reagents may bind tightly to their EV target and require removal by, for example proteases. In some cases, the EV‐binding molecule and/or the matrix may be recovered together with the EVs. This may not be an issue if downstream analyses are not affected by these materials (e.g., nucleic acid analysis of EVs contaminated with a protein‐based affinity probe), but it may be in other cases, (e.g., in functional uses, since the EV surface is modified by an EV‐binding molecule). Antibodies are particularly difficult to separate from EVs. Low pH treatment classically used to separate antibodies and antigens will likely affect the structure of EVs, and protease treatments may also digest proteins on the surface of the EVs.

In any molecular affinity approach, it is important to understand the degree to which the target molecule is associated with EVs versus NVEPs, or with one EV subtype versus others, and to assess the specificity of the capture reagent. For example, the use of CD9 or CD63 affinity capture for urinary EVs (uEVs) excludes uEVs from cells of the proximal nephron (Blijdorp et al., 2021; Limbutara et al., 2020). In another example, the literature on L1CAM affinity (a putative neuronal EV marker) has developed substantially since MISEV2018. Although L1CAM has been investigated as a membrane‐associated antigen to separate putative neuronal EVs from peripheral blood samples, it has more recently been described as being in a cleaved, mostly soluble form in certain EV sources (Norman et al., 2021). It is also found on EVs from a variety of sources, not just neurons, and a widely‐used anti‐L1CAM antibody might also recognise other targets (Gomes & Witwer, 2022; Norman et al., 2021).

**Summary: charge and molecular recognition affinity‐based methods**

- Separate components of EV preparations according to surface charge or exposure of a specific molecular determinant.
- Will co‐isolate all EV subtypes or NVEPs which expose a given charge or molecular determinant: specificity and recovery depend on the specificity versus universality of exposure of the chosen molecular determinant.
- Antibody‐based affinity separation leads to co‐isolation of the determinant‐exposing EPs with the antibody and/or isolation beads.
- Efficiency and selectivity must be quantified when establishing the protocol by quantifying material recovered in pull‐down versus flow‐through.

**Reporting recommendations**: for affinity‐based separation, report the following:

- molecule used as affinity probe (nature, source);
- matrix (beads, gel, column);
- incubation times;
- buffer and number of washes;
- elution process (such as elution buffer composition, time).

### 4.7 General considerations and caution on kit‐based approaches

Due to overlapping biophysical characteristics of EPs (Geeurickx et al., 2019; Karimi et al., 2018) and the abundance of many NVEPs in various EV sources, complementary separation techniques are increasingly applied sequentially (Benedikter et al., 2017; Stam et al., 2021; Zhang, Borg, et al., 2020) (arrows in Figure 2), which allows increased specificity. Examples of methods used to separate EVs from protein aggregates and other NVEPs include size exclusion chromatography, density gradient ultracentrifugation (Jeppesen et al., 2019), asymmetric flow field‐flow fractionation (AF4) (Zhang et al., 2018) and ultra‐high‐speed ultracentrifugation (Zhang, Higginbotham, et al., 2019; Zhang et al., 2021). However, some of these studies suggest that several proteins previously proposed to be sEV markers are equally, if not more, abundant in NVEPs, thus calling for re‐evaluation of the achieved EV selectivity.

Conversely, a growing realization since MISEV2018 is that some molecules that co‐isolate with EVs, including proteins, nucleic acids, sugars, and lipids, could be viewed not as ‘contaminants’, but rather as a part of a dynamic EV ‘corona’ (Buzas, 2022; Palviainen et al., 2020; Tóth et al., 2021). Molecules and even biological nanoparticles such as lipoproteins (Busatto et al., 2022; Sódar et al., 2016) may adsorb to the EV surface where they may serve as biomarkers or contribute to EV function (Musicò et al., 2023; Radeghieri et al., 2022). The EV corona may be removed in part or in full by separation processes including dUC and SEC (Singh et al., 2020; Wolf et al., 2022). Ongoing studies of the EV corona may change how we view contaminants and the perceived need for highly pure EVs; this point is also relevant for the next MISEV section, on EV characterization.

Only methods using readily (i.e., commercially) available devices and instruments are described in this section. However, new developments of separation methods, including those involving equipment built in individual laboratories, are occurring constantly and are strongly encouraged by ISEV. When establishing a new separation/concentration workflow, a good practice is to assess the extent of EV separation/concentration with methods discussed in Sections 5 and 6 and with careful and complete book‐keeping. Comparing the results with those of another already established method is also recommended. For example, EV marker proteins can be tracked and related to total isolated protein to determine fold enrichment over total protein reported and account for EV marker losses (Geeurickx et al., 2019; Zhang, Borg, et al., 2020). Results will indicate recovery and degree of enrichment and will also show whether the separated EV population is representative of the original population or has been selectively obtained.

Finally, some cautionary notes on commercial kits. Numerous kits are advertised as obtaining specific types of EVs (usually ‘exosomes’) or EVs from specific types of sources. These kits may or may not achieve EV separation or concentration based on a variety of principles, including polymer precipitation, membrane affinity, antibody capture and filtration. These kits may be helpful under certain circumstances, but EV researchers should be aware of several major caveats. Kits that do not disclose details of the principles of EV separation/concentration may produce results that are difficult to interpret, not least because they may introduce unknown contaminants (e.g., polyethylene glycol for some polymer precipitation kits). Extra work may be needed to compare these methods with results from other techniques and to place the results on the recovery/specificity grid (Figure 2) for better interpretation. Precipitation‐based kits in particular will concentrate all EPs in a mixture, even many free proteins, resulting in a highly impure preparation, especially from complex, NVEP‐rich sources such as blood plasma and serum. Use of such kits is strongly discouraged unless for volume reduction alone (Gámez‐Valero et al., 2016; Karttunen et al., 2019; Lobb et al., 2015; Paolini et al., 2016). In contrast, affinity‐based methods may isolate only subtypes of EVs, and the specificity of the affinity reagents may be difficult to assess if the exact reagents are not disclosed. Generally, kits that disclose contents and principles should be preferred over kits that make unsubstantiated claims (e.g., ‘exosome’ isolation) and do not provide details.

**Recommendations**
  - If separation/concentration is not done, indicate why. Otherwise, justify why each separation/concentration method was selected in terms of yield and specificity.
  - Provide sufficient methodologic detail to allow replication of each separation and concentration step.
  - Report any measurements that are used to assess the separation/concentration process(es). Where applicable and feasible, and especially when establishing a new workflow, check EVs before and after each step. For example, track EV marker protein levels relative to total protein to estimate fold enrichment and yield for each step.
  - For affinity‐based EV separation approaches, establish molecular specificity of reagents and EV/EV subtype‐specificity of all targeted markers.
    Consensus: 74.4% (743) of MISEV2023 survey respondents agreed “completely,” and 24.8% (248) agreed “mostly” with Section 4: EV separation and concentration. 0.1% (1) “mostly” disagreed, and 0.6% (6) stated that they had no opinion and/or expertise. No respondents disagreed “completely.”

## 5 EV CHARACTERIZATION

EV characterization is needed for estimation of EV quantity, to establish the presence of EVs, and to assess the contributions of non‐EV components to an EV preparation. Characterization is challenged by small particle size, heterogeneity of EV size and molecular heterogeneity, a lack of universal EV identification methods, and the non‐EV‐specificity of many measurement techniques. As a result, no single measurement or method is able to satisfy all EV characterization requirements, and use of orthogonal methods (those that do not have the same measurement limitations) is recommended.

If making claims about an EV preparation, the extent to which a sample will need to be characterised to justify the claims may depend upon the source of the material (see Section 4). This may mean additional characterization steps are needed with different samples and may also mean that additional reporting information is required to allow the influence of other preanalytical variables on EVs to be assessed.

Overall EV composition (contribution to total mass of proteins, lipids, nucleic acids and other biomolecules) varies by EV source. While measurement of these individual molecular classes can be used to estimate EV abundance, these values do not necessarily perfectly correlate with EV concentration, nor is there universality across source materials; thus, they should not be used as a sole measure of EV concentration.

Just as no single molecular class measurement can quantify all EVs, there are also no universal molecular markers of EVs or EV subtypes. Markers must be chosen based on source‐ and type‐specific evidence. Currently, no generic marker is known to identify all EVs irrespective of source. Although several proteins have been proposed as putative markers of EV biogenesis pathways (e.g., Annexin A1 (Jeppesen et al., 2019), SLC3A2, and BSG (Mathieu et al., 2019) for purported ectosomes, and Lamp1 (Mathieu et al., 2021)) for purported exosomes, the universality of these markers is not yet clear or accepted. Note that affinity‐based protocols involving the tetraspanins CD9, CD63 and CD81 are not specific for exosomes as an EV subtype; using antibodies to each of these tetraspanins enriches EV populations that do not completely overlap in molecular composition (Kowal et al., 2016; Mathieu et al., 2021). Additionally, not all EVs display these tetraspanins, therefore tetraspanin enrichment does not capture all EVs.

Orthogonal methods measurements of the same parameter are unlikely to have the same biases; for example, the derivation of diameter from optical versus non‐optical methods. Characterization of EV samples using orthogonal methods is critical to provide evidence that co‐isolates are not responsible for biomarker or functional findings. Due to many EV characterization methods being either not EV specific or unable to detect all EVs, transparent reporting of methods and results is needed for reproducibility of EV data. A framework for reporting EV data has been previously developed and updated in the form of EV‐TRACK (EV‐TRACK Consortium et al., 2017; Roux et al., 2020). Standardization of EV characterization has been supported by ISEV workshops, the ISEV Rigor and Standardization Task Forces, and ISEV position papers (Clayton et al., 2018; Nieuwland et al., 2020; Welsh, Van Der Pol, Arkesteijn, et al., 2020).

In the following sections different approaches to EV characterization are discussed, with each section providing recommendations if that characterization approach is taken. Overall recommendations for characterization, regardless of the method, are summarised below.

**Recommendations**
  - Each EV preparation should be defined by quantitative measures of the source of EVs (e.g., number of secreting cells, volume of biofluid, mass of tissue).
  - Approximations of the abundance of EVs should be made (particle number, protein, and/or lipid content).
  - EV preparations should be tested for the presence of components associated with EV subtypes or EVs generically, depending on desired specificity one wishes to achieve.
  - Establish the degree to which non‐vesicular, co‐isolated components are present.
  - Provide an indication of the instrument/method limit of detection (LOD) when EVs are characterised with quantitative metrics.

### 5.1 Quantification of particle number concentration

EV number can be used along with volume measurement to define the number concentration (in particles/mL), a metric that is widely reported and used for assay input standardization, assay output measurements, and in vivo dosing. However, it is often unreliable, since many techniques lack specificity for EVs and sensitivity for all EVs.

The ISEV Rigor and Standardization EV Reference Material Task Force recently outlined the considerations in measurement techniques, along with the challenges faced by the field in moving towards traceable measurements, for the development and reporting of well‐characterised EV reference materials (Welsh, van der Pol, Bettin, et al., 2020). A key highlight of this work is the need to report assay LOD, allowing others to validate findings irrespective of the sensitivity limit. Note that reported EV concentration in blood plasma spans six orders of magnitude depending on the measurement method (Johnsen et al., 2019). Greater confidence in EV concentration measurements may be achieved by using orthogonal methods, each with defined LODs, for example, detecting light scattering intensity, fluorescence intensity and physical size, since orthogonal methods do not share the same measurement limitations (Arab et al., 2021; Silva et al., 2021). For example, for resistive pulse sensing (RPS) techniques that are calibrated with size‐standards, a LOD can be reported in diameter. The lower LOD for RPS will most likely be due to sensitivity limitations, while the upper LOD will be influenced by the pore size. For optical techniques such as flow cytometry, the LOD may be reported in diameter, derived from light‐scattering optical models, or molecules of equivalent soluble fluorophore (MESF), derived from fluorescence intensities. These approaches result in concordant data across instruments and sensitivities (Welsh, Van Der Pol, Arkesteijn, et al., 2020; Welsh, Jones, et al., 2020; van der Pol, Sturk, et al., 2018). Currently, there is no method to derive a traceable LOD for nanoparticle tracking analysis (NTA), DLS, or imaging flow cytometry, due to the number of variables involved in particle detectability. Techniques that output concentration measurements without any phenotypic characterization, such as the use of membrane dyes, can lead to overestimation of EV concentration due to dye self‐aggregation and an inability to differentiate between EVs and other co‐isolates (Takov et al., 2017). A membrane dye lacking these problems could lead to underestimation unless it universally stained all EVs, irrespective of composition and derivation, and such a dye has not yet been reported. Further instrument and assay‐specific recommendations can be found in Section 6.

For techniques that cannot differentiate EVs from other potential co‐isolates or suspension contaminates, it is recommended that concentration be reported as ‘particle or EP concentration’ and not ‘EV concentration’, regardless of upstream separation steps.

**Recommendations**
  - Report the LOD of each assay, or state that it is not quantifiable or known.
  - Where possible, report data from dilution series to demonstrate that concentration derivations were in the linear region of system measurement.
  - Where possible, use orthogonal methods to determine particle number.
  - Unless methods are highly specific for EVs, the output of these measures should be described as pertaining to ‘particles’ or ‘EPs’.

### 5.2 Quantification of particle size

Measurements of EV size (in nm radius or diameter) rely on assumptions, such as of sphericity or mobility, and output can be influenced by upstream variables (Tian et al., 2020). Common high‐throughput methods assume that EVs are spherical. These include flow cytometry, NTA, RPS, multi‐angle light scattering and dynamic light scattering (DLS). While ‘size’ and ‘diameter’ are often used interchangeably between measurement methodologies, the way in which they are derived can also result in consistent differences in measurement techniques. For example, techniques relying on the mobility of particles, such as NTA or DLS, measure hydrodynamic diameter, resulting in an overestimation of size compared with an imaging method such as cryo‐EM (Chernyshev et al., 2015; Skliar et al., 2018). Few if any methods are able to measure EV size accurately throughout the entire possible EV diameter range, from tens of nanometres to microns. For example, while high‐resolution imaging by cryo‐EM is one of the most accurate methods (Yuana et al., 2013), it is relatively low‐throughput, and many larger EVs that tend to be orders of magnitude less abundant may not be quantified. The ability to quantify low contrast EVs below 100 nm may also be a limiting factor.

As more researchers use dedicated single‐particle techniques with increased sensitivity, it is becoming increasingly clear that many EV preparations display an asymmetric right‐skewed distribution, for example, a log‐normal distribution, with the majority of EVs <100 nm in diameter (Bachurski et al., 2019; Dong et al., 2020; Lennon et al., 2019; Tian et al., 2020, 2018; van der Pol, Coumans, Grootemaat, et al., 2014). Most single‐particle analysis techniques cannot resolve the full population of EVs, so the detected EV diameter distribution should be shared, not just a summary metric such as mean, mode, or median size, which can be easily skewed depending on the LOD and the asymmetric size distribution (Welsh, van der Pol, Bettin, et al., 2020). Be aware that the modal size statistic, as measured, for example, by NTA for low refractive index particles, may better approximate the instrument LOD than the true modal diameter of the EV population (Bachurski et al., 2019). Techniques using software with proprietary algorithms to determine particle diameter may also result in variation between software versions or software platforms (van der Pol, Coumans, Grootemaat, et al., 2014); software and version should therefore be reported. Techniques deriving size from refractive index assumptions may result in variation due to differing compositions and cargo. Derivation of size from fluorescent probes, such as membrane intercalating dyes, may result in variation due to different dye intercalation based on different membrane lipid compositions. For techniques that cannot differentiate EVs from co‐isolates/contaminants, it is recommended that diameter be reported as ‘particle’ or ‘EP’ diameter and not ‘EV diameter’, regardless of upstream separation steps.

**Recommendations**
  - Where possible, make orthogonal measurements to increase confidence in size distribution.
  - EV diameter distribution of a population should be shared, not just mean, mode, or median.
  - Consider the LOD of the method chosen and how this may influence the data.
  - Report instrument settings, software platforms and versions and possible influence of measurement reagents, especially intercalating dye.

### 5.3 Quantification of total protein

Total protein (in μg, or μg/mL for concentration) in an EV preparation can be approximated by colorimetric assays, fluorometric assays, global protein stain on SDS‐PAGE, or absorbance readings, each with differing sensitivities and accuracies (Vergauwen et al., 2017). As a bulk analysis technique, total protein quantification often overestimates EV concentration due to co‐isolated protein, especially for less specific methods of EV separation or complex biofluids. Conversely, highly purified, low‐yield EV preparations may challenge assay sensitivity. Since measured protein concentration may vary depending upon whether intact or disrupted EVs are measured, details of physical disruption and the nature and concentration of any detergent should be indicated.

Protein concentration as a surrogate of EV concentration should be used with caution and is generally not recommended, as enrichment of some proteins per EV may occur with different cellular phenotypes or stimulations. Since protein:particle ratios also depend on the LODs lower concentration limit of detection or lower size limit of detection of each assay/instrument, it is recommended to provide absolute protein and particle concentrations separately if ratios are reported.

**Recommendations**
  - Report output by ‘particles’ or ‘EPs’ unless evidence of upstream processing is highly specific for EVs.
  - Report the lower concentration limit of detection of each assay to facilitate interpretation.
  - For ratios, report the original constituent measurements, not just the ratio.
  - Protein concentration should be within the linear range of the reference curve, which should also be reported.
  - Report whether intact or disrupted preparations are used.

### 5.4 Quantification of total lipids

Total lipid quantification of EV samples can be achieved by colorimetric assays (Visnovitz et al., 2019), fluorescence of membrane intercalating dyes, total reflection Fourier‐transform infrared spectroscopy (FTIR) or chromatography (Mihaly et al., 2017). However, intercalating dye methods and FTIR may be insufficiently sensitive for small amounts of EVs, and some methods require highly specialised equipment. It remains unknown whether these techniques detect all EVs independent of lipid composition. Total lipid measurements may overestimate EVs due to co‐isolated NVEPs such as lipoproteins.

**Recommendations**
  - Consider the LOD of your assay.
  - Consider how co‐isolated NVEPs may influence your measurement.

### 5.5 Quantification of total RNA

RNA is a frequently studied EV‐associated molecule (see Section 6.5), so basic characterization of EV preparations may include total RNA quantification as a quality control component or for normalization in profiling and functional studies. Quantification of total EV RNA can be done by capillary electrophoresis and other methods. However, using total RNA as a surrogate for EV concentration or purity is difficult to recommend due to vastly more abundant extra‐EV RNA in many EV sources. Some methods of RNA quantification do not distinguish between RNA and DNA. Isolation kits have also been demonstrated to influence downstream results (Eldh et al., 2012). An early ISEV RNA position paper recommended the use of sensitive techniques such as Agilent Bioanalyzer pico chip or Quant‐iT RiboGreen RNA Assay for EV RNA quantification over less sensitive methods such as NanoDrop (Hill et al., 2013). However, several nucleic acids dyes, such as RiboGreen, are not specific for RNA over DNA. Additionally, small RNAs require specialised Bioanalyzer kits. Other sensitive methods include the Qubit microRNA Assay kit, which has sensitivity for small RNAs. Pre‐treatment with RNase‐free DNase may be useful for accurate RNA quantification since many techniques are sensitive to DNA contamination. However, DNase treatment may not completely remove all DNA contamination (Verwilt et al., 2020).

**Recommendations**
  - Consider the ability of your assay to discriminate between RNA and DNA, and the limits of detection of your chosen method.
  - Report any enzymatic pre‐treatments of the sample, for example, with DNase.

### 5.6 Characterization of EV morphology

EV morphology is currently best assessed for smaller EVs using high‐resolution imaging techniques such as: scanning electron microscopy (SEM) (Cavallaro, Hååg, et al., 2021) , transmission electron microscopy (TEM) (Théry et al., 2006), cryo‐EM (Arraud et al., 2014; Stoner et al., 2016; Wu et al., 2015); and scanning‐probe microscopy (SPM), including atomic force microscopy (AFM) (Sharma et al., 2011). EVs that are much larger than the light diffraction limit (≳200 nm diameter) might be assessed by conventional light microscopy. These techniques are not necessarily interchangeable or capable of attaining comparable image quality. For example, desiccated conditions may cause EVs to form an artefactual cup shape, not seen under hydrated conditions. Imaging techniques may allow assessment of EV purity, at least at the particle level, if they can visualise co‐isolated NVEPs equally well. Imaging techniques are often limited by low throughput and the potential for bias based on field‐of‐view selection (Rikkert et al., 2019).

**Recommendations**
  - Irrespective of imaging technique, report all experimental details. These include the instrument, software version, acquisition and analysis settings, sample preparation processes, how the imaged areas were selected, and controls and calibration information where relevant. Further details can be found in Sections 6.4.1 and 6.4.4.

### 5.7 Characterization of EVs by protein composition

Because of the heterogeneity of EVs, MISEV2023, like MISEV2018, cannot recommend molecular markers of specific EV subtypes. MISEV2023 recommends the five‐component framework introduced in MISEV2018 for reporting claims about the protein content of EVs (Table 3). Categories 1 and 2 assess the presence of EVs features. Category 3 assesses purity from common contaminants. Categories 4 and 5 provide additional information on possible intracellular origins of EVs (4) or co‐isolates (5). Ideally, enrichment or depletion of markers in EV preparations versus unfractionated source material should be shown. To avoid perceived restrictions on which EV proteins should be analysed, MISEV2023 gives only a few nominative examples (Table 3). Other putative marker proteins can be assigned to one of the categories using databases such as Uniprot (https://www.uniprot.org/), where the section ‘Subcellular location’ indicates subcellular compartments or extracellular location, and ‘features’ indicates topological and transmembrane domains. Although these categories apply to EVs regardless of analysis method, some of these markers may not be technically usable in some single‐EV analysis techniques, which may require other controls. A variety of methods exist to determine the presence of protein markers. The sensitivity, specificity, and reliability of these methods can vary. Current assay‐ and instrument‐specific reporting considerations are outlined in Section 6.

**TABLE 3:** Protein content‐based EV characterisation.

Category				
**1‐ Transmembrane (or GPI‐anchored) proteins associated with plasma membrane and/or endosomes** All EVs Non‐exhaustive examples, categorized a, b, c: by decreasing strength of membrane association.	**2‐ Cytosolic proteins in EVs** All EVs	**3‐ Major components of non‐EV co‐isolated structures (NVEPs)** All EVs as purity control	**4‐ Transmembrane, lipid‐bound and soluble proteins associated with intracellular compartments other than PM/endosomes** Subtypes of EVs and/or pathologic/atypical state, and/or novel separation method	**5‐ Secreted proteins recovered with EVs** Corona or functional component of EVs
**1a: multi‐pass transmembrane proteins**. Tetraspanins (CD9, CD63, CD81, CD82); other multi‐pass membrane proteins (CD47; heterotrimeric G proteins GNA*, TSAP6)	**2a: with lipid or membrane protein‐binding ability**. ESCRT‐I/II/III (TSG101, CHMP*) and accessory proteins: ALIX (PDCD6IP), VPS4A/B; ARRDC1; Flotillins (FLOT1/2); caveolins (CAV*); syntenin (SDCBP)	**3a: lipoproteins**. Produced mostly by liver, abundant in plasma, serum. Apolipoproteins	**4a: nucleus**. Histones (HIST1H**); Lamin A/C (LMNA/C)	**5a: blood‐derived corona proteins**. Partially overlapping with 3a/3b: apolipoproteins, complement, fibrinogen
**1b: single‐pass transmembrane proteins**. Major Histocompatibility Class I or II, Integrins (ITGA*/ITGB*), transferrin receptor (TFR2); LAMP1/2; heparan sulphate proteoglycans including syndecans (SDC*); EMMPRIN (BSG); ADAM10	**2b: promiscuous incorporation into EVs (and possibly NVEPs)**. Heat shock proteins HSC70 (HSPA8), and HSP84 (HSP90AB1) note that both are abundant also in NVEPs; cytoskeleton: actin (ACT*), tubulin (TUB*); enzymes (GAPDH)	**3b: protein and protein/nucleic acid aggregates**. Immunoglobulins (blood); Tamm‐Horsfall protein (Uromodulin/UMOD; urine); albumin. YWAH* (14‐3‐3*) and AGO* (can be present in EVs but generally more abundant in NVEPs).	**4b: mitochondria**. VDAC, cytochrome C (CYC1); TOMM20	**5b: cytokines and growth factors**. e.g., TGFB1/2; IFNG, VEGFA, FGF1/2, PDGF*, EGF, interleukins (IL*)
**1c: GPI‐ or lipid‐anchored proteins**. Glypicans (GPC1), 5ʹnucleotidase CD73 (NT5E), complement‐binding protein CD59		**3c: exomere or supermere‐enriched components**. HSP90AA/B, TGFBI, HSPA13, LDHA/B	**4c: secretory pathway**. Endoplasmic reticulum, Golgi apparatus: calnexin (CANX); Grp94 (HSP90B1); BIP (HSPA5), GM130 (GOLGA2)	**5c: adhesion and extracellular matrix proteins**. Fibronectin (FN1), Collagens (COL**), MFGE8; galectin3‐binding protein (LGALS3BP), CD5L; fetuin‐A (AHSG)
			**4d: others**. Autophagosomes, cytoskeleton… LC3 (MAP1LC3A), Actinin1/4 (ACTN1/4)	

At least one protein of categories 1, 2 and 3 should be analysed as EV hallmarks and to assess the presence of NVEPs in an EV preparation. Analysis of proteins of category 4 is optional, as they may be present in some subtypes of EVs, or under certain conditions, with no general rule. Proteins of category 5 may bind to EVs after their release and may be part of the recently described EV ‘corona’. ***Please note that this table provides a limited number of examples only*** for proteins commonly found in mammalian cell‐derived EVs. Other proteins that fall into the given categories may be equally valid, particularly for analysis of EVs from prokaryotic (bacteria) or non‐mammalian eukaryotic sources (including parasites and plants). For most proteins of interest, their subcellular location in intracellular compartments (for categories 1 and 4), or their transmembrane or lipid‐anchored nature (for categories 1 and 2), is provided in the Uniprot database (www.uniprot.org). *XX* = human gene names. XX* or XX** used for families of multiple proteins, for example, for integrins: ITGA* indicates any integrin alpha chain.

**Recommendation**

- Utilise the five‐component framework (Table 3) for reporting claims about EV protein content.

### 5.8 Non‐protein markers of EVs

Non‐protein markers, such as phosphatidylserine, glycans or specific nucleic acids, are seldom EV‐specific but in some cases may add support for the presence of a lipid bilayer or cytosolic components. Co‐localization with protein markers may also provide stronger evidence for the presence of EVs, for example, a membrane‐intercalating dye and a tetraspanin‐positive event, especially for single‐particle measurements. Non‐protein markers may be detected directly with techniques such as lipid mass spectrometry or Raman spectroscopy (Section 6.7), or indirectly using fluorescent probes such as membrane labels or intraluminal dyes. Recommendations for the reporting of EV labelling with non‐protein markers is outlined in Section 6.6. The non‐EV‐specificity of most non‐protein component markers urges caution. Membrane dyes may complex with any lipids, including those of NVEPs; dyes that are activated by intraluminal enzymes such as esterases may not be present in all EV preparations or subtypes; nucleic acid dyes have been used for EVs, but recommendations on controls and specificity are still needed (Liu et al., 2022).

**Recommendations**:
  - If non‐protein markers are used, consider using protein colocalization.

### 5.9 Localization of EV‐associated components

EV‐associated components such as proteins, nucleic acids and glycans, may be luminal, in the membrane, or external to the EV. Knowledge of topology may be important for understanding the biology. For example, must an EV fuse with a recipient cell to deliver a luminal cargo, or can the EV simply present a surface‐associated molecule to a receptor? The location of putative active components should therefore be determined by performing mild digestions, permeabilizations or affinity reagent accessibility by adopting or adapting previously published methods (Bonsergent & Lavieu, 2019; Cvjetkovic et al., 2016; Lai et al., 2015; Mateescu et al., 2017; McKenzie et al., 2016; Osteikoetxea et al., 2015; Sharma et al., 2010; Sung & Weaver, 2017).

**Recommendations**:
  - Consider how topology can be determined in method design.
    Consensus: 72.3% (722) of MISEV2023 survey respondents agreed “completely,” and 27.0% (269) agreed “mostly” with Section 5: EV characterization. 0.3% (3) “mostly” disagreed, and 0.4% (4) stated that they had no opinion and/or expertise. No respondents disagreed “completely.”

## 6 TECHNIQUE‐SPECIFIC REPORTING CONSIDERATIONS FOR EV CHARACTERIZATION

As utilization and expertise has expanded across a broad range of EV detection assays and instrumentation, the identification of pertinent reporting criteria has also grown to ensure reliable and reproducible interpretation of data. A collated list of minimal assay and instrument‐specific reporting considerations are detailed here. These are generally applicable irrespective of experiment design. The techniques listed in the following section are not exhaustive, and many detection technologies are under development or being actively researched. The techniques listed are, however, all commercially available, with existing literature from multiple researchers. These recommendations are not exhaustive, and further criteria are likely required due to subjective experimental parameters.

### 6.1 Flow cytometry‐based methods

#### 6.1.1 Bead‐based flow cytometry

Bead‐based flow cytometry has been used widely to interrogate EV surface proteins. Large beads capture particles regardless of their surface composition (e.g., surfactant‐free aldehyde/sulphate beads) (Théry et al., 2006), or antibody‐conjugated beads capture particles exposing the corresponding antigen. Commercially available EV multiplex kits allow interrogation of 30 or more surface antigens (Koliha et al., 2016; Wiklander et al., 2018). After capture, bead‐associated particles are labelled with a fluorescently conjugated affinity reagent (or mixture of several) for detection. Differences in staining intensity are semi‐quantitative only, since signal derives from multiple particles captured by individual beads. A difference in signal intensity might thus mean different particle concentration, epitope density, diameter distribution or relative abundances of EV subsets.

When reporting bead‐based approaches, controls should include isotypes as detection antibodies, or isotype‐conjugated capture beads, and capture beads with detection antibody alone (for antibody‐coated capture beads). Multiple EV input concentrations may be used to demonstrate titration of signal and rule out non‐specific binding (Wiklander et al., 2018; Welsh et al., 2022). Stained beads as a percentage is not a valid statistic; reporting normalised bead median fluorescence intensities is recommended (Welsh et al., 2022). Reporting data and median fluorescent intensity statistics in molecules of equivalent soluble fluorophore (MESF) (as with single EV flow cytometry) from singlet gated beads is recommended to allow standardization of data across instrument platforms and settings. If preparing beads in‐house, reagents and conjugation chemistry should be reported, while for commercial capture bead reagents, catalogue and lot numbers should be reported. Other reporting parameters include: total bead number, the sample‐bead incubation time, post‐bead incubation wash methodology, detection reagent staining time and post‐staining wash methodology.

**Recommendations**:
  - Controls should include isotypes as detection antibodies, or isotype‐conjugated capture beads, and capture beads with detection antibody alone (for antibody‐coated capture beads).
  - Multiple input EV concentrations should be used to demonstrate titration of signal.
  - If making beads, reagents and conjugation chemistry should be reported. For commercial capture beads reagent catalogue and lot numbers should be reported.
  - Report normalised bead median fluorescence intensities.
  - Report data and median fluorescent intensity statistics in molecules of equivalent soluble fluorophore (MESF) (as with single EV flow cytometry) from singlet gated beads.
  - Report full and detailed methodology.

#### 6.1.2 Single‐EV flow cytometry

Flow cytometry is an optical technique that has demonstrated detection of vesicles down to ∼40 nm in specialised cases (Zhu et al., 2014) and ∼100 nm using many modern conventional cytometers by light scatter and fluorescence (Morales‐Kastresana et al., 2019; Sandau et al., 2020; Stoner et al., 2016; Welsh, Killingsworth, et al., 2021). Through calibration of data, flow cytometry has been demonstrated to be capable of characterising particle diameter (Stoner et al., 2016; Tian et al., 2020; van der Pol, de Rond, et al., 2018; Welsh, Horak, et al., 2020), epitope abundance (Gorgens et al., 2019; Welsh, Jones, et al., 2020), epitope density (Welsh, Jones, et al., 2020), effective refractive index (Pleet et al., 2023; van der Pol, de Rond, et al., 2018) and number concentration within standardised size ranges (van der Pol, Sturk, et al., 2018). In 2023, a tri‐society working group (EV Flow Cytometry Working Group) initiative involving the International Society for Extracellular Vesicles, International Society for Advancement of Cytometry, International Society for Thrombosis & Haemostasis, published a single‐EV flow cytometry compendium to comprehensively address the considerations for developing a single‐EV flow cytometry assay (Welsh et al., 2023).

Calibration of fluorescent and light scatter parameters is critical for interpretation and replication of single‐EV flow cytometry results. If particle concentrations are reported using single‐EV flow cytometry, define the upper and lower LOD to allow replication and interpretation of data using orthogonal techniques. Currently, imaging cytometers use a dynamic triggering method that makes determination of the lower LOD difficult to define and therefore standardise.

In 2020, a comprehensive experiment and reporting framework was developed (MIFlowCyt‐EV) and published as a position paper by the EV Flow Cytometry Working Group (van der Pol et al., 2022; Welsh, Tang, et al., 2021; Welsh, Van Der Pol, Arkesteijn, et al. 2020). The MIFlowCyt‐EV reporting framework is split into categories of: preanalytical variables and experimental design, sample preparation, assay controls, instrument calibration & data acquisition, EV characterization, FC data reporting and FC data sharing. This reporting framework and learning resources for implementing the MIFlowCyt‐EV framework can be found on the EV Flow Cytometry Working Group website (www.evflowcytometry.org). Complete the MIFlowCyt‐EV spreadsheet and attach it as supplementary material for any manuscript with single‐EV flow cytometry. The MIFlowCyt‐EV framework is applicable to all flow cytometers, including conventional, spectral, imaging and single‐photon‐detecting cytometers.

**Recommendations**:
  - Refer to the ISEV‐ISAC‐ISTH MIFlowCyt‐EV framework Position Paper and utilise the reporting framework as supplementary material for any manuscript utilising single‐EV flow cytometry.
  - Ensure correct calibration of volume and fluorescent and light scatter parameters.
  - Define upper and lower limits of detection to allow others to validate your work.

### 6.2 Genetic protein tagging

EV proteins can be genetically labelled by introducing a genetic construct from which a tag, such as GFP, is ultimately co‐translated with a protein or protein domain of interest (Corso et al., 2019; Heusermann et al., 2016; Joshi et al., 2020; Mittelbrunn et al., 2011). The tagged protein may be chosen based on its status as a general EV or EV subtype marker (Section 5.7), and numerous markers have been labelled (Corso et al., 2019; Dooley et al., 2021). Tagged proteins have been used to interrogate EV/subtype release and uptake pathways (Mathieu et al., 2019; Mathieu et al., 2021) and to enable overall biodistribution and pharmacokinetics studies. Importantly, the tag itself or alterations in expression of the tagged protein may affect EV biogenesis (Fan et al., 2020), loading, release, or function, so unlabelled EVs are recommended as a control to assess these possibilities. The fusion protein may also affect subcellular localization or cellular functions. Localization of the chimeric versus wildtype protein should be confirmed. Certain tags (e.g., GFP) may be subject to quenching in acidic compartments (Corrigan et al., 2014). Construct maps should be provided and, where possible, plasmids deposited in Addgene (www.addgene.org) or other repositories.

**Recommendations**:
  - Carefully consider the selection of the tagged protein and its suitability as an EV or EV subtype marker.
  - Assess the subcellular localization and function of the chimeric versus wildtype protein in the cell and the EV by comparing engineered and wildtype cells and labelled/unlabelled EVs.
  - Report construct maps and deposit plasmids with a repository.

### 6.3 Mass spectrometry proteomics

Mass spectrometry (MS) measures mass‐to‐charge ratio of molecules and, in EV studies, is commonly used to detect and characterises EV‐associated proteins in both discovery and targeted applications (Aebersold & Mann, 2003; Hoshino et al., 2020; Pocsfalvi, Stanly, Vilasi, et al., 2016; Sodar et al., 2017). Targeted analyses are typically performed on a triple quadrupole (QQQ) liquid chromatography (LC)‐MS platform, while untargeted proteomics is commonly performed using Time‐of‐Flight (ToF) or Orbitrap MS platforms (Liebler & Zimmerman, 2013). Targeted and untargeted proteomic approaches have nuances in terms of applications, advantages and limitations in sample processing, data acquisition, and analysis (Granvogl et al., 2007; Klont et al., 2018). Untargeted proteomic studies are used to identify all detectable ions within the sample, whether from EV‐related proteins or contaminant matrix proteins. This approach provides a comprehensive understanding of the sample protein composition and is ideal for applications such as biomarker discovery (Nakayasu et al., 2021). For characterization of MISEV EV purity (Category 1, 2) and matrix contamination (Category 3) markers (Section 5.7), targeted peptide analysis may be more suitable, demonstrating the presence or absence of each analyte above a pre‐specified detection threshold. It can also quantify absolute protein abundance. Multiplexing, for example, as in LC‐MS workflows, can provide high sensitivity for limited sample volumes, such as for samples from clinical trials (Newman et al., 2022). Targeted proteomics may be more suitable to quantify changes in protein abundances, such as in a disease or therapeutic intervention (Pocsfalvi, Stanly, Fiumeet, al., 2016; Rodrigues et al., 2021). Inclusion of stable isotope labelled (SIL) peptide standards enables absolute quantification of the corresponding endogenous analyte when used in combination with ‘light’ peptide calibrators prepared in a matched matrix (Liebler & Zimmerman, 2013).

Instrument settings, including collision energy, gas flow and temperature, and capillary voltage, are platform and analyte‐specific and, as such, should be optimised and then kept constant for the duration of a project. MS instruments are sensitive to contamination by ion‐pairing reagents, buffer salts, and detergents, reducing sensitivity and assay performance. As such, EV peptide samples for targeted LC‐MS analysis should be prepared in low‐salt buffer/MS‐compatible solvent matrix and an appropriate concentration of SIL peptide standard. Positive controls containing proteins of interest and negative controls, such as EVs from alternative species or cell lysates not expressing a protein of interest, should be included in targeted analyses (Abbatiello et al., 2013; Bereman, 2015; Nakayasu et al., 2021). Report the sequences of target peptides and the strategy for peptide selection. The linearity of response and limits of detection and quantification should be defined using synthetic light and heavy‐labelled peptides spiked into an appropriate matrix. Report normalization, for example, by total protein, volume of starting material or particle count from which proteins were digested and injected for MS analysis. When reporting results from either targeted or untargeted proteomic studies, follow the Minimal Information About a Proteomic Experiment (MIAPE) guidelines for harmonization of methodology and rigor/reproducibility (Gandham et al., 2020; Kreimer et al., 2015; Taylor et al., 2007). All sample preparation techniques should be reported with reproducible experimental descriptions for each step. All data software and versions used should be reported to understand how data were processed. Filters, scores and confidence levels for both identifications and quantitation should also be reported, as well as the method used for quantitation if relevant (Martinez‐Bartolome et al., 2013). Data and metadata should be uploaded to a data repository to ensure that data generation and reporting remain rigorous and potentially reproducible for EV experiments.

**Recommendations**:
  - Optimise instrument settings and keep them constant for the duration of a project.
  - In targeted LC‐MS protein analyses, include both positive controls (containing proteins of interest) and negative controls, such as EVs from alternative species or cell lysates not expressing a protein of interest.
  - Spike SIL peptide standards into the EV matrix to assess matrix effects and to demonstrate the concordance of retention times and quantifier‐to‐qualifier ion transition ratios between standards and endogenous analytes.
  - For targeted MRM analyses, monitor at least one quantifier and one (preferably two) qualifier ion transitions.
  - Define linearity of response and limits of detection and quantification using synthetic light and heavy‐labelled peptides spiked into an appropriate matrix.
  - Report sequences of target peptides and the strategy for peptide selection, as well as the isotopic purity and source of synthetic peptides.
  - Sample preparation techniques, including the normalisation approach used, should be reported with detailed experimental descriptions for each step in the workflow.
  - Follow the reporting recommendations of the Minimal Information About a Proteomic Experiment (MIAPE).
  - Upload data and metadata to a data repository.

### 6.4 Microscopy‐based methods

#### 6.4.1 Atomic force microscopy

Atomic Force Microscopy (AFM) provides label‐ and stain‐free imaging of individual EVs and co‐isolated nanoparticles (Bordanaba‐Florit et al., 2021; Obeid et al., 2019; Sharma et al., 2018). AFM imaging requires analytes to be deposited on a solid surface (substrate). Measurements can then be performed after either drying the sample or keeping it submerged in liquid, such as saline or cell culture media. AFM morphometry can be used to obtain EV size distribution and ultrastructural details and to check for the presence and relative amounts of contaminants (Cavallaro, Pevere, et al., 2021; Paolini et al., 2020, 2016; Parisse et al., 2017). In addition, AFM is one of the very few techniques capable of measuring single‐vesicle nanomechanical properties (Gautron et al., 2021; Piontek et al., 2021), which were found to correlate with EV identity and function (Bortot et al., 2021; LeClaire et al., 2021; Romanò et al., 2022; Sorkin et al., 2018; Vorselen et al., 2018; Whitehead et al., 2015; Ye et al., 2021). The unique mechanical fingerprint of EVs can also be used to discriminate them from NVEPs of similar size and shape (Ridolfi et al., 2020).

Minimal reporting requirements for the AFM imaging of EV samples comprise detailed information on the preliminary sample deposition procedure, substrate type and pre‐treatment, immobilization method, sample concentration and deposition times, plus details on any rinsing and/or drying steps. AFM imaging mode, acquisition conditions, and probe information including expected tip curvature radius and spring constant should also be provided. If quantitative morphometry is performed, the heuristics employed to select the measured objects, as well as the procedure to extract morphological descriptors from them, should be described. Reporting the height of the detected particles, for example, greater than or less than the thickness of two lipid bilayers (∼8 nm) may help distinguish between deformed EVs and non‐EVs/collapsed EVs. In addition, EV mechanical studies should describe the assumed contact mechanic model (Calò et al., 2014; Ridolfi et al., 2021; Vorselen et al., 2017), and, ideally, provide enough data for the reader to be able to test alternative models.

**Recommendations**
  - Report preliminary sample deposition procedure, substrate type and pre‐treatment, immobilization method, sample concentration and deposition times, plus details on any rinsing and/or drying steps.
  - Provide AFM imaging mode, acquisition conditions and probe information, including expected tip curvature radius and spring constant.
  - If quantitative morphometry is performed, describe the heuristics used to select the measured objects, as well as the procedure to extract morphological descriptors.
  - EV mechanical studies should describe the assumed contact mechanic model (Calò et al., 2014; Ridolfi et al., 2021; Vorselen et al., 2017), and, ideally, provide enough data for the reader to be able to test alternative models.

#### 6.4.2 Diffraction‐limited fluorescence microscopy

Applications of fluorescence microscopy techniques can range from live cell imaging to single‐molecule localization. These approaches, including Total Internal Reflection Microscopy (TIRF‐M), confocal microscopy and more recently, light‐sheet microscopy, have been used to evaluate cell‐EV interactions such as EV release and uptake (Christianson et al., 2013; Elgamal et al., 2020; Feng et al., 2010; Heusermann et al., 2016; Joshi et al., 2020; Lai et al., 2015; Mittelbrunn et al., 2011), as well as the composition of single EVs (Corso et al., 2019; Han, Kang, et al., 2021). As a general consideration, since TIRF microscopy is limited to imaging the surface at the glass interface and has high signal‐to‐noise ratio that facilitates single molecule detection, it may be the most suitable system for analysing EV content (Han, Kang, et al., 2021). Confocal and light‐sheet microscopes, especially the most recent models, are capable of single‐molecule detection for calibration (Willy et al., 2021) and dynamic studies, but are more suitable for live cell imaging experiments (Elgamal et al., 2020; Mittelbrunn et al., 2011). These methods and potential drawbacks have been extensively reviewed (Chuo et al., 2018; Colombo et al., 2021; Gallego‐Perez et al., 2016; Panagopoulou et al., 2020).

In microscopy experiments, report the type of microscope, magnification, laser power and exposure time because fluorescently labelled samples have a limited number of labelled molecules. Each labelled sample can provide only a finite number of photons before photobleaching, so each experiment must be optimised to maximise the amount of information obtained from a limited ‘photon budget’ (Li et al., 2015). Consequently, the sample is exposed for a short time using minimal excitation to perform live‐cell experiments (Coffman & Wu, 2014; Heddleston et al., 2021) or at higher excitation power and longer camera exposure for single‐molecule detection (Elgamal et al., 2020). While calibration of the system is mandatory for quantitative microscopy experiments (Montero Llopis et al., 2021; Willy et al., 2021), we recommend where possible to extend it to any microscopy approach to obtain unbiased evaluation of sensitivity of the instrument. Calibration to a single fluorescent dye or labelled protein molecules is a well‐established approach that permits one to infer the total number of proteins or RNAs present on or in EVs (de Voogt et al., 2021; Higginbotham et al., 2011) and ensure that even molecules retained in few copies in EV can be detected. The software used to detect EVs should be reported including the specific parameters used to threshold the object intensities. Code developed for these purposes should be deposited and made accessible to the community. Available algorithms (Aguet et al., 2013; Elgamal et al., 2020; Jaqaman et al., 2008) take advantage of the small size of EVs, which are in general diffraction‐limited objects. These assume the same shape as the point spread function (PSF) of the imaging system and can be approximated to a Gaussian function in confocal, TIRF and light‐sheet microscopy.

**Recommendations**
  - Report the type of microscope, magnification, laser power and exposure time.
  - Calibration of the system is mandatory for quantitative microscopy experiments, but is also recommended for any microscopy approach to obtain unbiased evaluation of the sensitivity of the instrument.
  - The software used to detect EVs should be reported, including the specific parameters used to recognise the objects and, if applicable, threshold the object intensities. Any code written for these procedures should be made publicly available.

#### 6.4.3 Dynamic light scattering

DLS, also known as photon correlation spectroscopy (PCS) and quasi‐elastic light scattering (QELS), is a technique capable of determining the hydrodynamic diameter of sufficiently monodisperse particles in dilute aqueous dispersions (Berne & Pecora, 1976; Hackley & Clogston, 2011; “Particle size analysis — Dynamic light scattering (DLS)” 2017; Stetefeld et al., 2016). DLS can be performed as a cuvette analysis or as an inline analysis when connected to a fluidic pump, such as high‐performance liquid chromatography (HPLC). The hydrodynamic diameter is defined as the diameter of a solid sphere that would exhibit the same diffusion coefficient as the measured particle of interest. DLS measures the autocorrelation function of the intensity of laser light scattered by multiple particles in solution. The autocorrelation function carries information about the diffusion coefficient of the particles, which is related to the hydrodynamic diameter via the Stokes‐Einstein theory of Brownian motion.

Various algorithms can be used to derive the diffusion coefficient from the measured autocorrelation function. The most common method, the cumulant analysis, assumes a monodisperse size distribution, which EV samples do not have. Other approaches, such as the CONTIN algorithm, attempt to handle the drawbacks of the cumulant analysis (Provencher, 1982), but for polydisperse size distributions of EV samples (van der Pol, Coumans, Grootemaat et al., 2014), derivation of the diffusion coefficient distribution from the autocorrelation function becomes an ill‐posed mathematical problem. This implies that DLS should not be used to determine quantitative properties, such as the average hydrodynamic diameter, of EV samples, unless DLS is applied to a monodisperse size fraction of EVs, such as an EV sample fractionated by flow field‐flow fractionation using an inline analysis. On the other hand, DLS can be used to qualitatively confirm the presence of submicrometer particles and possible aggregates that may be present in EV samples (Palmieri et al., 2014). In either case, please follow the recommendations on nomenclature and reporting of DLS measurements from the international standard ISO 22412:2017 (“Particle size analysis — Dynamic light scattering (DLS)” 2017).

**Recommendations**
  - DLS should not be used to determine quantitative properties, such as the average hydrodynamic diameter, of EV samples, unless applied to a monodisperse size fraction of EVs.
  - DLS can be used to qualitatively confirm the presence of submicrometer particles and possible aggregates that may be present in EV samples.
  - Follow the recommendations on nomenclature and reporting of DLS measurements from the international standard ISO 22412:2017 (“Particle size analysis — Dynamic light scattering (DLS)”, 2017).

#### 6.4.4 Electron microscopy

Electron microscopy (EM) variants are among the few techniques capable of detecting EVs irrespective of size. The throughput of EM, however, means that larger EVs are statistically underestimated as compared with smaller EVs (van der Pol et al., 2022). While EV characterization by SEM (Cavallaro, Hååg, et al., 2021; Wu et al., 2015), TEM (van der Pol, Coumans, Grootemaat, et al., 2014), and cryo‐EM (de Vrij et al., 2013; Hoog & Lotvall, 2015; Linares et al., 2015) are all high‐resolution methods, they are not necessarily interchangeable or capable of providing images of comparable quality. For example, cryo‐EM clearly shows the lipid bilayer, better maintains EV morphology than the dehydrating conditions used to fix samples for TEM, and may be more quantitative, as all particles in a given volume can be imaged, not just those that adhere to a surface (the grid). TEM should be performed with a protocol adapted to EVs, which includes contrasting and embedding in a mixture of uranyl compounds and methylcellulose to maintain the lipid bilayer morphology (Théry et al., 2006). SEM shows the surface aspect of EVs of any size, but images obtained at the highest magnification required to visualise the smallest EVs may be more difficult to analyse.

There have been limited standardization studies across EM methods to determine minimal reporting requirements. For TEM, three major parameters should be reported: fixation, adsorption, and negative staining methods (Rikkert et al., 2019). Fixation includes: the fixative used, its concentration, and incubation time. Adsorption includes the grid material, mesh size, film type, coating, incubation time, and wash details. Negative staining details should include substance, concentration, and incubation time. Both low‐ and high‐magnification images should be shared, along with selection criteria.

**Recommendations**
  - TEM should be performed with a protocol adapted to EVs, which includes contrasting and embedding in a mixture of uranyl compounds and methylcellulose to maintain the bilayer morphology.
  - Three major criteria should be reported for any electron microscopy technique used: fixation, adsorption, and negative staining methods.
  - High‐ and low‐magnification images should be supplied for both high‐resolution EV images and an assessment of the broader quality of the sample.

#### 6.4.5 Nanoparticle tracking analysis

NTA, also known as single particle tracking, is a widely utilised optical technique in the EV field to estimate particle size and concentration. The use of NTA to determine effective refractive index and epitope existence has also been demonstrated (Gardiner et al., 2014; van der Pol, Coumans, Sturk, et al., 2014). NTA derives hydrodynamic diameter by measuring a particle's diffusion coefficient, usually implementing an algorithm that reduces variation in diameter distribution. It should be noted that the FTLA algorithm used on some platforms was developed to better represent monodisperse mixtures, of which EVs are not, and can result in artefactual multi‐modal distribution (van der Pol, Coumans, Grootemaat, et al., 2014; Walker, 2012). Currently, there is no method of determining or reporting a set LOD for NTA. Several standardization studies have been conducted comparing results between users and instruments (Bachurski et al., 2019; Hole et al., 2013; Vestad et al., 2017). The use of NTA to measure the diameter distributions and concentration of complex biofluids should be interpreted with caution due to counting of co‐isolates such as lipoproteins and large protein complexes, and EVs larger than a few hundred nanometers in diameter are difficult to quantify. Detection of particles with NTA can be done using light scattering, relying on refractive index and diameter, or fluorescence. Fluorescence NTA depends on removal of unbound label, photobleaching resistance of the dye, and the presence of detectable levels of dye per particle.

For NTA reporting, include instrument model, camera type, camera settings, laser wavelength, laser power, software version, analysis settings, and particles per frame. As outlined in Section 5.2, NTA diameter distributions are preferred over a single diameter statistic, since NTA statistics are easily skewed by the LOD. If known, the algorithm used to produce diameter distributions should be reported due to potential for differing results depending on the algorithm used (Kestens et al., 2017; Walker, 2012). A buffer‐only control is recommended in the case of light scatter or fluorescence detection modes. For fluorescent NTA, report the number of total particles in light scatter mode along with the number of labelled particles in fluorescence mode, along with label removal method and a buffer/reagent control to assess labelling artefacts. Report sample injection fluidics and settings if used.

**Recommendations**
  - Instrument model, camera type, camera settings, laser wavelength, laser power, software version, analysis settings and particles per frame should be reported.
  - Report NTA diameter distributions rather than a single diameter statistic.
  - If known, report the algorithm used to produce diameter distributions.
  - A buffer‐only control is recommended for both light scatter and fluorescence detection modes.
  - When using fluorescent NTA, it is recommended to report the number of total particles in light scatter mode, the number of labelled particles in fluorescence mode, along with label removal method, Use a buffer‐only/reagent control to assess labelling artefacts.
  - Report injection fluidics and settings if used.

#### 6.4.6 Single‐particle interferometric reflectance imaging sensing

Combined interferometric imaging/fluorescence imaging (Bachurski et al., 2019; Crescitelli et al., 2021; Daaboul et al., 2016; Dogrammatzis et al., 2021) involves particle capture by affinity agents (e.g., antibodies, peptides, aptamers) onto a multiplexed array of micron‐sized spots. In interference reflectance imaging sensor (IRIS) mode, interference patterns from scattered light are used to derive the size and number of captured particles (Young et al., 2018). Converting interference to nominal size depends on refractive index (RI), which can vary across EV populations (de Rond, Coumans, et al., 2018). Current SP‐IRIS platforms assume a constant RI (∼1.45), which may result in variation across orthogonal measurements and may undersize EVs with lower RI. It is thus recommended that software version and estimated refractive index parameter be reported.

In fluorescence mode, captured particles labelled with fluorescent probes are detected in one or more color channels. Some aspects of this mode require careful consideration of calibrations and control experiments to obtain rigorous results. For particles smaller than the diffraction limit, for example, <∼250 nm in diameter for visible light, validate the detected events to confirm that single particles were detected, for example, with a dilution series to ensure that fluorescence intensity per particle does not scale with solution concentration. To confirm that fluorescence is associated specifically with EVs, vesicle‐disrupting surfactant treatments can be used; however, consider that surfactants can also disrupt lipoprotein particles (Botha et al., 2022). For fluorophore detection, reporting recommendations are indicated below.

**Recommendations**
  - Report details of affinity reagent(s) printed onto the chip.
  - Report software version and estimated refractive index parameter.
  - For particles smaller than the diffraction limit, detection of single events should be validated.
  - To confirm that fluorescence is associated specifically with EVs, surfactants can be used to disrupt vesicles (although they may also disrupt certain NVEPs).
  - For fluorophore detection, report affinity reagent (e.g., antibody clone), conjugated fluorophore type, incubation concentration, light‐source wavelength, bandpass filter cut‐offs, analysis software version and fluorescence cut‐offs along with the method of choosing these cut‐offs. Negative controls such as nonspecific IgG capture spots or chips incubated with EV‐depleted materials are recommended for choosing these cut‐offs.

#### 6.4.7 Super‐resolution microscopy

To resolve fluorescence emitters events that are closer together than the diffraction limit of light, fluorescent super‐resolution microscopy methods modulate the light to ensure that neighbouring molecules do not emit simultaneously. A resolution 10‐fold below the diffraction limit can be achieved using two main approaches: (1) stimulated emission depletion (STED) (Hell & Wichmann, 1994; Klar & Hell, 1999), which spatially regulates activation of an ensemble of fluorophores using a synchronised two‐laser system with a phase plate; and (2) single molecule localization microscopy (SMLM) techniques, such as (d)STORM (Rust et al., 2006; Wombacher et al., 2010) and (f)PALM (Betzig et al., 2006; Hess et al., 2006), which temporally regulate stochastic activation of single fluorophores. The nanometer scale resolution of STED and SMLM is well suited for detecting and characterising individual EVs and their components, including EV membranes (Nizamudeen et al., 2018; Sharma et al., 2020; Zong et al., 2018), proteins (Avalos‐Padilla et al., 2021; Chen et al., 2016; Lennon et al. 2019; Maire et al. 2021; Mondal et al., 2019; Sanada et al., 2017; Wang et al., 2018; Zong et al., 2018), DNA fragments (Maire et al. 2021) and miRNAs (Chen et al., 2018; Oleksiuk et al., 2015). Using quantitative analysis, these methods have been further used to define EV size (Mondal et al., 2019; Zong et al., 2018; Nizamudeen et al. 2018; Lennon et al. 2019; Sharma et al., 2020) and to quantify protein content (Lennon et al., 2019) and number of localizations of miRNA (Oleksiuk et al., 2015) and DNA fragments in EVs (Maire et al., 2021). Additionally, STED and SMLM have been used to image cellular uptake (Chen et al., 2016; Chen et al., 2018; Polanco et al., 2018; Pfeiler et al., 2019; Toda et al., 2020) and release of EVs (Ambrose et al., 2020) or EV clusters (Valcz et al., 2019).

Super‐resolution microscopy methods comprise tailored approaches for sample preparation, sample imaging, and data analysis. To prepare samples for SMLM and STED imaging, EV membranes or cargo molecules are labelled with reagents that contain appropriate photo‐controllable fluorophores. Four typical strategies for labelling EVs are affinity labelling, genetic labelling, covalent labelling, and uptake of lipophilic molecules/lipid analogues. Reported details of labelling should include type of labelling, appropriate reagent controls and/or references, reagent concentration, incubation times/buffers, and method for removal of excess fluorescent reagents). If applicable (e.g., for isolated EVs), reporting should include coverslip modifications/coatings, the protocol for incubation of EVs on coverslips, fixation protocol, and controls for affinity separation (e.g., isotype or non‐fouling surface). Reported imaging parameters should include the major microscope components: laser lines, camera, filters, objectives and other relevant optical path components. Descriptions of protocols should include detailed imaging parameters such as laser powers, relevant microscope configuration and imaging conditions (including buffer for SMLM). Reports on multicolour imaging should detail the alignment between channels and any applied correction for chromatic aberration (Churchman & Spudich, 2012; Hebisch et al., 2017).

In STED, the resulting images consist of intensity maps, and analysis typically relies on approaches established in confocal microscopy (Gould et al., 2012); relevant processing/analysis parameters should be reported. SMLM images are reconstructed from the determined coordinates (i.e., localizations) of single molecules, and EV analysis typically employs segmentation and/or clustering algorithms (Khater et al., 2020). To quantify detected molecular densities and molecular organization with SMLM, it is important to define the photophysical properties of fluorescent reporters (e.g., average number of localizations per molecule, maximal dark time) (Khater et al., 2020). Thus, SMLM reporting should include details on image processing parameters, photophysical characterization of relevant fluorescent reporters, and data analysis parameters/algorithms. Newly developed analysis methods should be evaluated (e.g., using simulations or another validated approach), and custom written codes should be made publicly available.

**Recommendations**
  - Reporting details of the EV labelling protocol.
  - Where applicable, report coverslip modifications/coatings, the protocol for incubation of EVs on coverslips, fixation protocol and controls for affinity separation.
  - Report the major microscope components and imaging protocol parameters such as laser power, relevant microscope configuration and imaging conditions.
  - Reports on multicolor imaging should detail the alignment between channels and any applied correction for chromatic aberration.
  - SMLM reporting should also include details of image processing parameters, photophysical characterization of relevant fluorescent reporters and data analysis parameters/algorithms.
  - Newly developed analysis methods should be evaluated, and custom written codes should be made publicly available.

### 6.5 Nucleic acid characterization

Nucleic acids (NAs) are among the most commonly assayed EV constituents because of perceived biomarker potential and functional roles. RNA has been studied much more frequently than DNA, although there are more recent reports on EV DNA in intercellular communication (Clancy et al., 2022; Sansone et al., 2017) and as disease biomarkers (Cambier et al., 2021; García‐Silva et al., 2019; Möhrmann et al., 2018; Qu et al., 2019; Vagner et al., 2018) including in microbial infections (Bitto et al., 2017; Kameli et al., 2021). Some early EV studies reported DNA inside the EV lumen (Cai et al., 2013; Kahlert et al., 2014; Lee et al., 2014; Thakur et al., 2014), whereas some recent studies have suggested mostly EV surface‐association of DNA (Bitto et al., 2017; Lázaro‐Ibáñez et al., 2019; Liu et al., 2022; Maire et al., 2021; Saari et al., 2020). These seemingly contradictory findings might be due to the lack of standardised methods for protecting EV surface DNA from digestion during EV separation and characterization (Lázaro‐Ibáñez et al., 2019). Whether the major type of EV DNA is ssDNA or dsDNA is also still debated (Balaj et al., 2011; Lázaro‐Ibáñez et al., 2019; Liu et al., 2022; Thakur et al., 2014).

Challenges for RNA studies including input quantities, normalization and sensitivity are also relevant for EV DNA research. Most characterization of EV RNAs involves one or more of: detection, identification, quantification, localization (inside or outside the EV) and enrichment (packaging). Low‐input RNA sequencing (RNA‐Seq) and quantitative PCR (qPCR) are commonly used to identify specific sequences in EV preparations. ISEV has previously provided guidance on aspects of EV RNA studies ranging from sample collection to bioinformatic analysis (Hill et al., 2013; Soekmadji et al., 2018; Witwer et al., 2013), as has the US NIH Extracellular RNA Communication Consortium (ERCC) (Ainsztein et al., 2015; Das et al., 2019).

Regardless of RNA characterization method, biases may be introduced by RNA purification and pre‐assay preparations. Some RNA purification methods isolate mostly longer RNAs (>200 nt), while others are biased by design to concentrate short RNAs. For RNA‐Seq, library preparation methods may select for RNAs or inserts within a particular size range. Reverse transcription protocols may also select for specific RNAs such as polyA‐tailed transcripts. Tiled probe‐based imaging of longer RNAs becomes less sensitive for shorter/degraded transcripts. Adapter ligation‐based small RNA library preparation methods optimised for miRNAs will also enrich other RNAs containing 5′‐phosphates and 3′‐OH, while RNAs bearing different end‐chemistries will be underrepresented. Highly structured RNAs such as full‐length tRNAs are not efficiently reverse‐transcribed unless using thermostable enzymes. Accurate interpretation and reporting of results thus depend on understanding and reporting techniques with enough detail to assess biases.

Due to its ability to detect and measure small amounts of nucleic acids, reverse transcription real‐time quantitative PCR (qPCR) is widely used in the EV field. We recommend that qPCR experiments follow the Minimum Information for Publication of Quantitative Real‐Time PCR Experiments (MIQE) guidelines (Bustin et al., 2009) where possible, and the ISEV EV RNA checklist in the 2017 ISEV position paper (Mateescu et al., 2017). When sharing qPCR results, raw cycle of quantitation (Cq) values should be reported in addition to normalised or processed data for readers to assess abundance of the target RNA and reliability of the assay. Although Cq values depend on many variables and may not by themselves be informative, they tend to correlate with abundance, especially in liquid samples, and where the input sample volume can be reported. Possibly, not all MIQE principles broadly apply to extracellular samples. For example, when samples contain only minute amounts of carrier‐specific RNA, having identical input RNA levels in every sample might not always be possible. Some prefer to normalise by sample input volume, given the liquid nature of extracellular samples. Normalization strategy can greatly impact interpretation of the results and should be reported. Digital PCR, including droplet digital PCR (ddPCR), provides absolute quantification and has been shown to improve reproducibility and accuracy of EV RNA detection compared with conventional qPCR (Wang et al., 2019). Absolute quantification may also circumvent issues with normalization.

**Recommendations**
  - For qPCR‐based analysis, report sequences of reverse transcription adapters or primers as well as primers and probes (where relevant) for amplification steps; experimental design with biological and technical replicates; exact cycling conditions; and data inclusion and exclusion criteria.
  - For RNA‐Seq, report all details of nucleic acid fragmentation, reverse transcription, adapters and adapter attachment (ligation or ligation‐free), amplification and multiplexing, as well as clean‐up or size selection.
  - For sequencing data analysis, report pre‐processing, read mapping, overlapping annotations and database quality, quantification and normalization/differential expression analysis.

### 6.6 Protein‐ and non‐protein labelling of EVs

Most EV labelling reagents include fluorescence moieties, but other modes of detection are available and should share similar controls. Due the small size and thus limited cargo capacity of EVs, the detection of protein and non‐protein markers is difficult and can easily be confounded by unbound reagents from the labelling process or co‐isolates from the separation method. The degree to which unbound label requires removal increases with the sensitivity of the techniques. For techniques that can detect <10 molecules of a reagent, for example, super‐resolution microscopy, SP‐IRIS and single EV flow cytometry, the presence of unbound dye may easily lead to false positive events.

Lipid dyes are routinely used to bind to/insert into the EV membrane (de Rond, van der Pol, et al., 2018; Feng et al., 2010; Lundy et al., 2015; Sandau et al., 2020; Stoner et al., 2016). Lipid‐specificity does not guarantee EV‐specificity, since NVEPs such as lipoproteins may be co‐isolated and stained, and some reagents may also label proteins. Supplementation with an EV protein marker is therefore recommended. Lipid labels may also self‐aggregate (de Rond, van der Pol, et al., 2018; Pužar Dominkuš et al., 2018) and vary in affinity for EVs with different membrane composition.

Protein‐reactive dyes that label the EV surface (Lim et al., 2021; Roberts‐Dalton et al., 2017; Tian et al., 2010) may also label free protein and protein‐containing NVEPs. If the EV separation method does not completely remove non‐EV components, this possibility should be recognised and/or assessed. As above, a lipid marker might be used to complement protein labelling. When protein artefacts are a possibility, a low‐concentration detergent can be used to assess the lability of the EV membrane and reduction of associated signal (Gyorgy et al., 2011).

For antibodies, manufacturer‐matched isotype controls, used at the same concentration as the specific antibody, are one way to support specificity. Negative EV controls, for example, from cells that do not express the antibody epitope, are also useful controls.

In assays where purification is required after staining, procedural controls should be used to demonstrate before/after consistency of the EV population, that the purification procedure did not introduce artefacts, and that the dye was removed. For example:

1. Analyse a buffer with reagent control before and after label depletion method (e.g., SEC) to assess free/aggregated label removal.
2. Analyse unstained EVs before and after the label depletion method to demonstrate that it does not change or selectively enrich the EV population.
3. Analyse the reagent‐stained sample after label removal and compare with unstained results (above) to assess possible dye‐induced changes. Staining may noticeably increase the diameter or density of small EVs in particular.

**Recommendations**
  - Use a buffer/label‐only control to identify false‐positive artefacts arising from unbound label. However, label‐only artefacts are not the only potential labelling artefacts.
  - For antibodies, manufacturer‐matched isotype controls may be used at the same concentration as the specific antibody to evaluate binding specificity. Negative EV controls (lacking the antibody epitope) may also be used.
  - Be aware that EV protein labelling methods may also label free proteins and protein‐containing NVEPs, and that lipid dyes may label lipid‐containing NVEPs. Ensure that the EV separation method is appropriate for the downstream analysis. If the EV separation method does not completely remove non‐EV components, this possibility should be recognised and/or assessed.
  - To identify the contribution of non‐EV labelling artefacts, consider using protein and lipid labelling concurrently.
  - In assays where purification is required after staining, procedural controls should be used to demonstrate before/after consistency of the EV population, that the purification procedure did not introduce artefacts, and that excess dye was removed.

### 6.7 Raman spectroscopy

Raman spectroscopy (RS) is a label‐free analytical optical technique capable of qualitatively and quantitatively resolving the chemical composition of a small volume of a sample based on inelastically scattered photons originating from the sample upon irradiation with a narrow‐linewidth laser (Smith & Dent, 2005). A Raman spectrum is essentially a chemical fingerprint of the interrogated small volume of the sample within the focus of the laser beam. RS enables chemical specific, non‐destructive probing, minimal to no sample pre‐processing, and it is relatively inert to aqueous content of the measured sample (Smith & Dent, 2005). A strategy to overcome the weak signals of RS is the use of surface‐enhanced Raman scattering (SERS), which is a nano plasmonic‐assisted amplification derivative of RS (Langer et al., 2020; Jones et al., 2019). This method uses metal nanostructures to boost Raman scattering by many orders of magnitude. Both spontaneous and surface‐enhanced Raman methods have demonstrated utility for basic research and translational EV analyses (Carlomagno et al., 2021; Enciso‐Martinez et al., 2020; Gualerzi et al., 2017; Gualerzi et al., 2019; Kwizera et al., 2018; Lee et al., 2018; Ma et al., 2018; Park et al., 2017; Rojalin et al., 2020; Smith et al., 2015).

Inter‐ and intra‐device variability in Raman spectra can arise for several reasons, including laser variations and non‐uniform response of each of the optical elements, including the detector, to different light energies (known as spectral response). Raman systems should therefore be carefully calibrated (Raj et al., 2020). Modern commercial Raman systems have automatic calibration routines, but older and lab‐built systems do not, thus adding to the issue of reproducibility. Several aspects of the measurement should be reported, including laser wavelength and power, calibration routines, make/model of major optical components, numerical aperture and magnification of the objective (if applicable), probe type and specifications (typically for non‐microscope setups and measurements), and physical size of the laser spot. Spectra acquisition parameters should also be mentioned, for example, total number of spectra collected on each sample or sampled spot, signal collection time per one spectrum (also called as integration or acquisition time), and for scanning, the dimensions of the scanned area/volume (e.g., 100 × 100 area, step size of 400 nm, total scanned area 40 μm × 40 μm). Lastly, it is recommended to report all pertinent parameters of sample preparation. As EV samples are typically suspended in aqueous solutions with different concentrations of dissolved compounds, and thus osmotic pressures, there is a need to consider and report the EV formulation and whether the EVs were measured in suspension or dry. For example, EVs can be measured in suspension using SERS nanoprobes or dried onto a quartz glass slide for RS spectra acquisition (Cameron et al., 2018). It is unclear if there is an advantage to wet versus dry measurements (Butler et al., 2016), so both approaches are considered feasible provided that the EV sample preparation steps are detailed.

Along with instrument and sample considerations, data analysis and statistical procedures can impact the endpoints and conclusions of RS studies. All data analysis software and versions should be reported. If custom‐made program suites and algorithms are employed, it is recommended that the code be deposited in an online data repository for transparency and re‐usability. After acquisition (and before downstream analyses), spectra that are meant to be compared with each other should be postprocessed using identical data manipulation parameters. For example, if baseline correction and/or background subtraction is implemented, all related parameters should be kept constant for all spectra. All downstream spectral analyses and further statistical testing (e.g., multivariate analysis, machine learning, statistical hypothesis testing) should be reported in full and with data openly available.

**Recommendations**
  - Report all instrument and measurement parameters.
  - Report sample preparation/application parameters including buffer composition and wet/dry measurement.
  - Report data analysis software and versions. Deposit any code for custom‐made program suites and algorithms in an online data repository for transparency and re‐usability.
  - Report downstream spectral analyses and further statistical testing.

### 6.8 Resistive pulse sensing

Resistive pulse sensing (RPS) is a non‐optical technique utilising the Coulter principle to determine the concentration and diameter of particles (Hogg & Coulter, 1967), along with zeta potential on some platforms. Current implementations of RPS include pre‐calibrated fixed pores in a microfluidic cartridge format and uncalibrated stretchable pores, both with detection limits down to ∼50 nm in diameter and the capability to measure particles up to several microns. The use of RPS to measure the diameter distributions and concentration of EVs in complex biofluids should be interpreted with caution, since co‐isolates, such as lipoproteins and large protein complexes, are also counted and cannot be differentiated from EVs. RPS measurements do, however, have very high concordance with TEM data (van der Pol, Coumans, Grootemaat, et al., 2014).

When reporting RPS data it is recommended that instrument model, pore size, calibration bead diameter and source, and software version be reported. For stretchable pores, the applied voltage, applied stretch, and procedure to optimise settings should be shared (Coumans et al., 2014). For microfluidic RPS, appropriate dilution buffer to lower the surface tension of water should be considered and reported (Cimorelli et al., 2021). As outlined in Section 5.2, it is preferable to report RPS diameter distributions rather than a single diameter statistic for EV data, due to RPS statistics being easily skewed by the LOD. The inclusion of buffer‐only controls to identify background, along with detergent‐lysed samples run at the same concentration to determine label events is also recommended (Osteikoetxea et al., 2015). Due to RPS techniques being easily clogged by larger particles, pre‐analytical steps such as centrifugation or filtration may be used to remove larger particles. Since these approaches may alter the EV population being analysed and affect comparison with orthogonal methods, any preanalytical procedures should be clearly stated.

**Recommendations**
  - Report any preanalytical procedures applied prior to RPS.
  - For microfluidic RPS, appropriate dilution buffer should be considered and reported.
  - Include buffer‐only controls and detergent‐lysed samples run at the same concentration as the untreated sample.
  - Report all instrument and software details.
  - Report RPS diameter distributions rather than a single diameter statistic.

### 6.9 Western blotting

Western blotting is a commonly used method to detect proteins in EV‐containing preparations. Proteins are first separated by gel electrophoresis, then transferred to a membrane and probed with affinity reagents, usually antibodies. Input is often normalised by some aspect of the EV preparation (total protein, particle count) or some aspect of the EV source (biofluid volume, cultured cell number): the former allows comparison of amounts of EV cargo between similar groups of EVs, while the latter might also assess overall differences in EV production/uptake balance in the source system. For cell culture EVs, cell lysates, either in specified protein amount or in cell‐equivalent amounts, should be loaded onto the same gel to assess enrichment/depletion in EVs versus producing cells. This comparison, however, can be easily performed only for analysis of EVs from cell culture‐conditioned medium, since for other sources of EVs (e.g., biological samples), the source cells cannot be easily identified or recovered.

Where possible, known antigen‐positive and ‐negative control samples should be included beside the experimental samples. Controls for assessing the purity of the sample preparation should also be included if claiming the protein is present on or in EVs; see Section 5.7. Antibody information (specificity, clone, source, labelling concentration, incubation time), sample denaturing conditions, presence, and nature of reducing agent, transfer methodology, membrane type, buffers, and imaging equipment and parameters should all be reported. For transparency, it is recommended that uncropped images of Western blots (including controls and a molecular weight ladder) be provided at a minimum as supplementary information.

**Recommendations**
  - Provide details of protein enrichment and quantification.
  - Where possible, include antigen‐positive and ‐negative controls
  - If claiming EV‐association of a protein, include measures of the purity of the EV preparation.
  - Report all details of input normalization, gel electrophoresis, transfer methodology, probing and imaging/analysis. These include but are not limited to antibody information, sample denaturing and reducing conditions, transfer methodology, membrane type, buffers, and imaging equipment and parameters.
  - Provide uncropped images of all Western blots (e.g., as supplementary information if published in a journal).
    Consensus: 70.6% (705) of MISEV2023 survey respondents agreed “completely,” and 27.5% (274) agreed “mostly” with Section 6: Technique‐specific reporting considerations for EV characterization. 0.4% (4) “mostly” disagreed, and 1.5% (15) stated that they had no opinion and/or expertise. No respondents disagreed “completely.”

## 7 EV RELEASE AND UPTAKE

### 7.1 Approaches to modulate EV release

EV release can be visualised by a range of methods, including those employing fluorescent tags and dyes (Sections 6.2, 6.6), which permit real‐time imaging [reviewed in (Verweij et al., 2021)]. MISEV2018 discussed inhibition of EV release with a range of genetic manipulations and drugs, for example, *RAB27A/B* knockdown (Ostrowski et al., 2010), neutral sphingomyelinase inhibition (Trajkovic et al., 2008) and ARRDC1 inhibition (Mackenzie et al., 2016; Wang & Lu, 2017). More recent genetic and pharmacological manipulations are reviewed elsewhere (Catalano & O'Driscoll, 2020; Dixson et al., 2023; Zhang, Lu, et al., 2020). Some cellular manipulations can also stimulate EV release (Taher et al., 2019). While these treatments are often claimed to be specific for EVs of particular biogenesis pathways, they may affect EV formation and membrane trafficking more generally. It is thus difficult to exclude an impact on other EVs and/or non‐EV cellular processes (Izumi, 2021; Mathieu et al., 2019; Puca et al., 2013; Xiang et al., 2021). MISEV2018 highlighted the importance of identifying biogenesis machinery that is confined to particular EV subtypes, and this remains a priority, with very few specific additional regulators identified. Using complementary methods to attenuate and/or enhance the production of specific EV subtypes can add strength to data suggesting their association with specific functions. The resulting EVs and control preparations should be analysed using the physical and molecular methods described in Sections 5 and 6, with particular attention to normalization methods (e.g., based on the number/protein mass of secreting cells, or EV number, etc.), identification of unchanged as well as altered markers, where possible, for specificity, and the use of multiple cell types to test whether the mechanism is generic or cell type‐specific.

**Recommendations**
  - For genetic and pharmacological manipulations used to inhibit or stimulate EV secretion, report potential effects on other secretory or cell biological processes. For example, confirm that there is no change in cell viability, proliferation and secretion of non‐EV‐associated factors.
  - Where possible, assess whether inhibiting a specific EV production pathway leads to a change in other EV release mechanisms by assessing EV‐specific cargoes or activities.
  - Identification of unchanged markers and the use of appropriate normalization methods are important for rigorous comparative analysis of EV preparations.

### 7.2 EV interaction with cells

EVs can interact with target cells at different levels: binding, internalization, and fusion/content delivery. EVs contact the surface of cells, which might be referred to as ‘EV binding’. In contrast, ‘EV uptake’ encompasses several outcomes. It can mean fusion of the EV with the cell membrane and release of contents into the cytoplasm. It can also mean internalization into the endocytic and/or other intracellular compartments of the cell, with or without EV‐cellular membrane fusion. EV‐mediated effects on the recipient cell might thus be occasioned by EV binding to receptors at the cell surface or internally and/or by release of contents into the cell at the surface or internally. The relative importance of these different interactions remains unclear, even though most reports of EV function have assumed content delivery. However, EV uptake may occur only at a low rate (Bonsergent et al., 2021; Somiya & Kuroda, 2021a, 2021b) in some target cells, necessitating a high ratio of EVs to target cells to visualise this process (Jurgielewicz et al., 2020; Ragni et al., 2019).

How can these different modes of action be interrogated? Some fluorescence microscopy methods can identify subcellular fluorescent events associated with cells, while flow cytometry mostly detects EV ‘capture’ without discriminating between binding and uptake. For all methods, the long‐lived nature of EV labelling substances may not accurately reflect the presence of EVs in target cells, lipophilic dyes might change EV properties (Section 6.6), and detection of downstream receptor‐mediated cell signalling induced by EVs does not discriminate between different modes of action. While covalently bound dyes cannot be exchanged between EVs and cell membranes without fusion, lipophilic dyes can be exchanged without actual EV transfer, resulting in false positive signals (Simonsen, 2019). New approaches for assaying cargo delivery (including endosomal escape) have been developed since MISEV2018, for example, anti‐GFP fluobodies (Joshi et al., 2020), proteolytic cargo cleavage (Perrin et al., 2021), split‐luciferase reporters (Somiya & Kuroda, 2021a), CRISPR‐Cas9 reporters (de Jong et al., 2020), Cre reporters (Borghesan et al., 2019), trans‐activator delivery (Somiya & Kuroda, 2021b) and knockout of a cargo gene in recipient cells (Taha et al., 2020). By labelling specific EV subtypes, blocking their biogenesis and assaying cargo delivery, it may be possible to determine how EV‐target cell interaction mechanisms vary between different EV subtypes and EV donor‐acceptor combinations. Going forward, inhibition of specific EV ligand‐receptor interactions may establish discrete phenotypic effects: for example, by genetic approaches or addition of blocking antibodies or inhibitory compounds. Blockade of specific intracellular trafficking pathways will suggest which are critical for EV function.

Recommendations

- Assess the suitability of the labelling/reporting system in terms of the impact on normal cellular processes, the stability of the EV‐cell association, and longevity within an intracellular environment.
- Report EV:recipient cell ratios and the physiological relevance of the delivered dose.
- Report incubation conditions, exposure time, cell densities, and configuration, for example, 2D/3D.
- Evaluate binding, uptake and content transfer to identify critical mechanistic elements driving the cellular response(s).
  Consensus: 69.6% (695) of MISEV2023 survey respondents agreed “completely,” and 24.3% (243) agreed “mostly” with Section 7: EV release and uptake. 0.2% (2) “mostly” disagreed, and 5.8% (58) stated that they had no opinion and/or expertise. No respondents disagreed “completely.”

## 8 FUNCTIONAL STUDIES

MISEV2018 recommendations on functional studies of EVs continue to hold for MISEV2023. Because of the great diversity of functional studies in vivo and in vitro, we provide only general recommendations. First, physiologically informed dose‐response and time‐course studies are encouraged. Second, carefully selected EV negative controls are needed to assess the contribution of ‘background’ EV activity (such as EVs present in culture medium components) and/or non‐specific activity of EVs other than those of interest. For cell culture‐derived EVs, this might mean unconditioned medium that has been processed in the same way as conditioned medium (i.e., to separate any EVs that may be present in culture medium components). For EVs from a specific cell type, EVs from another cell type might serve as an appropriate control. For engineered EVs, consider EVs from unmanipulated cells or cells engineered with an irrelevant component (cell engineering) or EVs that have not been modified (post‐production engineering). For patient disease studies, use EVs sourced from healthy, matched or untreated donors. Third, controls consisting of non‐EV‐containing, EV‐depleted or enzymatically treated EV separation fractions can help to identify if a function is specific to EVs or associated with co‐isolating materials. Possibly complicating this analysis, evidence has emerged since MISEV2018 for a functional role of certain loosely tethered coronal elements, as discussed in Sections 3.4 and 4.7, and EV co‐isolates may indeed contribute along with EVs, additively or synergistically, to effects. Finally, the influence of EV separation/concentration, storage and formulation factors on EV activity should be studied, with the goal of maximising activity. Importantly, it is not expected that all conceivable controls will be studied simultaneously in any given system. Instead, potency assays (Gimona et al., 2021; Nguyen et al., 2020) can be used (or developed) to identify the most informative controls for pre‐clinical and clinical studies.

**Recommendations**
  - Perform dose‐response and time‐course studies to assess specificity, kinetics and saturability.
  - Report and justify the method(s) used to normalise input.
  - Evaluate negative EV controls where possible to rule out effects of ‘background’ EVs (e.g., from culture medium) and to evaluate the specific effects of EVs from a certain source or of specific EV elements.
  - Evaluate appropriate non‐EV (e.g., NVEP, soluble protein) negative controls to understand the EV‐association of specific activities.
  - Assess the effects of pre‐analysis factors, especially storage and formulation, on EV activity.
    Consensus: 71.1% (710) of MISEV2023 survey respondents agreed “completely,” and 25.1% (250) agreed “mostly” with Section 8: Functional studies. 0.3% (3) “mostly” disagreed, and 0.1% (1) “completely” disagreed. 3.4% (34) stated that they had no opinion and/or expertise.

## 9 EV ANALYSIS IN VIVO

In vivo EV studies can provide mechanistic insights into EV release, biodistribution, pharmacokinetics and function (Verweij et al., 2021) and may be performed in a wide variety of species, including but not limited to model organisms that recapitulate aspects of human health and disease. In genetically tractable organisms, progress may be facilitated by EV tags and cellular reporter systems (Section 6.2). The relative ease of genetic manipulation of invertebrate and vertebrate model organisms allows hypothesis testing and specific EV labelling approaches (Beckett et al., 2013; Budnik et al., 2016; Fan et al., 2020; Gross et al., 2012; Verweij et al., 2019), including for EV subtype‐specific mechanisms (Beer et al., 2018; Fan et al., 2020). Table 4 presents non‐exhaustive examples of in vivo models for EV studies, each of which has specific strengths and limitations. For example, enlarged endosomal compartments in secondary cells of the fruit fly *Drosophila melanogaster* allow visualization of intraluminal vesicle biogenesis (Corrigan et al., 2014; Fan et al., 2020), while larval motor neurons express multiple EV cargoes with known physiological roles, such that EV regulatory mechanisms can be tested through functional assays (Koles et al., 2012; Korkut et al., 2013; Walsh et al., 2021). The transparent nematode *Caenorhabditis elegans* has also provided insights into the cellular, developmental, and behavioral roles of EVs in addition to EV biogenesis (Beer & Wehman, 2017; Wang et al., 2014; Wehman et al., 2011). EV separation and concentration are challenging for small invertebrates but have been reported from nematode worms (Nikonorova et al., 2022; Russell et al., 2020) and fruit flies, (Thomas et al., 2018; Tsai et al., 2019). By virtue of its transparency, the zebrafish embryo can be used for real‐time biodistribution and uptake studies (Hyenne et al., 2019; Verweij et al., 2019). In contrast, larger mammalian models may be needed to recapitulate some aspects of human physiology and disease processes. A key strength of in vivo models is the opportunity to assess the release of physiological levels of EVs and their interaction with target cells.

**TABLE 4:** Studying EV biology in vivo.

In vivo models	EV‐releasing cells or other EV source	Other specific strengths	Genetic tractability	Genetic similarity to humans
Budding yeast *Saccharomyces cerevisiae*	Unicellular yeast (Oliveira et al., 2010; Zhao et al., 2019)	Whole organism analysis in vivo	++++	+
Green algae *Chlamydomonas reinhardtii*	Flagellated unicellular algae (Wood et al., 2013)	Cilia biology	++++	+
Flowering plant *Arabadopsis thaliana*	Leaf cells (Baldrich et al., 2019; He et al., 2021)	Plant immunity	++++	+
Nematode *Caenorhabditis elegans*	Embryonic cells (Beer et al., 2018; Wehman et al., 2011)	EV release mechanisms; whole organism analysis in vivo	++++	++
	Larval epithelial cells (Liégeois et al., 2006; Hyenne et al., 2015)	EV release mechanisms; whole organism analysis in vivo		
	Ciliated sensory neurons (Clupper et al., 2022; Nikonorova et al., 2022; Razzauti & Laurent, 2021; Wang et al., 2015)	Cilia biology; whole organism analysis in vivo; reproductive functions		
Fly *Drosophila* *melanogaster*	Larval wing imaginal disc (Beckett et al., 2013; Gradilla et al., 2014; Gross et al., 2012; Matusek et al., 2014)	Wnt/Hedgehog morphogen signalling	++++	++
	Larval motor neuron axon terminals (Koles et al., 2012; Korkut et al., 2013; Walsh et al., 2021)	Synaptic function		
	Larval haemocytes (Tassetto et al., 2017)	Adaptive immune system		
	Adult male secondary cells (Corrigan et al., 2014; Fan et al., 2020; Marie et al., 2023)	Large MVBs: exosome subtype biogenesis; reproductive functions		
	Adult muscle cells (Jewett et al., 2021)	Neurodegeneration		
Zebrafish *Dario rerio*	Embryonic yolk syncytial layer (Verweij et al., 2019)	Transparent embryos: EV imaging in bloodstream; target cell biodistribution; metabolic functions	+++	+++
	Adult osteoblasts (Kobayashi‐Sun et al., 2020)	Fracture healing		
	Larval and adult cardiomyocytes (Scott et al., 2021)	Cardiovascular disease		
	Tumor cell lines (Hyenne et al., 2019)	Melanoma		
Chicken *Gallus gallus* *domesticus*	Chorioallantoic membrane (CAM) cells (Sung et al., 2015)	High‐resolution live imaging of cell migration	+	+++
Mouse *Mus musculus*	Endothelial cells (McCann et al., 2020)	Cell type‐specific EVs in plasma	++	++++
	Red blood cells; heart (Valkov et al., 2021)	Ischaemic heart		
	Mouse tumour cells	Pre‐clinical metastasis (syngeneic grafts) (Ge et al., 2021; Ghoroghi et al., 2021)		
	Human tumour xenografts (Costa‐Silva et al., 2015; Hoshino et al., 2015; Peinado et al., 2012; Zomer et al., 2015; Zomer et al., 2016)	Metastasis		

A non‐exhaustive list of cellular models from different organisms, with particular emphasis on those that are widely used in genetic studies. Nomenclature: genetic tractability and genetic similarity to humans are rated from: weak (‘+’) to strong (‘++++’). Please note that citations are examples only.

Some in vivo studies examine endogenous EVs, usually using fluorescent (Estrada et al., 2022; Hegyesi et al., 2022; Neckles et al., 2019; Nørgård et al., 2022) or bioluminescent tags (Gupta et al., 2020; Luo et al., 2020; Rufino‐Ramos et al., 2022). Pre‐clinical studies with syngeneic models and human cancer cell line xenograft models have allowed tumour and other EVs to be specifically labelled and traced (Driedonks et al., 2022; Hyenne et al., 2019; Liu et al., 2016; Pucci et al., 2016; Wiklander et al., 2015). Functions have been assigned to these EVs, such as roles in metastasis, by pharmacologically or genetically manipulating putative EV biogenesis regulators (Costa‐Silva et al., 2015; Peinado et al., 2012; Wen et al., 2016); however, see caveats on blocking biogenesis that are discussed in Section 7.1 and on the relationship between uptake and function discussed in Section 7.2. Attempts to assess cytoplasmic delivery of EV cargo have involved, for example, EV‐loaded mRNA for the DNA recombinase Cre and its detection in target reporter cells (Zomer et al., 2015). Parabiosis, whereby the circulations of two animals are joined, permits labelled EVs from one mouse to be visualised in the other (Liu, Kou, et al., 2018; Zhang et al., 2022).

Other in vivo studies introduce exogenous EVs into an organism. These EV may be unlabelled when a disease or physiologic outcome is targeted and imaging is not done. For studies with imaging, EVs are often fluorescently or bioluminescently labelled (Alexander et al., 2015; García‐Silva et al., 2021; Kang et al., 2021; Long et al., 2017; Royo et al., 2019). Exogenous EVs have also been labelled, for example, with species‐specific RNAs (Ciullo et al., 2022) and by substances compatible with magnetic resonance imaging (MRI), X‐ray computed tomography (CT) imaging, magnetic particle imaging (MPI), single‐photon emission computed tomography (SPECT) or positron emission tomography (PET) (Arifin et al., 2022; Skotland et al., 2022). There are several caveats to the exogenous approach. Specific labels may affect biodistribution patterns and detectability thresholds (Lázaro‐Ibáñez et al., 2021), necessitating standardization (Herrmann et al., 2021). Exogenous EVs may also differ from endogenous EVs in route and timing of administration (bolus/continuous), dose, non‐EV components of the administered preparation, and of course composition, and physiologic relevance should be carefully pondered (see also Section 8) (Ridder et al., 2014).

For detection and tracking endogenous and exogenous EVs, several additional technical considerations apply. In vivo EV tracking and ex vivo detection will be limited by technique‐specific sensitivity and spatial resolution, for example, a fluorescent signal may represent a single EV, clustered EVs or non‐EV labelled substances. Caveats associated with genetic labels such as the common CD63‐GFP approaches are discussed in Section 6.2 and elsewhere (Verweij et al., 2021). They include the potential disruption of protein, EV or cellular biology through fusion protein (over)expression; possible quenching in acidic compartments; labelling of only specific EV subtypes; labelling of different EV subtypes by a specific marker in different cell types and species; and possible separation of the tag from its host protein. A knock‐in strategy, by which a fluorescently tagged fusion construct (e.g., CD63‐GFP) replaces the respective EV gene in its endogenous locus, or the use of multiple EV markers, provide possible solutions to some of these problems.

**Recommendation**s *(Note: These recommendations are broad, as this section of MISEV2023 is meant to raise awareness of the diversity of in vivo studies and not to make prescriptive guidelines. Innovative new approaches should thrive in diverse organisms to move the field forward.)*
  - Report all details of labelling and detection/imaging technologies to allow replication studies.
  - For exogenous EV administration, report all parameters of administration, including anatomical site, timing (bolus/continuous) and dose.
  - Consider and control for the possible effects of EV labelling on EV biodistribution, pharmacokinetics and function.
  - Consider that pharmacologic or genetic manipulations meant to block EV production in vivo may have off‐target consequences.
  - Consider the possibility of different behaviour of endogenous and exogenous EVs.
    Consensus: 65.5% (654) of MISEV2023 survey respondents agreed “completely,” and 21.6% (216) agreed “mostly” with Section 9: EV analysis in vivo. 0.1% (1) “mostly” disagreed, and 12.7% (127) stated that they had no opinion and/or expertise. No respondents disagreed “completely.”

## 10 CONCLUSIONS

Consensus building was achieved for MISEV2023 through a lengthy process. Suggestions for the new MISEV were gathered from the ISEV community and MISEV2018 authorship through a 2020 survey that received >750 responses (Witwer et al., 2021). A five‐member MISEV2023 organising committee was then formed during the strategic planning session of the ISEV Board of Directors in November 2020, consisting of Deborah Goberdhan, Lorraine O'Driscoll, Clotilde Théry, Joshua Welsh and Kenneth Witwer. An initial MISEV2023 draft went through rounds of review and revision by members of the ISEV board and other individuals, including task force members, who were invited by the organising team because of their subject expertise relevant to specific sections. An exhaustive MISEV2023 survey was circulated to ∼5700 EV researchers, and 1025 responses were received. Refinements were made to the manuscript by the organising committee and invited co‐authors based on these responses. The manuscript was then submitted to the Journal of Extracellular Vesicles. The journal selected more than 30 individual experts to review the manuscript, and reviews were shared with the organising committee along with editorial suggestions. The manuscript was then revised by the organising committee and subject experts, and the ISEV Board of Directors was consulted on matters of timing and logistics. At the request of the ISEV Board, the revised manuscript was sent via survey to all who were involved in developing the guidelines and who had indicated willingness to accept co‐authorship. The results of this authorship survey were used to gauge consensus on each section and to determine the final author lists before resubmission to the journal. The consensus statements at the end of Sections 1 through 9 reflect the complete answers of 998 unique MISEV2023 authorship confirmation survey respondents. There were 1039 responses in total, including several duplicates, one triplicate, three declines, and several incomplete responses. Note that several confirmed authors did not complete the survey for reasons that were deemed valid, including technical issues.

MISEV2023 has compiled recommendations for EV research, from basic to advanced, state‐of‐the‐art technologies and methodologies. As such, it can serve both as a handbook for those new to EV research and also as an inspiration for more advanced science in the field. In generating this document, an overarching goal has been to reach a high degree of agreement from a large group of scientists within the EV community. As with any consensus document, not every co‐author necessarily agrees with every section or every recommendation. We also recognise that new methods will appear, while some advanced techniques may become easier to use: the field is dynamic, not static. Nevertheless, we propose that MISEV2023 describes the current best practice in the field and represents the current consensus position of the extracellular vesicle community.

## AUTHOR CONTRIBUTIONS

**Joshua A. Welsh**: Project administration; visualization; writing—original draft; writing—review and editing. **Deborah C. I. Goberdhan**: Project administration; writing—original draft; writing—review and editing. **Lorraine O'Driscoll**: Project administration; writing—original draft; writing—review and editing. **Edit I. Buzas**: Writing—review and editing. **Cherie Blenkiron**: Writing—review and editing. **Benedetta Bussolati**: Writing—review and editing. **Houjian Cai**: Writing—review and editing. **Dolores Di Vizio**: Writing—review and editing. **Tom A. P. Driedonks**: Writing—review and editing. **Uta Erdbrügger**: Writing—review and editing. **Juan M. Falcon‐Perez**: Writing—review and editing. **Qing‐Ling Fu**: Writing—review and editing. **Andrew F. Hill**: Writing—review and editing. **Metka Lenassi**: Writing—review and editing. **Sai Kiang Lim**: Writing—review and editing. **Mỹ G. Mahoney**: Writing—review and editing. **Sujata Mohanty**: Writing—review and editing. **Andreas Möller**: Writing—review and editing. **Rienk Nieuwland**: Writing—review and editing. **Takahiro Ochiya**: Writing—review and editing. **Susmita Sahoo**: Writing—review and editing. **Ana C. Torrecilhas**: Writing—review and editing. **Lei Zheng**: Writing—review and editing. **Andries Zijlstra**: Writing—review and editing. **Sarah Abuelreich**: Writing—review and editing. **Reem Bagabas**: Writing—review and editing. **Paolo Bergese**: Writing—review and editing. **Esther M. Bridges**: Writing—review and editing. **Marco Brucale**: Writing—review and editing. **Dylan Burger**: Writing—review and editing. **Randy P. Carney**: Writing—review and editing. **Emanuele Cocucci**: Writing—review and editing. **Federico Colombo**: Writing—review and editing. **Rossella Crescitelli**: Writing—review and editing. **Edveena Hanser**: Writing—review and editing. **Adrian L. Harris**: Writing—review and editing. **Norman J. Haughey**: Writing—review and editing. **An Hendrix**: Writing—review and editing. **Alexander R. Ivanov**: Writing—review and editing. **Tijana Jovanovic‐Talisman**: Writing—review and editing. **Nicole A. Kruh‐Garcia**: Writing—review and editing. **Diego Kyburz**: Writing—review and editing. **Cecilia Lässer**: Writing—review and editing. **Kathleen M. Lennon**: Writing—review and editing. **Jan Lötvall**: Writing—review and editing. **Elena S. Martens‐Uzunova**: Writing—review and editing. **Rachel R. Mizenko**: Writing—review and editing. **Lauren A. Newman**: Writing—review and editing. **Andrea Ridolfi**: Writing—review and editing. **Eva Rohde**: Writing—review and editing. **Tatu Rojalin**: Writing—review and editing. **Andrew Rowland**: Writing—review and editing. **Andras Saftics**: Writing—review and editing. **Ursula S. Sandau**: Writing—review and editing. **Julie A. Saugstad**: Writing—review and editing. **Faezeh Shekari**: Writing—review and editing. **Simon Swift**: Writing—review and editing. **Dmitry Ter‐Ovanesyan**: Writing—review and editing. **Juan P. Tosar**: Writing—review and editing. **Zivile Useckaite**: Writing—review and editing. **Francesco Valle**: Writing—review and editing. **Zoltan Varga**: Writing—review and editing. **Edwin van der**: Writing—review and editing. **Martijn J. C. van Herwijnen**: Writing—review and editing. **Marca H. M. Wauben**: Writing—review and editing. **Ann M. Wehman**: Writing—review and editing. **Sarah Williams**: Writing—review and editing. **Andrea Zendrini**: Writing—review and editing. **Alan J. Zimmerman**: Writing—review and editing. **Clotilde Théry**: Project administration; writing—original draft; writing—review and editing. **Kenneth W. Witwer**: Project administration; writing—original draft; writing—review and editing.

## AUTHORSHIP AND PARTICIPATION

MISEV Consortium: **Sarah Abuelreich** (Department of Molecular Medicine, Beckman Research Institute, City of Hope Comprehensive Cancer Center, Duarte, CA, USA), **Samar Ahmad** (Department of Biochemistry, University of Toronto, Toronto, Canada), **Dina AK Ahmed** (University of Westminster, London, UK; Egypt Center for Research and Regenerative Medicine, Cairo, Egypt), **Sarah H Ahmed** (Biotechnology Department, Faculty of Science, Cairo University, Cairo, Egypt; Center of Excellence for Stem Cells and Regenerative Medicine, Zewail City for Science and Technology, Cairo, Egypt), **Elena Aikawa** (Brigham and Women's Hospital, Boston, MA, USA; Harvard Medical School, Boston, MA, USA), **Naveed Akbar** (Division of Cardiovascular Medicine, Radcliffe Department of Medicine, University of Oxford, Oxford, UK), **Kazunari Akiyoshi** (Kyoto University, Kyoto, Japan), **David P Al‐Adra** (Division of Transplantation, Department of Surgery, University of Wisconsin, Madison, WI, USA), **Maimonah E Al‐Masawa** (Centre for Tissue Engineering and Regenerative Medicine, Faculty of Medicine, Universiti Kebangsaan Malaysia Medical Centre, Jalan Yaacob Latif, Kuala Lumpur, Malaysia), **Manuel Albanese** (Department for Clinical Sciences and Community Health (DISCCO), University of Milan, Milan, Italy; National Institute of Molecular Genetics (INGM), Milan, Italy), **Ainhoa Alberro** (Multiple Sclerosis Group, Biogipuzkoa Health Research Institute, San Sebastian, Spain), **María José Alcaraz** (Department of Pharmacology, University of Valencia, Spain), **Jen Alexander‐Brett** (Washington University School of Medicine, St. Louis, MO, USA), **Kimberley L Alexander** (Neurosurgery Department, Chris O'Brien Lifehouse, Camperdown, Australia; Neuropathology Department, Royal Prince Alfred Hospital, Camperdown, Australia; School of Medical Sciences, Faculty of Medicine and Health, The University of Sydney, Australia), **Nilufar Ali** (Boise State University, Boise, ID, USA; Azymus Therapeutics Inc., Boise, ID, USA), **Faisal J Alibhai** (University Health Network, Toronto, Canada), **Susann Allelein** (Fraunhofer Institut for Cell Therapy and Immunology IZI, Leipzig, Germany), **Mark C Allenby** (BioMimetic Systems Engineering Laboratory, School of Chemical Engineering, Faculty of Engineering, Architecture and Information Technology, The University of Queensland, St Lucia, Australia; Centre for Biomedical Technologies, School of Mechanical, Medical, and Process Engineering, Faculty of Engineering, Queensland University of Technology, Brisbane, Australia), **Fausto Almeida** (University of Sao Paulo, Ribeirao Preto Medical School, Sao Paulo, Brazil), **Luis Pereira de Almeida** (Center for Neuroscience and Cell Biology, University of Coimbra, Coimbra, Portugal; Center for Innovative Biomedicine and Biotechnology, University of Coimbra, Coimbra, Portugal; Faculty of Pharmacy, University of Coimbra, Coimbra, Portugal), **Sameh W Almousa** (Wake Forest School of Medicine, Winston‐Salem, NC, USA), **Nihal Altan‐Bonnet** (National Institutes of Health, Bethesda, MD, USA), **Wanessa F Altei** (Molecular Oncology Research Center, Barretos Cancer Hospital, Barretos, Brazil; Radiation Oncology Department, Barretos Cancer Hospital, Barretos, Brazil), **Gloria Alvarez‐Llamas** (Immunology Department, IIS‐Fundacion Jimenez Diaz, Fundacion Jimenez Diaz University Hospital‐UAM, Madrid, Spain; Department of Biochemistry and Molecular Biology, Complutense University, Madrid, Spain), **Cora L Alvarez** (Instituto de Química y Fisicoquímica Biológicas “Prof. Alejandro C. Paladini”, Buenos Aires, Argentina; Consejo Nacional de Investigaciones Científicas y Tecnicas, Argentina; Facultad de Ciencias Exactas y Naturales, University of Buenos Aires, Buenos Aires, Argentina), **Hyo Jung An** (Department of Pathology, Gyeongsang National University Changwon Hospital, Changwon, Republic of Korea; lnstitute of Medical Sciences, Gyeongsang National University, Jinju, Republic of Korea; Department of Pathology, Gyeongsang National University School of Medicine, Jinju, Republic of Korea), **Krishnan Anand** (Department of Chemical Pathology, School of Pathology, Faculty of Health Sciences, University of the Free State, Bloemfontein, South Africa), **Samir EL Andaloussi** (Department of Laboratory Medicine, Karolinska Institutet, Stockholm, Sweden; ME Cell Therapy and Allogenic Stem Cell Transplantation CAST, Karolinska University Hospital, Stockholm, Sweden), **Johnathon D Anderson** (University of California, Davis School of Medicine, Sacramento, CA, USA), **Ramaroson Andriantsitohaina** (INSERM U1046, UMR CNRS 9214, University of Montpellier, Montpellier, France), **Khairul I Ansari** (Inoviq Limited, Notting Hill, Australia), **Achille Anselmo** (FRACTAL—Flow Cytometry Resource, Advanced Cytometry Technical Applications Laboratory, IRCCS Ospedale San Raffaele Scientific Institute, Milan, Italy; Università Vita‐Salute San Raffaele, Milan, Italy), **Anna Antoniou** (Department for Neurodegenerative Diseases and Geriatric Psychiatry, University of Bonn Medical Center, Bonn, Germany; German Center for Neurodegenerative Diseases, Bonn, Germany), **Farrukh Aqil** (Department of Medicine, University of Louisville, Louisville, KY, USA; Brown Cancer Center, University of Louisville, Louisville, KY, USA), **Tanina Arab** (Department of Molecular and Comparative Pathobiology, Johns Hopkins University School of Medicine, Baltimore, MD, USA), **Fabienne Archer** (IVPC UMR754, INRAE, EPHE, Universite Claude Bernard Lyon 1, Lyon, France), **Syrine Arif** (Faculté de Médecine, Université Laval, Quebec, Canada; Centre de Recherche du Centre Hospitalier Universitaire de Québec, Université Laval, Quebec, Canada; Centre de Recherche en Organogénèse Expérimentale de l'Université Laval/LOEX, Quebec, Canada), **David A Armstrong** (Dartmouth Health, Lebanon, NH, USA; Veterans Affairs Medical Center, White River Junction, VT, USA), **Onno J Arntz** (Experimental Rheumatology, Radboud University Medical Center, Nijmegen, The Netherlands), **Pierre Arsène** (Mursla Ltd, Cambridge, UK; Exosla Ltd, Cambridge, UK), **Luis Arteaga‐Blanco** (Laboratory on Thymus Research, Oswaldo Cruz Institute/Fiocruz, Rio de Janeiro, Brazil), **Nandini Asokan** (INSERM U1109, Strasbourg France; Center Research Biomedicine De Strasbourg, France), **Trude Aspelin** (Department of Medical Biochemistry, Oslo University Hospital, Oslo, Norway), **Georgia K Atkin‐Smith** (Walter and Eliza Hall Institute of Medical Research, Melbourne, Australia; La Trobe Institute for Molecular Science, La Trobe University, Melbourne, Australia), **Dimitri Aubert** (Vesiculab Ltd, Nottingham, UK), **Kanchana K Ayyar** (Boston Medical Center, Boston, MA, USA), **Maryam Azlan** (School of Health Sciences, Universiti Sains Malaysia, George Town, Malaysia), **Ioannis Azoidis** (Johns Hopkins University School of Medicine, Baltimore, MD, USA; University of Birmingham, Birmingham, UK), **Anaïs Bécot** (INSERM U1266, Institute of Psychiatry and Neurosciences of Paris, Paris, France), **Jean‐Marie Bach** (Oniris, INRAE, IECM, Nantes, France), **Daniel Bachurski** (Center for Integrated Oncology Aachen Bonn Cologne Duesseldorf, Department I of Internal Medicine, Faculty of Medicine, University of Cologne, and University Hospital Cologne, Cologne, Germany; CECAD Center of Excellence on Cellular Stress Responses in Aging‐Associated Diseases, University of Cologne, Cologne, Germany; Center for Molecular Medicine Cologne, University of Cologne, Cologne, Germany), **Seoyoon Bae** (Department of Life Sciences, Pohang University of Science and Technology, Pohang, Republic of Korea), **Reem Bagabas** (Department of Molecular Medicine, Beckman Research Institute, City of Hope Comprehensive Cancer Center, Duarte, CA, USA), **Roger Olofsson Bagge** (Sahlgrenska Center for Cancer Research, Department of Surgery, Institute of Clinical Sciences, Sahlgrenska Academy, University of Gothenburg, Gothenburg, Sweden; Department of Surgery, Sahlgrenska University Hospital, Gothenburg, Sweden; Wallenberg Centre for Molecular and Translational Medicine, Institute of Clinical Sciences, Sahlgrenska Academy, University of Gothenburg, Gothenburg, Sweden), **Monika Baj‐Krzyworzeka** (Department of Clinical Immunology, Medical College, Jagiellonian University, Krakow, Poland), **Leonora Balaj** (Department of Neurosurgery, Massachusetts General Hospital, Harvard Medical School, Boston, MA, USA), **Carolina Balbi** (Center for Molecular Cardiology, University of Zurich, Zurich, Switzerland; Laboratory of Cellular and Molecular Cardiology, Istituto Cardiocentro Ticino‐EOC, Lugano, Switzerland), **Bas WM van Balkom** (Department of Nephrology and Hypertension, University Medical Center Utrecht, Utrecht, The Netherlands), **Abhijna R Ballal** (Centre for Molecular Neurosciences, Kasturba Medical College Manipal, Manipal Academy of Higher Education, Manipal, India), **Afsareen Bano** (Maharshi Dayanand University, Rohtak, Haryana, India), **Sébastien Banzet** (Institut de Recherche Biomédicale des Armées, Brétigny sur Orge, France; INSERM UMR‐MD‐1197, Clamart, France; Centre de Transfusion des Armées, Clamart, France), **Yonis Bare** (Institut de Recherche en Infectiologie de Montpellier (IRIM), UMR9004 CNRS, Montpellier, France; University of Montpellier, Montpellier, France), **Lucio Barile** (Cardiovascular Theranostics, Istituto Cardiocentro Ticino, Ente Ospedaliero Cantonale, Bellinzona, Switzerland; Euler Institute, Faculty of Biomedical Sciences, Università Svizzera Italiana, Lugano, Switzerland), **Bahnisikha Barman** (Vanderbilt University School of Medicine, Nashville, TN, USA), **Isabel Barranco** (Department of Medicine and Animal Surgery, Veterinary Science, University of Murcia, Murcia, Spain), **Valeria Barreca** (Istituto Superiore di Sanità, Rome, Italy), **Geneviève Bart** (Developmental Biology Laboratory, Disease Networks RU, Faculty of Biochemistry and Molecular Medicine, University Oulu, Oulu, Finland), **Natasha S Barteneva** (Nazarbayev University, Astana, Kazakhstan), **Manuela Basso** (Department of Cellular, Computational and Integrative Biology (CIBIO), University of Trento, Trento, Italy), **Mona Batish** (Department of Medical and Molecular Sciences, University of Delaware, Newark, DE,USA), **Natalie R Bauer** (Frederick P. Whiddon College of Medicine, University of South Alabama, Mobile, AL, USA), **Amy A Baxter** (La Trobe University, Melbourne, Australia), **Wilfried W Bazié** (Axe de Recherche Maladies Infectieuses et Immunitaires, Centre de Recherche du CHU de Québec, Université Laval, Quebec, Canada; Programme de Recherche sur les Maladies Infectieuses, Centre Muraz, Institut National de Santé Publique, Bobo‐Dioulasso, Burkina Faso), **Erica Bazzan** (Department of Cardiac, Thoracic, Vascular Sciences and Public Health, University of Padua, Padua, Italy), **Joel EJ Beaumont** (Department of Radiotherapy, GROW‐School for Oncology and Reproduction, Maastricht University Medical Centre+, Maastricht, The Netherlands), **Mary Bebawy** (Private consultant), **Maarten P Bebelman** (Max Planck Institute of Molecular Cell Biology and Genetics, Dresden, Germany; Amsterdam UMC, Amsterdam, The Netherlands), **Apolonija Bedina‐Zavec** (National Institute of Chemistry, Ljubljana, Slovenia), **Danielle J Beetler** (Mayo Clinic Center for Clinical and Translational Science, Rochester, MN, USA; Mayo Clinic Department of Cardiovascular Medicine, Jacksonville, Florida, USA; Mayo Clinic Graduate School of Biomedical Sciences, Rochester, Minnesota, USA), **Tamás Beke‐Somfai** (Institute of Materials and Environmental Chemistry, Biomolecular Self‐assembly Research Group, Reserach Centre for Natural Sciences, Budapest, Hungary), **Clémence Belleannée** (Faculty of Medicine, Université Laval, Quebec, Canada; CHU de Quebec Research Center, Quebec, Canada), **Birke J Benedikter** (University Eye Clinic Maastricht, MHeNs School for Mental Health and Neuroscience, Maastricht University Medical Center+, Maastricht, The Netherlands), **Berglind E Benediktsdóttir** (Faculty of Pharmaceutical Sciences, School of Health Sciences, University of Iceland, Reykjavik, Iceland), **Anna C Berardi** (Ospedale Santo Spirito, Pescara, Italy), **Mathilde Bergamelli** (Division of Obstetrics and Gynecology, Department of Clinical Science, Intervention and Technology, Karolinska Institutet, Stockholm, Sweden; Department of Gynecology and Reproductive Medicine, Karolinska University Hospital, Stockholm, Sweden), **Paolo Bergese** (Department of Molecular and Translational Medicine, University of Brescia, Brescia, Italy; Center for Colloid and Surface Science (CSGI), Florence, Italy; National Center for Gene Therapy and Drugs based on RNA Technology, Padua, Italy), **Irene Bertolini** (Immunology, Microenvironment and Metastasis Program, The Wistar Institute, Philadelphia, PA, USA), **Asima Bhattacharyya** (School of Biological Sciences, National Institute of Science Education and Research (NISER) Bhubaneswar, An OCC of Homi Bhabha National Institute, Odisha, India), **Suvendra N Bhattacharyya** (CSIR‐Indian Institute of Chemical Biology, Kolkata, India; Department of Pharmacology and Experimental Neuroscience, University of Nebraska Medical Center, Omaha, NE, USA), **Steven J Biller** (Wellesley College, Wellesley, MA, USA), **Clotilde Billottet** (BRIC‐ Bordeaux Institute of Oncology‐Inserm U1312, University of Bordeaux, Bordeaux, France), **John J Bissler** (St. Jude Children's Reseach Hospital, Memphis, TN, USA; Le Bonheur Children's Hospital, Memphis, TN, USA; University of Tennessee Health Sciences Center, Memphis, TN, USA), **Olivier Blanc‐Brude** (INSERM, Paris Center for Cardiovascular Research‐ParCC, Université Paris Cité, Paris, France), **Cherie Blenkiron** (Faculty of Medical and Health Sciences, The University of Auckland, Auckland, New Zealand), **Charles J Blijdorp** (Erasmus MC, Rotterdam, The Netherlands), **Sylwia Bobis‐Wozowicz** (Department of Cell Biology, Faculty of Biochemistry, Biophysics and Biotechnology, Jagiellonian University, Krakow, Poland), **Victor Bodart‐Santos** (Department of Neuroscience, Mayo Clinic Florida, Jacksonville, FL, USA), **Bernadett R Bodnár** (Department of Genetics, Cell‐ and Immunobiology, Semmelweis University, Budapest, Hungary; HCEMM‐SU Extracellular Vesicle Research Group, Semmelweis University, Budapest, Hungary), **Eric Boilard** (Centre de Recherche du Centre Hospitalier Universitaire de Québec, Université Laval, Quebec, Canada; Centre de Recherche ARThrite (Arthrite Recherche Traitements) de l'Université Laval, Quebec, Canada), **Wilfrid Boireau** (Institut Femto‐ST, CNRS, Université de Franche‐Comté, Besançon, France), **Vladimir Bokun** (Department of Metabolism, Digestion and Reproduction, Imperial College London, London, UK), **Stephanie M Bollard** (School of Medicine, University College Dublin, Dublin, Ireland), **Sveva Bollini** (Department of Experimental Medicine (DIMES), University of Genova, Genova, Italy; IRCCS Ospedale Policlinico San Martino, Genova, Italy), **Antonella Bongiovanni** (Cell‐Tech HUB at Institute for Research and Biomedical Innovation, National Research Council of Italy (CNR), Palermo, Italy), **Laura Bongiovanni** (Department of Veterinary Medicine, University of Teramo, Teramo, Italy; Department of Biomolecular Health Sciences, Faculty of Veterinary Medicine, Utrecht University, Utrecht, The Netherlands), **Amandine Bonifay** (C2VN, INSERM 1263, INRAE 1260, Aix‐Marseille University, Marseille, France), **Marni D Boppart** (University of Illinois at Urbana‐Champaign, Urbana‐Champaign, IL, USA; Beckman Institute for Advanced Science and Technology, University of Illinois at Urbana‐Champaign, Urbana‐Champaign, IL, USA), **Francesc E Borràs** (REMAR‐IVECAT Group, Germans Trias i Pujol Research Institute (IGTP) & Nephrology Department, University Hospital Germans Trias i Pujol (HUGTiP), Can Ruti Campus, Badalona, Spain; Department of Cell Biology, Physiology and Immunology, Universitat de Barcelona (UB), Barcelona, Spain), **Steffi Bosch** (Oniris IECM Laboratory, INRAE, USC1383, Nantes, France), **Daniela Boselli** (FRACTAL—Flow Cytometry Resource, Advanced Cytometry Technical Applications Laboratory, IRCCS Ospedale San Raffaele Scientific Institute, Milan, Italy), **Massimo Bottini** (Department of Experimental Medicine, University of Rome Tor Vergata, Rome, Italy; Sanford Burnham Prebys, La Jolla, CA, USA), **Jeff Bouffard** (Concordia University, Montreal, Canada), **Chantal M Boulanger** (Paris‐Cardiovascular Research Center, INSERM, Université Paris Cité, Paris, France), **Paul C Boutros** (Department of Human Genetics, University of California, Los Angeles, Los Angeles, CA, USA; Department of Urology, University of California, Los Angeles, Los Angeles, CA, USA; Jonsson Comprehensive Cancer Center, University of California Los Angeles, Los Angeles, CA, USA), **Oscar Boyadjian** (McGill University, Montreal, Canada), **Anders T Boysen** (Department of Clinical Medicine, Aarhus University, Aarhus, Denmark), **Batuhan T Bozkurt** (Department of Biomedical Engineering, University of California, Davis, Davis, CA, USA), **Kyle P Bramich** (La Trobe Institute for Molecular Science, La Trobe University, Melbourne, Australia), **Fabian Braun** (III. Department of Medicine, University Medical Center Hamburg‐Eppendorf, Hamburg, Germany; Hamburg Center for Kidney Health (HCKH), University Medical Center Hamburg‐Eppendorf, Hamburg, Germany; Martin Zeitz Centre for Rare Diseases, University Medical Center Hamburg‐Eppendorf, Hamburg, Germany), **Rocío del Carmen Bravo‐Miana** (Multiple Sclerosis Group, Biogipuzkoa Health Research Institute, San Sebastian, Spain), **Xandra O Breakefield** (Massachusetts General Hospital and Harvard Medical School, Boston, MA, USA), **Santra Brenna** (Neurology Department, Experimental Research in Stroke and Inflammation (ERSI), University Medical Center Hamburg‐Eppendorf, Hamburg, Germany), **Kieran Brennan** (School of Biomolecular and Biomedical Science, University College Dublin, Dublin, Ireland; Conway Institute of Biomolecular and Biomedical Research, University College Dublin, Dublin, Ireland), **Meadhbh Á Brennan** (University of Galway, Galway, Ireland), **Koen Breyne** (Molecular Neurogenetics Unit, Department of Neurology, Massachusetts General Hospital, Harvard Medical School, Boston, MA, USA), **Esther M Bridges** (Weatherall Institute of Molecular Medicine, University of Oxford, Oxford, UK), **David R Brigstock** (Center for Clinical and Translational Research, The Research Institute at Nationwide Children's Hospital, Columbus, OH, USA; Department of Surgery, Wexner Medical Center, The Ohio State University, Columbus OH, USA), **Alain R Brisson** (University of Bordeaux, Pessac, France), **Chaya Brodie** (Mina and Everard Goodman Faculty of Life Sciences, Bar‐Ilan University, Ramat‐Gan, Israel; Institute of Nanotechnology and Advanced Materials (BINA), Bar‐Ilan University, Ramat‐Gan , Israel; Hermelin Brain Tumor Center, Henry Ford Health, Detroit, MI 48202, USA), **Marco Brucale** (Consiglio Nazionale delle Ricerche—Istituto per lo Studio dei Materiali Nanostrutturati, Bologna, Italy; Consorzio Interuniversitario per lo Sviluppo dei Sistemi a Grande Interfase, Florence, Italy), **Katelyn A Bruno** (University of Florida, Gainesville, FL, USA; Mayo Clinic, Jacksonville, FL, USA), **Cecilia Bucci** (Department of Experimental Medicine, University of Salento, Lecce, Italy), **Shilpa Buch** (University of Nebraska Medical Center, Omaha, NE, USA), **Amy H Buck** (Institute of Immunology & Infection Research, University of Edinburgh, Edinburgh, UK), **Mátyás Bukva** (Laboratory of Microscopic Image Analysis and Machine Learning, Hungarian Research Network (HUN‐REN), Biological Research Centre, Szeged, Hungary; Department of Immunology, University of Szeged, Szeged, Hungary; Doctoral School of Interdisciplinary Medicine, University of Szeged, Szeged, Hungary), **Jeff WM Bulte** (Russell H. Morgan Dept. of Radiology and Radiological Science, Division of MR Research, Johns Hopkins University School of Medicine, Baltimore, MD, USA; Cellular Imaging Section and Vascular Biology Program, Institute for Cell Engineering, Johns Hopkins University School of Medicine, Baltimore, MD, USA), **Sandra Buratta** (Department of Chemistry, Biology and Biotechnology, University of Perugia, Perugia, Italy), **Dylan Burger** (Kidney Research Centre, Ottawa Hopsital Research Institute, Ottawa, Canada; Department of Cellular and Molecular Medicine, University of Ottawa, Ottawa, Canada; School of Pharmaceutical Sciences, University of Ottawa, Ottawa, Canada), **Olivier Burgy** (INSERM U1231, Faculty of Medicine and Pharmacy, University of Bourgogne‐Franche Comté, Dijon, France), **Julia V Burnier** (Cancer Research Program, Research Institute of the McGill University Health Centre, Montreal, Canada; Gerald Bronfman Department of Oncology, McGill University, Montreal, Canada; Department of Pathology, McGill University, Montreal, Canada), **Kaiping Burrows** (Laureate Institute for Brain Research, Tulsa, OK, USA), **Sara Busatto** (Vascular Biology Program, Boston Children's Hospital, Boston, MA, USA; Department of Surgery, Boston Children's Hospital and Harvard Medical School, Boston, MA, USA), **Benedetta Bussolati** (Department of Molecular Biotechnology and Health Sciences, University of Turin, Turin, Italy), **Edit I Buzas** (Department of Genetics, Cell‐ and Immunobiology, Semmelweis University, Budapest, Hungary; HCEMM‐SU Extracellular Vesicle Research Group, Semmelweis University, Budapest, Hungary; HUN‐REN‐SU Translational Extracellular Vesicle Research Group, Semmelweis University, Budapest, Hungary), **Krisztina Buzas** (HUN‐REN Biological Research Centre, Szeged, Hungary; Department of Immunology, University of Szeged, Szeged, Hungary), **J Brian Byrd** (Department of Medicine, University of Michigan, Ann Arbor, MI, USA), **Albano Cáceres‐Verschae** (Department of Oncology‐Pathology, Karolinska Institutet, Stockholm, Sweden; Centro de Innovación e Investigación Biomédica, Universidad de Los Andes, Santiago, Chile; Centro de Biología Celular y Biomedicina, Universidad San Sebastián, Santiago, Chile), **Houjian Cai** (University of Georgia, Athens, GA, USA), **Hugo R Caires** (i3S‐Institute for Research and Innovation in Health, University of Porto, Porto, Portugal), **Carmen Campos‐Silva** (Universidad Loyola, Sevilla, España), **Giovanni Camussi** (Department of Medical Sciences, University of Turin, Turin, Italy), **Paola de Candia** (Dipartimento di Medicina Molecolare e Biotecnologie Mediche, Università degli Studi di Napoli Federico II, Napoli, Italy), **Carmen Carceller** (Department of Dentistry, Faculty of Health Sciences, Universidad Europea de Valencia, Valencia, Spain), **Carmen Fernandez‐Becerra** (ISGlobal, Barcelona Institute for Global Health, Hospital Clínic‐Universitat de Barcelona, Barcelona, Spain; IGTP, Germans Trias i Pujol Health Research Institute, Badalona, Spain; CIBERINFEC, ISCIII‐CIBER de Enfermedades Infecciosas, Instituto de Salud Carlos III, Madrid, Spain), **Randy P Carney** (Department of Biomedical Engineering, University of California, Davis, Davis, CA, USA), **Alexis G Murillo Carrasco** (Centro de Investigação Translacional em Oncologia, Instituto do Câncer do Estado de São Paulo, São Paulo, Brazil; Comprehensive Center for Precision Oncology, Universidade de São Paulo, São Paulo, Brazil), **David RF Carter** (Evox Therapeutics Limited, Oxford, UK; Department of Biological and Medical Sciences, Faculty of Health and Life Sciences, Oxford Brookes University, Oxford, UK), **Sara Cavallaro** (Krantz Family Center for Cancer Research, Massachusetts General Hospital, Boston, MA, USA; Department of Medicine, Harvard Medical School, Boston, MA, USA), **Serena Cavallero** (Department of Public Health and Infectious Diseases, Sapienza University of Rome, Rome, Italy; Pasteur Institute Italy Fondazione Cenci Bolognetti, Rome,Italy), **Sophie Cavallero** (Département des Effets Biologiques des Rayonnements, Institut de Recherche Biomédicale des Armées (IRBA), Bretigny sur orge, France), **Cristóbal Cerda‐Troncoso** (Centro de Biología Celular y Biomedicina, Facultad de Medicina y Ciencia, Universidad San Sebastián, Santiago, Chile; Centro Ciencia & Vida, Fundación Ciencia & Vida, Santiago, Chile; Department of Human Genetics, KU Leuven, Leuven, Belgium), **Richard Chahwan** (Institute of Experimental Immunology, University of Zurich, Zurich, Switzerland; Faculty of Medicine, University of Zurich, Zurich, Switzerland; Faculty of Science, University of Zurich, Zurich, Switzerland), **Renata Chalupská** (AstraZeneca, Mölndal, Sweden), **Lawrence W Chamley** (Department of Obstetrics and Gynaecology, University of Auckland, New Zealand; Hub for Extracellular Investigations, University of Auckland, New Zealand), **Partha K Chandra** (Department of Pharmacology, Tulane University School of Medicine, New Orleans, LA, USA), **Wen‐Wei Chang** (Department of Biomedical Sciences, Chung Shan Medical University, Taichung City, Taiwan), **Al Charest** (Department of Medicine, Beth Israel Deaconess Medical Center, Boston, MA, USA; Harvard Medical School, Boston, MA, USA), **Chihchen Chen** (Institute of Nanoengineering and Microsystems, Department of Power Mechanical Engineering, National Tsing Hua University, Hsinchu, Taiwan), **Hao Chen** (Medical School of Nantong University, Nantong, China; Guangzhou Medical University, Guangzhou, China), **Qiang Chen** (Cancer Center, Faculty of Health Sciences, University of Macau, Taipa, Macau SAR, China; MOE Frontier Science Centre for Precision Oncology, University of Macau, Taipa, Macau SAR, China), **Shuai Chen** (Department of Reproduction Biology/Leibniz‐Institute for Zoo and Wildlife Research (IZW), Berlin, Germany), **Siyu Chen** (The University of Queensland, Brisbane, Australia; The University of California, Los Angeles, Los Angeles, CA, USA), **Yunxi Chen** (Research Institute of McGill University Health Centre, Montreal, Canada), **Lesley Cheng** (Department of Biochemistry and Chemistry, La Trobe Institute for Molecular Science, La Trobe University, Melbourne, Australia), **Vasiliy S Chernyshev** (National Medical Research Center for Obstetrics, Gynecology and Perinatology named after Academician V.I. Kulakov, Moscow, Russia), **Venkatesh Kumar Chetty** (University Hospital Essen, Essen, Germany), **Sai V Chitti** (Department of Biochemistry, La Trobe Institute for Molecular Science, La Trobe University, Melbourne, Australia), **Ssang‐Goo Cho** (Department of Stem Cell and Regenerative Biotechnology, Molecular & Cellular Reprogramming Center and Institute of Advanced Regenerative Science, Konkuk University, Seoul, Republic of Korea; StemExOne Co., Ltd., Seoul, Republic of Korea), **Yoon‐Kyoung Cho** (Department of Biomedical Engineering, Ulsan National Institute of Science and Technology, Ulsan, Republic of Korea; Center for Soft and Living Matter, Institute for Basic Science, Ulsan, Republic of Korea), **Byeong Hyeon Choi** (Department of Thoracic and Cardiovascular Surgery, Korea University Guro Hospital, College of Medicine, Korea University, Seoul, Republic of Korea; Image Guided Precision Cancer Surgery Institute, Korea University, Seoul, Republic of Korea), **Somchai Chutipongtanate** (MILCH and Novel Therapeutics Lab, Division of Epidemiology, Department of Environmental and Public Health Sciences, University of Cincinnati College of Medicine, Cincinnati, OH, USA), **Maria Elena Cicardi** (Jefferson Weinberg Center for ALS, Department of Neuroscience, Thomas Jefferson University, Philadelphia, PA, USA), **Anna Cifuentes‐Rius** (Exopharm Ltd, Melbourne, Australia), **Alessandra Ciullo** (Smidt Heart Institute, Cedars‐Sinai Medical Center, Los Angeles, CA, USA), **Aled Clayton** (Division of Cancer and Genetics, School of Medicine, Cardiff University, Cardiff, UK), **Jacob A Cleary** (Wake Forest School of Medicine, Winston‐Salem, NC, USA), **Federico Cocozza** (INSERM U932, Institut Curie Centre de Recherche, PSL Research University, Paris, France), **Emanuele Cocucci** (Division of Pharmaceutics and Pharmacology, College of Pharmacy, The Ohio State University, Columbus, OH, USA; Comprehensive Cancer Center, The Ohio State University, Columbus, OH, USA), **Robert J Coffey** (Epithelial Biology Center, Department of Medicine, Vanderbilt University Medical Center, Nashville, TN, USA), **Federica Collino** (Department of Clinical Sciences and Community Health, University of Milano, Milan, Italy.; Pediatric Nephrology, Dialysis and Transplant Unit, Fondazione IRCCS Ca' Granda‐Ospedale Maggiore Policlinico, Milano, Italy), **Federico Colombo** (Division of Pharmaceutics and Pharmacology, College of Pharmacy, The Ohio State University, Columbus, OH, USA), **Pascal Colosetti** (CarMeN Laboratory, UMR INRAE 1397/INSERM 1060, University of Lyon, Lyon, France), **Alvaro Compañ‐Bertomeu** (Universitat de Valencia, Valencia, Spain; Farmacia María Luisa Bertomeu Navajas, Valencia, Spain; Universidad Internacional de Valencia, Valencia, Spain), **Julie Constanzo** (Institut de Recherche en Cancérologie de Montpellier (IRCM), INSERM U1194, Nuclear Medicine Department, Institut régional du Cancer de Montpellier (ICM), University of Montpellier, Montpellier, France), **Denis Corbeil** (Biotechnology Center (BIOTEC) and Center for Molecular and Cellular Bioengineering, Technische Universität Dresden, Dresden, Germany; Tissue Engineering Laboratories, Medizinische Fakultät der Technischen Universität Dresden, Dresden, Germany), **Anabela Cordeiro‐da‐Silva** (Faculty of Pharmacy, University of Porto, Porto, Portugal; Institute for Research and Innovation in Health, University of Porto, Porto, Portugal), **Júlia Costa** (Instituto de Tecnologia Química e Biológica António Xavier, Universidade Nova de Lisboa, Lisbon, Portugal), **Yvonne Couch** (St Hilda's College, University of Oxford, Oxford, UK; Somerville College, University of Oxford, Oxford, UK), **Yvan Courageux** (ICREC Research Program, Health Science Research Institute Germans Trias i Pujol (IGTP), Badalona, Spain; Heart Institute (iCor), Germans Trias i Pujol University Hospital, Badalona, Spain; Department of Biochemistry, Molecular Biology and Biomedicine, Universitat Autònoma de Barcelona (UAB), Barcelona, Spain), **Kelly Coutant** (Faculté de Médecine, Université Laval, Quebec, Canada), **Beth Coyle** (Children's Brain Tumour Research Centre, School of Medicine, University of Nottingham Biodiscovery Institute, University of Nottingham, University Park, Nottingham, UK), **Rossella Crescitelli** (Sahlgrenska Center for Cancer Research, Department of Surgery, Institute of Clinical Sciences, Sahlgrenska Academy, University of Gothenburg, Gothenburg, Sweden; Wallenberg Centre for Molecular and Translational Medicine, Institute of Clinical Sciences, Sahlgrenska Academy, University of Gothenburg, Gothenburg, Sweden), **Marina Cretich** (Istituto di Scienze e Tecnologie Chimiche “Giulio Natta”, Consiglio Nazionale delle Ricerche, Milan, Italy), **André Cronemberger‐Andrade** (MSC—Matière et Systèmes Complexes/CNRS, Université Paris Cité, Paris, France), **Rachel E Crossland** (Translational and Clinical Research Institute, Faculty of Medical Science, Newcastle University, Newcastle upon Tyne, UK), **Marcela A Cucher** (Department of Microbiology, School of Medicine, University of Buenos Aires, Buenos Aires, Argentina; Institute of Research on Microbiology and Medical Parasitology (IMPaM, UBA‐CONICET), University of Buenos Aires, Buenos Aires, Argentina.), **Malgorzata Czystowska‐Kuzmicz** (Department of Biochemistry, Medical University of Warsaw, Warsaw, Poland), **Pasquale D'Acunzo** (Center for Dementia Research, Nathan S. Kline Institute for Psychiatric Research, Orangeburg, NY, USA; Department of Psychiatry, New York University Grossman School of Medicine, New York, NY, USA), **Igea D'Agnano** (Institute for Biomedical Technologies‐CNR, Segrate, Italy), **Vito G D'Agostino** (Department of Cellular, Computational and Integrative Biology (CIBIO), University of Trento, Trento, Italy), **Daniele D'Arrigo** (Laboratoire Matière et Systèmes Complexes , CNRS UMR 7057, Université Paris Cité, Paris, France; Abbelight, Cachan, France), **Crislyn D'Souza‐Schorey** (Department of Biological Sciences, University of Notre Dame, Notre Dame, IN, USA), **Raghubendra S Dagur** (SciBiz consulting, LLC, Sacramento, CA, USA), **Kirsty M Danielson** (University of Otago, Wellington, New Zealand), **Saumya Das** (Massachusetts General Hospital, Boston, MA, USA), **Thibaud Dauphin** (Oniris VetAgroBio Nantes, INRAE, IECM, Nantes, France), **Sean M Davidson** (University College London, London, UK), **Owen G Davies** (School of Sport Exercise and Health Sciences, Loughborough University, Loughborough, UK), **Rebecca L Davies** (Centre for Regenerative Medicine Research, School of Medicine, Keele University, Keele, UK; Robert Jones and Agnes Hunt Orthopaedic Hospital, Oswestry, UK), **Chelsea N Davis** (Aberystwyth University, Aberystwyth, UK), **Gagan Deep** (Department of Cancer Biology, Wake Forest University School of Medicine, Winston‐Salem, NC, USA; Sticht Center for Healthy Aging and Alzheimer's Prevention, Wake Forest School of Medicine, Winston‐Salem, North Carolina, USA; Atirum Health Wake Forest Baptist Comprehensive Cancer Center, Wake Forest University School of Medicine, Winston‐Salem, North Carolina, USA), **Jonathan Degosserie** (Department of Laboratory Medicine, CHU UCL Namur, Université Catholique de Louvain, Yvoir, Belgium; Namur Molecular Tech, CHU UCL Namur, Université Catholique de Louvain, Yvoir, Belgium; Namur Research Institute of Life Sciences, CHU UCL Namur, Université de Namur, Yvoir, Belgium), **Mats Van Delen** (Laboratory of Experimental Hematology, Vaccine and Infectious Disease Institute (Vaxinfectio), University of Antwerp, Antwerp, Belgium; Health Department, Flemish Institute for Technological Research (VITO), Mol, Belgium), **Vatsal Deliwala** (Australian Institute for Bioengineering and Nanotechnology, The University of Queensland, Brisbane, Australia; Centre for Advanced Imaging, The University of Queensland, Brisbane, Australia; School of Chemical Engineering, The University of Queensland, Brisbane, Australia), **Elizabeth R Dellar** (Nuffield Department of Clinical Neurosciences, John Radcliffe Hospital, University of Oxford, Oxford, UK), **Jan Van Deun** (Department of Dermatology, Universitätsklinikum Erlangen, Friedrich‐Alexander‐Universität Erlangen‐Nürnberg, Erlangen, Germany), **Apurba Dev** (Uppsala University, Uppsala, Sweden; KTH Royal Institute of Technology, Stockholm, Sweden), **Sarah Deville** (Ghent University, Ghent, Belgium), **Andrew Devitt** (School of Biosciences, College of Health & Life Sciences, Aston University, Birmingham, UK), **Bert Dhondt** (Department of Human Structure and Repair, Ghent University, Ghent, Belgium), **Lothar C Dieterich** (European Center of Angioscience, Medical Faculty Mannheim, Heidelberg University, Mannheim, Germany), **Dirk P Dittmer** (Lineberger Comprehensive Cancer Center, Department of Microbiology and Immunology, The University of North Carolina at Chapel Hill, Chapel Hill, NC, USA), **Brian Dobosh** (Department of Pediatrics, Emory University School of Medicine, Atlanta, GA, USA; Center for CF and Airways Disease Research, Children's Healthcare of Atlanta, Atlanta, GA, USA), **Gabriella Dobra** (Laboratory of Microscopic Image Analysis and Machine Learning, Hungarian Research Network (HUN‐REN), Biological Research Centre, Szeged, Hungary; Department of Immunology, University of Szeged, Szeged, Hungary), **Navneet Dogra** (Department of Pathology, Molecular and Cell‐Based Medicine, Icahn Genomics Institute, New York, NY, USA), **Eisuke Dohi** (Department of Mental Disorder Research, National Institute of Neuroscience, National Center of Neurology and Psychiatry, Tokyo, Japan), **Vincenza Dolo** (Department of Life, Health and Environmental Sciences, University of L'Aquila, L'Aquila, Italy), **Timothy V Domashevich** (Departments of Ophthalmology, University of Colorado Anschutz Medical Campus, Aurora, CO, USA), **Massimo Dominici** (Laboratory of Cellular Therapy, Division of Oncology, Department of Medical and Surgical Sciences for Children & Adults, University of Modena and Reggio Emilia, Modena, Italy; Division of Oncology, Department of Oncology and Hematology, University Hospital of Modena, Modena, Italy), **Liang Dong** (Department of Urology, Renji Hospital, Shanghai Jiao Tong University School of Medicine, Shanghai, China), **Etienne Doré** (Centre de Recherche du Centre Hospitalier Universitaire de Québec, Université Laval, Quebec, Canada; Centre de Recherche ARThrite (Arthrite Recherche Traitements) de l'Université Laval, Quebec, Canada), **Rebecca A Dragovic** (Nuffield Department of Women's and Reproductive Health, Oxford Endometriosis CaRe Centre, University of Oxford, Oxford, UK), **Tom AP Driedonks** (Department CDL Research, University Medical Center Utrecht, Utrecht, The Netherlands), **Lila Drittanti** (AGS Therapeutics, Paris, France; Markets & Listing, Paris, France), **Marvin Droste** (Department of Pediatrics II, Pediatric Nephrology, University Hospital Essen, University of Duisburg‐Essen, Essen, Germany), **Wei Duan** (Deakin University, Geelong, Australia), **Esmahan Durmaz** (School of Optometry and Vision Sciences, Cardiff University, Cardiff, UK), **Suman Dutta** (Nuffield Department of Clinical Neurosciences, John Radcliffe Hospital, University of Oxford, Oxford, UK; Kavli Institute for Nanoscience Discovery, Dorothy Crowfoot Hodgkin Building, University of Oxford, Oxford, UK), **Takanori Eguchi** (Department of Dental Pharmacology, Faculty of Medicine, Dentistry and Pharmaceutical Sciences, Okayama University, Okayama, Japan; Advanced Research Center for Oral and Craniofacial Sciences, Okayama University, Okayama, Japan), **Ramon M Eichenberger** (Institute of Chemistry and Biotechnology, Zurich University of Applied Sciences, Wädenswil, Switzerland), **Erez Eitan** (NeuroDex INC, Natick, MA, USA), **Karin Ekström** (Sahlgrenska Center for Cancer Research, Department of Surgery, Institute of Clinical Sciences, Sahlgrenska Academy, University of Gothenburg, Gothenburg, Sweden; Wallenberg Centre for Molecular and Translational Medicine, Institute of Clinical Sciences, Sahlgrenska Academy, University of Gothenburg, Gothenburg, Sweden), **Maria Eldh** (Division of Immunology and Allergy, Department of Medicine Solna, Karolinska Institutet, Stockholm, Sweden), **Celine Elie‐Caille** (FEMTO‐ST Institute, CNRS UMR‐6174, Université de Bourgogne Franche‐Comté, Besançon, France), **Agustin Enciso‐Martinez** (Ten Dijke/Chemical Signaling Laboratory, Department of Cell and Chemical Biology, Leiden University Medical Center and Oncode Institute, Leiden, The Netherlands; Department of Biomedical Engineering and Physics, Amsterdam University Medical Centers, Amsterdam, The Netherlands; Amsterdam Vesicle Center, Amsterdam University Medical Centers, Location AMC, University of Amsterdam, Amsterdam, The Netherlands), **Uta Erdbrügger** (University of Virginia Health System, Charlottesville, VA, USA), **Rezvan Esmaeili** (Genetics Department, Breast Cancer Research Center, Motamed Cancer Institute, Academic Center for Education Culture and Research, Tehran, Iran; Department of Experimental Radiation Oncology, The University of Texas MD Anderson Cancer Center, Houston, TX, USA), **Camille Ettelaie** (Biomedical Science, Hull‐York Medical School, Hull, UK), **András I Försönits** (Department of Genetics, Cell‐ and Immunobiology, Semmelweis University, Budapest, Hungary), **Muller Fabbri** (Center for Cancer and Immunology Research, Children's National Hospital, Washington, DC, USA; GW Cancer Center, George Washington University, Washington, DC, USA), **Marco Falasca** (Department of Medicine and Surgery, University of Parma, Parma, Italy; Metabolic Signalling Group, Curtin Medical School, Curtin Health and Innovation Research Institute, Curtin University, Bentley, Australia), **Juan M Falcon‐Perez** (Exosomes Laboratory, Center for Cooperative Research in Biosciences, Basque Research and Technology Alliance, Derio, Spain; Metabolomics Platform, Center for Cooperative Research in Biosciences, Basque Research and Technology Alliance, Derio, Spain; IKERBASQUE, Basque Foundation for Science, Bilbao, Spain), **Hongkuan Fan** (Department of Pathology and Laboratory Medicine, Medical University of South Carolina, Charleston, USA), **Farah Fatima** (Chalmers University of Technology, Gothenburg, Sweden), **Vroniqa Ku'ulei‐Lyn Faustino** (Department of Molecular Medicine, Beckman Research Institute, City of Hope Comprehensive Cancer Center, Duarte, CA, USA), **Alireza Fazeli** (Estonian University of Life Sciences, Tartu, Estonia; University of Tartu, Tartu, Estonia; University of Sheffield, Sheffield, UK), **María Fernández‐Rhodes** (Biomedical Research Institute of Lleida Dr. Pifarré Foundation (IRBLLEIDA), University Hospital Arnau de Vilanova (HUAV), Lleida, Spain; Department of Medical Basic Sciences, University of Lleida (UdL), Lleida, Spain), **Christopher Fernandez‐Prada** (Faculty of Veterinary Medicine, Université de Montréal, Montreal, Canada), **Mariola J Ferraro** (Department of Microbiology and Cell Science, University of Florida, Gainesville, FL, USA), **Joao N Ferreira** (Avatar Biotechnologies for Oral Health and Health Longevity Research Unit, Faculty of Dentistry, Chulalongkorn University, Bangkok, Thailand; Department of Research Affairs, Faculty of Dentistry, Chulalongkorn University, Bangkok, Thailand), **Rafaela F Ferreira** (Institute of Animal Science and Physiology, University of Bonn, Bonn, Germany), **Leandra K Figueroa‐Hall** (Laureate Institute for Brain Research, Tulsa, OK, USA; The University of Tulsa, Tulsa, OK, USA), **Aliosha I Figueroa‐Valdés** (IMPACT, Center of Interventional Medicine for Precision and Advanced Cellular Therapy, Santiago, Chile; Laboratory of Nano‐Regenerative Medicine, Centro de Investigación e Innovación Biomédica (CiiB), Universidad de los Andes, Santiago, Chile; Cells for Cells, Santiago, Chile), **Paolo V Fioretti** (University of Trento, Trento, Italy), **Sabine Flenady** (StemXo, Melbourne, Australia; Melbourne University, Melbourne, Australia; Central Queensland University, Norman Gardens, Australia), **Miguel Flores‐Bellver** (CellSight Ocular Stem Cell and Regeneration Program, Department of Ophthalmology, Sue Anschutz‐Rodgers Eye Center, University of Colorado Anschutz Medical Campus, Aurora, CO, USA), **Ellis K Fok** (School of Biomedical Sciences, Faculty of Medicine, The Chinese University of Hong Kong, Hong Kong SAR; Sichuan University‐The Chinese University of Hong Kong Joint Laboratory for Reproductive Medicine, West China Second University Hospital, Chengdu, China; School of Biomedical Sciences Core Laboratory, Shenzhen Research Institute, The Chinese University of Hong Kong, Shenzhen, China), **Pamali Fonseka** (Department of Biochemistry, La Trobe Institute for Molecular Science, La Trobe University, Melbourne, Australia), **Karen Forbes** (Leeds Institute of Cardiovascular and Metabolic Medicine, University of Leeds, Leeds, UK), **Verity J Ford** (Clinical Center, National Institutes of Health, Bethesda, MD, USA), **Cristina Fornaguera** (Grup d'Enginyeria de Materials (Gemat), Institut Químic de Sarrià (IQS), Universitat Ramon Llull (URL), Barcelona, Spain), **Dorian Forte** (Department of Medical and Surgical Sciences, Institute of Hematology “L. and A. Seràgnoli”, University of Bologna, Bologna, Italy), **Stefano Forte** (IOM Ricerca, Viagrande, Italy; Mediterranean Institute of Oncology, Viagrande, Italy), **Orazio Fortunato** (Fondazione IRCCS Istituto Nazionale dei Tumori, Milan, Italy), **Jeffrey L Franklin** (Department of Medicine, Gastroenterology, Vanderbilt University Medical Center, Nashville, TN, USA; Department of Cell and Developmental Biology, Vanderbilt University, Nashville, TN, USA; Epithelial Biology Center, Department of Medicine, Vanderbilt University Medical Center, Nashville, TN, USA), **Daniela Freitas** (i3S‐Institute for Research and Innovation in Health, University of Porto, Porto, Portugal; IPATIMUP‐Institute of Molecular Pathology and Immunology, University of Porto, Porto, Portugal), **Annie Frelet‐Barrand** (Institute FEMTO‐ST, Université de Franche‐Comté, CNRS, Besançon, France), **Qing‐Ling Fu** (Otorhinolaryngology Hospital, The First Affiliated Hospital, Sun Yat‐sen University, Guangzhou, China; Extracellular Vesicle Research and Clinical Translational Center, The First Affiliated Hospital, Sun Yat‐sen University, Guangzhou, China), **Yu Fujita** (The Jikei University Shool of Medicine, Tokyo, Japan), **Kathrin Gärtner** (Eximmium Biotechnologies GmbH, Munich, Germany), **André Görgens** (Department of Laboratory Medicine, Karolinska Institutet, Stockholm, Sweden; Department of Cellular Therapy and Allogeneic Stem Cell Transplantation (CAST), Karolinska University Hospital Huddinge and Karolinska Comprehensive Cancer Center, Stockholm, Sweden; Institute for Transfusion Medicine, University Hospital Essen, University of Duisburg‐Essen, Essen, Germany), **Áurea M Gabriel** (Institute of Hygiene and Tropical Medicine, NOVA University of Lisbon, Lisbon, Portugal; Institute of Biological Sciences, Federal University of Pará, Belém, Brazil), **Martina Gabrielli** (CNR Institute of Neuroscience, Vedano al Lambro, Italy), **Susanne Gabrielsson** (Division of Immunology and Allergy, Department of Medicine Solna, Karolinska Institutet, Stockholm, Sweden; Center for Molecular Medicine, Karolinska University Hospital, Stockholm, Sweden; Department of Clinical Immunology and Transfusion Medicine, Karolinska University Hospital, Stockholm, Sweden), **Alicia Galinsoga** (Department of Genetics, Cell‐ and Immunobiology, Semmelweis University, Budapest, Hungary), **Andrea Galisova** (Institute for Clinical and Experimental Medicine, Prague, Czech Republic), **Teena KJB Gamage** (Department of Obstetrics and Gynaecology, University of Auckland, New Zealand; Department of Physiology, Faculty of Medical and Health Sciences, The University of Auckland, Auckland, New Zealand), **Yingtang Gao** (Tianjin Key Laboratory of Extracorporeal Life Support for Critical Diseases, Tianjin Institute of Hepatobiliary Disease, Nankai University Affiliated the Third Center Hospital, Tianjin, China), **Marta Garcia‐Contreras ** (MGH/Harvard Medical School, Boston, MA, USA), **M Madhy Garcia Garcia** (University of California Irvine, Irvine, CA, USA), **Maria Noé Garcia** (Institute of Studies of the Humoral Immunity (IDEHU), National Council of Science and Technology (CONICET), University of Buenos Aires, Buenos Aires, Argentina), **Ernesto Gargiulo** (Department of Hematology, Rigshospitalet, Copenhagen University Hospital, Copenhagen, Denmark), **Hernán González‐King Garibotti** (Research and Early Development, Cardiovascular, Renal and Metabolism, BioPharmaceuticals R&D, AstraZeneca, Mölndal, Sweden), **Margaret M Mc Gee** (School of Biomolecular and Biomedical Science, University College Dublin, Dublin, Ireland; Conway Institute of Biomolecular and Biomedical Research, University College Dublin, Dublin, Ireland), **Géraldine C Genard** (Division of Biomedical Physics in Radiation Oncology, German Cancer Research Center (DKFZ), Heidelberg, Germany), **Fabiana Geraci** (Department of Biological, Chemical and Pharmaceutical Sciences and Technologies, University of Palermo, Palermo, Italy), **Jamal Ghanam** (Department of Pediatrics III, University Hospital Essen, Essen, Germany), **Subhadip Ghatak** (McGowan Center for Regenerative Medicine, Department of Surgery, University of Pittsburgh, Pittsburgh, PA, USA), **Mahlegha Ghavami** (Pathology Department, Dalhousie University, Halifax, Canada), **Raluca E Ghebosu** (Australian Institute for Bioengineering and Nanotechnology, The University of Queensland, Brisbane, Australia), **Yong Song Gho** (Pohang University of Science and Technology, Pohang, Republic of Korea), **Sayam Ghosal** (Department of Genetics, Cell‐ and Immunobiology, Semmelweis University, Budapest, Hungary; HCEMM‐SU Extracellular Vesicle Research Group, Semmelweis University, Budapest, Hungary), **Georgios Giamas** (Department of Biochemistry and Biomedicine, School of Life Sciences, University of Sussex, Brighton, UK), **Bernd Giebel** (Institute for Transfusion Medicine, University Hospital Essen, University of Duisburg‐Essen, Essen, Germany), **Caroline Gilbert** (Division of Infectious and Immune Diseases, CHU de Quebec Research Center, Quebec, Canada; Department of Microbiology, Infectious Disease and Immunology, Faculty of Medicine, Université Laval, Quebec, Canada), **Mario Gimona** (Paracelsus Medical University, Salzburg, Austria), **Henrique Girão** (Coimbra Institute for Clinical and Biomedical Research (iCBR), Faculty of Medicine, University of Coimbra, Coimbra, Portugal; Center for Innovative Biomedicine and Biotechnology, University of Coimbra, Coimbra, Portugal; Academic and Clinical Center of Coimbra, Coimbra, Portugal), **Ilaria Giusti** (Department of Life, Health and Environmental Sciences, University of L'Aquila, L'Aquila, Italy), **Evan A Gizzie** (Meso Scale Diagnostics, LLC., Rockville, MD, USA), **Sofija Glamočlija** (Institute for the Application of Nuclear Energy, Belgrade, Serbia), **Sarah E Glass** (Vanderbilt University Medical Center, Nashville, TN, USA), **Jessica Gobbo** (Department of Medical Oncology, Centre Georges‐François Leclerc, Dijon, France; INSERM UMR 1231, «Equipe labellisée» Ligue National contre le Cancer and Laboratoire d'Excellence LipSTIC, Dijon, France; University of Bourgogne, Dijon, France), **Deborah CI Goberdhan** (Nuffield Department of Women's and Reproductive Health, University of Oxford, Women's Centre, John Radcliffe Hospital, Oxford, UK), **Nihar Godbole** (Translational Extracellular Vesicles in Obstetrics and Gynae‐Oncology Group, University of Queensland Centre for Clinical Research, Faculty of Medicine, Royal Brisbane and Women's Hospital, The University of Queensland, Brisbane, Australia), **Jacky G Goetz** (Tumor Biomechanics, INSERM UMR_S1109, Strasbourg, France; University of Strasbourg, Strasbourg, France; Equipe Labellisée Ligue Contre le Cancer), **Olesia Gololobova** (Department of Molecular and Comparative Pathobiology, Johns Hopkins University School of Medicine, Baltimore, MD, USA; EV Core Facility “EXCEL”, Institute for Basic Biomedical Sciences, Johns Hopkins University School of Medicine, Baltimore, MD, USA), **Manuel Gomez‐Florit** (Health Research Institute of the Balearic Islands (IdISBa), Palma, Spain; Cell Therapy and Tissue Engineering Group, Research Institute on Health Sciences (IUNICS), University of the Balearic Islands, Palma, Spain), **Jenifer Pendiuk Goncalves** (Australian Institute for Bioengineering and Nanotechnology, The University of Queensland, Brisbane, Australia), **Cansu Gorgun** (School of Pharmacy and Biomolecular Sciences, Royal College of Surgeons in Ireland, Dublin, Ireland; Trinity Centre for Biomedical Engineering, Trinity Biomedical Sciences Institute, Trinity College, Dublin, Ireland; Advanced Materials and Bioengineering Research Centre, Trinity College Dublin and Royal College of Surgeons in Ireland, Dublin, Ireland), **Alessandro Gori** (Institute of Chemical Sciences and Technologies, National Research Council of Italy, Milan, Italy), **Sabina Gorska** (Hirszfeld Institute of Immunology and Experimental Therapy, Polish Academy of Sciences, Wroclaw, Poland), **Michael W Graner** (University of Colorado Anschutz Medical Campus, Aurora, CO, USA), **Georges E Grau** (Vascular Immunology Unit, School of Medical Sciences, The University of Sydney, Sydney, Australia; Sydney Nano Institute, The University of Sydney, Australia; Sydney Infectious Diseases Institute, The University of Sydney, Australia), **Laura Grech** (Faculty of Medicine and Surgery, University of Malta, Msida, Malta), **David W Greening** (Baker Heart & Diabetes Institute, Melbourne, Australia; La Trobe University, Melbourne, Australia; The University of Melbourne, Melbourne, Australia), **Rüdiger M Groß** (Institute of Molecular Virology, Ulm University Medical Center, Ulm, Germany), **Julia C Gross** (Institute of Molecular Medicine, Health and Medical University, Potsdam, Germany), **Jens Gruber** (Curexsys GmbH, Goettingen, Germany), **Alice Gualerzi** (IRCCS Fondazione Don Carlo Gnocchi Onlus, Milan, Italy), **Dominic Guanzon** (The University of Queensland, Brisbane, Australia), **Johann M Gudbergsson** (Department of Biomedicine, Aarhus University, Aarhus, Denmark), **Coralie L Guerin** (Cytometry Platform, Institut Curie, Paris, France; Extracellular Vesicles Platform, Institut Curie, Paris, France), **Flora Guerra** (Department of Biological and Environmental Sciences and Technologies, Università del Salento, Lecce, Italy), **Maria I Guillén** (Faculty of Health Sciences, Cardenal Herrera CEU University, Alfara del Patriarca, Valencia, Spain; Interuniversity Research Institute for Molecular Recognition and Technological Development (IDM), University of Valencia, Polytechnic University of Valencia, Valencia, Spain), **Vikramsingh Gujar** (Oklahoma State University Center for Health Sciences, Tulsa, OK, USA), **Wei Guo** (Department of Biology, University of Pennsylvania, Philadelphia, PA, USA), **Veer Bala Gupta** (Faculty of Heath, School of Medicine, Deakin University, Geelong, Australia), **Vivek Kumar Gupta** (Faculty of Medicine, Health and Human Sciences, Macquarie Medical School, Macquarie University, Sydney, Australia; School of Life Sciences, Pooja Bhagwat Memorial Mahajana Education Centre, Mysore, Karnataka, India), **Dakota Gustafson** (Department of Laboratory Medicine & Pathobiology, University of Toronto, Toronto, ON, Canada; Faculty of Health Sciences, Queen's University, Kingston, ON, Canada), **Edina Gyukity‐Sebestyén** (Laboratory of Microscopic Image Analysis and Machine Learning, Hungarian Research Network (HUN‐REN), Biological Research Centre, Szeged, Hungary; Department of Immunology, Albert Szent‐Györgyi Medical School, Faculty of Science and Informatics, University of Szeged, Szeged, Hungary), **Patrick Hölker** (University Hospital Cologne, Cologne, Germany), **Mangesh D Hade** (Nationwide Children's Hospital, Columbus, OH, USA; The Ohio State University, Columbus, OH, USA), **Daniel W Hagey** (Department of Laboratory Medicine, Karolinska Institutet, Stockholm, Sweden; Department of Cellular Therapy and Allogeneic Stem Cell Transplantation (CAST), Karolinska University Hospital Huddinge and Karolinska Comprehensive Cancer Center, Stockholm, Sweden), **Chungmin Han** (Wyss Institute for Biologically Inspired Engineering, Harvard University, Boston, MA, USA; Brigham and Women's Hospital, Boston, MA, USA; Harvard Medical School, Boston, MA, USA), **Pingping Han** (School of Dentistry, The University of Queensland, Brisbane, Australia), **Rikinari Hanayama** (WPI Nano Life Science Institute (NanoLSI), Kanazawa University, Kanazawa, Japan; Department of Immunology, Graduate School of Medical Sciences, Kanazawa University, Kanazawa, Japan), **Aase Handberg** (Department of Clinical Biochemistry, Aalborg University Hospital, Aalborg, Denmark; Department of Clinical Medicine, Aarhus University, Aarhus, Denmark), **Edveena Hanser** (Department of Biomedicine, University Hospital Basel, Basel, Switzerland; Department of Biomedicine, University of Basel, Basel, Switzerland), **Masako Harada** (Department of Biomedical Engineering, Michigan State University, East Lansing, MI, USA; Institute for Quantitative Health Science and Engineering (IQ), Department of Pharmacology & Toxicology, Michigan State University, East Lansing, MI, USA), **Maria Harmati** (Laboratory of Microscopic Image Analysis and Machine Learning, Hungarian Research Network (HUN‐REN), Biological Research Centre, Szeged, Hungary; Department of Immunology, University of Szeged, Szeged, Hungary), **Adrian L Harris** (Department of Oncology, University of Oxford, Oxford, UK), **Paul Harrison** (Institute of Inflammation and Ageing, University of Birmingham, Birmingham, UK), **Rane A Harrison** (Hemab Therapeutics, Cambridge, MA, USA), **Norman J Haughey** (Departments of Neurology and Psychiatry, Johns Hopkins University School of Medicine, Baltimore, MD, USA), **Paul A Haynes** (School of Natural Sciences, Macquarie University, Sydney, Australia), **Mei He** (Department of Pharmaceutics, College of Pharmacy, University of Florida, Gainesville, FL, USA; University of Florida Health Cancer Center, Gainesville, FL, USA), **Hargita Hegyesi** (Department of Genetics, Cell‐ and Immunobiology, Semmelweis University, Budapest, Hungary), **An Hendrix** (Laboratory of Experimental Cancer Research, Department of Human Structure and Repair, Ghent University, Ghent, Belgium; Cancer Research Institute Ghent, Ghent, Belgium), **Martijn JC van Herwijnen** (Department of Biomolecular Health Sciences, Faculty of Veterinary Medicine, Utrecht University, Utrecht, The Netherlands), **Andrew F Hill** (Institute for Health and Sport, Victoria University, Melbourne, Australia), **Colin L Hisey** (Department of Biomedical Engineering, The Ohio State University, Columbus, OH, USA; Department of Chemical and Biomolecular Engineering, The Ohio State University, Columbus, OH, USA; Northwestern University, Department of Biomedical Engineering, Evanston, IL, USA), **Fred H Hochberg** (Massachusetts General Hospital, Boston, MA, USA (retired); Scintillon Institute, San Diego, CA, USA; PIOMA, Sarasota, FL, USA), **Esther NM Nolte‐'t Hoen** (Department of Biomolecular Health Sciences, Faculty of Veterinary Medicine, Utrecht University, Utrecht, The Netherlands), **Marija Holcar** (Institute for Biochemistry and Molecular Genetics, Faculty of Medicine, University of Ljubljana, Ljubljana, Slovenia), **Beth Holder** (Imperial College London, London, UK), **Wolfgang Holnthoner** (Ludwig Boltzmann Institute for Traumatology, The Research Centre in Cooperation with AUVA, Vienna, Austria; Austrian Cluster for Tissue Regeneration, Vienna, Austria), **Harry Holthofer** (Finnish Institute of Molecular Medicine (FIMM), University of Helsinki, Helsinki, Finland), **D Craig Hooper** (Thomas Jefferson University, Philadelphia, PA, USA), **Nicole Noren Hooten** (National Institute on Aging, National Institutes of Health, Baltimore, MD, USA), **Elham Hosseini‐Beheshti** (Asbestos and Dust Diseases Research Institute, Sydney, Australia), **Baharak Hosseinkhani** (Laboratory of Angiogenesis and Vascular Metabolism, Center for Cancer Biology (CCB), VIB and Department of Oncology, Leuven Cancer Institute (LKI), KU Leuven, Leuven, Belgium), **Jane Howard** (UCD School of Medicine, College of Health, and Agricultural Sciences (CHAS), University College Dublin, Dublin, Ireland; UCD Conway Institute of Biomolecular and Biomedical Research, University College Dublin, Dublin, Ireland), **Kathryn L Howe** (University Health Network, Toronto, Canada; University of Toronto, Toronto, Canada; Peter Munk Cardiac Centre, Toronto, Canada), **Nicholas R Hoyle** (ClinBioConsulting FRG, Eschenlohe, Germany), **Jiri Hrdy** (Institute of Immunology and Microbiology, First Faculty of Medicine, Charles University and General University Hospital in Prague, Prague, Czech Republic), **Guoku Hu** (University of Nebraska Medical Center, Omaha, NE, USA), **Yiyao Huang** (Department of Molecular and Comparative Pathobiology, Johns Hopkins University School of Medicine, Baltimore, MD, USA; Department of Laboratory Medicine, Nanfang Hospital, Southern Medical University, Guangzhou, China), **Veronica Huber** (Translational Immunology Unit, Fondazione IRCCS Istituto Nazionale dei Tumori, Milan, Italy), **Samo Hudoklin** (Institute of Cell Biology, Faculty of Medicine, University of Ljubljana, Ljubljana, Slovenia), **Antonia Hufnagel** (Novo Nordisk Foundation Center for Basic Metabolic Research, Faculty of Health and Medical Sciences, University of Copenhagen, Copenhagen, Denmark), **Mark D Hulett** (Department of Biochemistry and Chemistry, La Trobe Institute for Molecular Science, La Trobe University, Melbourne, Australia), **Stuart Hunt** (The University of Sheffield, Sheffield, UK), **Vincent Hyenne** (INSERM UMR_S1109, Tumor Biomechanics, Strasbourg, France; University of Strasbourg, Strasbourg, France; CNRS SNC5055, Strasbourg, France), **Emilio Di Ianni** (Department of Neurology, Massachusetts General Hospital, Harvard Medical School, Boston, MA, USA), **Dalila Iannotta** (School of Chemical Engineering, The University of Queensland, Brisbane, Australia), **Ahmed GE Ibrahim** (Smidt Heart Institute, Cedars‐Sinai Medical Center, Los Angeles, CA, USA), **Sherif A Ibrahim** (AviceRNA, Konya, Turkey; Department of Pediatrics and Human Development, Michigan State University, East Lansing, MI, USA; Department of Histology and Cell Biology, Faculty of Medicine, Mansoura University, Mansoura, Egypt), **Seiko Ikezu** (Department of Neuroscience, Mayo Clinic Florida, Jacksonville, FL, USA), **Tsuneya Ikezu** (Department of Neuroscience, Mayo Clinic Florida, Jacksonville, FL, USA), **Hyungsoon Im** (Center for Systems Biology, Massachusetts General Hospital, Boston, MA, USA; Department of Radiology, Massachusetts General Hospital, Boston, MA, USA), **Jameel M Inal** (London Metropolitan University, School of Human Sciences, Cell Communication in Disease Pathology, London, UK; University of Hertfordshire, School of Life and Medical Sciences, Biosciences Research Group, Hatfield, UK), **Aleksandra Inic‐Kanada** (Institute of Specific Prophylaxis and Tropical Medicine, Center for Pathophysiology, Infectiology, and Immunology, Medical University of Vienna, Vienna, Austria), **Marit Inngjerdingen** (Department of Pharmacology, University of Oslo, Oslo, Norway), **Yasuo Inoshima** (Gifu University, Gifu, Japan), **Alexander R Ivanov** (Barnett Institute of Chemical and Biological Analysis, Department of Chemistry and Chemical Biology, Northeastern University, Boston, MA, USA), **Alena Ivanova** (Discovery Biology, Discovery Sciences, BioPharmaceuticals R&D, AstraZeneca, Mölndal, Sweden), **Elena Izquierdo** (Departamento de Ciencias Médicas Básicas, Instituto de Medicina Molecular Aplicada (IMMA) Nemesio Díez, Facultad de Medicina, Universidad San Pablo‐CEU, CEU Universities, Madrid, Spain), **Malene Møller Jørgensen** (Department of Clinical Immunology, Aalborg University Hospital, Aalborg, Denmark; Department of Clinical Medicine, Aalborg University, Aalborg, Denmark), **Hannah K Jackson** (Pain Centre Versus Arthritis, School of Life Sciences, Queen's Medical Centre, University of Nottingham, Nottingham, UK; Children's Brain Tumour Research Centre, Biodiscovery Institute, University of Nottingham, Nottingham, UK), **Soren Jacobsen** (COPEACT, Department of Rheumatology, Rigshospitalet, Copenhagen, Denmark; Department of Clinical Medicine, University of Copenhagen, Denmark), **Fernanda Jadue** (Universidad de Los Andes, Santiago, Chile; Universidad del Desarrollo, Santiago, Chile), **Naureen Javeed** (Mayo Clinic, Rochester, MN, USA), **Steven M Jay** (University of Maryland, College Park, MD, USA), **Muthuvel Jayachandran** (Mayo Clinic College of Medicine and Science, Rochester, MN, USA), **Migara K Jayasinghe** (Department of Pharmacology, Yong Loo Lin School of Medicine, National University of Singapore, Singapore), **Guido Jenster** (Erasmus Medical Center, Department of Urology, Rotterdam, The Netherlands), **Dennis K Jeppesen** (Department of Medicine, Vanderbilt University Medical Center, Nashville, TN, USA), **Carmen Jerónimo** (Cancer Biology and Epigenetics Group, Research Center, Portuguese Oncology Institute of Porto (IPO Porto), Porto, Portugal; Department of Pathology and Molecular Immunology, ICBAS‐ School of Medicine and Biomedical Sciences, University of Porto, Porto, Portugal), **Linglei Jiang** (CNBG‐Virogin Biotech (Shanghai) Co., Ltd., Shanghai, China), **Jing Jin** (Beijing Jizhongke Biotechnology Co., LTD, Beijing, China), **Kentaro Jingushi** (Laboratory of Molecular and Cellular Physiology, Graduate School of Pharmaceutical Sciences, Osaka University, Osaka, Japan), **Dong‐Gyu Jo** (School of Pharmacy, Sungkyunkwan University, Seoul, Republic of Korea; Institute of Quantum Biophysics, Sungkyunkwan University, Seoul, Republic of Korea; Biomedical Institute for Convergence, Sungkyunkwan University, Seoul, Republic of Korea), **Marianne S Joerger‐Messerli** (Department for BioMedical Research (DBMR), University of Bern, Bern, Switzerland; Department of Obstetrics and Feto‐maternal Medicine, University Women's Hospital, Inselspital, Bern University Hospital, Bern, Switzerland), **Jennifer C Jones** (Center for Cancer Research, National Cancer Institute, National Institutes of Health, Bethesda, MD, USA; Translational Nanobiology Section, Laboratory of Pathology, National Cancer Institute, National Institutes of Health, Bethesda, MD, USA), **Melissa K Jones** (IFAS, University of Florida, Gainesville, FL, USA), **Olivier G de Jong** (Department of Pharmaceutics, Utrecht Institute of Pharmaceutical Sciences (UIPS), Utrecht University, Utrecht, The Netherlands), **Tijana Jovanovic‐Talisman** (Department of Cancer Biology and Molecular Medicine, Beckman Research Institute, City of Hope Comprehensive Cancer Center, Duarte, CA, USA), **Anthony W Ferrante, Jr** (Naomi Berrie Diabetes Center, Columbia University, New York, NY, USA), **Leon G Coleman Jr** (Bowles Center for Alcohol Studies, Department of Pharmacology, University of North Carolina at Chapel Hill School of Medicine, Chapel Hill, NC, USA), **David Juncker** (McGill University, Montreal, Canada), **Stephanie Jung** (Institute of Cardiovascular Immunology, University Hospital Bonn, University of Bonn, Bonn, Germany), **Benjamin Jurek** (Max‐Planck Insitute for Psychiatry, Munich, Germany), **Marcin Jurga** (EXO Biologics SA, Liège, Belgium), **Verline Justilien** (Department of Cancer Biology, Mayo Clinic, Jacksonville, FL, USA), **Mehdi Kabani** (CNRS, CEA, Laboratoire des Maladies Neurodégénératives, Université Paris‐Saclay, Fontenay‐aux‐Roses, France), **Raghu Kalluri** (The University of Texas MD Anderson Cancer Center, Houston, TX, USA ), **Masood Kamali‐Moghaddam** (Department of Immunology, Genetics and Pathology, Science for Life Laboratory, Uppsala University, Uppsala, Sweden), **Masamitsu Kanada** (Institute for Quantitative Health Science and Engineering (IQ), Department of Pharmacology & Toxicology, Michigan State University, East Lansing, MI, USA), **Taeyoung Kang** (Department of Biochemistry, La Trobe Institute for Molecular Science, La Trobe University, Melbourne, Australia), **Shin‐ichi Kano** (University of Alabama at Birmingham, Birmingham, AL, USA), **Maria Kaparakis‐Liaskos** (Department of Microbiology, Anatomy, Physiology and Pharmacology, La Trobe University, Melbourne, Australia), **Elżbieta Karnas** (Department of Cell Biology, Faculty of Biochemistry, Biophysics and Biotechnology, Jagiellonian University, Krakow, Poland), **Antoine Karoichan** (Faculty of Dental Medicine and Oral Health Sciences, McGill University, Montreal, Canada), **Fatah Kashanchi** (Laboratory of Molecular Virology, George Mason University, Manassas, VA, USA), **Sara Assar Kashani** (Centre for Motor Neuron Disease Research, Macquarie Medical School, Macquarie University, Sydney, Australia), **Namita N Kashyap** (Centre for Molecular Neurosciences, Kasturba Medical College Manipal, Manipal Academy of Higher Education, Manipal, India), **Miroslava Katsur** (The Hatter Cardiovascular Institute, University College London, London, UK), **Silvio Kau‐Strebinger** (Department of Pathobiology, University of Veterinary Medicine Vienna, Vienna, Austria), **Amy C Kauffman** (Corning Life Sciences, Kennebunk, ME, USA), **Sukhbir Kaur** (Laboratory of Pathology, National Cancer Institute, National Institutes of Health, Bethesda, MD, USA), **Oksana Kehoe** (Centre for Regenerative Medicine Research, School of Medicine, Keele University, Keele, UK; Robert Jones and Agnes Hunt Orthopaedic Hospital, Oswestry, UK), **Richard JR Kelwick** (Imperial College London, London, UK), **Amirmohammad Nasiri Kenari** (Research Centre for Advanced Science and Technology, The University of Tokyo, Tokyo, Japan), **Brachyahu M Kestecher** (Department of Genetics, Cell‐ and Immunobiology, Semmelweis University, Budapest, Hungary; HCEMM‐SU Extracellular Vesicle Research Group, Semmelweis University, Budapest, Hungary; HUN‐REN‐SU Translational Extracellular Vesicle Research Group, Semmelweis University, Budapest, Hungary), **Tom G Keulers** (Department of Radiotherapy, GROW‐School for Oncology and Reproduction, Maastricht University Medical Centre+, Maastricht, The Netherlands), **Kendall R Van Keuren‐Jensen** (Neurogenomics, TGen, Phoenix, AZ, USA), **Kasra Khalaj** (The Hospital for Sick Children, Toronto, Canada; University of Toronto, Toronto, Canada), **Delaram Khamari** (Department of Genetics, Cell‐ and Immunobiology, Semmelweis University, Budapest, Hungary), **Ramin Khanabdali** (Inoviq Limited, Notting Hill, Australia), **Elena Khomyakova** (Exosome Analytics, Evry, France), **Amanda Khoo** (Department of Medical Biophysics, University of Toronto, Toronto, Canada; Princess Margaret Cancer Centre, University Health Network, Toronto, Canada), **Daniel H Kim** (University of California, Santa Cruz, Santa Cruz, CA, USA; Canary Center at Stanford for Cancer Early Detection, Palo Alto, USA; Stanford RNA Medicine Program, Palo Alto, USA), **Dongin Kim** (College of Pharmacy, Oklahoma University Health Sciences Center, Oklahoma City, OK, USA), **Han Sang Kim** (Division of Medical Oncology, Department of Internal Medicine, Graduate School of Medical Science, Brain Korea 21 Project, Yonsei University College of Medicine, Seoul, Korea), **In‐San Kim** (Chemical and Biological Integrative Research Center, Biomedical Research Division, Korea Institute of Science and Technology (KIST), Seoul, Republic of Korea; KU‐KIST Graduate School of Converging Science and Technology, Korea University, Seoul, Republic of Korea), **Soo Kim** (Brexogen Inc., Seoul, Republic of Korea), **Yohan Kim** (Nathan S. Kline Institute for Psychiatric Research, Orangeburg, NY, USA; Department of Psychiatry, New York University School of Medicine, New York, NY, USA), **Peter E Kima** (Microbiology and Cell Science, University of Florida, Gainesville, FL, USA), **Thomas Kislinger** (Princess Margaret Cancer Centre, University Health Network, Toronto, ON, Canada; University of Toronto, Department of Medical Biophysics, Toronto, Canada), **Mikael Klingeborn** (McLaughlin Research Institute, Great Falls, MT, USA), **Rob Knight** (Cellese Inc., Irvine, CA, USA), **Hiroaki Komuro** (Smidt Heart Institute, Cedars‐Sinai Medical Center, Los Angeles, CA, USA; Department of Cardiovascular Medicine, Tokyo Medical and Dental University, Tokyo, Japan), **Anna Koncz** (Department of Genetics, Cell‐ and Immunobiology, Semmelweis University, Budapest, Hungary), **Timothea Konstantinou** (Cardiff University, Cardiff, UK), **Luke van der Koog** (Department of Molecular Pharmacology, Groningen Research Institute of Pharmacy, Faculty of Science and Engineering, University of Groningen, Groningen, The Netherlands; GRIAC, Groningen Research Institute for Asthma and COPD, University Medical Center Groningen, Groningen, The Netherlands), **Sander AA Kooijmans** (CDL Research, University Medical Center Utrecht, Utrecht, The Netherlands), **Miroslaw T Kornek** (Department of Internal Medicine I, University Hospital Bonn of the Rheinische Friedrich‐Wilhelms‐University, Bonn, Germany; Department of General, Visceral and Thoracic Surgery, German Armed Forces Central Hospital, Koblenz, Germany), **Maja Kosanović** (Institute for the Application of Nuclear Energy, INEP, University of Belgrade, Belgrade, Serbia), **Enis Kostallari** (Division of Gastroenterology and Hepatology, Mayo Clinic, Rochester, MN, USA), **Tiana F Koukoulis** (The Florey Institute of Neuroscience and Mental Health, The University of Melbourne, Parkville, Australia), **Stella Kourembanas** (Division of Newborn Medicine, Boston Children's Hospital, Boston, MA, USA; Harvard Medical School, Boston, MA, USA), **Eva‐Maria Krämer‐Albers** (Institute for Developmental Biology and Neurobiology, Johannes Gutenberg University Mainz, Mainz, Germany), **Veronika Kralj‐Iglic** (Laboratory of Clinical Biophysics, Faculty of Health Sciences, University of Ljubljana, Ljubljana, Slovenia), **Susanne Krasemann** (Institute of Neuropathology, University Medical Center Hamburg‐Eppendorf, Hamburg, Germany; Core Facility for Pathology, University Medical Center Hamburg‐Eppendorf, Hamburg, Germany), **Anna D Krasnodembskaya** (Queen's University Belfast, Belfast, UK), **Natalia J Krawczynska** (Beckman Institute for Advanced Science and Technology, University of Illinois at Urbana‐Champaign, Urbana‐Champaign, IL, USA; Department of Molecular and Integrative Physiology, University of Illinois at Urbana‐Champaign, IL, USA), **Mateja E Kreft** (Institute of Cell Biology, Faculty of Medicine, University of Ljubljana, Ljubljana, Slovenia), **Nicole A Kruh‐Garcia** (Bio‐pharmaceutical Manufacturing and Academic Resource Center (BioMARC), Infectious Disease Research Center, Colorado State University, Fort Collins, CO, USA), **Meta J Kuehn** (Duke University Medical Center, Durham, NC, USA), **Marije E Kuipers** (Department of Biomolecular Health Sciences, Faculty of Veterinary Medicine, Utrecht University, Utrecht, The Netherlands; Department of Parasitology, Leiden University Medical Center, Leiden, The Netherlands), **Konxhe Kulaj** (Molecular Cell Biology, Institute for Theoretical Medicine, Medical Faculty, University of Augsburg, Augsburg, Germany; Institute for Diabetes and Obesity, Helmholtz Zentrum München, Neuherberg, Germany; German Center for Diabetes Research (DZD), Neuherberg, Germany), **Julia Kuligowski** (Health Research Institute La Fe, Valencia, Spain), **Yumi Kumagai** (Juntendo University, Tokyo, Japan), **Ashish Kumar** (Department of Cancer Biology, Wake Forest School of Medicine, Winston‐Salem, NC, USA), **Saroj Kumar** (Department of Biophysics, All India Institute of Medical Sciences, New Delhi, India; Department of Health Science, Lulea University of Technology, Lulea, Sweden), **Sharad Kumar** (Centre for Cancer Biology, University of South Australia, Adelaide, Australia; Adelaide Medical School, Faculty of Health and Medical Sciences, The University of Adelaide, Adelaide, Australia), **Meena Kumari** (Kansas State University, Manhattan, KS, USA), **Gabrielis Kundrotas** (EXO Biologics SA, Liège, Belgium), **Igor V Kurochkin** (Central Research Laboratories, Sysmex Co., Kobe, Japan), **Masahiko Kuroda** (Department of Molecular Pathology, Tokyo Medical University, Tokyo, Japan), **Marzena Kurzawa‐Akanbi** (Biosciences Institute, Faculty of Medical Sciences, Newcastle University, Newcastle upon Tyne, UK ), **Sasha J Kweskin** (Bayer AG, St. Louis, MO, USA), **Diego Kyburz** (Department of Biomedicine, University of Basel, Basel, Switzerland; Department of Rheumatology, University Hospital Basel, Basel, Switzerland), **Elisa Lázaro‐Ibáñez** (Advanced Drug Delivery, Pharmaceutical Sciences, Biopharmaceutics R&D, AstraZeneca, Gothenburg, Sweden), **Cecilia Lässer** (Krefting Research Centre, Department of Internal Medicine and Clinical Nutrition, Institute of Medicine at Sahlgrenska Academy, University of Gothenburg, Gothenburg, Sweden), **Jan Lötvall** (Krefting Research Centre, Institute of Medicine at Sahlgrenska Academy, University of Gothenburg, Gothenburg, Sweden), **Ákos M Lőrincz** (Department of Physiology, Semmelweis University, Budapest, Hungary; Second Department of Internal Medicine, Szent György Hospital, Székesfehérvár, Hungary), **Andrew Lai** (Translational Extracellular Vesicles in Obstetrics and Gynae‐Oncology Group, University of Queensland Centre for Clinical Research, Faculty of Medicine, Royal Brisbane and Women's Hospital, The University of Queensland, Brisbane, Australia), **Charles P Lai** (Institute of Atomic and Molecular Sciences, Academia Sinica, Taipei, Taiwan; Chemical Biology and Molecular Biophysics Program, TIGP, Academia Sinica, Taipei, Taiwan; Genome and Systems Biology Degree Program, National Taiwan University, Taipei, Taiwan), **Saara Laitinen** (Finnish Red Cross Blood Service, Helsinki, Finland), **Solange Landreville** (Université Laval, Quebec, Canada; Centre de Recherche du Centre Hospitalier Universitaire de Québec, Université Laval, Quebec, Canada), **Sigrun Lange** (Pathobiology and Extracellular Vesicles Research Group, School of Life Sciences, University of Westminster, London, UK), **Scott M Langevin** (University of Vermont Larner College of Medicine, Burlington, VT, USA; University of Vermont Cancer Center, Burlington, VT, USA), **Marc‐André Langlois** (Department of Biochemistry, Microbiology & Immunology, Faculty of Medicine, University of Ottawa, Ottawa, Canada), **Lucia R Languino** (Thomas Jefferson University, Philadelphia, PA, USA), **Joanne Lannigan** (Flow Cytometry Support Services, LLC, Alexandria, VA, USA), **Daniel S Lark** (Colorado State University, Fort Collins, CO, USA), **Adriana T Larregina** (University of Pittsburgh School of Medicine, Pittsburgh, PA, USA), **Louise C Laurent** (Division of Maternal Fetal Medicine, Department of Obstetrics, Gynecology, and Reproductive Sciences, University of California, San Diego, San Diego, CA, USA), **David Laurin** (Research Center INSERM U1209/CNRS UMR 5309, Etablissement Français du Sang, Université Grenoble Alpes, Grenoble, France), **Gregory Lavieu** (INSERM U1316, CNRS UMR7057, Université Paris Cité, Paris, France), **Charlotte Lawson** (Department of Comparative Biomedical Sciences, Royal Veterinary College, London, UK; School of Pharmacy and Biomedical Sciences, University of Central Lancashire, Preston, UK), **Soazig Le Lay** (CNRS, INSERM, l'institut du thorax, Nantes University, Nantes, France; Univ Angers, SFR ICAT, Angers, France), **Kevin Leandro** (Center for Neuroscience and Cell Biology, University of Coimbra, Coimbra, Portugal; Center for Innovative Biomedicine and Biotechnology, University of Coimbra, Coimbra, Portugal; Faculty of Pharmacy, University of Coimbra, Coimbra, Portugal), **Aurélie Ledreux** (Department of Neurosurgery, School of Medicine, University of Colorado Anschutz Medical Campus, Aurora, CO, USA), **Changjin Lee** (SL Bigen, Inc., Incheon, Republic of Korea), **Dong‐Sup Lee** (Seoul National University College of Medicine, Seoul, Republic of Korea), **Hakho Lee** (Center for Systems Biology, Massachusetts General Hospital, Boston, MA, USA; Department of Radiology, Massachusetts General Hospital, Harvard Medical School, Boston, MA, USA), **Heon‐Jin Lee** (Department of Microbiology and Immunology, School of Dentistry, Kyungpook National University, Daegu, Republic of Korea), **Sun Young Lee** (Department of Ophthalmology and Physiology & Neuroscience , University of Southern California, Los Angeles, CA, USA), **Tae Ryong Lee** (SL Bigen, Inc., Incheon, Republic of Korea), **Wai‐Leng Lee** (School of Science, Monash University Malaysia, Bandar Sunway, Malaysia), **Iliya Lefterov** (University of Pittsburgh, Pittsburgh, PA, USA; Medical University Varna, Varna, Bulgaria), **Xinhua Lei** (Beijing Jizhongke Biotechnology Co., LTD, Beijing, China), **Janne Leivo** (Department of Life Technologies, University of Turku, Turku, Finland; InFLAMES Research Flagship, University of Turku, Turku, Finland), **Quentin Lemaire** (CNRS, UMR 8576 ‐ UGSF—Unité de Glycobiologie Structurale et Fonctionnelle, University of Lille, Lille, France), **Adriana F Paes Leme** (Laboratório Nacional de Biociências—LNBio, Centro Nacional de Pesquisa em Energia e Materiais—CNPEM, Campinas, Brazil), **Stanley M Lemon** (Lineberger Comprehensive Cancer Center and Departments of Medicine and Microbiology & Immunology, The University of North Carolina at Chapel Hill, Chapel Hill, NC, USA), **Metka Lenassi** (University of Ljubljana, Faculty of Medicine, Ljubljana, Slovenia), **Kathleen M. Lennon** (Department of Molecular Medicine, Beckman Research Institute, City of Hope Comprehensive Cancer Center, Duarte, CA, USA), **Stephen Lenzini** (RoosterBio, Frederick, MD, USA), **Jonathan Leor** (Neufeld Cardiac Research Institute, School of Medicine, Tel Aviv University, Tel Aviv, Israel; Tamman Cardiovascular Research Institute, Heart Center, Sheba Medical Center, Ramat Gan, Israel), **Efrat levy** (New York University Langone Health, New York, NY, USA; Nathan S. Kline Institute for Psychiatric Research, Orangeburg, NY, USA), **Bo Li** (Department of Laboratory Medicine, Nanfang Hospital, Southern Medical University, Guangzhou, China; Guangdong Engineering and Technology Research Center for Rapid Diagnostic Biosensors, Nanfang Hospital, Southern Medical University, Guangzhou, China), **Guoping Li** (Cardiovascular Research Center, Massachusetts General Hospital, Boston, MA, USA; Harvard Medical School, Boston, MA, USA), **Jiao Jiao Li** (University of Technology Sydney, Sydney, Australia; Kolling Institute, Sydney, Australia), **Qiubai Li** (Department of Rheumatology and Immunology, Union Hospital, Tongji Medical College, Huazhong University of Science and Technology, Wuhan, China; Hubei Engineering Research Center for Application of Extracelluar Vesicle, Hubei University of Science and Technology, Xianning, China), **Xinlei Li** (The Abigail Wexner Research Institute, Nationwide Children's Hospital, Columbus, OH, USA), **Xiuming Liang** (Biomolecular Medicine, Clinical Research Center, Department of Laboratory Medicine, Karolinska Institutet, Stockholm, Sweden; Cancer Research Laboratory, Shandong University‐Karolinska Institutet Collaborative Laboratory, School of Basic Medical Science, Shandong University, Jinan, China), **Rebecca Lim** (Hudson Institute, Melbourne, Australia; Monash University, Melbourne, Australia), **Sai Kiang Lim** (Institute of Molecular and Cell Biology (IMCB), Agency for Science, Technology and Research (A*STAR), Singapore; Paracrine Therapeutics Pte. Ltd., Singapore; Department of Surgery, YLL School of Medicine, National University Singapore, Singapore), **Tania Limongi** (Department of Applied Science and Technology, Politecnico di Torino, Turin, Italy), **Aija Linē** (Latvian Biomedical Research and Study Centre, Riga, Latvia), **Paula Pincela Lins** (Hasselt University, Faculty of Medicine and Life Sciences, Biomedical Research Institute, Diepenbeek, Belgium; Health Department, Flemish Institute for Technological Research (VITO), Mol, Belgium), **Lien Lippens** (Laboratory of Experimental Cancer Research, Department of Human Structure and Repair, Ghent University, Ghent, Belgium; Cancer Research Institute Ghent, Ghent, Belgium), **Guanshu Liu** (Department of Radiology, Johns Hopkins University School of Medicine, Baltimore, MD, USA; FM Kirby Center, Kennedy Krieger Institute, Baltimore, MD, USA), **Alicia Llorente** (Department of Molecular Cell Biology, Institute for Cancer Research, Oslo University Hospital, Oslo, Norway; Department for Mechanical, Electrical and Chemical Engineering, Oslo Metropolitan University, Oslo, Norway; Centre for Cancer Cell Reprogramming, University of Oslo, Oslo, Norway), **Modeline N Longjohn** (Faculty of Medicine, Memorial University of Newfoundland and Labrador, St. John's, NL, Canada), **Fons AJ van de Loo** (Experimental Rheumatology, Radboud University Medical Center, Nijmegen, The Netherlands), **Magdalena J Lorenowicz** (Biomedical Primate Research Centre, Rijswijk, The Netherlands), **Aurelio Lorico** (Touro University Nevada College of Medicine, Henderson, NV, USA), **Olivier Loudig** (Center for Discovery and Innovation, Hackensack Meridian Health, Nutley, NJ, USA), **Xavier Loyer** (PARCC, INSERM, Université de Paris, Paris, France), **Estefanía Lozano‐Andrés** (Department of Biomolecular Health Sciences, Faculty of Veterinary Medicine, Utrecht University, Utrecht, The Netherlands), **Biao Lu** (Department of Bioengineering, School of Engineering, Santa Clara University, Santa Clara, CA, USA), **Quan Lu** (Department of Environmental Health, Harvard T.H. Chan School of Public Health, Boston, MA, USA), **Quentin Lubart** (Abbelight, Cachan, France), **Fabrice Lucien** (Department of Urology, Mayo Clinic, Rochester, MN, USA; Department of Immunology, Mayo Clinic, Rochester, MN, USA), **Taral R Lunavat** (Department of Biomedicine, University of Bergen, Bergen, Norway), **Ludwig Ermann Lundberg** (Swedish University of Agricultural Sciences, Uppsala, Sweden; BioGaia AB, Stockholm, Sweden), **David J Lundy** (College of Biomedical Engineering, Taipei Medical University, Taipei, Taiwan; Center for Cell Therapy, Taipei Medical University Hospital, Taipei, Taiwan), **Jens C Luoto** (Åbo Akademi University, Turku, Finland; Turku Bioscience, Turku, Finland), **David C Lyden** (Departments of Pediatrics and Cell and Developmental Biology, Meyer Cancer Center, Weill Cornell Medicine, New York, NY, USA), **Andreas Möller** (Chinese University of Hong Kong, Hong Kong, Hong Kong SAR; QIMR Berghofer Medical Research Institute, Brisbane, Australia), **Janis A Müller** (Institute of Virology, Philipps University Marburg, Marburg, Germany), **Daniel J MacPhee** (Department of Veterinary Biomedical Sciences, Western College of Veterinary Medicine, Saskatoon, Canada; University of Saskatchewan, Saskatoon, Canada), **Adam L. Maddox** (Department of Molecular Medicine, Beckman Research Institute, City of Hope Comprehensive Cancer Center, Duarte, CA, USA), **Elise Madec** (MSC‐med, Paris, France; EverZom, Paris, France), **Setty M Magaña** (The Abigail Wexner Research Institute, Nationwide Children's Hospital, Columbus, OH, USA), **Vasiliki Mahairaki** (Department of Genetic Medicine, Johns Hopkins University School of Medicine, Baltimore, MD, USA; The Richman Family Precision Medicine Center of Excellence in Alzheimer's Disease, Johns Hopkins University School of Medicine, Baltimore, MD, USA), **Mỹ G Mahoney** (Thomas Jefferson University, Philadelphia, PA, USA), **Harmeet Malhi** (Mayo Clinic, Rochester, MN, USA), **Cécile E Malnou** (Institut Toulousain des Maladies Infectieuses et Inflammatoires, INSERM, CNRS, UPS, Université de Toulouse, Toulouse, France), **Doste R Mamand** (Biomolecular and Cellular Medicine, Clinical Research Center, Department of Laboratory Medicine, Karolinska Institutet, Huddinge, Sweden), **Kenny Man** (Department of Oral and Maxillofacial Surgery & Special Dental Care, University Medical Center Utrecht, Utrecht, The Netherlands; UMC Utrecht Regenerative Medicine Center, Circulatory Health Research Center, University Medical Center Utrecht, Utrecht, The Netherlands), **Mauro Manno** (National Research Council of Italy, Institute of Biophysics, Palermo, Italy), **Pierre‐Yves Mantel** (Christine Kühne—Center for Allergy Research and Education, Davos, Switzerland; Department of Oncology, Microbiology, and Immunology, University of Fribourg, Fribourg, Switzerland), **Tecla Marangon** (Biomedical Engineering, College of Science and Engineering, University of Galway, Galway, Ireland), **Eduardo Marbán** (Smidt Heart Institute, Cedars‐Sinai Medical Center, Los Angeles, CA ,USA), **Antonio Marcilla** (Área de Parasitología, Dept. Farmacia y Tecnología Farmacéutica y Parasitología, F. Farmàcia, Universitat de València, Valencia, Spain; Joint Unit on Endocrinology, Nutrition and Clinical Dietetics, IIS La Fe‐Universitat de València, Valencia, Spain), **Krishna P Maremanda** (Department of Biochemistry and Biophysics, Texas A&M University, College Station, TX, USA), **Leonid Margolis** (National Institutes of Health, Bethesda, MD, USA (retired)), **Luis Mariñas‐Pardo** (Universidad Internacional de Valencia, Valencia, Spain; Instituto de Salud Carlos III, Majadahonda, Spain), **Ivica Marić** (Faculty of Medicine, University of Ljubljana, Ljubljana, Slovenia), **Silvia Sánchez Martín** (Institut d'Investigació Sanitària Pere Virgili, Tarragona, España), **Eduardo Martínez‐Martínez** (Instituto Nacional de Medicina Genómica, Mexico City, Mexico), **Catherine Martel** (Faculty of Medicine, Université de Montréal, Montreal, Canada; Montreal Heart Institute, Montreal, Canada), **Elena S Martens‐Uzunova** (Erasmus MC Cancer Institute, University Medical Center Rotterdam, Department of Urology, Rotterdam, The Netherlands), **Pilar Martin‐Duque** (Instituto de Salud Carlos III, Majadahonda, Spain; IIS Aragón, Zaragoza, Spain), **Lorena Martin‐Jaular** (Institut Curie, INSERM U932, PSL University, Paris, France; CurieCoreTech Extracellular Vesicles, Institut Curie, Paris, France), **Paola A Martinez‐Murillo** (CK‐CARE, Davos, Switzerland), **Sarai Martinez‐Pacheco** (School of Pharmacy and Pharmaceutical Sciences, Panoz Institute, Trinity College Dublin, Dublin, Ireland; Trinity Biomedical Sciences Institute, Trinity College Dublin, Dublin, Ireland; Trinity St. James's Cancer Institute, Trinity College Dublin, Dublin, Ireland), **Tania Martins‐Marques** (Coimbra Institute for Clinical and Biomedical Research (iCBR), Faculty of Medicine, University of Coimbra, Coimbra, Portugal; Center for Innovative Biomedicine and Biotechnology, University of Coimbra, Coimbra, Portugal), **Benjamin Mary** (Tumor Biomechanics, INSERM UMR_S1109, Centre de Recherche en Biomédecine de Strasbourg, Strasbourg, France), **Akbar L Marzan** (La Trobe Institute for Molecular Science, La Trobe University, Melbourne, Australia), **Andreu Matamoros‐Angles** (Institute of Neuropathology, University Medical Center Hamburg‐Eppendorf, Hamburg, Germany), **Suresh Mathivanan** (La Trobe Institute for Molecular Science, La Trobe University, Melbourne, Australia), **Juntaro Matsuzaki** (Division of Pharmacotherapeutics, Keio University Faculty of Pharmacy, Tokyo, Japan), **Maria D Mayan** (CellCOM Research Group, Instituto de Investigación Biomédica de A Coruña (INIBIC), University Hospital Complex A Coruña, Coruña, Spain; Servizo Galego de Saúde (SERGAS), Galicia, Spain), **Carla Mazzeo** (Boston University, Boston, MA, USA), **Mariama Mbengue** (Cardiff University, Cardiff, UK), **Mark J McCann** (The New Zealand Institute for Plant and Food Research Limited, Palmerston North, New Zealand; Te Ohu Rangahau Kai, AgResearch Limited, Palmerston North, New Zealand), **Luke C McIlvenna** (Blizard Institute, Barts and the London School of Medicine and Dentistry, Queen Mary University of London, London, UK), **Mark J McVey** (Department of Anesthesia and Pain Medicine Hospital for Sick Children, Toronto, ON, Canada; Anesthesiology and Pain Medicine, University of Toronto, Toronto, ON, Canada; Department of Physics, Toronto Metropolitan University, Toronto, ON, Canada), **Nicole Meisner‐Kober** (Department of Biosciences and Medical Biology, Paris‐Lodron University Salzburg, Salzburg, Austria), **Maiken Mellergaard** (Department of Clinical Biochemistry, Aalborg University Hospital, Aalborg, Denmark; Department of Clinical Medicine, Aalborg University, Aalborg, Denmark), **Giorgia Melli** (Neurocenter of Southern Switzerland, Ente Ospedaliero Cantonale, Lugano, Switzerland; Laboratories for Translational Research, Ente Ospedaliero Cantonale, Bellinzona, Switzerland; Faculty of Biomedical Sciences, Università della Svizzera Italiana, Lugano, Switzerland), **Kerstin Menck** (Department of Medicine A, University of Muenster, Muenster, Germany), **Nico G Menjivar** (Colorado State University, Fort Collins, CO, USA), **Ramkumar Menon** (Department of Obstetrics and Gynecology, The University of Texas Medical Branch at Galveston, Galveston, TX, USA), **Kyle I Mentkowski** (Massachusetts General Hospital, Boston, MA, USA), Flemish Institute for Technological Research (VITO), Mol, Belgium (University of Antwerp, Antwerp, Belgium), **John J Miklavcic** (Chapman University, Orange, CA, USA), **Andras G Miklosi** (ONI (Oxford Nanoimaging Ltd), Oxford, UK), **Bojana Milutinovic** (MD Anderson Cancer Center, University of Texas, Houston, TX, USA), **Valentina R Minciacchi** (Center for Thrombosis and Hemostasis, Johannes Gutenberg University Medical Center, Mainz, Germany), **Mehdi Mirzaei** (Macquarie Medical School, Macquarie University, Sydney, Australia), **Shalini Mishra** (Wake Forest School of Medicine, Winston‐Salem, NC, USA), **Megan I Mitchell** (Center for Discovery and Innovation, Hackensack Meridian Health, Nutley, NJ, USA), **Rachel R Mizenko** (Department of Biomedical Engineering, University of California, Davis, Davis, CA, USA), **Danilo Mladenović** (HansaBioMed Life Sciences, Tallinn, Estonia; Tallinn University, Tallinn, Estonia), **Eqbal Mohamadi** (NIGEB, Tehran, Iran; KNT, Javanrud, Iran), **Sujata Mohanty** (Stem Cell Facility, All India Institute of Medical Sciences, New Delhi, India), **Fatemeh Momen‐Heravi** (Cancer Biology and Immunology Laboratory, Columbia University Irving Medical Center, New York, NY, USA; Herbert Irving Comprehensive Cancer Center, Columbia University Irving Medical Center, New York, NY, USA; College of Dental Medicine, Columbia University, New YorkCity, NY, USA), **Sujan K Mondal** (Michigan State University, East Lansing, MI, USA), **Marta Monguió‐Tortajada** (Department of Immunobiology, University of Lausanne, Epalinges, Switzerland), **Jisook Moon** (College of Life Science, Department of Biotechnology, CHA University, Seoul, Republic of Korea), **Mattia I Morandi** (Institute of Organic Chemistry and Biochemistry of the Czech Academy of Science, Prague, Czech Republic), **Violaine Moreau** (INSERM, BRIC, U1312, University of Bordeaux, Bordeaux, France), **Lissette Retana Moreira** (Departamento de Parasitología, Facultad de Microbiología, Universidad de Costa Rica, San José, Costa Rica; Centro de Investigación en Enfermedades Tropicales (CIET), Universidad de Costa Rica, San José, Costa Rica), **Adrian E Morelli** (Thomas E Starzl Transplantation Institute, Department of Surgery, University of Pittsburgh, Pittsburgh, PA, USA), **Marcelo A Mori** (Universidade Estadual de Campinas (UNICAMP), Campinas, Brazil), **Masahiro Morimoto** (Department of Oral Diagnosis and Medicine, Hokkaido University Faculty of Dental Medicine, Sapporo, Japan), **Mathilde Mosser** (Oniris VetAgroBio Nantes, INRAE, IECM, Nantes, France), **Thabiso E Motaung** (Forestry and Agricultural Biotechnology Institute, University of Pretoria, Pretoria, South Africa), **Etienne Moussay** (Tumor Stroma Interactions, Department of Cancer Research, Luxembourg Institute of Health, Luxembourg, Luxembourg), **Vera Mugoni** (Department of Cellular, Computational and Integrative Biology (CIBIO), University of Trento, Trento, Italy), **Francois Mullier** (Université catholique de Louvain, CHU UCL Namur, Namur Thrombosis and Hemostasis Center (NTHC), Namur Research Institute for Life Sciences (NARILIS), Hematology Laboratory, Yvoir, Belgium; Institut de Recherche Expérimentale et Clinique (IREC), Pôle Mont, Université catholique de Louvain (UCLouvain), Yvoir, Belgium), **Maurizio Muraca** (University of Padua, Padua, Italy; Istituto di Ricerca Pediatrica “Città della Speranza”, Padua, Italy), **Saravanakumar Murugesan** (University of Alabama at Birmingham, Birmingham, AL, USA), **Luca Musante** (School of Veterinary Medicine, University of Pennsylvania, Philadelphia, PA, USA), **Angelo Musicò** (Department of Molecular and Translational Medicine, University of Brescia, Brescia, Italy; CSGI, Center for Colloid and Surface Science, Florence, Italy), **Afrodité Németh** (Pázmány Péter Catholic University, Budapest, Hungary), **Krisztina Németh** (Department of Genetics, Cell‐ and Immunobiology, Semmelweis University, Budapest, Hungary; HUN‐REN‐SU Translational Extracellular Vesicle Research Group, Semmelweis University, Budapest, Hungary), **Amélie Nadeau** (Research Institute of McGill University Health Centre, Montreal, Canada; Department of Pathology, McGill University, Montreal, Canada), **Gi‐Hoon Nam** (Department of Biochemistry and Molecular Biology, Korea University College of Medicine, Seoul, Republic of Korea; SHIFTBIO.INC, Seoul, Republic of Korea), **Honami Naora** (University of Texas MD Anderson Cancer Center, Houston, TX, USA), **Riccardo Natoli** (Clear Vision Research Group, Eccles Institute of Neuroscience, John Curtin School of Medical Research, College of Health and Medicine, The Australian National University, Acton, ACT, Australia; School of Medicine and Psychology, College of Health and Medicine, The Australian National University, Acton, ACT, Australia), **Muhammad Nawaz** (Department of Rheumatology and Inflammation Research, Institute of Medicine, Sahlgrenska Academy, University of Gothenburg, Gothenburg, Sweden), **Irina Nazarenko** (Institute for Infection Prevention and Control, Faculty of Medicine, Medical Center—University of Freiburg, Freiburg, Germany; Hahn‐Schikard Institute, Freiburg, Germany ), **Justus C Ndukaife** (Department of Electrical and Computer Engineering, Vanderbilt University, Nashville, TN, USA; Department of Mechanical Engineering, Vanderbilt University, Nashville, TN, USA; The Vanderbilt Center for Extracellular Vesicle Research, School of Medicine, Vanderbilt University, Nashville, TN, USA), **Christina Nedeva** (La Trobe Institute for Molecular Science, La Trobe University, Melbourne, Australia), **Peter Nejsum** (Department of Clinical Medicine, Aarhus University, Aarhus, Denmark), **Inge Nelissen** (Health Department, Flemish Institute for Technological Research (VITO), Mol, Belgium), **Christian Neri** (Institute of Biology Paris‐Seine, Sorbonne Université, Paris, France; Centre National de la Recherche Scientifique, Paris, France; Institut National de la Santé et de la Recherche Médicale, Paris, France), **Tommaso Neri** (Centro Dipartimentale di Biologia Cellulare Cardiorespiratoria, Dipartimento di Patologia Chirurgica, Medica, Molecolare e dell'Area Critica, Università di Pisa, Pisa, Italy), **Paolo Neviani** (The Saban Research Institute of Children's Hospital Los Angeles, University of Southern California, Los Angeles, CA, USA), **Lauren A Newman** (College of Medicine and Public Health, Flinders University, Adelaide, Australia), **Chiew Yong Ng** (Universiti Kebangsaan Malaysia Medical Centre, Kuala Lumpur, Malaysia; National University of Singapore, Singapore), **Guillaume van Niel** (Institute of Psychiatry and Neuroscience of Paris (IPNP), INSERM U1266, Université Paris Cité, Paris, France; GHU‐Paris Psychiatrie et Neurosciences, Hôpital Sainte Anne, Paris, France), **Rienk Nieuwland** (Laboratory of Experimental Clinical Chemistry, Amsterdam University Medical Centers, Location AMC, University of Amsterdam, Amsterdam, The Netherlands; Amsterdam Vesicle Center, Amsterdam University Medical Centers, Location AMC, University of Amsterdam, Amsterdam, The Netherlands), **Nadezhda Nikiforova** (Rochester Institute of Technology, Rochester, NY, USA), **Leonardo Nimrichter** (Instituto de Microbiologia Paulo de Góes, Universidade Federal do Rio de Janeiro, Rio de Janeiro, Brazil), **Chitranshi Nitin** (Macquarie University, Sydney, Australia), **Makon‐Sébastien Njock** (Department of Pneumology, University Hospital of Liège, Liège, Belgium; University of Liège/GIGA Research Centre/Laboratory of Pneumology, Liège, Belgium), **Daniele Noël** (IRMB, INSERM, University of Montpellier, Montpellier, France), **Alessio Noghero** (Lovelace Biomedical Research Institute, Albuquerque, NM, USA), **John P Nolan** (Scintillon Institute, San Diego, CA, USA), **Sam Noppen** (Department of Microbiology, Immunology and Transplantation, Rega Institute, Laboratory of Virology and Chemotherapy, KU Leuven, Leuven, Belgium), **Antonio da Silva Novaes** (Department of Medicine, Federal University of Sao Paulo, Sao Paulo, Brazil; Department of Medicine, State University of New York at Stony Brook, Stony Brook, NY, USA), **Lorraine O'Driscoll** (School of Pharmacy and Pharmaceutical Sciences, Trinity College Dublin, Dublin, Ireland; Trinity Biomedical Sciences Institute, Trinity College Dublin, Dublin, Ireland; Trinity St. James's Cancer Institute, Trinity College Dublin, Dublin, Ireland), **Ana O'Loghlen** (Centre for Biological Research (CIB), Spanish National Research Council (CSIC), Madrid, Spain), **Takahiro Ochiya** (Tokyo Medical University, Tokyo, Japan), **Johannes Oesterreicher** (Ludwig Boltzmann Institute for Traumatology, The Research Centre in Cooperation with AUVA, Vienna, Austria; Austrian Cluster for Tissue Regeneration, Vienna, Austria), **Seung W Oh** (MDimune Inc., Seoul, Republic of Korea; BioDrone Therapeutics Inc., Seattle, WA, USA), **Attila Oláh** (Department of Physiology, Faculty of Medicine, University of Debrecen, Debrecen, Hungary), **Martin Olivier** (McGill University, Montreal, Canada; Research Institute of McGill University Health Centre, Montreal, Canada), **Siew Ling Ong** (New Zealand Leather & Shoe Research Association (LASRA), Palmerston North, New Zealand; AgResearch Limited, Palmerston North, New Zealand), **Angelica Ortiz** (New York University Grossman School of Medicine, New York, NY, USA), **Luis A Ortiz** (Department of Environmental and Occupational Health, Graduate School of Public Health, University of Pittsburgh, Pittsburgh, PA, USA), **Ole Østergaard** (Novo Nordisk Foundation, Center for Protein Research, Faculty of Health and Medical Sciences, University of Copenhagen, Copenhagen, Denmark), **Omar A Osorio** (Department of Medicine, Division of Pulmonary and Critical Care Medicine, Washington University School Of Medicine, St. Louis, MO, USA), **Xabier Osteikoetxea** (Semmelweis University, Budapest, Hungary; Hungarian Centre of Excellence for Molecular Medicine, Szeged, Hungary), **Matias Ostrowski** (INBIRS Institute, CONICET, University of Buenos Aires, Buenos Aires, Argentina), **David Otaegui** (Biogipuzkoa Health Research Institute, San Sebastián, Spain), **Alexander Otahal** (Center for Regenerative Medicine, University for Continuing Education Krems, Krems, Austria), **Patricia M M Ozawa** (Vanderbilt University, Nashville, TN, USA), **Dilara C Ozkocak** (Department of Biochemistry and Chemistry, La Trobe Institute for Molecular Science, La Trobe University, Melbourne, Australia; Research Centre for Extracellular Vesicles, La Trobe University, Melbourne, Australia), **Krisztina Pálóczi** (Semmelweis University, Budapest, Hungary), **Rocío Pérez‐González** (Alicante Institute for Health and Biomedical Research (ISABIAL), Alicante, Spain; Institute of Neuroscience (IN), University Miguel Hernández‐CSIC, San Juan de Alicante, Alicante, Spain), **Bianca C Pachane** (Universidade Federal de São Carlos—UFSCar, São Carlos, Brazil), **Hafiza Padinharayil** (Jubilee Mission Medical College and Research Institute, Thrissur, India), **Daan Paget** (Division of Cardiovascular Medicine, Radcliffe Department of Medicine, University of Oxford, Oxford, UK; Department of Pharmacology, University of Oxford, Oxford, UK; Department of Physiology and Pharmacology, Karolinska Institutet, Stockholm, Sweden), **Jerome Paggetti** (Tumor Stroma Interactions, Department of Cancer Research, Luxembourg Institute of Health, Luxembourg, Luxembourg), **Paola C Loreto Palacio** (Abigail Wexner Research Institute, Nationwide Children's Hospital, Columbus, OH, USA), **Christian P Pallasch** (Department I of Internal Medicine, Centre for Integrated Oncology (CIO) Aachen‐Bonn‐ Cologne Duesseldorf, University of Cologne, Cologne, Germany; Cologne Excellence Cluster for Cellular Stress Responses in Ageing‐Associated Diseases (CECAD), University of Cologne, Cologne, Germany; Centre for Molecular Medicine Cologne (CMMC), University of Cologne, Cologne, Germany), **Roberta Palmulli** (Department of Pathology, University of Cambridge, Cambridge, UK; Department of Biochemistry, University of Cambridge, Cambridge, UK), **Bairen Pang** (The First Affiliated Hospital of Ningbo University, Ningbo, China), **Liliia Paniushkina** (ExCT, PMU, Salzburg, Austria; Micalis, Inrae, Paris, France), **Paschalia Pantazi** (Institute of Reproductive and Developmental Biology, Imperial College London, London, UK), **Lucia Paolini** (Department of Medical and Surgical Specialties, Radiological Sciences and Public Health (DSMC), University of Brescia, Brescia, Italy ; Center for Colloid and Surface Science (CSGI), Florence, Italy), **Daniela L Papademetrio** (Departamento de Inmunología, Facultad de Farmacia y Bioquímica, University of Buenos Aires, Buenos Aires, Argentina; Hospital de Alta Complejidad del Bicentenario Esteban Echeverría, Unidad de Conocimiento Traslacional, Buenos Aires, Argentina), **Pietro Parisse** (Istituto Officina dei Materiali, National Research Council of Italy, Trieste, Italy; Elettra Sincrotrone Trieste S.C.p.A., Trieste, Italy), **Dong Jun Park** (University of California, San Diego, San Diego, CA, USA), **Juhee Park** (Center for Soft and Living Matter, Institute for Basic Science, Ulsan, Republic of Korea), **Young‐Gyun Park** (Korea Advanced Institute of Science and Technology, Daejeon, Republic of Korea), **James G Patton** (Department of Biological Sciences, Vanderbilt University, Nashville, TN, USA), **Nicholas J Peake** (Sheffield Hallam University, Sheffield, UK), **D Michiel Pegtel** (Department of Pathology, Amsterdam UMC, Vrije Universiteit Amsterdam, Amsterdam, The Netherlands; Cancer Center Amsterdam, Imaging and Biomarkers, Amsterdam, The Netherlands), **Héctor Peinado** (Microenvironment and Metastasis Laboratory, Molecular Oncology Programme, Spanish National Cancer Research Center (CNIO), Madrid, Spain), **Francesca Perut** (Biomedical Science and Technologies and Nanobiotechnology Laboratory, IRCCS Istituto Ortopedico Rizzoli, Bologna, Italy), **Michael W Pfaffl** (Division of Animal Physiology and Immunology, School of Life Sciences Weihenstephan, Technical University of Munich, Freising, Germany), **Annika Pfeiffer** (Laboratory of Allergic Diseases, Division of Intramural Research, National Institute of Allergy and Infectious Diseases, National Institutes of Health, Bethesda, MD, USA), **Thanh Kha Phan** (Department of Biochemistry and Chemistry, La Trobe Institute for Molecular Science, La Trobe University, Melbourne, Australia), **Donald G Phinney** (Department of Molecular Medicine, The Herbert Wertheim UF Scripps Institute for Biomedical Innovation and Technology, Jupiter, FL, USA), **Leonidas A Phylactou** (The Cyprus Institute of Neurology & Genetics, Nicosia, Cyprus), **Silvia Picciolini** (IRCCS Fondazione Don Carlo Gnocchi Onlus, Milan, Italy), **Monika Pietrowska** (Maria Sklodowska‐Curie National Research Institute of Oncology, Gliwice, Poland), **Max Piffoux** (Centre Léon Bérard, Lyon, France; Hospices civils de Lyon, Lyon, France), **Cláudio Pinheiro** (Laboratory of Experimental Cancer Research, Department of Human Structure and Repair, Ghent University, Ghent, Belgium; Cancer Research Institute Ghent, Ghent, Belgium), **Ryan C Pink** (Department of Biological and Medical Sciences, Faculty of Health and Life Sciences, Oxford Brookes University, Oxford, UK), **Michelle L Pleet** (Viral Immunology Section, Neuroimmunology Branch, National Institute of Neurological Disorders and Stroke, National Institutes of Health, Bethesda, MD, USA), **Gabriella Pocsfalvi** (Institute of Biosciences and BioResourses, National Research Council, Napoli, Italy), **Qi Hui Poh** (La Trobe University, Melbourne, Australia; Baker Heart & Diabetes Institute, Melbourne, Australia), **Edwin van der Pol** (Biomedical Engineering and Physics, Amsterdam UMC, location AMC, University of Amsterdam, Amsterdam, The Netherlands; Laboratory of Experimental Clinical Chemistry, Amsterdam UMC, location AMC, University of Amsterdam, Amsterdam, The Netherlands; Amsterdam Vesicle Center, Amsterdam University Medical Centers, Location AMC, University of Amsterdam, Amsterdam, The Netherlands), **Ganesha Poojary** (Department of Physiotherapy, Manipal College of Health Professions, Manipal Academy of Higher Education, Manipal, Karnataka, India), **Ivan KH Poon** (Department of Biochemistry and Chemistry, La Trobe Institute for Molecular Science, La Trobe University, Melbourne, Australia; Research Centre for Extracellular Vesicles, La Trobe University, Melbourne, Australia), **Giuseppina Poppa** (Department of Life, Health and Environmental Sciences, University of L'Aquila, L'Aquila, Italy), **Hernando A del Portillo** (ISGlobal, Barcelona Institute for Global Health, Hospital Clínic‐Universitat de Barcelona, Barcelona, Spain; IGTP, Germans Trias i Pujol Health Research Institute, Badalona, Spain; Catalan Institution for Research and Advanced Studies (ICREA), Barcelona, Spain), **Vendula Pospichalova** (Faculty of Science, Masaryk University, Brno, Czech Republic), **Shirley Potter** (Mater Misericordiae University Hospital Dublin, Dublin, Ireland; The Conway Institute of Biomedical Science, University College Dublin, Dublin, Ireland; Irish Cancer Society, Dublin, Ireland), **Bonita H Powell** (Johns Hopkins University, Baltimore, MD, USA), **Simon J Powis** (University of St Andrews, St Andrews, UK), **Ilaria Prada** (Axxam S.p.A., Bresso, Milan, Italy), **Indira Prasadam** (Centre for Biomedical Technologies, School of Mechanical, Medical, and Process Engineering, Faculty of Engineering, Queensland University of Technology, Brisbane, Australia), **Christian Preußer** (Institute for Tumor Immunology, Philipps University Marburg, Marburg, Germany; EV Core Facility, Philipps University Marburg, Marburg, Germany), **Heather H Pua** (Department of Pathology, Microbiology, and Immunology, Vanderbilt University Medical Center, Nashville, TN, USA; Vanderbilt Center for Immunobiology, Vanderbilt University Medical Center, Nashville, TN, USA), **Ferdinando Pucci** (Department of Otolaryngology, Head & Neck Surgery, Oregon Health & Science University, Portland, OR, USA; Cell, Developmental and Cancer Biology, Oregon Health & Science University, Portland, OR, USA), **Florian Puhm** (Département de microbiologie et immunologie, Faculté de Médecine de l'Université Laval, Université Laval, Quebec, Canada; Centre de Recherche ARThrite (Arthrite Recherche Traitements) de l'Université Laval, Quebec, Canada), **Berta Puig** (Experimental Research in Stroke and Inflammation (ERSI) Group, Neurology Department, University Medical Center Hamburg‐Eppendorf, Hamburg, Germany), **Lynn Pulliam** (University of California, San Francisco, San Francisco, CA, USA; Veterans Affairs Health Care, San Francisco, San Francisco, CA, USA), **Adityas Purnianto** (The University of Melbourne, Parkville, Australia; The Florey Institute of Neuroscience and Mental Health, The University of Melbourne, Parkville, Australia), **Johanna MM Puutio** (EV Group, Molecular and Integrative Biosciences Research Programme, Faculty of Biological and Environmental Sciences, University of Helsinki, Helsinki, Finland; EV Core, Molecular and Integrative Biosciences Research Programme, Faculty of Biological and Environmental Sciences, University of Helsinki, Helsinki, Finland), **Rachel C Quilang** (Leiden University Medical Center, Leiden, The Netherlands), **Piul S Rabbani** (New York University School of Medicine, New York, NY, USA), **Gorjana Rackov** (BioMed X GmbH, Heidelberg, Germany), **Annalisa Radeghieri** (University of Brescia, Department of Molecular and Translational Medicine, Brescia, Italy; CSGI—Research Center for Colloids and Nanoscience, Florence, Italy), **Claudia M Radu** (Department of Medicine, University of Padua, Padua, Italy), **Robert L Raffai** (University of California, San Francisco, San Francisco, CA, USA; Department of Veterans Affairs, San Francisco, CA, USA), **Alok Raghav** (Department of Anatomy and Cell Biology, Lee Gill Ya Cancer and Diabetes Institute, Gachon University, Incheon, Republic of Korea; Multidisciplinary Research Unit, GSVM Medical College Kanpur, Uttar Pradesh, India), **Mohammad Rahbari** (German Cancer Research Center, Division of Chronic Inflammation and Cancer, Heidelberg, Germany; Department of Surgery, University Mannheim, Medical Faculty Mannheim, University Hospital Heidelberg, Mannheim, Germany), **MD Matiur Rahman** (Department of Medicine, Faculty of Veterinary, Animal and Biomedical Sciences, Sylhet Agricultural University, Sylhet, Bangladesh; Laboratory of Food and Environmental Hygiene, Gifu University, Gifu, Japan), **Md Mostafizur Rahman** (South Asian University, New Delhi, India), **Alex J Rai** (Department of Pathology & Cell Biology, Columbia University Irving Medical Center, New York, NY, USA), **Stefania Raimondo** (Department of Biomedicine, Neuroscience and Advanced Diagnostics (Bi.N.D.), University of Palermo, Palermo, Italy), **Sneha Raju** (Faculty of Medicine, University of Toronto, Toronto, Canada), **Janusz Rak** (McGill University, Montreal, Canada), **Lausonia Ramaswamy** (Sysmex Corporation, Kobe, Japan), **Javier Ramirez‐Ricardo** (Department of Pathology, Microbiology, and Immunology, Vanderbilt University Medical Center, Nashville, TN, USA), **Marcel I Ramirez** (Carlos Chagas Institute, Oswaldo Cruz Foundation (Fiocruz), Curitiba, Brazil), **Shikha Rani** (The University of Queensland, Brisbane, Australia), **Graca Raposo** (Institut Curie, PSL University, Paris, France; CNRS UMR144, Paris, France), **Hilal A Rather** (Wake Forest School of Medicine, Winston‐Salem, NC, USA; Duke University, Durham, NC, USA), **Agnieszka Razim** (Medical University of Vienna, Vienna, Austria; Hirszfeld Institute of Immunology and Experimental Therapy, Polish Academy of Sciences, Wroclaw, Poland), **Antonia Reale** (Myeloma Research Group, Australian Centre for Blood Diseases, Central Clinical School, Monash University—The Alfred, Melbourne, Australia; Medical Oncology, Cabrini Malvern, Melbourne, Australia), **Eduardo Reategui** (William G. Lowrie Department of Chemical and Biomolecular Engineering, The Ohio State University, Columbus, OH, USA; Comprehensive Cancer Center, The Ohio State University, Columbus, OH, USA), **Caroline J Reddel** (ANZAC Research Institute, Concord Repatriation General Hospital, Concord, Australia; The University of Sydney, Camperdown, Australia), **Shivakumar K Reddy** (Centre for Molecular Neurosciences, Kasturba Medical College Manipal, Manipal Academy of Higher Education, Manipal, India), **Stephen Redenti** (Department of Biology, Lehman College, City University of New York, New York, NY, USA; Biochemistry and Biology Doctoral Programs, City University of New York, New York, NY, USA), **Samantha L Reed** (Emory University Department of Human Genetics, Atlanta, GA, USA), **Neta Regev‐Rudzki** (Department of Biomolecular Sciences, Weizmann Institute of Science, Rehovot, Israel), **Katrin S Reiners** (Institute of Clinical Chemistry and Clinical Pharmacology, University Hospital Bonn, Bonn, Germany), **Nataša Resnik** (Institute of Cell Biology, Faculty of Medicine, University of Ljubljana, Ljubljana, Slovenia), **Gregory E Rice** (Centre for Clinical Research, The University of Queensland, Herston, Australia; Inoviq Limited, Notting Hill, Australia), **Franz L Ricklefs** (Department of Neurosurgery, University Medical Center Hamburg‐Eppendorf, Hamburg, Germany), **Andrea Ridolfi** (Department of Physics and Astronomy, and LaserLaB Amsterdam, Vrije Universiteit Amsterdam, Amsterdam, The Netherlands), **Kirsi Rilla** (University of Eastern Finland, Kuopio, Finland), **Michael P Rimmer** (Institute of Regeneration and Repair, Centre for Reproductive Health, University of Edinburgh, Edinburgh, UK; School of Medicine, University of St Andrews, Fife, UK), **Kelly CS Roballo** (Edward Via College of Osteopathic Medicine, Blacksburg, VA, USA; Virginia Maryland College of Veterinary Medicine, Virginia Tech, Blacksburg, Virginia, USA), **Paul D Robbins** (Institute on the Biology of Aging, University of Minnesota, Minneapolis, MN, USA), **David D Roberts** (Laboratory of Pathology, Center for Cancer Research, National Cancer Institute, National Institutes of Health, Bethesda, MD, USA), **Jordi Roca** (Department of Medicine and Animal Surgery, Veterinary Science, University of Murcia, Murcia, Spain), **Avital A Rodal** (Brandeis University, Waltham, MA, USA), **Dorival M Rodrigues‐Junior** (Department of Medical Biochemistry and Microbiology, Science for Life Laboratory, Biomedical Center, Uppsala University, Uppsala, Sweden), **Marcio L Rodrigues** (Carlos Chagas Institute, Oswaldo Cruz Foundation (Fiocruz), Curitiba, Brazil; Paulo de Goes Microbiology Institute, Federal University of Rio de Janeiro (UFRJ), Rio de Janeiro, Brazil), **Marieke T Roefs** (Evercyte GmbH, Vienna, Austria), **Russell G Rogers** (Smidt Heart Institute, Cedars‐Sinai Medical Center, Los Angeles, CA, USA), **Eva Rohde** (Department of Transfusion Medicine, University Hospital, Salzburger Landeskliniken GmbH of Paracelsus Medical University, Salzburg, Austria; GMP Unit, Paracelsus Medical University, Salzburg, Austria; Transfer Centre for Extracellular Vesicle Theralytic Technologies, EV‐TT, Salzburg, Austria), **Tatu Rojalin** (Department of Biomedical Engineering, University of California, Davis, Davis, CA, USA; Expansion Therapeutics, Structural Biology and Biophysics, Jupiter, FL, USA), **Rita Romani** (Department of Medicine and Surgery, University of Perugia, Perugia, Italy), **Miriam Romano** (Center for Colloid and Surface Science (CSGI), Florence, Italy; Department of Molecular and Translational Medicine, University of Brescia, Brescia, Italy), **Sophie Rome** (CarMeN Laboratory, UMR INRAE 1397/INSERM 1060, University of Lyon, Lyon, France), **Rok Romih** (Institute of Cell Biology, Faculty of Medicine, University of Ljubljana, Ljubljana, Slovenia), **Anna Romolo** (Laboratory of Clinical Biophysics, Faculty of Health Sciences, University of Ljubljana, Ljubljana, Slovenia), **Tatiane De Rossi** (Brazilian Biosciences National Laboratory, Brazilian Center for Research in Energy and Materials (CNPEM), Campinas, Brazil), **Kasper M Rouschop** (Department of Radiotherapy, GROW‐School for Oncology and Reproduction, Maastricht University Medical Centre+, Maastricht, The Netherlands), **David A Routenberg** (Meso Scale Diagnostics, LLC., Rockville, MD, USA), **Quentin Roux** (Centre de Recherche en Cancérologie et Immunologie Intégrée Nantes Angers, INSERM U1307, CNRS UMR6075, Nantes University, Nantes, France), **Andrew Rowland** (College of Medicine and Public Health, Flinders University, Adelaide, Australia), **Martin E van Royen** (Department of Pathology, Erasmus MC, Rotterdam, The Netherlands), **Annaïg J Rozo** (Aston University, Birmingham, UK), **David Rufino‐Ramos** (Center for Neuroscience and Cell Biology, University of Coimbra, Coimbra, Portugal; Center for Innovative Biomedicine and Biotechnology, University of Coimbra, Coimbra, Portugal; Center for Genomic Medicine, Massachusetts General Hospital, Boston, MA, USA), **Aurelia Rughetti** (Department Experimental Medicine, Sapienza University of Rome, Rome, Italy ), **Ashley E Russell** (Department of Biology, School of Science, Penn State Erie, The Behrend College, Erie, PA, USA), **Stephanie F Rutter** (La Trobe Institute for Molecular Science, La Trobe University, Melbourne, Australia), **Myltykbay S Rysmakhanov** (West‐Kazakhstan Medical University, Aktobe, Kazakhstan), **Catherine A Sánchez** (Academic Department, Clínica Las Condes, Santiago, Chile; Faculty of Medicine, Universidad de Chile, Santiago, Chile), **Yoel Sadovsky** (Magee Womens Research Institute, University of Pittsburgh, Pittsburgh, PA, USA), **Reihaneh Safavi‐Sohi** (Department of Chemistry and Biochemistry, University of Notre Dame, Notre Dame, IN, USA; Harper Cancer Research Institute, University of Notre Dame, Notre Dame, IN, USA; Department of Chemistry and Biochemistry, Seton Hall University, South Orange, NJ, USA), **Andras Saftics** (Department of Molecular Medicine, Beckman Research Institute, City of Hope Comprehensive Cancer Center, Duarte, CA, USA), **Ram Sagar** (Department of Genetic Medicine, Johns Hopkins University School of Medicine, Baltimore, MD, USA), **Susmita Sahoo** (Icahn School of Medicine at Mount Sinai, New York, NY, USA), **Nathaniel EB Saidu** (Department of Cancer Immunology, Institute of Cancer Research, Oslo University Hospital, Oslo, Norway), **Julien Saint‐Pol** (Blood‐Brain Barrier Laboratory (LBHE), UR 2465, University of Artois, Lens, France), **Edison Salas‐Huenuleo** (Advanced Integrated Technologies, Santiago, Chile), **Ana I Salazar‐Puerta** (The Ohio State University, Columbus, OH, USA), **Ayesha Saleem** (Faculty of Kinesiology, University of Manitoba, Winnipeg, Canada; Children's Hospital Research Institute of Manitoba (CHRIM), Winnipeg, Canada), **Ghasem Hosseini Salekdeh** (School of Natural Sciences, Macquarie University, Sydney, Australia), **Carlos Salomon** (Translational Extracellular Vesicles in Obstetrics and Gynae‐Oncology Group, University of Queensland Centre for Clinical Research, Faculty of Medicine, Royal Brisbane and Women's Hospital, The University of Queensland, Brisbane, Australia; Departamento de Investigación, Postgrado y Educación Continua (DIPEC), Facultad de Ciencias de la Salud, Universidad del Alba, Santiago, Chile), **Amanda Salviano‐Silva** (Department of Neurosurgery, University Medical Center Hamburg‐Eppendorf, Hamburg, Germany), **Amankeldi A Salybekov** (Qazaq Institute of Innovative Medicine, Regenerative Medicine Division, Cell and Gene Therapy Department, Astana, Kazakhstan; Kidney Disease and Transplant Center, Shonan Kamakura General Hospital, Kamakura, Japan), **Mark Samuels** (Department of Biochemistry and Biomedicine, School of Life Sciences, University of Sussex, Brighton, UK), **Ursula S Sandau** (Department of Anesthesiology & Perioperative Medicine, Oregon Health & Science University, Portland, OR, USA), **Jascinta P Santavanond** (Department of Biochemistry and Chemistry, La Trobe Institute for Molecular Science, La Trobe University, Melbourne, Australia; Research Centre for Extracellular Vesicles, La Trobe University, Melbourne, Australia), **Jessie Santoro** (School of Pharmacy and Pharmaceutical Sciences & Trinity Biomedical Sciences Institute, Trinity College Dublin, Dublin, Ireland), **Mark Santos** (Touro University Nevada, Henderson, NV, USA), **Rahul Sanwlani** (La Trobe Institute for Molecular Science, La Trobe University, Melbourne, Australia; Faculty of Health and Medical Sciences, School of Biosciences, University of Surrey, Surrey, UK), **Julie A Saugstad** (Department of Anesthesiology & Perioperative Medicine, Oregon Health & Science University, Portland, OR, USA), **Meike J Saul** (II. Medical Clinic and Polyclinic, University Medical Center Hamburg‐Eppendorf, Hamburg, Germany), **Tine Hiorth Schøyen** (Cardiovascular Research Group, Department of Clinical Medicine, University of Tromsø—The Arctic University of Norway, Tromsø, Norway), **Irma Schabussova** (Institute of Specific Prophylaxis and Tropical Medicine, Center for Pathophysiology, Infectiology, and Immunology, Medical University of Vienna, Vienna, Austria), **Emilia Scharrig** (Thomas Jefferson University, Philadelphia, PA, USA), **Randy Schekman** (Department of Molecular and Cell Biology and Howard Hughes Medical Institute, University of California, Berkeley, Berkeley, CA, USA), **Jessica Schiavi‐Tritz** (CNRS, LRGP, University of Lorraine, Nancy, France), **Raymond M Schiffelers** (CDL Research, University Medical Center Utrecht, Utrecht, The Netherlands), **Anna M Schmid** (Institute of Specific Prophylaxis and Tropical Medicine, Center for Pathophysiology, Infectiology, and Immunology, Medical University of Vienna, Vienna, Austria), **Raphael Schneider** (University of Toronto, Toronto, Canada), **Stefan Schneider** (Curexsys GmbH, Goettingen, Germany), **Andreina Schoeberlein** (Department of Obstetrics and Feto‐maternal Medicine, University Women's Hospital, Inselspital, Bern University Hospital, Bern, Switzerland; Department for BioMedical Research (DBMR), University of Bern, Bern, Switzerland), **Jeffrey S Schorey** (Department of Biological Sciences, University of Notre Dame, Notre Dame, IN, USA), **Naohiro Seo** (Nanobio Device Laboratory, Graduate School of Bioengineering, The University of Tokyo, Tokyo, Japan), **Joaquin Seras‐Franzoso** (Clinical Biochemistry, Drug Delivery & Therapy (CB‐DDT), Vall d'Hebron Institut de Recerca (VHIR), Universitat Autònoma de Barcelona (UAB), Barcelona, Spain; Networking Research Center on Bioengineering, Biomaterials and Nanomedicine (CIBER‐BBN), Instituto de Salud Carlos III, Madrid, Spain; Department of Genetics and Microbiology, Universitat Autònoma de Barcelona (UAB), Bellaterra, Spain), **Sanjay Shahi** (Department of Biochemistry and Genetics, La Trobe Institute for Molecular Science, La Trobe University, Melbourne, Australia), **Olga Shatnyeva** (Cell Therapy, Evotec International GmbH, Goettingen, Germany), **Deanna F Shea** (School of Biological Sciences, University of Auckland, Auckland, New Zealand), **Faezeh Shekari** (Department of Stem Cells and Developmental Biology, Cell Science Research Center, Royan Institute for Stem Cell Biology and Technology, ACECR, Tehran, Iran; Celer Diagnostics, Toronto, Canada), **Ganesh V Shelke** (Neurosciences and Cellular and Structural Biology Division, Eunice Kennedy Shriver National Institute of Child Health and Human Development, National Institutes of Health, Bethesda, MD, USA), **Ashok K Shetty** (Institute for Regenerative Medicine, Texas A&M University School of Medicine, College Station, Texas, USA; Department of Cell Biology and Genetics, Texas A&M University School of Medicine, College Station, Texas, USA), **Kiyotaka Shiba** (Cancer Institute, Japanese Foundation for Cancer Research, Tokyo, Japan), **Thomas Michael Shiju** (Cole Eye Institute, Cleveland Clinic, Cleveland, OH, USA), **Surya Shrivastava** (Novartis Biomedical Research, San Diego, CA, USA), **Sachin Shukla** (L V Prasad Eye Institute, Hyderabad, India), **Pia R‐M Siljander** (EV Group, Molecular and Integrative Biosciences Research Programme, Faculty of Biological and Environmental Sciences, University of Helsinki, Helsinki, Finland; EV Core, Molecular and Integrative Biosciences Research Programme, Faculty of Biological and Environmental Sciences, University of Helsinki, Helsinki, Finland), **Andreia M Silva** (Anjarium Biosciences AG, Schlieren, Switzerland), **Ajay P Singh** (Department of Pathology and Mitchell Cancer Institute, Frederick P. Whiddon College of Medicine, University of South Alabama, Mobile, AL, USA), **Sangeeta Singh** (Wake Forest University, Winston‐Salem, NC, USA), **Mikhail Skliar** (The University of Utah, Salt Lake City, UT, USA), **Johan Skog** (Exosome Diagnostics, a Bio‐Techne brand, Boston, MA, USA), **Joost PG Sluijter** (Laboratory of Experimental Cardiology, Department of Cardiology, University Medical Center Utrecht, The Netherlands; UMC Utrecht Regenerative Medicine Center, Circulatory Health Research Center, University Medical Center Utrecht, Utrecht, The Netherlands), **Orman L Snyder** (Kansas State University, Manhattan, KS, USA), **Carolina Soekmadji** (School of Biomedical Sciences, Faculty of Medicine, The University of Queensland, Brisbane, Australia), **Ahmed Somaida** (Department of Pharmaceutics and Biopharmaceutics, Philipps University Marburg, Marburg, Germany), **Masaharu Somiya** (SANKEN, Osaka University, Ibaraki, Japan), **Karolina Soroczyńska** (Department of Biochemistry, Medical University of Warsaw, Warsaw, Poland; Postgraduate School of Molecular Medicine, Medical University of Warsaw, Warsaw, Poland), **Javier Sotillo** (Instituto de Salud Carlos III, Majadahonda, Spain), **Fernando Souza‐Fonseca‐Guimaraes** (Frazer Institute, The University of Queensland, Woolloongabba, Australia), **Sheila Spada** (Tumor of Immunology and Immunotherapy Unit, IRCCS Regina Elena National Cancer Institute, Rome, Italy), **Harry VM Spiers** (Department of Surgery, University of Cambridge, Cambridge, UK; Wellcome‐MRC Cambridge Stem Cell Unit, University of Cambridge, Cambridge, UK; Department of Transplantation, Addenbrooke's Hospital, Cambridge, UK), **Joshua D Spitzberg** (Center for Systems Biology, Massachusetts General Hospital, Boston, MA, USA), **Akhil Srivastava** (Ellis Fischel Cancer Center, University of Missouri School of Medicine, Columbia, MO, USA), **Amit K Srivastava** (Department of Medicine, Sidney Kimmel Medical College, Thomas Jefferson University, Cardeza Foundation for Hematologic Research, Philadelphia, PA, USA), **Ewa Ł Stępień** (Department of Medical Physics, M. Smoluchowski Institute of Physics, Faculty of Physics, Astronomy and Applied Computer Science, Jagiellonian University, Krakow, Poland; Center for Theranostics, Jagiellonian University, Krakow, Poland), **Frederic St‐Denis‐Bissonnette** (Health Canada, Ontario, Canada; University of Ottawa, Ottawa, Canada), **Philip D Stahl** (Washington University, St. Louis, MO, USA), **Janine Stam** (Department of Analytical Biochemistry, Groningen Research Institute of Pharmacy, University of Groningen, Groningen, The Netherlands), **Oumaima Stambouli** (Institute for Transfusion Medicine, University Hospital Essen, University of Duisburg‐Essen, Essen, Germany), **Bruce A Stanton** (Geisel School of Medicine at Dartmouth, Hanover, NH, USA), **Frank RM Stassen** (Maastricht University, Maastricht, The Netherlands), **Oskar Staufer** (INM—Leibniz Institute for New Materials, Saarbruecken, Germany; Max Planck Bristol Center for Minimal Biology, Bristol, UK; Center for Biophysics, Saarbruecken University, Saarbruecken, Germany), **Loïc Steiner** (Division of Immunology and Allergy, Department of Medicine, Karolinska Institutet, Stockholm, Sweden; Department of Clinical Immunology and Transfusion Medicine, Karolinska University Hospital, Stockholm, Sweden), **Ganna Stepanova** (Faculty of Medicine, Institute of Translational Medicine, Semmelweis University, Budapest, Hungary), **Veronika Stoka** (J. Stefan Institute, Ljubljana, Slovenia), **Willem Stoorvogel** (Department Biomolecular Health Sciences, Faculty of Veterinary Medicine, Utrecht University, Utrecht, The Netherlands), **Elke Pogge von Strandmann** (Institute for Tumor Immunology, Philipps University Marburg, Marburg, Germany; EV Core Facility, Philipps University Marburg, Marburg, Germany), **Dirk Strunk** (Cell Therapy Institute, Paracelsus Medical University, Salzburg, Austria), **Stanley S Stylli** (Department of Surgery (RMH), The University of Melbourne, Parkville, Australia; Department of Neurosurgery, Royal Melbourne Hospital, Parkville, Australia), **Huaqi Su** (The Florey Institute of Neuroscience and Mental Health, The University of Melbourne, Parkville, Australia), **Subbaya Subramanian** (Department of Surgery, University of Minnesota, Minneapolis, MN, USA; Center for Immunology, University of Minnesota, Minneapolis, MN, USA; Masonic Cancer Center, University of Minnesota, Minneapolis, MN, USA), **Bingdong Sui** (The Fourth Military Medical University, Xi'an, China), **Sonal Sukreet** (University of California, San Diego, San Diego, CA, USA; University of Nebraska‐Lincoln, Lincoln, NE, USA), **Elias Sulaiman** (The Hatter Cardiovascular Institute, University College London, London, UK), **Bong Hwan Sung** (Department of Cell and Developmental Biology, School of Medicine, Vanderbilt University, Nashville, TN, USA; The Vanderbilt Center for Extracellular Vesicle Research, School of Medicine, Vanderbilt University, Nashville, TN, USA), **Vijaya Sunkara** (Ulsan National Institute of Science & Technology, Ulsan, Republic of Korea; Center for Soft and Living Matter, Institute for Basic Science, Ulsan, Republic of Korea), **Zucai Suo** (Department of Biomedical Sciences, Florida State University College of Medicine, Tallahassee, FL, USA), **Per Svenningsen** (Department of Molecular Medicine, University of Southern Denmark, Odense, Denmark), **Julian Swatler** (Nencki Institute of Experimental Biology, Warsaw, Poland; IRCCS Humanitas Research Hospital, Rozzano, Milan, Italy), **Simon Swift** (Waipapa Taumata Rau University of Auckland, Auckland, New Zealand), **Emma KC Symonds** (University of Otago, Wellington, New Zealand), **Viktoria Szeifert** (Stanford University, Department of Pathology, Stanford, CA, USA), **Imola Cs Szigyártó** (Research Centre for Natural Sciences, Institute of Materials and Environmental Chemistry, Budapest, Hungary), **Eszter Á Tóth** (Department of Genetics, Cell‐ and Immunobiology, Semmelweis University, Budapest, Hungary), **Neslihan P Taşlı** (Department of Genetics and Biotechnology, Yeditepe University, İstanbul, Turkey), **Hidetoshi Tahara** (Institute of Biomedical & Health Sciences, Department of Cellular and Molecular Biology, Hiroshima University, Hiroshima, Japan), **Ryou‐u Takahashi** (Department of Cellular and Molecular Biology, Graduate School of Biomedical and Health Science, Hiroshima University, Hiroshima, Japan), **Yoshinobu Takakura** (Graduate School of Pharmaceutical Sciences, Kyoto University, Kyoto, Japan), **Osamu Takikawa** (National Center for Geriatrics and Gerontology, Aichi, Japan), **Kaloyan Takov** (National Heart and Lung Institute, Imperial College London, London, UK), **Vera A Tang** (Flow Cytometry & Virometry Core Facility, Department of Biochemistry, Microbiology, & Immunology, University of Ottawa, Ottawa, Canada), **Simona Taverna** (Institute of Translational Pharmacology (IFT), National Research Council of Italy (CNR), Palermo, Italy), **Nadim Tawil** (Research Institute of McGill University Health Centre, Montreal, Canada), **Loes Teeuwen** (Karolinska Institutet, Solna, Sweden), **Sandra Tejedor** (System Biology Research Center, University of Skövde, Skövde, Sweden ; Research and Early Development, Cardiovascular, Renal and Metabolism (CVRM), Biopharmaceuticals R&D, AstraZeneca, Gothenburg, Sweden), **Dmitry Ter‐Ovanesyan** (Wyss Institute for Biologically Inspired Engineering, Harvard University, Boston, MA, USA), **Tobias Tertel** (Institute for Transfusion Medicine, University Hospital Essen, University of Duisburg‐Essen, Essen, Germany), **Clotilde Théry** (Institut Curie, INSERM U932, PSL University, Paris, France; CurieCoreTech Extracellular Vesicles, Institut Curie, Paris, France), **Abhimanyu Thakur** (Pritzker School of Molecular Engineering, Ben May Department for Cancer Research, University of Chicago, Chicago, IL, USA; Department of Neurosurgery, Massachusetts General Hospital, Harvard Medical School, Boston, MA, USA), **Tara Thompson‐Felix** (Yale University, New Haven, CT, USA), **Changhai Tian** (Department of Toxicology and Cancer Biology, University of Kentucky College of Medicine, Lexington, KY, USA), **Aleksei Tikhonov** (Gustave Roussy, Villejuif, France), **Swasti Tiwari** (Sanjay Gandhi Postgraduate Institute of Medical Sciences, Lucknow, India; Georgetown University, Washington, DC, USA), **Wei Seong Toh** (Department of Orthopaedic Surgery, Yong Loo Lin School of Medicine, National University of Singapore, Singapore), **John J Tomes** (Aberystwyth University, Aberystwyth, UK), **Elisa Tonoli** (Nottingham Trent University, Nottingham, UK), **Ana C Torrecilhas** (Laboratório de Imunologia Celular e Bioquímica de Fungos e Protozoários, Departamento de Ciências Farmacêuticas, Instituto de Ciências Ambientais, Químicas e Farmacêuticas, Universidade Federal de São Paulo (UNIFESP) Campus Diadema, Diadema, Brazil), **Juan P Tosar** (Universidad de la República, Montevideo, Uruguay; Institut Pasteur de Montevideo, Montevideo, Uruguay), **Camille V Trinidad** (University of Kansas Medical Center, Kansas City, KS, USA), **Lucienne Tritten** (Swiss Tropical and Public Health Institute, Allschwil, Switzerland; University of Basel, Basel, Switzerland; Institute of Parasitology, University of Zurich, Zurich, Switzerland), **Rucha Trivedi** (School of Biomedical Science, University of North Texas Health Science Center, Fort Worth, TX, USA), **Zach Troyer** (Department of Molecular and Comparative Pathobiology, Johns Hopkins University School of Medicine, Baltimore, MD, USA), **Migmar Tsamchoe** (Department of Anatomy and Cell Biology, McGill University, Quebec, Canada; McGill University Health Centre Research Institute, Quebec, Canada), **Vera Tscherrig** (Department of Obstetrics and Feto‐maternal Medicine, University Women's Hospital, Inselspital, Bern University Hospital, Bern, Switzerland; Graduate School for Cellular and Biomedical Sciences (GCB), University of Bern, Bern, Switzerland; Department for BioMedical Research (DBMR), University of Bern, Bern, Switzerland), **Thupten Tsering** (Research Institute of McGill University Health Centre, Montreal, Canada; Department of Pathology, McGill University, Montreal, Canada), **Kristyna Turkova** (Institute of Biophysics of the Czech Academy of Sciences, Brno, Czech Republic; St. Anne's University Hospital Brno, International Clinical Research Center,Brno, Czech Republic), **Oleg S Tutanov** (Vanderbilt University Medical Center, Nashville, TN, USA), **Koji Ueda** (Japanese Foundation for Cancer Research, Tokyo, Japan), **Dinesh Upadhya** (Centre for Molecular Neurosciences, Kasturba Medical College Manipal, Manipal Academy of Higher Education, Manipal, India), **Fumihiko Urabe** (Department of Urology, The Jikei University School of Medicine, Tokyo, Japan), **Lorena Urbanelli** (Department of Chemistry, Biology and Biotechnology, University of Perugia, Perugia, Italy), **Ornella Urzì** (Dipartimento di Biomedicina, Neuroscienze e Diagnostica Avanzata (Bi.N.D), sezione di Biologia e Genetica, University of Palermo, Palermo, Italy; Sahlgrenska Center for Cancer Research and Wallenberg Centre for Molecular and Translational Medicine, Department of Surgery, Institute of Clinical Sciences, Sahlgrenska Academy, University of Gothenburg, Gothenburg, Sweden), **Zivile Useckaite** (College of Medicine and Public Health, Flinders University, Adelaide, Australia), **Elena Vacchi** (Neurodegenerative Diseases Group, Laboratory for Translational Research, Ente Ospedaliero Cantonale, Bellinzona, Switzerland), **Pieter Vader** (University Medical Center Utrecht, Utrecht, The Netherlands), **Riccardo Vago** (IRCCS San Raffaele Scientific Institute, Milan, Italy; Università Vita‐Salute San Raffaele, Milan, Italy), **Hadi Valadi** (Department of Rheumatology and Inflammation Research, Institute of Medicine, Sahlgrenska Academy, University of Gothenburg, Gothenburg, Sweden), **Sami Valkonen** (Division of Pharmaceutical Biosciences, Faculty of Pharmacy, University of Helsinki, Helsinki, Finland; School of Pharmacy, University of Eastern Finland, Kuopio, Finland), **Francesco Valle** (Consiglio Nazionale delle Ricerche—Istituto per lo Studio dei Materiali Nanostrutturati, Bologna, Italy; Consorzio Interuniversitario per lo Sviluppo dei Sistemi a Grande Interfase, Florence, Italy), **Manuel Varas‐Godoy** (Centro de Biología Celular y Biomedicina, Facultad de Medicina y Ciencia, Universidad San Sebastián, Santiago, Chile; Centro Ciencia & Vida, Fundación Ciencia & Vida, Santiago, Chile; Advanced Center for Chronic Diseases, Santiago, Chile), **Zoltán Varga** (Biological Nanochemistry Research Group, Institute of Materials and Environmental Chemistry, Research Centre for Natural Sciences, Budapest, Hungary; Department of Physical Chemistry and Materials Science, Faculty of Chemical Technology and Biotechnology, Budapest University of Technology and Economics, Budapest, Hungary), **Zoltan Varga** (Biological Nanochemistry Research Group, Institute of Materials and Environmental Chemistry, Research Centre for Natural Sciences, Budapest, Hungary; Department of Biophysics and Radiation Biology, Semmelweis University, Budapest, Hungary), **M Helena Vasconcelos** (Faculty of Pharmacy, University of Porto, Porto, Portugal; Institute for Research and Innovation in Health, University of Porto, Porto, Portugal), **Ivan J Vechetti** (University of Nebraska‐Lincoln, Lincoln, NE, USA), **Sara I Veiga** (Krantz Family Center for Cancer Research, Massachusetts General Hospital, Boston, MA, USA; Department of Medicine, Harvard Medical School, Boston, MA, USA), **Laura J Vella** (The Florey Institute of Neuroscience and Mental Health, The University of Melbourne, Parkville, Australia; Department of Surgery, The Royal Melbourne Hospital, Melbourne, Australia; The University of Melbourne, Parkville, Australia), **Émilie Velot** (French National Center for Scientific Research, Molecular Engineering and Physiopathology, University of Lorraine, Nancy, France), **Frederik J Verweij** (Department of Cell Biology, Neurobiology and Biophysics, Utrecht University, Utrecht, The Netherlands; Centre for Living Technologies, Alliance Eindhoven University of Technology, Wageningen University & Research, University Medical Center Utrecht, The Netherlands), **Beate Vestad** (Research Institute of Internal Medicine, Oslo University Hospital Rikshospitalet, Oslo, Norway; Norwegian PSC Research Center, Oslo University Hospital Rikshospitalet, Oslo, Norway), **Ludovic Vinay** (Faculty of Medicine, Université Laval, Quebec, Canada; CHU de Quebec Research Center, Quebec, Canada), **Margarida Viola** (University Medical Center Utrecht, Utrecht, The Netherlands), **Tamás Visnovitz** (Department of Genetics, Cell‐ and Immunobiology, Semmelweis University, Budapest, Hungary; Department of Plant Physiology and Molecular Plant Biology, ELTE Eötvös Loránd University, Budapest, Hungary), **Dolores Di Vizio** (Department of Surgery, Division of Cancer Biology and Therapeutics, Cedars‐Sinai Medical Center, Los Angeles, CA, USA), **Wyatt N Vreeland** (National Institute of Standards & Technology, Gaithersburg, MD, USA), **Krisztina V Vukman** (Department of Genetics, Cell‐ and Immunobiology, Semmelweis University, Budapest, Hungary), **Philippa K Wade** (Centre for Biomolecular Sciences, Biodiscovery Institute 3, School of Medicine, University of Nottingham, Nottingham, UK), **Simonides I van de Wakker** (UMC Utrecht Regenerative Medicine Center, Circulatory Health Research Center, University Medical Center Utrecht, Utrecht, The Netherlands), **Lucas Walther** (Transgene SA, Illkirch‐Graffenstaden, France; INSERM UMR_S1109, Tumor Biomechanics, Strasbourg, France; University of Strasbourg, Strasbourg, France), **Tong Wang** (MOE Key Laboratory of Tumor Molecular Biology, Institute of Life and Health Engineering, College of Life Science and Technology, The First Affiliated Hospital, Jinan University, Guangzhou, China), **Xiaoqin Wang** (Advanced Drug Delivery, Pharmaceutical Sciences, Biopharmaceutics R&D, AstraZeneca, Gothenburg, Sweden), **Dionysios C Watson** (Sylvester Comprehensive Cancer Center, University of Miami, Miami, FL, USA), **Marca HM Wauben** (Department of Biomolecular Health Sciences, Faculty of Veterinary Medicine, Utrecht University, Utrecht, The Netherlands), **Alissa M Weaver** (Vanderbilt University School of Medicine, Nashville, TN, USA), **Jason P Webber** (Institute of Life Science, Swansea University Medical School, Swansea University, Swansea, UK), **Viktoria Weber** (Department for Biomedical Research, University for Continuing Education Krems, Krems, Austria), **Ann M Wehman** (University of Denver, Denver, USA), **Luisa Weiss** (School of Biomolecular and Biomedical Science, University College Dublin, Dublin, Ireland; Conway SPHERE Research Group, Conway Institute, University College Dublin, Dublin, Ireland), **Mark L Weiss** (Kansas State University, Manhattan, KS, USA), **René Weiss** (Center for Biomedical Technology, Department for Biomedical Research, University for Continuing Education Krems, Krems, Austria), **Ralph Weissleder** (Center for Systems Biology, Harvard Medical School, Boston, MA, USA; Interventional Radiology, Massachusetts General Hospital, Boston, MA, USA), **Joshua A Welsh** (Translational Nanobiology Section, Laboratory of Pathology, National Cancer Institute, National Institutes of Health, Bethesda, MD, USA), **Yi Wen** (Kansas State University College of Veterinary Medicine, Manhattan, KS, USA; Department of Molecular and Comparative Pathobiology, Johns Hopkins University School of Medicine, Baltimore, MD, USA), **Olivier de Wever** (Laboratory of Experimental Cancer Research, Department of Human Structure and Repair, Ghent University, Ghent, Belgium; Cancer Research Institute Ghent, Ghent, Belgium), **Asa M Wheelock** (Respiratory Medicine Unit, Department of Medicine Solna & Centre for Molecular Medicine, Karolinska Institutet, Stockholm, Sweden; Department of Respiratory Medicine and Allergy, Karolinska University Hospital Solna, Stockholm, Sweden), **Katherine E White** (University of Nottingham, Nottingham, UK), **Bradley Whitehead** (Department of Clinical Medicine, Aarhus University, Aarhus, Denmark), **Theresa L Whiteside** (University of Pittsburgh Medical Center, Pittsburgh, PA, USA), **Joseph Whitley** (Department of Pharmaceutical and Biomedical Sciences, University of Georgia, Athens, GA, USA), **Zoltán Wiener** (Department of Genetics, Cell‐ and Immunobiology, Semmelweis University, Budapest, Hungary), **Andre J van Wijnen** (Department of Biochemistry, University of Vermont, Burlington, VT, USA), **Oscar PB Wiklander** (Department of Laboratory Medicine, Karolinska Institutet, Stockholm, Sweden), **Sarah Williams** (International Society for Extracellular Vesicles), **Charisse N Winston** (Alzheimer's Therapeutic Research Institute, University of Southern California, Los Angeles, CA, USA), **Kenneth W Witwer** (Department of Molecular and Comparative Pathobiology, Johns Hopkins University School of Medicine, Baltimore, MD, USA; EV Core Facility “EXCEL”, Institute for Basic Biomedical Sciences, Johns Hopkins University School of Medicine, Baltimore, MD, USA; The Richman Family Precision Medicine Center of Excellence in Alzheimer's Disease, Johns Hopkins University School of Medicine, Baltimore, MD, USA), **Martin Wolf** (Cell Therapy Institute, Paracelsus Medical University, Salzburg, Austria), **Joy Wolfram** (School of Chemical Engineering, The University of Queensland, Brisbane, Australia; Australian Institute for Bioengineering and Nanotechnology, The University of Queensland, Brisbane, Australia; Department of Nanomedicine, Houston Methodist Research Institute, Houston, TX, USA), **Liang Wu** (Department of Nephrology, The First Affiliated Hospital of Shaoyang University, Shaoyang, Hunan, China; Erasmus MC Transplant Institute, University Medical Center Rotterdam, Department of Internal Medicine, Division of Nephrology and Transplantation, Rotterdam, The Netherlands), **Yunjie Wu** (Department of Pharmacology, University of Oslo, Oslo, Norway), **Magdalena E Wysmołek** (Division of Parasitology, Department of Preclinical Sciences, Institute of Veterinary Medicine, Warsaw University of Life Sciences‐SGGW, Warsaw, Poland; Institute of Specific Prophylaxis and Tropical Medicine, Center for Pathophysiology, Infectiology, and Immunology, Medical University of Vienna, Vienna, Austria), **Patricia Xander** (Instituto de Ciências Ambientais, Químicas e Farmacêuticas, Departamento de Ciências Farmacêuticas, Universidade Federal de São Paulo Campus Diadema, Diadema, Brazil; Instituto de Ciências Ambientais, Químicas e Farmacêuticas, Programa de Pós‐Graduação Biologia‐Química, Universidade Federal de São Paulo Campus Diadema, Diadema, Brazil), **Cristina PR Xavier** (Instituto de Investigação e Inovação em Saúde (i3S), University of Porto, Porto, Portugal; Cancer Drug Resistance Group, Institute of Molecular Pathology and Immunology (IPATIMUP), University of Porto, Porto, Portugal), **Yu Xiao** (School of Pharmaceutical Sciences, Tsinghua University, Beijing, China; Tsinghua University‐Peking University Joint Center for Life Sciences, Tsinghua University, Beijing, China; Beijing Advanced Innovation Center for Structural Biology, Tsinghua University, Beijing, China), **Rong Xu** (Australian Centre for Blood Diseases, Central Clinical School, Monash University, Melbourne, Australia; Victorian Heart Institute, Monash University, Melbourne, Australia), **María Yáñez‐Mó** (Dept Biología Molecular, Instituto Universitario de Biología Molecular, Universidad Autónoma de Madrid, Madrid, Spain; Centro de Biología Molecular Severo Ochoa, Instituto de Investigaciones Sanitarias Princesa, Madrid, Spain), **Tomofumi Yamamoto** (Tokyo Medical University, Tokyo, Japan; National Institute of Health Sciences, Kanagawa, Japan), **Yuki Yamamoto** (Department of Cellular and Molecular Biology, Graduate School of Biomedical and Health Science, Hiroshima University, Hiroshima, Japan), **Yusuke Yamamoto** (Laboratory of Integrative Oncology, National Cancer Center Research Institute, Tokyo, Japan), **Xiaomei Yan** (Department of Chemical Biology, Xiamen University, Xiamen, China), **Lifang Yang** (Eastern Virginia Medical School, Norfolk, VA, USA), **Yongkang Yang** (Institute for Cell Engineering, Johns Hopkins University School of Medicine, Baltimore, MD, USA; The Johns Hopkins University School of Medicine, Sidney Kimmel Comprehensive Cancer Center, Baltimore, MD, USA), **Reza Yarani** (Translational Type 1 Diabetes Research, Department of Clinical Research, Steno Diabetes Center Copenhagen, Herlev, Denmark), **Kyungmoo Yea** (Department of New Biology, DGIST, Daegu, Republic of Korea.; New Biology Research Center, DGIST, Daegu 43024, Republic of Korea.), **Laura Yedigaryan** (Translational Cardiomyology Laboratory, Stem Cell and Developmental Biology, Department of Development and Regeneration, KU Leuven, Leuven, Belgium), **Vengala Rao Yenuganti** (Department of Animal Biology, School of Life Sciences, University of Hyderabad, Hyderabad, India), **Saigopalakrishna S Yerneni** (Carnegie Mellon University, Pittsburgh, PA, USA), **Vincent Yeung** (Harvard Medical School, Boston, MA, USA; Schepens Eye Research Institute of Mass Eye and Ear, Boston, MA, USA), **Yagmur Yildizhan** (Rega Institute for Medical Research, KU Leuven, Leuven, Belgium), **Hang Yin** (School of Pharmaceutical Sciences, Tsinghua University, Beijing, China), **Akira Yokoi** (Nagoya University Graduate School of Medicine, Nagoya, Japan; Nagoya University Institute for Advanced Research, Nagoya, Japan), **Yusuke Yoshioka** (Department of Molecular and Cellular Medicine, Institute of Medical Science, Tokyo Medical University, Tokyo, Japan), **Yang You** (Department of Neuroscience, Mayo Clinic Florida, Jacksonville, FL, USA), **Ling‐Qing Yuan** (Department of Metabolism and Endocrinology, National Clinical Research Center for Metabolic Diseases, the Second Xiangya Hospital, Central South University, Changsha, China), **Samuel Tassi Yunga** (Cancer Early Detection Advanced Research Center (CEDAR), Knight Cancer Institute, School of Medicine, Oregon Health & Science University, Portland, OR, USA; Department of Biomedical Engineering, School of Medicine, Oregon Health & Science University, Portland, OR, USA), **Amin Zakeri** (Department of Clinical Medicine, Aarhus University, Aarhus, Denmark), **Augusto Zani** (Developmental and Stem Cell Biology Program, SickKids Research institute, Toronto, Canada; Division of General and Thoracic Surgery, Hospital for Sick Children, Toronto, Canada; Department of Surgery, University of Toronto, Toronto, Canada), **Michele Zanoni** (IRCCS Istituto Romagnolo per lo Studio dei Tumori (IRST) “Dino Amadori”, Meldola, Italy), **Valentina Zappulli** (Department of Comparative Biomedicine and Food Science, University of Padua, Padua, Italy), **Natasa Zarovni** (Day One Srl, Rome, Italy), **Jana Zarubova** (Department of Bioengineering, University of California, Los Angeles, Los Angeles, CA, USA), **Janos Zempleni** (University of Nebraska‐Lincoln, Lincoln, NE, USA), **Vytautas Žėkas** (Department of Physiology, Biochemistry, Microbiology and Laboratory Medicine, Institute of Biomedical Sciences, Faculty of Medicine, Vilnius University, Vilnius, Lithuania), **Andrea Zendrini** (Center for Colloid and Surface Science (CSGI), Florence, Italy; Department of Molecular and Translational Medicine, University of Brescia, Brescia, Italy), **Hao Zhang** (Institute of Precision Cancer Medicine and Pathology, School of Medicine, Jinan University, Guangzhou, China), **Qin Zhang** (Department of Medicine, Vanderbilt University Medical Center, Nashville, TN, USA), **Zheng Zhao** (Sartorius Stedim North America, Ann Arbor, MI, USA), **Lei Zheng** (Department of Laboratory Medicine, Nanfang Hospital, Southern Medical University, Guangzhou, China), **Yinghong Zhou** (School of Dentistry, The University of Queensland, Brisbane, Australia), **Antje M Zickler** (Department of Laboratory Medicine, Karolinska Institutet, Stockholm, Sweden; ME Cell Therapy and Allogenic Stem Cell Transplantation CAST, Karolinska University Hospital, Stockholm, Sweden), **Andries Zijlstra** (Department of Pathology, Vanderbilt University Medical Center, Nashville, TN, USA; Genentech, South San Francisco, CA, USA), **Alan J Zimmerman** (Barnett Institute of Chemical and Biological Analysis, Department of Chemistry and Chemical Biology, Northeastern University, Boston, MA, USA), **Pascale Zimmermann** (KU Leuven, Leuven, Belgium; Centre de Recherche en Cancérologie de Marseille, Merseille, France), **Angela M Zivkovic** (Department of Nutrition, University of California, Davis, Davis, CA, USA), **Davide Zocco** (Lonza Siena, Siena, Italy), **Ewa K Zuba‐Surma** (Department of Cell Biology, Faculty of Biochemistry, Biophysics and Biotechnology, Jagiellonian University, Krakow, Poland), **Haseeb Zubair** (Surgical Sciences Division, Department of Surgery, School of Medicine, University of Maryland, Baltimore, MD, USA; Program in Oncology, UM Greenebaum Comprehensive Cancer Center, Baltimore, MD, USA).

## CONFLICT OF INTEREST STATEMENT

Pierre Arsène is CEO of Mursla Ltd. and Chair of Exosla Ltd; Antonella Bongiovanni has filed the patent (PCT/EP2020/086622) related to microalgal‐derived extracellular vesicles and is co‐founder and CEO of the spin‐off company EVEBiofactory srl; Paul C Boutros sits on the scientific advisory boards of Sage Bionetworks, Intersect Diagnostics Inc and BioSymetrics Inc; Xandra O Breakefield is Scientific Advisor for Evox and MGB‐Cannon; Edit I Buzas is a member of the Scientific Advisory Boards of Sphere Gene Therapeutics Inc (Boston, MA, USA) and ReNeuron (UK); David RF Carter is an Evox Therapeutics Ltd, employee and stock option holder; Anna Cifuentes‐Rius was employed by Exopharm Ltd when the survey was conducted ACR is a shareholder of Exopharm Ltd; Rossella Crescitelli has developed multiple EV‐associated patents for putative clinical utilisation and they own equity in Exocure Sweden AB; Andrew Devitt is Chief Technical Officer, co‐founder, and director of EVolution Therapeutics; Erez Eitan works and has equity in NeuroDex, a company that develops EV‐based diagnostics; Samir EL Andaloussi is co‐founder of Evox Therapeutics; Ludwig Ermann Lundberg is an employee of BioGaia; Susanne Gabrielsson has a patent on B cell derived EVs in immune therapy and is part of the Scientific Advisory Board of Anjarium Biosciences; Ernesto Gargiulo is a medical writer at Novo Nordisk A/S; Bernd Giebel is a member of the Scientific Advisory Boards of Mursla Ltd, ReNeuron, and PLBioscience and is the founding director of Exosla Ltd; André Görgens is a consultant for and has equity interest in Evox Therapeutics (Oxford, UK) and is an inventor on several patent applications and patents related to EV isolation, modification, and analytics; Ahmed GE Ibrahim owns stock in Capricor Therapeutics; Marzena Kurzawa‐Akanbi Kurzawa‐Akanbi is an academic founder and Chief Scientific Officer at ESP Diagnostics Limited; Quentin Lubart is an employee of Abbelight (Cachan, France), which constructs and sells super‐resolution microscopes to characterize EVs; Fabrice Lucien receives consulting fees from Mursla Bio and Early is Good; Elisa Lázaro‐Ibáñez is employed by AstraZeneca R&D; Jan Lötvall is co‐founder of two companies aiming to develop EV‐based therapeutics, Exocure Sweden AB and Nexo Therapeutics AB, has been or is a scientific consultant for NanoSight, Clara Biotech and ExoCoBio, and was Editor‐in‐Chief of the Journal of Extracellular Vesicles during the development and publication of MISEV2023; Eduardo Marbán has founder's equity in Capricor Therapeutics Inc; Maurizio Muraca is a consultant for EXO Biologics (Liège, Belgium); Irina Nazarenko is a scientific adviser of CapCO Bio GmbH; D Michiel Pegtel has research funding from Takeda, Amgen, Abbvie, and Gilead, is an advisor of Y2Y BV, and has equity in Y2Y BV; Janusz Rak is inventor on a patent on oncogene‐carrying EVs that is licensed to NXPharmaGene; Gregory E Rice is Chief Scientific Officer, Inoviq Ltd; Andrew Rowland is a recipient of investigator‐initiated research funding outside of the scope of this publication from AstraZeneca, Boehringer Ingelheim, and Pfizer and is a recipient of speakers fees from Boehringer Ingelheim and Genentech; Susmita Sahoo performs research funded by Evox Therapeutics; Randy Schekman is a member of the Scientific Advisory Boards of companies involved in the analysis and diagnostic/therapeutic application of various forms of synthetic or native extracellular vesicles in diagnostics: Sail (formerly Senda) Biomedicines, Invaio Sciences, Mercy BioAnalytics, and Esperovax; Raymond M Schiffelers is CSO of Excytex bv; Johan Skog is an employee of Bio‐Techne and an inventor on patents for exosome isolation and analysis; Vera A Tang is a consultant for Beckman Coulter on small particle flow cytometry; Clotilde Théry is an inventor on a submitted patent on therapeutic use of EVs; Edwin van der Pol is cofounder and shareholder of Exometry, Amsterdam, The Netherlands; Joshua A Welsh is an inventor on patents and patent applications related to EV analysis; Oscar PB Wiklander has stock options with Evox Therapeutics; Kenneth W Witwer is or has been an advisory board member of ShiftBio, Exopharm, NeuroDex, NovaDip, and ReNeuron; holds NeuroDex options; privately consults as Kenneth Witwer Consulting; and conducts research under a sponsored research agreement with Ionis Pharmaceuticals.

## References

[jev212404-bib-0001] Aalberts, M. , van Dissel‐Emiliani, F. M. , van Adrichem, N. P. , van Wijnen, M. , Wauben, M. H. , Stout, T. A. , & Stoorvogel, W. (2012). Identification of distinct populations of prostasomes that differentially express prostate stem cell antigen, annexin A1, and GLIPR2 in humans. Biology of Reproduction, 86, 82. 22133690 10.1095/biolreprod.111.095760

[jev212404-bib-0002] Aasebø, E. , Opsahl, J. A. , Bjørlykke, Y. , Myhr, K. M. , Kroksveen, A. C. , & Berven, F. S. (2014). Effects of blood contamination and the rostro‐caudal gradient on the human cerebrospinal fluid proteome. PLoS ONE, 9, e90429. 24599184 10.1371/journal.pone.0090429 PMC3943968

[jev212404-bib-0003] Abbatiello, S. E. , Mani, D. R. , Schilling, B. , Maclean, B. , Zimmerman, L. J. , Feng, X. , Cusack, M. P. , Sedransk, N. , Hall, S. C. , Addona, T. , Allen, S. , Dodder, N. G. , Ghosh, M. , Held, J. M. , Hedrick, V. , Inerowicz, H. D. , Jackson, A. , Keshishian, H. , Kim, J. W. , … Carr, S. A. (2013). Design, implementation and multisite evaluation of a system suitability protocol for the quantitative assessment of instrument performance in liquid chromatography‐multiple reaction monitoring‐MS (LC‐MRM‐MS). Molecular & Cellular Proteomics, 12, 2623–2639. 23689285 10.1074/mcp.M112.027078 PMC3769335

[jev212404-bib-0004] Aebersold, R. , & Mann, M. (2003). Mass spectrometry‐based proteomics. Nature, 422, 198–207. 12634793 10.1038/nature01511

[jev212404-bib-0005] Aguet, F. , Antonescu, C. N. , Mettlen, M. , Schmid, S. L. , & Danuser, G. (2013). Advances in analysis of low signal‐to‐noise images link dynamin and AP2 to the functions of an endocytic checkpoint. Developmental Cell, 26, 279–291. 23891661 10.1016/j.devcel.2013.06.019 PMC3939604

[jev212404-bib-0006] Ainsztein, A. M. , Brooks, P. J. , Dugan, V. G. , Ganguly, A. , Guo, M. , Howcroft, T. K. , Kelley, C. A. , Kuo, L. S. , Labosky, P. A. , Lenzi, R. , McKie, G. A. , Mohla, S. , Procaccini, D. , Reilly, M. , Satterlee, J. S. , Srinivas, P. R. , Church, E. S. , Sutherland, M. , Tagle, D. A. , … Venkatachalam, S. (2015). The NIH extracellular RNA communication consortium. Journal of Extracellular Vesicles, 4, 27493. 26320938 10.3402/jev.v4.27493 PMC4553264

[jev212404-bib-0007] Alexander, M. , Hu, R. , Runtsch, M. C. , Kagele, D. A. , Mosbruger, T. L. , Tolmachova, T. , Seabra, M. C. , Round, J. L. , Ward, D. M. , & O'Connell, R. M. (2015). Exosome‐delivered microRNAs modulate the inflammatory response to endotoxin. Nature Communications, 6, 7321. 10.1038/ncomms8321 PMC4557301 26084661

[jev212404-bib-0008] Ambrose, A. R. , Dechantsreiter, S. , Shah, R. , Montero, M. A. , Quinn, A. M. , Hessel, E. M. , Beinke, S. , Tannahill, G. M. , & Davis, D. M. (2020). Corrected super‐resolution microscopy enables nanoscale imaging of autofluorescent lung macrophages. Biophysical Journal, 119, 2403–2417. 33217385 10.1016/j.bpj.2020.10.041 PMC7822748

[jev212404-bib-0009] Aps, J. K. , & Martens, L. C. (2005). Review: The physiology of saliva and transfer of drugs into saliva. Forensic Science International, 150, 119–131. 15944052 10.1016/j.forsciint.2004.10.026

[jev212404-bib-0010] Arab, T. , Mallick, E. R. , Huang, Y. , Dong, L. , Liao, Z. , Zhao, Z. , Gololobova, O. , Smith, B. , Haughey, N. J. , Pienta, K. J. , Slusher, B. S. , Tarwater, P. M. , Tosar, J. P. , Zivkovic, A. M. , Vreeland, W. N. , Paulaitis, M. E. , & Witwer, K. W. (2021). Characterization of extracellular vesicles and synthetic nanoparticles with four orthogonal single‐particle analysis platforms. Journal of Extracellular Vesicles, 10, e12079. 33850608 10.1002/jev2.12079 PMC8023330

[jev212404-bib-0011] Arifin, D. R. , Witwer, K. W. , & Bulte, J. W. M. (2022). Non‐Invasive imaging of extracellular vesicles: Quo vaditis in vivo? Journal of Extracellular Vesicles, 11, e12241. 35844061 10.1002/jev2.12241 PMC9289215

[jev212404-bib-0012] Arigony, A. L. , de Oliveira, I. M. , Machado, M. , Bordin, D. L. , Bergter, L. , Prá, D. , & Henriques, J. A. (2013). The influence of micronutrients in cell culture: A reflection on viability and genomic stability. BioMed Research International, 2013, 597282. 23781504 10.1155/2013/597282 PMC3678455

[jev212404-bib-0013] Arraud, N. , Linares, R. , Tan, S. , Gounou, C. , Pasquet, J. M. , Mornet, S. , & Brisson, A. R. (2014). Extracellular vesicles from blood plasma: Determination of their morphology, size, phenotype and concentration. Journal of Thrombosis and Haemostasis, 12, 614–627. 24618123 10.1111/jth.12554

[jev212404-bib-0014] Avalos‐Padilla, Y. , Georgiev, V. N. , Lantero, E. , Pujals, S. , Verhoef, R. , Borgheti‐Cardoso L, N. , Albertazzi, L. , Dimova, R. , & Fernàndez‐Busquets, X. (2021). The ESCRT‐III machinery participates in the production of extracellular vesicles and protein export during *Plasmodium falciparum* infection. Plos Pathogens, 17, e1009455. 33798247 10.1371/journal.ppat.1009455 PMC9159051

[jev212404-bib-0015] Bachurski, D. , Schuldner, M. , Nguyen, P. H. , Malz, A. , Reiners, K. S. , Grenzi, P. C. , Babatz, F. , Schauss, A. C. , Hansen, H. P. , Hallek, M. , & Pogge von Strandmann, E. (2019). Extracellular vesicle measurements with nanoparticle tracking analysis – An accuracy and repeatability comparison between NanoSight NS300 and ZetaView. Journal of Extracellular Vesicles, 8, 1596016. 30988894 10.1080/20013078.2019.1596016 PMC6450530

[jev212404-bib-0016] Bai, L. , Du, Y. , Peng, J. , Liu, Y. , Wang, Y. , Yang, Y. , & Wang, C. (2014). Peptide‐based isolation of circulating tumor cells by magnetic nanoparticles. Journal of Materials Chemistry B, 2, 4080–4088. 32261739 10.1039/c4tb00456f

[jev212404-bib-0017] Balaj, L. , Atai, N. A. , Chen, W. , Mu, D. , Tannous, B. A. , Breakefield, X. O. , Skog, J. , & Maguire, C. A. (2015). Heparin affinity purification of extracellular vesicles. Scientific Reports, 5, 10266. 25988257 10.1038/srep10266 PMC4437317

[jev212404-bib-0018] Balaj, L. , Lessard, R. , Dai, L. , Cho, Y. J. , Pomeroy, S. L. , Breakefield, X. O. , & Skog, J. (2011). Tumour microvesicles contain retrotransposon elements and amplified oncogene sequences. Nature Communications, 2, 180. 10.1038/ncomms1180 PMC3040683 21285958

[jev212404-bib-0019] Baldrich, P. , Rutter, B. D. , Karimi, H. Z. , Podicheti, R. , Meyers, B. C. , & Innes, R. W. (2019). Plant extracellular vesicles contain diverse small RNA species and are enriched in 10‐ to 17‐nucleotide “Tiny” RNAs. Plant Cell, 31, 315–324. 30705133 10.1105/tpc.18.00872 PMC6447009

[jev212404-bib-0020] Ballard, O. , & Morrow, A. L. (2013). Human milk composition: Nutrients and bioactive factors. Pediatric Clinics of North America, 60, 49–74. 23178060 10.1016/j.pcl.2012.10.002 PMC3586783

[jev212404-bib-0021] Banigan, M. G. , Kao, P. F. , Kozubek, J. A. , Winslow, A. R. , Medina, J. , Costa, J. , Schmitt, A. , Schneider, A. , Cabral, H. , Cagsal‐Getkin, O. , Vanderburg, C. R. , & Delalle, I. (2013). Differential expression of exosomal microRNAs in prefrontal cortices of schizophrenia and bipolar disorder patients. PLoS ONE, 8, e48814. 23382797 10.1371/journal.pone.0048814 PMC3559697

[jev212404-bib-0022] Barreiro, K. , Dwivedi, O. P. , Valkonen, S. , Groop, P. H. , Tuomi, T. , Holthofer, H. , Rannikko, A. , Yliperttula, M. , Siljander, P. , Laitinen, S. , Serkkola, E. , Af Hallstrom, T. , Forsblom, C. , Groop, L. , & Puhka, M. (2021). Urinary extracellular vesicles: Assessment of pre‐analytical variables and development of a quality control with focus on transcriptomic biomarker research. Journal of Extracellular Vesicles, 10, e12158. 34651466 10.1002/jev2.12158 PMC8517090

[jev212404-bib-0023] Beale, D. J. , Jones, O. A. , Karpe, A. V. , Dayalan, S. , Oh, D. Y. , Kouremenos, K. A. , Ahmed, W. , & Palombo, E. A. (2016). A review of analytical techniques and their application in disease diagnosis in breathomics and salivaomics research. International Journal of Molecular Sciences, 18, 24. 28025547 10.3390/ijms18010024 PMC5297659

[jev212404-bib-0024] Beckett, K. , Monier, S. , Palmer, L. , Alexandre, C. , Green, H. , Bonneil, E. , Raposo, G. , Thibault, P. , Borgne, R. L. , & Vincent, J. P. (2013). Drosophila S2 cells secrete wingless on exosome‐like vesicles but the wingless gradient forms independently of exosomes. Traffic (Copenhagen, Denmark), 14, 82–96. 23035643 10.1111/tra.12016 PMC4337976

[jev212404-bib-0025] Beer, K. B. , Rivas‐Castillo, J. , Kuhn, K. , Fazeli, G. , Karmann, B. , Nance, J. F. , Stigloher, C. , & Wehman, A. M. (2018). Extracellular vesicle budding is inhibited by redundant regulators of TAT‐5 flippase localization and phospholipid asymmetry. PNAS, 115, E1127–E1136. 29367422 10.1073/pnas.1714085115 PMC5819400

[jev212404-bib-0026] Beer, K. B. , & Wehman, A. M. (2017). Mechanisms and functions of extracellular vesicle release in vivo‐What we can learn from flies and worms. Cell Adhesion & Migration, 11, 135–150. 27689411 10.1080/19336918.2016.1236899 PMC5351733

[jev212404-bib-0027] Benedikter, B. J. , Bouwman, F. G. , Vajen, T. , Heinzmann, A. C. A. , Grauls, G. , Mariman, E. C. , Wouters, E. F. M. , Savelkoul, P. H. , Lopez‐Iglesias, C. , Koenen, R. R. , Rohde, G. G. U. , & Stassen, F. R. M. (2017). Ultrafiltration combined with size exclusion chromatography efficiently isolates extracellular vesicles from cell culture media for compositional and functional studies. Scientific Reports, 7, 15297. 29127410 10.1038/s41598-017-15717-7 PMC5681555

[jev212404-bib-0028] Benmoussa, A. , Michel, S. , Gilbert, C. , & Provost, P. (2020). Isolating multiple extracellular vesicles subsets, including exosomes and membrane vesicles, from bovine milk using sodium citrate and differential ultracentrifugation. Bio‐Protocol, 10, e3636. 33659307 10.21769/BioProtoc.3636 PMC7842771

[jev212404-bib-0029] Bereman, M. S. (2015). Tools for monitoring system suitability in LC MS/MS centric proteomic experiments. Proteomics, 15, 891–902. 25327420 10.1002/pmic.201400373

[jev212404-bib-0030] Berne, B. J. , & Pecora, R. (1976). Dynamic light scattering: With applications to chemistry, biology, and physics . Wiley.

[jev212404-bib-0031] Bettin, B. , Gasecka, A. , Li, B. , Dhondt, B. , Hendrix, A. , Nieuwland, R. , & van der Pol, E. (2022). Removal of platelets from blood plasma to improve the quality of extracellular vesicle research. Journal of Thrombosis and Haemostasis, 20, 2679–2685. 36043239 10.1111/jth.15867 PMC9825910

[jev212404-bib-0032] Betzig, E. , Patterson, G. H. , Sougrat, R. , Lindwasser, O. W. , Olenych, S. , Bonifacino, J. S. , Davidson, M. W. , Lippincott‐Schwartz, J. , & Hess, H. F. (2006). Imaging intracellular fluorescent proteins at nanometer resolution. Science, 313, 1642–1645. 16902090 10.1126/science.1127344

[jev212404-bib-0033] Bhattarai, K. R. , Kim, H. R. , & Chae, H. J. (2018). Compliance with saliva collection protocol in healthy volunteers: strategies for managing risk and errors. International journal of Medical Sciences, 15, 823–831. 30008593 10.7150/ijms.25146 PMC6036086

[jev212404-bib-0034] Bitto, N. J. , Chapman, R. , Pidot, S. , Costin, A. , Lo, C. , Choi, J. , D'Cruze, T. , Reynolds, E. C. , Dashper, S. G. , Turnbull, L. , Whitchurch, C. B. , Stinear, T. P. , Stacey, K. J. , & Ferrero, R. L. (2017). Bacterial membrane vesicles transport their DNA cargo into host cells. Scientific Reports, 7, 7072. 28765539 10.1038/s41598-017-07288-4 PMC5539193

[jev212404-bib-0035] Bitto, N. J. , Cheng, L. , Johnston, E. L. , Pathirana, R. , Phan, T. K. , Poon, I. K. H. , O'Brien‐Simpson, N. M. , Hill, A. F. , Stinear, T. P. , & Kaparakis‐Liaskos, M. (2021). Staphylococcus aureus membrane vesicles contain immunostimulatory DNA, RNA and peptidoglycan that activate innate immune receptors and induce autophagy. Journal of Extracellular Vesicles, 10, e12080. 33815695 10.1002/jev2.12080 PMC8015888

[jev212404-bib-0036] Bitto, N. J. , & Kaparakis‐Liaskos, M. (2022). Methods of bacterial membrane vesicle production, purification, quantification, and examination of their immunogenic functions. Methods in Molecular Biology, 2523, 43–61. 35759190 10.1007/978-1-0716-2449-4_4

[jev212404-bib-0037] Bitto, N. J. , Zavan, L. , Johnston, E. L. , Stinear, T. P. , Hill, A. F. , & Kaparakis‐Liaskos, M. (2021). Considerations for the analysis of bacterial membrane vesicles: Methods of vesicle production and quantification can influence biological and experimental outcomes. Microbiology Spectrum, 9, e0127321. 34937167 10.1128/Spectrum.01273-21 PMC8694105

[jev212404-bib-0038] Blijdorp, C. J. , Tutakhel, O. A. Z. , Hartjes, T. A. , van den Bosch, T. P. P. , van Heugten, M. H. , Rigalli, J. P. , Willemsen, R. , Musterd‐Bhaggoe, U. M. , Barros, E. R. , Carles‐Fontana, R. , Carvajal, C. A. , Arntz, O. J. , van de Loo, F. A. J. , Jenster, G. , Clahsen‐van Groningen, M. C. , Cuevas, C. A. , Severs, D. , Fenton, R. A. , van Royen, M. E. , … Hoorn, E. J. (2021). Comparing approaches to normalize, quantify, and characterize urinary extracellular vesicles. Journal of the American Society of Nephrology, 32, 1210–1226. 33782168 10.1681/ASN.2020081142 PMC8259679

[jev212404-bib-0039] Boere, J. , van de Lest, C. H. A. , de Grauw, J. C. , Plomp, S. G. M. , Libregts, S. , Arkesteijn, G. J. A. , Malda, J. , Wauben, M. H. M. , & van Weeren, P. R. (2019). Extracellular vesicles in synovial fluid from juvenile horses: No age‐related changes in the quantitative profile. Veterinary Journal, 244, 91–93. 30825901 10.1016/j.tvjl.2018.12.010 PMC7116028

[jev212404-bib-0040] Boing, A. N. , van der Pol, E. , Grootemaat, A. E. , Coumans, F. A. , Sturk, A. , & Nieuwland, R. (2014). Single‐step isolation of extracellular vesicles by size‐exclusion chromatography. Journal of Extracellular Vesicles, 3, 00–00. 10.3402/jev.v3.23430 PMC4159761 25279113

[jev212404-bib-0041] Bonsergent, E. , Grisard, E. , Buchrieser, J. , Schwartz, O. , Théry, C. , & Lavieu, G. (2021). Quantitative characterization of extracellular vesicle uptake and content delivery within mammalian cells. Nature Communications, 12, 1864. 10.1038/s41467-021-22126-y PMC7994380 33767144

[jev212404-bib-0042] Bonsergent, E. , & Lavieu, G. (2019). Content release of extracellular vesicles in a cell‐free extract. Febs Letters, 593, 1983–1992. 31175663 10.1002/1873-3468.13472

[jev212404-bib-0043] Bordanaba‐Florit, G. , Royo, F. , Kruglik, S. G. , & Falcón‐Pérez, J. M. (2021). Using single‐vesicle technologies to unravel the heterogeneity of extracellular vesicles. Nature Protocols, 16, 3163–3185. 34135505 10.1038/s41596-021-00551-z

[jev212404-bib-0044] Borghesan, M. , Fafián‐Labora, J. , Eleftheriadou, O. , Carpintero‐Fernández, P. , Paez‐Ribes, M. , Vizcay‐Barrena, G. , Swisa, A. , Kolodkin‐Gal, D. , Ximénez‐Embún, P. , Lowe, R. , Martín‐Martín, B. , Peinado, H. , Muñoz, J. , Fleck, R. A. , Dor, Y. , Ben‐Porath, I. , Vossenkamper, A. , Muñoz‐Espin, D. , & O'Loghlen, A. (2019). Small extracellular vesicles are key regulators of non‐cell autonomous intercellular communication in senescence via the interferon protein IFITM3. Cell Reports, 27, 3956–3971.e6. 31242426 10.1016/j.celrep.2019.05.095 PMC6613042

[jev212404-bib-0045] Bortot, B. , Apollonio, M. , Rampazzo, E. , Valle, F. , Brucale, M. , Ridolfi, A. , Ura, B. , Addobbati, R. , Di Lorenzo, G. , Romano, F. , Buonomo, F. , Ripepi, C. , Ricci, G. , & Biffi, S. (2021). Small extracellular vesicles from malignant ascites of patients with advanced ovarian cancer provide insights into the dynamics of the extracellular matrix. Molecular Oncology, 15, 3596–3614. 34614287 10.1002/1878-0261.13110 PMC8637559

[jev212404-bib-0046] Bose, S. , Aggarwal, S. , Singh, D. V. , & Acharya, N. (2020). Extracellular vesicles: An emerging platform in gram‐positive bacteria. Microbial Cell, 7, 312–322. 33335921 10.15698/mic2020.12.737 PMC7713254

[jev212404-bib-0047] Botha, J. , Handberg, A. , & Simonsen, J. B. (2022). Lipid‐based strategies used to identify extracellular vesicles in flow cytometry can be confounded by lipoproteins: Evaluations of annexin V, lactadherin, and detergent lysis. Journal of Extracellular Vesicles, 11, e12200. 35362259 10.1002/jev2.12200 PMC8971177

[jev212404-bib-0048] Bracht, J. W. P. , Los, M. , van Eijndhoven, M. A. J. , Bettin, B. , van der Pol, E. , Pegtel, D. M. , & Nieuwland, R. (2023). Platelet removal from human blood plasma improves detection of extracellular vesicle‐associated miRNA. Journal of Extracellular Vesicles, 12, e12302. 36788785 10.1002/jev2.12302 PMC9929339

[jev212404-bib-0049] Brown, L. , Wolf, J. M. , Prados‐Rosales, R. , & Casadevall, A. (2015). Through the wall: Extracellular vesicles in Gram‐positive bacteria, mycobacteria and fungi. Nature Reviews Microbiology, 13, 620–630. 26324094 10.1038/nrmicro3480 PMC4860279

[jev212404-bib-0050] Budnik, V. , Ruiz‐Cañada, C. , & Wendler, F. (2016). Extracellular vesicles round off communication in the nervous system. Nature Reviews Neuroscience, 17, 160–172. 26891626 10.1038/nrn.2015.29 PMC4989863

[jev212404-bib-0051] Buntsma, N. C. , Gąsecka, A. , Roos, Y. , van Leeuwen, T. G. , van der Pol, E. , & Nieuwland, R. (2022). EDTA stabilizes the concentration of platelet‐derived extracellular vesicles during blood collection and handling. Platelets, 33, 764–771. 34697987 10.1080/09537104.2021.1991569

[jev212404-bib-0052] Burger, D. , Thibodeau, J. F. , Holterman, C. E. , Burns, K. D. , Touyz, R. M. , & Kennedy, C. R. (2014). Urinary podocyte microparticles identify prealbuminuric diabetic glomerular injury. Journal of the American Society of Nephrology, 25, 1401–1407. 24676640 10.1681/ASN.2013070763 PMC4073432

[jev212404-bib-0053] Busatto, S. , Vilanilam, G. , Ticer, T. , Lin, W. L. , Dickson, D. W. , Shapiro, S. , Bergese, P. , & Wolfram, J. (2018). Tangential flow filtration for highly efficient concentration of extracellular vesicles from large volumes of fluid. Cells, 7(12), 273. 30558352 10.3390/cells7120273 PMC6315734

[jev212404-bib-0054] Busatto, S. , Yang, Y. , Iannotta, D. , Davidovich, I. , Talmon, Y. , & Wolfram, J. (2022). Considerations for extracellular vesicle and lipoprotein interactions in cell culture assays. Journal of Extracellular Vesicles, 11, e12202. 35362268 10.1002/jev2.12202 PMC8971175

[jev212404-bib-0055] Busatto, S. , Zendrini, A. , Radeghieri, A. , Paolini, L. , Romano, M. , Presta, M. , & Bergese, P. (2019). The nanostructured secretome. Biomaterials Science, 8, 39–63. 31799977 10.1039/c9bm01007f

[jev212404-bib-0056] Bustin, S. A. , Benes, V. , Garson, J. A. , Hellemans, J. , Huggett, J. , Kubista, M. , Mueller, R. , Nolan, T. , Pfaffl, M. W. , Shipley, G. L. , Vandesompele, J. , & Wittwer, C. T. (2009). The MIQE Guidelines: Minimum information for publication of quantitative real‐time PCR experiments. Clinical Chemistry, 55, 611–622. 19246619 10.1373/clinchem.2008.112797

[jev212404-bib-0057] Butler, H. J. , Ashton, L. , Bird, B. , Cinque, G. , Curtis, K. , Dorney, J. , Esmonde‐White, K. , Fullwood, N. J. , Gardner, B. , Martin‐Hirsch, P. L. , Walsh, M. J. , McAinsh, M. R. , Stone, N. , & Martin, F. L. (2016). Using Raman spectroscopy to characterize biological materials. Nature Protocols, 11, 664–687. 26963630 10.1038/nprot.2016.036

[jev212404-bib-0058] Buzas, E. I. (2022). Opportunities and challenges in studying the extracellular vesicle corona. Nature Cell Biology, 24, 1322–1325. 36042293 10.1038/s41556-022-00983-z

[jev212404-bib-0059] Cai, J. , Han, Y. , Ren, H. , Chen, C. , He, D. , Zhou, L. , Eisner, G. M. , Asico, L. D. , Jose, P. A. , & Zeng, C. (2013). Extracellular vesicle‐mediated transfer of donor genomic DNA to recipient cells is a novel mechanism for genetic influence between cells. Journal of Molecular Cell Biology, 5, 227–238. 23580760 10.1093/jmcb/mjt011 PMC3733418

[jev212404-bib-0060] Calò, A. , Reguera, D. , Oncins, G. , Persuy, M. A. , Sanz, G. , Lobasso, S. , Corcelli, A. , Pajot‐Augy, E. , & Gomila, G. (2014). Force measurements on natural membrane nanovesicles reveal a composition‐independent, high Young's modulus. Nanoscale, 6, 2275–2285. 24407152 10.1039/c3nr05107b

[jev212404-bib-0061] Cambier, L. , Stachelek, K. , Triska, M. , Jubran, R. , Huang, M. , Li, W. , Zhang, J. , Li, J. , & Cobrinik, D. (2021). Extracellular vesicle‐associated repetitive element DNAs as candidate osteosarcoma biomarkers. Scientific Reports, 11, 94. 33420117 10.1038/s41598-020-77398-z PMC7794510

[jev212404-bib-0062] Cameron, J. M. , Butler, H. J. , Palmer, D. S. , & Baker, M. J. (2018). Biofluid spectroscopic disease diagnostics: A review on the processes and spectral impact of drying. Journal of Biophotonics, 11, e201700299. 29377638 10.1002/jbio.201700299

[jev212404-bib-0063] Cameron, S. , Gillio‐Meina, C. , Ranger, A. , Choong, K. , & Fraser, D. D. (2019). Collection and analyses of cerebrospinal fluid for pediatric translational research. Pediatric Neurology, 98, 3–17. 31280949 10.1016/j.pediatrneurol.2019.05.011

[jev212404-bib-0064] Carlomagno, C. , Giannasi, C. , Niada, S. , Bedoni, M. , Gualerzi, A. , & Brini, A. T. (2021). Raman fingerprint of extracellular vesicles and conditioned media for the reproducibility assessment of cell‐free therapeutics. Frontiers in Bioengineering and Biotechnology, 9, 640617. 33928071 10.3389/fbioe.2021.640617 PMC8076682

[jev212404-bib-0065] Carrel, A. , & Burrows, M. T. (1911). Cultivation of tissues in vitro and its technique. Journal of Experimental Medicine, 13, 387–396. 19867420 10.1084/jem.13.3.387 PMC2125263

[jev212404-bib-0066] Carreras‐Planella, L. , Cucchiari, D. , Cañas, L. , Juega, J. , Franquesa, M. , Bonet, J. , Revuelta, I. , Diekmann, F. , Taco, O. , Lauzurica, R. , & Borràs, F. E. (2021). Urinary vitronectin identifies patients with high levels of fibrosis in kidney grafts. Journal of Nephrology, 34, 861–874. 33275196 10.1007/s40620-020-00886-y PMC8192319

[jev212404-bib-0067] Catalano, M. , & O'Driscoll, L. (2020). Inhibiting extracellular vesicles formation and release: A review of EV inhibitors. Journal of Extracellular Vesicles, 9, 1703244. 32002167 10.1080/20013078.2019.1703244 PMC6968539

[jev212404-bib-0068] Cavallaro, S. , Pevere, F. , Stridfeldt, F. , Görgens, A. , Paba, C. , Sahu, S. S. , Mamand, D. R. , Gupta, D. , El Andaloussi, S. , Linnros, J. , & Dev, A. (2021). Multiparametric profiling of single nanoscale extracellular vesicles by combined atomic force and fluorescence microscopy: Correlation and heterogeneity in their molecular and biophysical features. Small, 17, e2008155. 33682363 10.1002/smll.202008155

[jev212404-bib-0069] Cavallaro, S. , Hååg, P. , Viktorsson, K. , Krozer, A. , Fogel, K. , Lewensohn, R. , Linnros, J. , & Dev, A. (2021). Comparison and optimization of nanoscale extracellular vesicle imaging by scanning electron microscopy for accurate size‐based profiling and morphological analysis. Nanoscale Advances, 3(11), 3053–3063. 36133670 10.1039/d0na00948b PMC9419097

[jev212404-bib-0070] Champagne‐Jorgensen, K. , Mian, M. F. , McVey Neufeld, K. A. , Stanisz, A. M. , & Bienenstock, J. (2021). Membrane vesicles of *Lacticaseibacillus rhamnosus* JB‐1 contain immunomodulatory lipoteichoic acid and are endocytosed by intestinal epithelial cells. Scientific Reports, 11, 13756. 34215822 10.1038/s41598-021-93311-8 PMC8253831

[jev212404-bib-0071] Chen, C. , Zong, S. , Wang, Z. , Lu, J. , Zhu, D. , Zhang, Y. , & Cui, Y. (2016). Imaging and intracellular tracking of cancer‐derived exosomes using single‐molecule localization‐based super‐resolution microscope. ACS Applied Materials & Interfaces, 8, 25825–25833. 27617891 10.1021/acsami.6b09442

[jev212404-bib-0072] Chen, C. , Zong, S. , Wang, Z. , Lu, J. , Zhu, D. , Zhang, Y. , Zhang, R. , & Cui, Y. (2018). Visualization and intracellular dynamic tracking of exosomes and exosomal miRNAs using single molecule localization microscopy. Nanoscale, 10, 5154–5162. 29492481 10.1039/c7nr08800k

[jev212404-bib-0073] Chernyshev, V. S. , Rachamadugu, R. , Tseng, Y. H. , Belnap, D. M. , Jia, Y. , Branch, K. J. , Butterfield, A. E. , Pease, L. F. , Bernard, P. S. , & Skliar, M. (2015). Size and shape characterization of hydrated and desiccated exosomes. Analytical and Bioanalytical Chemistry, 407, 3285–3301. 25821114 10.1007/s00216-015-8535-3

[jev212404-bib-0074] Chiappin, S. , Antonelli, G. , Gatti, R. , & De Palo, E. F. (2007). Saliva specimen: A new laboratory tool for diagnostic and basic investigation. Clinica Chimica Acta, 383, 30–40. 10.1016/j.cca.2007.04.011 17512510

[jev212404-bib-0075] Christianson, H. C. , Svensson, K. J. , van Kuppevelt, T. H. , Li, J. P. , & Belting, M. (2013). Cancer cell exosomes depend on cell‐surface heparan sulfate proteoglycans for their internalization and functional activity. PNAS, 110, 17380–17385. 24101524 10.1073/pnas.1304266110 PMC3808637

[jev212404-bib-0076] Chuo, S. T. , Chien, J. C. , & Lai, C. P. (2018). Imaging extracellular vesicles: Current and emerging methods. Journal of Biomedical Science, 25, 91. 30580764 10.1186/s12929-018-0494-5 PMC6304785

[jev212404-bib-0077] Churchman, L. S , & Spudich, J. A. (2012). Colocalization of fluorescent probes: Accurate and precise registration with nanometer resolution. Cold Spring Harbor Protocols, 2012, 141–149. 22301660 10.1101/pdb.top067918 PMC4799658

[jev212404-bib-0078] Cianciaruso, C. , Beltraminelli, T. , Duval, F. , Nassiri, S. , Hamelin, R. , Mozes, A. , Gallart‐Ayala, H. , Ceada Torres, G. , Torchia, B. , Ries, C. H. , Ivanisevic, J. , & De Palma, M. (2019). Molecular profiling and functional analysis of macrophage‐derived tumor extracellular vesicles. Cell Reports, 27, 3062–3080.e11. 31167148 10.1016/j.celrep.2019.05.008 PMC6581796

[jev212404-bib-0079] Cimorelli, M. , Nieuwland, R. , Varga, Z. , & van der Pol, E. (2021). Standardized procedure to measure the size distribution of extracellular vesicles together with other particles in biofluids with microfluidic resistive pulse sensing. PLoS ONE, 16, e0249603. 33793681 10.1371/journal.pone.0249603 PMC8016234

[jev212404-bib-0080] Ciullo, A. , Li, C. , Li, L. , Ungerleider, K. C. , Peck, K. , Marbán, E. , & Ibrahim, A. G. E. (2022). Biodistribution of unmodified cardiosphere‐derived cell extracellular vesicles using single RNA tracing. Journal of Extracellular Vesicles, 11, e12178. 35005847 10.1002/jev2.12178 PMC8743874

[jev212404-bib-0081] Clancy, J. W. , Sheehan, C. S. , Boomgarden, A. C. , & D'Souza‐Schorey, C. (2022). Recruitment of DNA to tumor‐derived microvesicles. Cell Reports, 38, 110443. 35235806 10.1016/j.celrep.2022.110443 PMC10351678

[jev212404-bib-0082] Clayton, A. , Boilard, E. , Buzas, E. I. , Cheng, L. , Falcón‐Perez, J. M. , Gardiner, C. , Gustafson, D. , Gualerzi, A. , Hendrix, A. , Hoffman, A. , Jones, J. , Lässer, C. , Lawson, C. , Lenassi, M. , Nazarenko, I. , O'Driscoll, L. , Pink, R. , Siljander, P. R. , Soekmadji, C. , … Nieuwland, R. (2019). Considerations towards a roadmap for collection, handling and storage of blood extracellular vesicles. Journal of Extracellular Vesicles, 8, 1647027. 31489143 10.1080/20013078.2019.1647027 PMC6711123

[jev212404-bib-0083] Clayton, A. , Buschmann, D. , Byrd, J. B. , Carter, D. R. F. , Cheng, L. , Compton, C. , Daaboul, G. , Devitt, A. , Falcon‐Perez, J. M. , Gardiner, C. , Gustafson, D. , Harrison, P. , Helmbrecht, C. , Hendrix, A. , Hill, A. , Hoffman, A. , Jones, J. C. , Kalluri, R. , Kang, J. Y. , … Nieuwland, R. (2018). Summary of the ISEV workshop on extracellular vesicles as disease biomarkers, held in Birmingham, UK, during December 2017. Journal of Extracellular Vesicles, 7, 1473707. 31162490 10.1080/20013078.2018.1473707 PMC5965025

[jev212404-bib-0084] Clupper, M. , Gill, R. , Elsayyid, M. , Touroutine, D. , Caplan, J. L. , & Tanis, J. E. (2022). Kinesin‐2 motors differentially impact biogenesis of extracellular vesicle subpopulations shed from sensory cilia. Iscience, 25, 105262. 36304122 10.1016/j.isci.2022.105262 PMC9593189

[jev212404-bib-0085] Coffman, V. C. , & Wu, J. Q. (2014). Every laboratory with a fluorescence microscope should consider counting molecules. Molecular Biology of the Cell, 25, 1545–1548. 24825827 10.1091/mbc.E13-05-0249 PMC4019486

[jev212404-bib-0086] Colombo, F. , Norton, E. G. , & Cocucci, E. (2021). Microscopy approaches to study extracellular vesicles. Biochimica et Biophysica Acta (BBA) ‐ General Subjects, 1865, 129752. 32991970 10.1016/j.bbagen.2020.129752

[jev212404-bib-0087] Correll, V. L. , Otto, J. J. , Risi, C. M. , Main, B. P. , Boutros, P. C. , Kislinger, T. , Galkin, V. E. , Nyalwidhe, J. O. , Semmes, O. J. , & Yang, L. (2022). Optimization of small extracellular vesicle isolation from expressed prostatic secretions in urine for in‐depth proteomic analysis. Journal of Extracellular Vesicles, 11, e12184. 35119778 10.1002/jev2.12184 PMC8815402

[jev212404-bib-0088] Corrigan, L. , Redhai, S. , Leiblich, A. , Fan, S. J. , Perera, S. M. , Patel, R. , Gandy, C. , Wainwright, S. M. , Morris, J. F. , Hamdy, F. , Goberdhan, D. C. , & Wilson, C. (2014). BMP‐regulated exosomes from Drosophila male reproductive glands reprogram female behavior. Journal of Cell Biology, 206, 671–688. 25154396 10.1083/jcb.201401072 PMC4151142

[jev212404-bib-0089] Corso, G. , Heusermann, W. , Trojer, D. , Görgens, A. , Steib, E. , Voshol, J. , Graff, A. , Genoud, C. , Lee, Y. , Hean, J. , Nordin, J. Z. , Wiklander, O. P. B. , El Andaloussi, S. , & Meisner‐Kober, N. (2019). Systematic characterization of extracellular vesicle sorting domains and quantification at the single molecule – single vesicle level by fluorescence correlation spectroscopy and single particle imaging. Journal of Extracellular Vesicles, 8, 1663043. 31579435 10.1080/20013078.2019.1663043 PMC6758720

[jev212404-bib-0090] Costa‐Silva, B. , Aiello, N. M. , Ocean, A. J. , Singh, S. , Zhang, H. , Thakur, B. K. , Becker, A. , Hoshino, A. , Mark, M. T. , Molina, H. , Xiang, J. , Zhang, T. , Theilen, T. M. , García‐Santos, G. , Williams, C. , Ararso, Y. , Huang, Y. , Rodrigues, G. , Shen, T. L. , … Lyden, D. (2015). Pancreatic cancer exosomes initiate pre‐metastatic niche formation in the liver. Nature Cell Biology, 17, 816–826. 25985394 10.1038/ncb3169 PMC5769922

[jev212404-bib-0091] Coumans, F. A. , van der Pol, E. , Boing, A. N. , Hajji, N. , Sturk, G. , van Leeuwen, T. G. , & Nieuwland, R. (2014). Reproducible extracellular vesicle size and concentration determination with tunable resistive pulse sensing. Journal of Extracellular Vesicles, 3, 25922. 25498889 10.3402/jev.v3.25922 PMC4263901

[jev212404-bib-0092] Coumans, F. A. W. , Brisson, A. R. , Buzas, E. I. , Dignat‐George, F. , Drees, E. E. E. , El‐Andaloussi, S. , Emanueli, C. , Gasecka, A. , Hendrix, A. , Hill, A. F. , Lacroix, R. , Lee, Y. , van Leeuwen, T. G. , Mackman, N. , Mager, I. , Nolan, J. P. , van der Pol, E. , Pegtel, D. M. , Sahoo, S. , … Nieuwland, R. (2017). Methodological guidelines to study extracellular vesicles. Circulation Research, 120, 1632–1648. 28495994 10.1161/CIRCRESAHA.117.309417

[jev212404-bib-0093] Crescitelli, R. , Lässer, C. , Jang, S. C. , Cvjetkovic, A. , Malmhäll, C. , Karimi, N. , Höög, J. L. , Johansson, I. , Fuchs, J. , Thorsell, A. , Gho, Y. S. , Bagge, R. O , & Lötvall, J. (2020). Subpopulations of extracellular vesicles from human metastatic melanoma tissue identified by quantitative proteomics after optimized isolation. Journal of Extracellular Vesicles, 9, 1722433. 32128073 10.1080/20013078.2020.1722433 PMC7034452

[jev212404-bib-0094] Crescitelli, R. , Lasser, C. , & Lotvall, J. (2021). Isolation and characterization of extracellular vesicle subpopulations from tissues. Nature Protocols, 16, 1548–1580. 33495626 10.1038/s41596-020-00466-1

[jev212404-bib-0095] Crescitelli, R. , Lässer, C. , Szabó, T. G. , Kittel, A. , Eldh, M. , Dianzani, I. , Buzás, E. I. , & Lötvall, J. (2013). Distinct RNA profiles in subpopulations of extracellular vesicles: Apoptotic bodies, microvesicles and exosomes. Journal of Extracellular Vesicles, 2, 20677. 10.3402/jev.v2i0.20677 PMC3823106 24223256

[jev212404-bib-0096] Cvjetkovic, A. , Jang, S. C. , Konečná, B. , Höög, J. L. , Sihlbom, C. , Lässer, C. , & Lötvall, J. (2016). Detailed analysis of protein topology of extracellular vesicles – Evidence of unconventional membrane protein orientation. Scientific Reports, 6, 36338. 27821849 10.1038/srep36338 PMC5099568

[jev212404-bib-0097] Czarniak, N. , Kamińska, J. , Matowicka‐Karna, J. , & Koper‐Lenkiewicz, O. M. (2023). Cerebrospinal fluid‐basic concepts review. Biomedicines, 11, 1461. 37239132 10.3390/biomedicines11051461 PMC10216641

[jev212404-bib-0098] Daaboul, G. G. , Gagni, P. , Benussi, L. , Bettotti, P. , Ciani, M. , Cretich, M. , Freedman, D. S. , Ghidoni, R. , Ozkumur, A. Y. , Piotto, C. , Prosperi, D. , Santini, B. , Unlu, M. S. , & Chiari, M. (2016). Digital detection of exosomes by interferometric imaging. Scientific Reports, 6, 37246. 27853258 10.1038/srep37246 PMC5112555

[jev212404-bib-0099] Das, S. , Ansel, K. M. , Bitzer, M. , Breakefield, X. O. , Charest, A. , Galas, D. J. , Gerstein, M. B. , Gupta, M. , Milosavljevic, A. , McManus, M. T. , Patel, T. , Raffai, R. L. , Rozowsky, J. , Roth, M. E. , Saugstad, J. A. , Van Keuren‐Jensen, K. , Weaver, A. M. , & Laurent, L. C. (2019). The extracellular RNA communication consortium: Establishing foundational knowledge and technologies for extracellular RNA research. Cell, 177, 231–242. 30951667 10.1016/j.cell.2019.03.023 PMC6601620

[jev212404-bib-0100] Singorenko, D. P. , Chang, V. , Whitcombe, A. , Simonov, D. , Hong, J. , Phillips, A. , Swift, S. , & Blenkiron, C. (2017). Isolation of membrane vesicles from prokaryotes: A technical and biological comparison reveals heterogeneity. Journal of Extracellular Vesicles, 6, 1324731. 28717421 10.1080/20013078.2017.1324731 PMC5505020

[jev212404-bib-0101] de Jong, O. G. , Murphy, D. E. , Mäger, I. , Willms, E. , Garcia‐Guerra, A. , Gitz‐Francois, J. J. , Lefferts, J. , Gupta, D. , Steenbeek, S. C. , van Rheenen, J. , El Andaloussi, S. , Schiffelers, R. M. , Wood, M. J. A. , & Vader, P. (2020). A CRISPR‐Cas9‐based reporter system for single‐cell detection of extracellular vesicle‐mediated functional transfer of RNA. Nature Communications, 11, 1113. 10.1038/s41467-020-14977-8 PMC7048928 32111843

[jev212404-bib-0102] de Rond, L. , Coumans, F. A. W. , Nieuwland, R. , van Leeuwen, T. G. , & van der Pol, E. (2018). Deriving extracellular vesicle size from scatter intensities measured by flow cytometry. Current Protocols in Cytometry, 86, e43. 30168659 10.1002/cpcy.43

[jev212404-bib-0103] de Rond, L. , van der Pol, E. , Hau, C. M. , Varga, Z. , Sturk, A. , van Leeuwen, T. G. , Nieuwland, R. , & Coumans, F. A. W. (2018). Comparison of generic fluorescent markers for detection of extracellular vesicles by flow cytometry. Clinical Chemistry, 64, 680–689. 29453194 10.1373/clinchem.2017.278978

[jev212404-bib-0104] de Voogt, W. S. , Tanenbaum, M. E. , & Vader, P. (2021). Illuminating RNA trafficking and functional delivery by extracellular vesicles. Advanced Drug Delivery Reviews, 174, 250–264. 33894328 10.1016/j.addr.2021.04.017

[jev212404-bib-0105] de Vrij, J. , Maas, S. L. , van Nispen, M. , Sena‐Esteves, M. , Limpens, R. W. , Koster, A. J. , Leenstra, S. , Lamfers, M. L. , & Broekman, M. L. (2013). Quantification of nanosized extracellular membrane vesicles with scanning ion occlusion sensing. Nanomedicine (Lond), 8, 1443–1458. 23384702 10.2217/nnm.12.173

[jev212404-bib-0106] Dhondt, B. , Geeurickx, E. , Tulkens, J. , Van Deun, J. , Vergauwen, G. , Lippens, L. , Miinalainen, I. , Rappu, P. , Heino, J. , Ost, P. , Lumen, N. , De Wever, O. , & Hendrix, A. (2020). Unravelling the proteomic landscape of extracellular vesicles in prostate cancer by density‐based fractionation of urine. Journal of Extracellular Vesicles, 9, 1736935. 32284825 10.1080/20013078.2020.1736935 PMC7144211

[jev212404-bib-0107] Dhondt, B. , Pinheiro, C. , Geeurickx, E. , Tulkens, J. , Vergauwen, G. , Van Der Pol, E. , Nieuwland, R. , Decock, A. , Miinalainen, I. , Rappu, P. , Schroth, G. , Kuersten, S. , Vandesompele, J. , Mestdagh, P. , Lumen, N. , De Wever, O. , & Hendrix, A. (2023). Benchmarking blood collection tubes and processing intervals for extracellular vesicle performance metrics. Journal of Extracellular Vesicles, 12, e12315. 37202906 10.1002/jev2.12315 PMC10196222

[jev212404-bib-0108] Dixson, A. C. , Dawson, T. R. , Di Vizio, D. , & Weaver, A. M. (2023). Context‐specific regulation of extracellular vesicle biogenesis and cargo selection. Nature Reviews Molecular Cell Biology, 24, 454–476. 36765164 10.1038/s41580-023-00576-0 PMC10330318

[jev212404-bib-0109] Dogrammatzis, C. , Saleh, S. , Deighan, C. , & Kalamvoki, M. (2021). Diverse populations of extracellular vesicles with opposite functions during herpes simplex virus 1 infection. Journal of Virology, 95(6), e02357–20. 33361424 10.1128/JVI.02357-20 PMC8094966

[jev212404-bib-0110] Dong, L. , Zieren, R. C. , Horie, K. , Kim, C. J. , Mallick, E. , Jing, Y. , Feng, M. , Kuczler, M. D. , Green, J. , Amend, S. R. , Witwer, K. W. , de Reijke, T. M. , Cho, Y. K. , Pienta, K. J. , & Xue, W. (2020). Comprehensive evaluation of methods for small extracellular vesicles separation from human plasma, urine and cell culture medium. Journal of Extracellular Vesicles, 10, e12044. 33489012 10.1002/jev2.12044 PMC7810129

[jev212404-bib-0111] Dooley, K. , McConnell, R. E. , Xu, K. , Lewis, N. D. , Haupt, S. , Youniss, M. R. , Martin, S. , Sia, C. L. , McCoy, C. , Moniz, R. J. , Burenkova, O. , Sanchez‐Salazar, J. , Jang, S. C. , Choi, B. , Harrison, R. A. , Houde, D. , Burzyn, D. , Leng, C. , Kirwin, K. , … Williams, D. E. (2021). A versatile platform for generating engineered extracellular vesicles with defined therapeutic properties. Molecular Therapy, 29, 1729–1743. 33484965 10.1016/j.ymthe.2021.01.020 PMC8116569

[jev212404-bib-0112] Driedonks, T. A. P. , Nijen Twilhaar, M. K. , & Nolte‐’t Hoen, E. N. M. (2019). Technical approaches to reduce interference of fetal calf serum derived RNA in the analysis of extracellular vesicle RNA from cultured cells. Journal of Extracellular Vesicles, 8(1), 1552059. 30559953 10.1080/20013078.2018.1552059 PMC6292350

[jev212404-bib-0113] Driedonks, T. , Jiang, L. , Carlson, B. , Han, Z. , Liu, G. , Queen, S. E. , Shirk, E. N. , Gololobova, O. , Liao, Z. , Nyberg, L. H. , Lima, G. , Paniushkina, L. , Garcia‐Contreras, M. , Schonvisky, K. , Castell, N. , Stover, M. , Guerrero‐Martin, S. , Richardson, R. , Smith, B. , … Witwer, K. W. (2022). Pharmacokinetics and biodistribution of extracellular vesicles administered intravenously and intranasally to Macaca nemestrina. Journal of Extracellular Biology, 1, e59. 36591537 10.1002/jex2.59 PMC9799283

[jev212404-bib-0114] Duijvesz, D. , Versluis, C. Y. , van der Fels, C. A. , Vredenbregt‐van den Berg, M. S. , Leivo, J. , Peltola, M. T. , Bangma, C. H. , Pettersson, K. S. , & Jenster, G. (2015). Immuno‐based detection of extracellular vesicles in urine as diagnostic marker for prostate cancer. International Journal of Cancer, 137, 2869–2878. 26139298 10.1002/ijc.29664

[jev212404-bib-0115] Eldh, M. , Lötvall, J. , Malmhäll, C. , & Ekström, K. (2012). Importance of RNA isolation methods for analysis of exosomal RNA: Evaluation of different methods. Molecular Immunology, 50, 278–286. 22424315 10.1016/j.molimm.2012.02.001

[jev212404-bib-0116] Elgamal, S. , Colombo, F. , Cottini, F. , Byrd, J. C. , & Cocucci, E. (2020). Imaging intercellular interaction and extracellular vesicle exchange in a co‐culture model of chronic lymphocytic leukemia and stromal cells by lattice light‐sheet fluorescence microscopy. Methods in Enzymology, 645, 79–107. 33565979 10.1016/bs.mie.2020.06.015

[jev212404-bib-0117] Enciso‐Martinez, A. , van der Pol, E. , Lenferink, A. T. M. , Terstappen, L. , van Leeuwen, T. G. , & Otto, C. (2020). Synchronized Rayleigh and Raman scattering for the characterization of single optically trapped extracellular vesicles. Nanomedicine, 24, 102109. 31669420 10.1016/j.nano.2019.102109

[jev212404-bib-0118] Erdbrügger, U. , Blijdorp, C. J. , Bijnsdorp, I. V. , Borràs, F. E. , Burger, D. , Bussolati, B. , Byrd, J. B. , Clayton, A. , Dear, J. W. , Falcón‐Pérez, J. M. , Grange, C. , Hill, A. F. , Holthöfer, H. , Hoorn, E. J. , Jenster, G. , Jimenez, C. R. , Junker, K. , Klein, J. , Knepper, M. A. , … Martens‐Uzunova, E. S. (2021). Urinary extracellular vesicles: A position paper by the Urine Task Force of the International Society for Extracellular Vesicles. Journal of Extracellular Vesicles, 10, e12093. 34035881 10.1002/jev2.12093 PMC8138533

[jev212404-bib-0119] Estrada, A. L. , Valenti, Z. J. , Hehn, G. , Amorese, A. J. , Williams, N. S. , Balestrieri, N. P. , Deighan, C. , Allen, C. P. , Spangenburg, E. E. , Kruh‐Garcia, N. A. , & Lark, D. S. (2022). Extracellular vesicle secretion is tissue‐dependent ex vivo and skeletal muscle myofiber extracellular vesicles reach the circulation in vivo. American Journal of Physiology. Cell Physiology, 322, C246–C259. 34910603 10.1152/ajpcell.00580.2020 PMC8816621

[jev212404-bib-0120] EV‐TRACK Consortium , van Deun, J. , Mestdagh, P. , Agostinis, P. , Akay, O. , Anand, S. , Anckaert, J. , Martinez, Z. A. , Baetens, T. , Beghein, E. , Bertier, L. , Berx, G. , Boere, J. , Boukouris, S. , Bremer, M. , Buschmann, D. , Byrd, J. B. , Casert, C. , Cheng, L. , … Hendrix, A. (2017). EV‐TRACK: Transparent reporting and centralizing knowledge in extracellular vesicle research. Nature Methods, 14, 228–232. 28245209 10.1038/nmeth.4185

[jev212404-bib-0121] Evtushenko, E. G. , Bagrov, D. V. , Lazarev, V. N. , Livshits, M. A. , & Khomyakova, E. (2020). Adsorption of extracellular vesicles onto the tube walls during storage in solution. PLoS ONE, 15, e0243738. 33370319 10.1371/journal.pone.0243738 PMC7769454

[jev212404-bib-0122] Fan, S. J. , Kroeger, B. , Marie, P. P. , Bridges, E. M. , Mason, J. D. , McCormick, K. , Zois, C. E. , Sheldon, H. , Khalid Alham, N. , Johnson, E. , Ellis, M. , Stefana, M. I. , Mendes, C. C. , Wainwright, S. M. , Cunningham, C. , Hamdy, F. C. , Morris, J. F. , Harris, A. L. , Wilson, C. , & Goberdhan, D. C. (2020). Glutamine deprivation alters the origin and function of cancer cell exosomes. Embo Journal, 39, e103009. 32720716 10.15252/embj.2019103009 PMC7429491

[jev212404-bib-0123] Feng, D. , Zhao, W. L. , Ye, Y. Y. , Bai, X. C. , Liu, R. Q. , Chang, L. F. , Zhou, Q. , & Sui, S. F. (2010). Cellular internalization of exosomes occurs through phagocytosis. Traffic (Copenhagen, Denmark), 11, 675–687. 20136776 10.1111/j.1600-0854.2010.01041.x

[jev212404-bib-0124] Foers, A. D. , Chatfield, S. , Dagley, L. F. , Scicluna, B. J. , Webb, A. I. , Cheng, L. , Hill, A. F. , Wicks, I. P. , & Pang, K. C. (2018). Enrichment of extracellular vesicles from human synovial fluid using size exclusion chromatography. Journal of Extracellular Vesicles, 7, 1490145. 29963299 10.1080/20013078.2018.1490145 PMC6022248

[jev212404-bib-0125] Foers, A. D. , Dagley, L. F. , Chatfield, S. , Webb, A. I. , Cheng, L. , Hill, A. F. , Wicks, I. P. , & Pang, K. C. (2020). Proteomic analysis of extracellular vesicles reveals an immunogenic cargo in rheumatoid arthritis synovial fluid. Clinical & Translational Immunology, 9, e1185. 10.1002/cti2.1185 PMC7648259 33204424

[jev212404-bib-0126] Gaetani, L. , Blennow, K. , Calabresi, P. , Di Filippo, M. , Parnetti, L. , & Zetterberg, H. (2019). Neurofilament light chain as a biomarker in neurological disorders. Journal of Neurology, Neurosurgery, and Psychiatry, 90, 870–881. 30967444 10.1136/jnnp-2018-320106

[jev212404-bib-0127] Gallart‐Palau, X. , Serra, A. , & Sze, S. K. (2016). Enrichment of extracellular vesicles from tissues of the central nervous system by PROSPR. Molecular Neurodegeneration, 11, 41. 27216497 10.1186/s13024-016-0108-1 PMC4877958

[jev212404-bib-0128] Gallego‐Perez, D. , Chang, L. , Shi, J. , Ma, J. , Kim, S. H. , Zhao, X. , Malkoc, V. , Wang, X. , Minata, M. , Kwak, K. J. , Wu, Y. , Lafyatis, G. P. , Lu, W. , Hansford, D. J. , Nakano, I. , & Lee, L. J. (2016). On‐chip clonal analysis of glioma‐stem‐cell motility and therapy resistance. Nano Letters, 16, 5326–5332. 27420544 10.1021/acs.nanolett.6b00902 PMC5040341

[jev212404-bib-0129] Gámez‐Valero, A. , Monguió‐Tortajada, M. , Carreras‐Planella, L. , Franquesa, M. I. , Beyer, K. , & Borràs, F. E. (2016). Size‐exclusion chromatography‐based isolation minimally alters extracellular vesicles’ characteristics compared to precipitating agents. Scientific Reports, 6, 33641. 27640641 10.1038/srep33641 PMC5027519

[jev212404-bib-0130] Gandham, S. , Su, X. , Wood, J. , Nocera, A L. , Chandra Alli, S. , Milane, L. , Zimmerman, A. , Amiji, M. , & Ivanov, A R. (2020). Technologies and standardization in research on extracellular vesicles. Trends in Biotechnology, 38(10), 1066–1098. 32564882 10.1016/j.tibtech.2020.05.012 PMC7302792

[jev212404-bib-0131] Gao, H. N. , Guo, H. Y. , Zhang, H. , Xie, X. L. , Wen, P. C. , & Ren, F. Z. (2019). Yak‐milk‐derived exosomes promote proliferation of intestinal epithelial cells in an hypoxic environment. Journal of Dairy Science, 102, 985–996. 30580945 10.3168/jds.2018-14946

[jev212404-bib-0132] Gao, K. , Zhu, W. , Li, H. , Ma, D. , Liu, W. , Yu, W. , Wang, L. , Cao, Y. , & Jiang, Y. (2020). Association between cytokines and exosomes in synovial fluid of individuals with knee osteoarthritis. Modern Rheumatology, 30, 758–764. 31370732 10.1080/14397595.2019.1651445

[jev212404-bib-0133] Gao, X. , Ran, N. , Dong, X. , Zuo, B. , Yang, R. , Zhou, Q. , Moulton, H. M. , Seow, Y. , & Yin, H. (2018). Anchor peptide captures, targets, and loads exosomes of diverse origins for diagnostics and therapy. Science Translational Medicine, 10(444), eaat0195. 29875202 10.1126/scitranslmed.aat0195

[jev212404-bib-0134] García‐Silva, S. , Benito‐Martín, A. , Nogués, L. , Hernández‐Barranco, A. , Mazariegos, M. S. , Santos, V. , Hergueta‐Redondo, M. , Ximénez‐Embún, P. , Kataru, R. P. , Lopez, A. A. , Merino, C. , Sánchez‐Redondo, S. , Graña‐Castro, O. , Matei, I. , Nicolás‐Avila, J. Á. , Torres‐Ruiz, R. , Rodríguez‐Perales, S. , Martínez, L. , Pérez‐Martínez, M. , … Peinado, H. (2021). Melanoma‐derived small extracellular vesicles induce lymphangiogenesis and metastasis through an NGFR‐dependent mechanism. Nature Cancer, 2, 1387–1405. 34957415 10.1038/s43018-021-00272-y PMC8697753

[jev212404-bib-0135] García‐Silva, S. , Benito‐Martín, A. , Sánchez‐Redondo, S. , Hernández‐Barranco, A. , Ximénez‐Embún, P. , Nogués, L. , Mazariegos, M. S. , Brinkmann, K. , Amor López, A. , Meyer, L. , Rodríguez, C. , García‐Martín, C. , Boskovic, J. , Letón, R. , Montero, C. , Robledo, M. , Santambrogio, L. , Sue Brady, M. , Szumera‐Ciećkiewicz, A. , … Peinado, H. (2019). Use of extracellular vesicles from lymphatic drainage as surrogate markers of melanoma progression and BRAF (V600E) mutation. Journal of Experimental Medicine, 216, 1061–1070. 30975894 10.1084/jem.20181522 PMC6504207

[jev212404-bib-0136] Gardiner, C. , Di Vizio, D. , Sahoo, S. , Théry, C. , Witwer, K. W. , Wauben, M. , & Hill, A. F. (2016). Techniques used for the isolation and characterization of extracellular vesicles: Results of a worldwide survey. Journal of Extracellular Vesicles, 5, 32945. 27802845 10.3402/jev.v5.32945 PMC5090131

[jev212404-bib-0137] Gardiner, C. , Shaw, M. , Hole, P. , Smith, J. , Tannetta, D. , Redman, C. W. , & Sargent, I. L. (2014). Measurement of refractive index by nanoparticle tracking analysis reveals heterogeneity in extracellular vesicles. Journal of Extracellular Vesicles, 3, 25361. 25425324 10.3402/jev.v3.25361 PMC4247498

[jev212404-bib-0138] Gaurivaud, P. , Ganter, S. , Villard, A. , Manso‐Silvan, L. , Chevret, D. , Boulé, C. , Monnet, V. , & Tardy, F. (2018). Mycoplasmas are no exception to extracellular vesicles release: Revisiting old concepts. PLoS ONE, 13, e0208160. 30485365 10.1371/journal.pone.0208160 PMC6261642

[jev212404-bib-0139] Gautron, J. , Stapane, L. , Roy, N. L. , Nys, Y. , Rodriguez‐Navarro, A. B. , & Hincke, M. T. (2021). Avian eggshell biomineralization: An update on its structure, mineralogy and protein tool kit. BMC Molecular and Cell Biology, 22, 11. 33579194 10.1186/s12860-021-00350-0 PMC7881572

[jev212404-bib-0140] Ge, X. , Meng, Q. , Wei, L. , Liu, J. , Li, M. , Liang, X. , Lin, F. , Zhang, Y. , Li, Y. , Liu, Z. , Fan, H. , & Zhou, X. (2021). Myocardial ischemia‐reperfusion induced cardiac extracellular vesicles harbour proinflammatory features and aggravate heart injury. Journal of Extracellular Vesicles, 10, e12072. 33664937 10.1002/jev2.12072 PMC7902529

[jev212404-bib-0141] Geeurickx, E. , Tulkens, J. , Dhondt, B. , Van Deun, J. , Lippens, L. , Vergauwen, G. , Heyrman, E. , De Sutter, D. , Gevaert, K. , Impens, F. , Miinalainen, I. , Van Bockstal, P. J. , De Beer, T. , Wauben, M. H. M. , Nolte‐’t‐Hoen, E. N. M. , Bloch, K. , Swinnen, J. V. , van der Pol, E. , Nieuwland, R. , … Hendrix, A. (2019). The generation and use of recombinant extracellular vesicles as biological reference material. Nature Communications, 10, 3288. 10.1038/s41467-019-11182-0 PMC6650486 31337761

[jev212404-bib-0142] Gelibter, S. , Marostica, G. , Mandelli, A. , Siciliani, S. , Podini, P. , Finardi, A. , & Furlan, R. (2022). The impact of storage on extracellular vesicles: A systematic study. Journal of Extracellular Vesicles, 11, e12162. 35102719 10.1002/jev2.12162 PMC8804350

[jev212404-bib-0143] Ghoroghi, S. , Mary, B. , Larnicol, A. , Asokan, N. , Klein, A. , Osmani, N. , Busnelli, I. , Delalande, F. , Paul, N. , Halary, S. , Gros, F. , Fouillen, L. , Haeberle, A. M. , Royer, C. , Spiegelhalter, C. , André‐Grégoire, G. , Mittelheisser, V. , Detappe, A. , Murphy, K. , … Hyenne, V. (2021). Ral GTPases promote breast cancer metastasis by controlling biogenesis and organ targeting of exosomes. Elife, 10, e61539. 33404012 10.7554/eLife.61539 PMC7822591

[jev212404-bib-0144] Giddings, J. C. , Yang, F. J. , & Myers, M. N. (1976). Flow‐field‐flow fractionation: A versatile new separation method. Science, 193, 1244–1245. 959835 10.1126/science.959835

[jev212404-bib-0145] Gimona, M. , Brizzi, M. F. , Choo, A. B. H. , Dominici, M. , Davidson, S. M. , Grillari, J. , Hermann, D. M. , Hill, A. F. , de Kleijn, D. , Lai, R. C. , Lai, C. P. , Lim, R. , Monguió‐Tortajada, M. , Muraca, M. , Ochiya, T. , Ortiz, L. A. , Toh, W. S. , Yi, Y. W. , Witwer, K. W. , … Lim, S. K. (2021). Critical considerations for the development of potency tests for therapeutic applications of mesenchymal stromal cell‐derived small extracellular vesicles. Cytotherapy, 23, 373–380. 33934807 10.1016/j.jcyt.2021.01.001

[jev212404-bib-0146] Gobbo, J. , Marcion, G. , Cordonnier, M. , Dias, A. M. M. , Pernet, N. , Hammann, A. , Richaud, S. , Mjahed, H. , Isambert, N. , Clausse, V. , Rébé, C. , Bertaut, A. , Goussot, V. , Lirussi, F. , Ghiringhelli, F. , de Thonel, A. , Fumoleau, P. , Seigneuric, R. , & Garrido, C. (2016). Restoring anticancer immune response by targeting tumor‐derived exosomes with a HSP70 peptide aptamer. JNCI: Journal of the National Cancer Institute, 108, 00–00. 10.1093/jnci/djv330 26598503

[jev212404-bib-0147] Gomar‐Vercher, S. , Simón‐Soro, A. , Montiel‐Company, J. M. , Almerich‐Silla, J. M. , & Mira, A. (2018). Stimulated and unstimulated saliva samples have significantly different bacterial profiles. PLoS ONE, 13, e0198021. 29856779 10.1371/journal.pone.0198021 PMC5983451

[jev212404-bib-0148] Gomes, D. E. , & Witwer, K. W. (2022). L1CAM‐associated extracellular vesicles: A systematic review of nomenclature, sources, separation, and characterization. Journal of Extracellular Biology, 1(3), e35. 35492832 10.1002/jex2.35 PMC9045013

[jev212404-bib-0149] Gorgens, A. , Bremer, M. , Ferrer‐Tur, R. , Murke, F. , Tertel, T. , Horn, P. A. , Thalmann, S. , Welsh, J. A. , Probst, C. , Guerin, C. , Boulanger, C. M. , Jones, J. C. , Hanenberg, H. , Erdbrugger, U. , Lannigan, J. , Ricklefs, F. L. , El‐Andaloussi, S. , & Giebel, B. (2019). Optimisation of imaging flow cytometry for the analysis of single extracellular vesicles by using fluorescence‐tagged vesicles as biological reference material. Journal of Extracellular Vesicles, 8, 1587567. 30949308 10.1080/20013078.2019.1587567 PMC6442110

[jev212404-bib-0150] Görgens, A. , Corso, G. , Hagey, D. W. , Jawad Wiklander, R. , Gustafsson, M. O. , Felldin, U. , Lee, Y. , Bostancioglu, R. B. , Sork, H. , Liang, X. , Zheng, W. , Mohammad, D. K. , van de Wakker, S. I. , Vader, P. , Zickler, A. M. , Mamand, D. R. , Ma, L. , Holme, M. N. , Stevens, M. M. , … El Andaloussi, S. (2022). Identification of storage conditions stabilizing extracellular vesicles preparations. Journal of Extracellular Vesicles, 11, e12238. 35716060 10.1002/jev2.12238 PMC9206228

[jev212404-bib-0151] Gori, A. , Romanato, A. , Greta, B. , Strada, A. , Gagni, P. , Frigerio, R. , Brambilla, D. , Vago, R. , Galbiati, S. , Picciolini, S. , Bedoni, M. , Daaboul, G. G. , Chiari, M. , & Cretich, M. (2020). Membrane‐binding peptides for extracellular vesicles on‐chip analysis. Journal of Extracellular Vesicles, 9, 1751428. 32363015 10.1080/20013078.2020.1751428 PMC7178839

[jev212404-bib-0152] Gould, T. J. , Hess, S. T. , & Bewersdorf, J. (2012). Optical nanoscopy: From acquisition to analysis. Annual Review of Biomedical Engineering, 14, 231–254. 10.1146/annurev-bioeng-071811-150025 PMC3568761 22559319

[jev212404-bib-0153] Gradilla, A. C. , González, E. , Seijo, I. , Andrés, G. , Bischoff, M. , González‐Mendez, L. , Sánchez, V. , Callejo, A. , Ibáñez, C. , Guerra, M. , Ortigão‐Farias, J. R. , Sutherland, J. D. , González, M. , Barrio, R. , Falcón‐Pérez, J. M. , & Guerrero, I. (2014). Exosomes as Hedgehog carriers in cytoneme‐mediated transport and secretion. Nature Communications, 5, 5649. 10.1038/ncomms6649 25472772

[jev212404-bib-0154] Granvogl, B. , Ploscher, M. , & Eichacker, L. A. (2007). Sample preparation by in‐gel digestion for mass spectrometry‐based proteomics. Analytical and Bioanalytical Chemistry, 389, 991–1002. 17639354 10.1007/s00216-007-1451-4

[jev212404-bib-0155] Gross, J. C. , Chaudhary, V. , Bartscherer, K. , & Boutros, M. (2012). Active Wnt proteins are secreted on exosomes. Nature Cell Biology, 14, 1036–1045. 22983114 10.1038/ncb2574

[jev212404-bib-0156] Gualerzi, A. , Kooijmans, S. A. A. , Niada, S. , Picciolini, S. , Brini, A. T. , Camussi, G. , & Bedoni, M. (2019). Raman spectroscopy as a quick tool to assess purity of extracellular vesicle preparations and predict their functionality. Journal of Extracellular Vesicles, 8, 1568780. 30728924 10.1080/20013078.2019.1568780 PMC6352930

[jev212404-bib-0157] Gualerzi, A. , Niada, S. , Giannasi, C. , Picciolini, S. , Morasso, C. , Vanna, R. , Rossella, V. , Masserini, M. , Bedoni, M. , Ciceri, F. , Bernardo, M. E. , Brini, A. T. , & Gramatica, F. (2017). Raman spectroscopy uncovers biochemical tissue‐related features of extracellular vesicles from mesenchymal stromal cells. Scientific Reports, 7, 9820. 28852131 10.1038/s41598-017-10448-1 PMC5575260

[jev212404-bib-0158] Gupta, D. , Liang, X. , Pavlova, S. , Wiklander, O. P. B. , Corso, G. , Zhao, Y. , Saher, O. , Bost, J. , Zickler, A. M. , Piffko, A. , Maire, C. L. , Ricklefs, F. L. , Gustafsson, O. , Llorente, V. C. , Gustafsson, M. O. , Bostancioglu, R. B. , Mamand, D. R. , Hagey, D. W. , Görgens, A. , … El Andaloussi, S. (2020). Quantification of extracellular vesicles in vitro and in vivo using sensitive bioluminescence imaging. Journal of Extracellular Vesicles, 9, 1800222. 32944187 10.1080/20013078.2020.1800222 PMC7481830

[jev212404-bib-0159] Gyorgy, B. , Modos, K. , Pallinger, E. , Paloczi, K. , Pasztoi, M. , Misjak, P. , Deli, M. A. , Sipos, A. , Szalai, A. , Voszka, I. , Polgar, A. , Toth, K. , Csete, M. , Nagy, G. , Gay, S. , Falus, A. , Kittel, A. , & Buzas, E. I. (2011). Detection and isolation of cell‐derived microparticles are compromised by protein complexes resulting from shared biophysical parameters. Blood, 117, e39–e48. 21041717 10.1182/blood-2010-09-307595

[jev212404-bib-0160] Gyorgy, B. , Paloczi, K. , Kovacs, A. , Barabas, E. , Beko, G. , Varnai, K. , Pallinger, E. , Szabo‐Taylor, K. , Szabo, T. G. , Kiss, A. A. , Falus, A. , & Buzas, E. I. (2014). Improved circulating microparticle analysis in acid‐citrate dextrose (ACD) anticoagulant tube. Thrombosis Research, 133, 285–292. 24360116 10.1016/j.thromres.2013.11.010

[jev212404-bib-0161] Hackley, V. A. , & Clogston, J. D. (2011). Measuring the hydrodynamic size of nanoparticles in aqueous media using batch‐mode dynamic light scattering. Methods in Molecular Biology, 697, 35–52. 21116952 10.1007/978-1-60327-198-1_4

[jev212404-bib-0162] Han, C. , Kang, H. , Yi, J. , Kang, M. , Lee, H. , Kwon, Y. , Jung, J. , Lee, J. , & Park, J. (2021). Single‐vesicle imaging and co‐localization analysis for tetraspanin profiling of individual extracellular vesicles. Journal of Extracellular Vesicles, 10(3), e12047. 33456726 10.1002/jev2.12047 PMC7797949

[jev212404-bib-0163] Han, P. , Bartold, P. M. , Salomon, C. , & Ivanovski, S. (2021). Salivary outer membrane vesicles and DNA methylation of small extracellular vesicles as biomarkers for periodontal status: A pilot study. International Journal of Molecular Sciences, 22, 2423. 33670900 10.3390/ijms22052423 PMC7957785

[jev212404-bib-0164] He, B. , Cai, Q. , Qiao, L. , Huang, C. Y. , Wang, S. , Miao, W. , Ha, T. , Wang, Y. , & Jin, H. (2021). RNA‐binding proteins contribute to small RNA loading in plant extracellular vesicles. Nature Plants, 7, 342–352. 33633358 10.1038/s41477-021-00863-8 PMC7979528

[jev212404-bib-0165] Hebisch, E. , Wagner, E. , Westphal, V. , Sieber, J. J. , & Lehnart, S. E. (2017). A protocol for registration and correction of multicolour STED superresolution images. Journal of Microscopy, 267, 160–175. 28370211 10.1111/jmi.12556

[jev212404-bib-0166] Heddleston, J. M. , Aaron, J. S. , Khuon, S. , & Chew, T. L. (2021). A guide to accurate reporting in digital image acquisition – can anyone replicate your microscopy data? Journal of Cell Science, 134(6), jcs254144. 33785608 10.1242/jcs.254144

[jev212404-bib-0167] Hegyesi, H. , Pallinger, É. , Mecsei, S. , Hornyák, B. , Kovácsházi, C. , Brenner, G. B. , Giricz, Z. , Pálóczi, K. , Kittel, Á. , Tóvári, J. , Turiak, L. , Khamari, D. , Ferdinandy, P. , & Buzás, E. I. (2022). Circulating cardiomyocyte‐derived extracellular vesicles reflect cardiac injury during systemic inflammatory response syndrome in mice. Cellular and Molecular Life Sciences, 79, 84. 35059851 10.1007/s00018-021-04125-w PMC8776681

[jev212404-bib-0168] Hell, S. W. , & Wichmann, J. (1994). Breaking the diffraction resolution limit by stimulated emission: Stimulated‐emission‐depletion fluorescence microscopy. Optics Letters, 19, 780–782. 19844443 10.1364/ol.19.000780

[jev212404-bib-0169] Hendrix, A. , Lippens, L. , Pinheiro, C. , Théry, C. , Martin‐Jaular, L. , Lötvall, J. , Lässer, C. , Hill, A F. , & Witwer, K. W. (2023). Extracellular vesicle analysis. Nature Reviews Methods Primers, 3, 56. 10.1038/s43586-023-00240-z PMC12968065 41809514

[jev212404-bib-0170] Herrmann, I. K. , Wood, M. J. A. , & Fuhrmann, G. (2021). Extracellular vesicles as a next‐generation drug delivery platform. Nature Nanotechnology, 16, 748–759. 10.1038/s41565-021-00931-2 34211166

[jev212404-bib-0171] Hess, S. T. , Girirajan, T. P. K. , & Mason, M. D. (2006). Ultra‐high resolution imaging by fluorescence photoactivation localization microscopy. Biophysical Journal, 91, 4258–4272. 16980368 10.1529/biophysj.106.091116 PMC1635685

[jev212404-bib-0172] Heusermann, W. , Hean, J. , Trojer, D. , Steib, E. , von Bueren, S. , Graff‐Meyer, A. , Genoud, C. , Martin, K. , Pizzato, N. , Voshol, J. , Morrissey, D. V. , Andaloussi, S. E. , Wood, M. J. , & Meisner‐Kober, N. C. (2016). Exosomes surf on filopodia to enter cells at endocytic hot spots, traffic within endosomes, and are targeted to the ER. Journal of Cell Biology, 213, 173–184. 27114500 10.1083/jcb.201506084 PMC5084269

[jev212404-bib-0173] Higginbotham, J. N. , Demory Beckler, M. , Gephart, J. D. , Franklin, J. L. , Bogatcheva, G. , Kremers, G. J. , Piston, D. W. , Ayers, G. D. , McConnell, R. E. , Tyska, M. J. , & Coffey, R. J. (2011). Amphiregulin exosomes increase cancer cell invasion. Current Biology, 21, 779–786. 21514161 10.1016/j.cub.2011.03.043 PMC3417320

[jev212404-bib-0174] Hill, A. F. , Pegtel, D. M. , Lambertz, U. , Leonardi, T. , O'Driscoll, L. , Pluchino, S. , Ter‐Ovanesyan, D. , & Nolte‐’t Hoen, E. N. (2013). ISEV position paper: Extracellular vesicle RNA analysis and bioinformatics. Journal of Extracellular Vesicles, 2, 00–00. 10.3402/jev.v2i0.22859 PMC3873759 24376909

[jev212404-bib-0175] Hogg, W. R. , & Coulter, W. H. (1967). Apparatus and method for measuring a dividing particle size of a particulate system. In United States.

[jev212404-bib-0176] Hole, P. , Sillence, K. , Hannell, C. , Maguire, C. M. , Roesslein, M. , Suarez, G. , Capracotta, S. , Magdolenova, Z. , Horev‐Azaria, L. , Dybowska, A. , Cooke, L. , Haase, A. , Contal, S. , Mano, S. , Vennemann, A. , Sauvain, J. J. , Staunton, K. C. , Anguissola, S. , Luch, A. , … Wick, P. (2013). Interlaboratory comparison of size measurements on nanoparticles using nanoparticle tracking analysis (NTA). Journal of Nanoparticle Research, 15, 2101. 24348090 10.1007/s11051-013-2101-8 PMC3857864

[jev212404-bib-0177] Hong, J. , Dauros‐Singorenko, P. , Whitcombe, A. , Payne, L. , Blenkiron, C. , Phillips, A. , & Swift, S. (2019). Analysis of the *Escherichia coli* extracellular vesicle proteome identifies markers of purity and culture conditions. Journal of Extracellular Vesicles, 8, 1632099. 31275533 10.1080/20013078.2019.1632099 PMC6598517

[jev212404-bib-0178] Hood, R. R. , DeVoe, D. L. , Atencia, J. , Vreeland, W. N. , & Omiatek, D. M. (2014). A facile route to the synthesis of monodisperse nanoscale liposomes using 3D microfluidic hydrodynamic focusing in a concentric capillary array. Lab on A Chip, 14, 2403–2409. 24825622 10.1039/c4lc00334a

[jev212404-bib-0179] Hoog, J. L. , & Lotvall, J. (2015). Diversity of extracellular vesicles in human ejaculates revealed by cryo‐electron microscopy. Journal of Extracellular Vesicles, 4, 28680. 26563734 10.3402/jev.v4.28680 PMC4643196

[jev212404-bib-0180] Hoshino, A. , Costa‐Silva, B. , Shen, T. L. , Rodrigues, G. , Hashimoto, A. , Tesic Mark, M. , Molina, H. , Kohsaka, S. , Di Giannatale, A. , Ceder, S. , Singh, S. , Williams, C. , Soplop, N. , Uryu, K. , Pharmer, L. , King, T. , Bojmar, L. , Davies, A. E. , Ararso, Y. , … Lyden, D. (2015). Tumour exosome integrins determine organotropic metastasis. Nature, 527, 329–335. 26524530 10.1038/nature15756 PMC4788391

[jev212404-bib-0181] Hoshino, A. , Kim, H. S. , Bojmar, L. , Gyan, K. E. , Cioffi, M. , Hernandez, J. , Zambirinis, C. P. , Rodrigues, G. , Molina, H. , Heissel, S. , Mark, M. T. , Steiner, L. , Benito‐Martin, A. , Lucotti, S. , Di Giannatale, A. , Offer, K. , Nakajima, M. , Williams, C. , Nogués, L. , … Lyden, D. (2020). Extracellular vesicle and particle biomarkers define multiple human cancers. Cell, 182, 1044–1061.e18. 32795414 10.1016/j.cell.2020.07.009 PMC7522766

[jev212404-bib-0182] Howell, J. C. , Watts, K. D. , Parker, M. W. , Wu, J. , Kollhoff, A. , Wingo, T. S. , Dorbin, C. D. , Qiu, D. , & Hu, W. T. (2017). Race modifies the relationship between cognition and Alzheimer's disease cerebrospinal fluid biomarkers. Alzheimer's Research & Therapy, 9, 88. 10.1186/s13195-017-0315-1 PMC5668981 29096697

[jev212404-bib-0183] Huang, Y. , Cheng, L. , Turchinovich, A. , Mahairaki, V. , Troncoso, J. C. , Pletniková, O. , Haughey, N. J. , Vella, L. J. , Hill, A. F. , Zheng, L. , & Witwer, K. W. (2020). Influence of species and processing parameters on recovery and content of brain tissue‐derived extracellular vesicles. Journal of Extracellular Vesicles, 9, 1785746. 32944174 10.1080/20013078.2020.1785746 PMC7480582

[jev212404-bib-0184] Hühmer, A. F. , Biringer, R. G. , Amato, H. , Fonteh, A. N. , & Harrington, M. G. (2006). Protein analysis in human cerebrospinal fluid: Physiological aspects, current progress and future challenges. Disease Markers, 22, 3–26. 16410649 10.1155/2006/158797 PMC3850820

[jev212404-bib-0185] Hurwitz, S. N. , Olcese, J. M. , & Meckes, D. G. Jr (2019). Extraction of extracellular vesicles from whole tissue. Journal of Visualized Experiments: JoVE, (144), 10.3791/59143 PMC7098067 30799860

[jev212404-bib-0186] Hurwitz, S. N. , Sun, L. , Cole, K. Y. , Ford, C. R. 3rd , Olcese, J. M. , & Meckes, D. G. Jr (2018). An optimized method for enrichment of whole brain‐derived extracellular vesicles reveals insight into neurodegenerative processes in a mouse model of Alzheimer's disease. Journal of Neuroscience Methods, 307, 210–220. 29894726 10.1016/j.jneumeth.2018.05.022 PMC7052957

[jev212404-bib-0187] Hyenne, V. , Apaydin, A. , Rodriguez, D. , Spiegelhalter, C. , Hoff‐Yoessle, S. , Diem, M. , Tak, S. , Lefebvre, O. , Schwab, Y. , Goetz, J. G. , & Labouesse, M. (2015). RAL‐1 controls multivesicular body biogenesis and exosome secretion. Journal of Cell Biology, 211, 27–37. 26459596 10.1083/jcb.201504136 PMC4602040

[jev212404-bib-0188] Hyenne, V. , Ghoroghi, S. , Collot, M. , Bons, J. , Follain, G. , Harlepp, S. , Mary, B. , Bauer, J. , Mercier, L. , Busnelli, I. , Lefebvre, O. , Fekonja, N. , Garcia‐Leon, M. J. , Machado, P. , Delalande, F. , López, A. A. , Silva, S. G. , Verweij, F. J. , van Niel, G. , … Goetz, J. G. (2019). Studying the fate of tumor extracellular vesicles at high spatiotemporal resolution using the zebrafish embryo. Developmental Cell, 48, 554–572.e7. 30745140 10.1016/j.devcel.2019.01.014

[jev212404-bib-0189] Ishida, T. , Hashimoto, T. , Masaki, K. , Funabashi, H. , Hirota, R. , Ikeda, T. , Tajima, H. , & Kuroda, A. (2020). Application of peptides with an affinity for phospholipid membranes during the automated purification of extracellular vesicles. Scientific Reports, 10, 18718. 33127950 10.1038/s41598-020-75561-0 PMC7603496

[jev212404-bib-0190] Izumi, T. (2021). In vivo roles of Rab27 and its effectors in exocytosis. Cell Structure and Function, 46, 79–94. 34483204 10.1247/csf.21043 PMC10511049

[jev212404-bib-0191] Jack, C. R. Jr. , Bennett, D. A. , Blennow, K. , Carrillo, M. C. , Dunn, B. , Haeberlein, S. B. , Holtzman, D. M. , Jagust, W. , Jessen, F. , Karlawish, J. , Liu, E. , Molinuevo, J. L. , Montine, T. , Phelps, C. , Rankin, K. P. , Rowe, C. C. , Scheltens, P. , Siemers, E. , Snyder, H. M. , & Sperling, R. (2018). NIA‐AA research framework: Toward a biological definition of Alzheimer's disease. Alzheimers Dement, 14, 535–562. 29653606 10.1016/j.jalz.2018.02.018 PMC5958625

[jev212404-bib-0192] Jang, S. C. , Crescitelli, R. , Cvjetkovic, A. , Belgrano, V. , Olofsson Bagge, R. , Sundfeldt, K. , Ochiya, T. , Kalluri, R. , & Lötvall, J. (2019). Mitochondrial protein enriched extracellular vesicles discovered in human melanoma tissues can be detected in patient plasma. Journal of Extracellular Vesicles, 8, 1635420. 31497264 10.1080/20013078.2019.1635420 PMC6719261

[jev212404-bib-0193] Jaqaman, K. , Loerke, D. , Mettlen, M. , Kuwata, H. , Grinstein, S. , Schmid, S. L. , & Danuser, G. (2008). Robust single‐particle tracking in live‐cell time‐lapse sequences. Nature Methods, 5, 695–702. 18641657 10.1038/nmeth.1237 PMC2747604

[jev212404-bib-0194] Jeppesen, D. K. , Fenix, A. M. , Franklin, J. L. , Higginbotham, J. N. , Zhang, Q. , Zimmerman, L. J. , Liebler, D. C. , Ping, J. , Liu, Q. , Evans, R. , Fissell, W. H. , Patton, J. G. , Rome, L. H. , Burnette, D. T. , & Coffey, R. J. (2019). Reassessment of exosome composition. Cell, 177, 428–445.e18. 30951670 10.1016/j.cell.2019.02.029 PMC6664447

[jev212404-bib-0195] Jeurissen, S. , Vergauwen, G. , Van Deun, J. , Lapeire, L. , Depoorter, V. , Miinalainen, I. , Sormunen, R. , Van den Broecke, R. , Braems, G. , Cocquyt, V. , Denys, H. , & Hendrix, A. (2017). The isolation of morphologically intact and biologically active extracellular vesicles from the secretome of cancer‐associated adipose tissue. Cell Adhesion & Migration, 11, 196–204. 28146372 10.1080/19336918.2017.1279784 PMC5351718

[jev212404-bib-0196] Jewett, K. A. , Thomas, R. E. , Phan, C. Q. , Lin, B. , Milstein, G. , Yu, S. , Bettcher, L. F. , Neto, F. C. , Djukovic, D. , Raftery, D. , Pallanck, L. J. , & Davis, M. Y. (2021). Glucocerebrosidase reduces the spread of protein aggregation in a Drosophila melanogaster model of neurodegeneration by regulating proteins trafficked by extracellular vesicles. Plos Genetics, 17, e1008859. 33539341 10.1371/journal.pgen.1008859 PMC7888665

[jev212404-bib-0197] Jingami, N. , Uemura, K. , Asada‐Utsugi, M. , Kuzuya, A. , Yamada, S. , Ishikawa, M. , Kawahara, T. , Iwasaki, T. , Atsuchi, M. , Takahashi, R. , & Kinoshita, A. (2019). Two‐point dynamic observation of Alzheimer's disease cerebrospinal fluid biomarkers in idiopathic normal pressure hydrocephalus. Journal of Alzheimer's Disease, 72, 271–277. 10.3233/JAD-190775 PMC6839467 31561378

[jev212404-bib-0198] Jingushi, K. , Uemura, M. , Ohnishi, N. , Nakata, W. , Fujita, K. , Naito, T. , Fujii, R. , Saichi, N. , Nonomura, N. , Tsujikawa, K. , & Ueda, K. (2018). Extracellular vesicles isolated from human renal cell carcinoma tissues disrupt vascular endothelial cell morphology via azurocidin. International Journal of Cancer, 142, 607–617. 28975613 10.1002/ijc.31080

[jev212404-bib-0199] Johnsen, K. B. , Gudbergsson, J. M. , Andresen, T. L. , & Simonsen, J. B. (2019). What is the blood concentration of extracellular vesicles? Implications for the use of extracellular vesicles as blood‐borne biomarkers of cancer. Biochimica et Biophysica Acta (BBA) ‐ Reviews on Cancer, 1871, 109–116. 30528756 10.1016/j.bbcan.2018.11.006

[jev212404-bib-0200] Jones, R. R. , Hooper, D. C. , Zhang, L. , Wolverson, D. , & Valev, V. K. (2019). Raman techniques: Fundamentals and frontiers. Nanoscale Research Letters, 14, 231. 31300945 10.1186/s11671-019-3039-2 PMC6626094

[jev212404-bib-0201] Joshi, B. S. , de Beer, M. A. , Giepmans, B. N. G. , & Zuhorn, I. S. (2020). Endocytosis of extracellular vesicles and release of their cargo from endosomes. ACS Nano, 14, 4444–4455. 32282185 10.1021/acsnano.9b10033 PMC7199215

[jev212404-bib-0202] Joy, A. P. , Ayre, D. C. , Chute, I. C. , Beauregard, A. P. , Wajnberg, G. , Ghosh, A. , Lewis, S. M. , Ouellette, R. J. , & Barnett, D. A. (2018). Proteome profiling of extracellular vesicles captured with the affinity peptide Vn96: Comparison of Laemmli and TRIzol© protein‐extraction methods. Journal of Extracellular Vesicles, 7, 1438727. 29511462 10.1080/20013078.2018.1438727 PMC5827780

[jev212404-bib-0203] Jurgielewicz, B. J. , Yao, Y. , & Stice, S. L. (2020). Kinetics and specificity of HEK293T extracellular vesicle uptake using imaging flow cytometry. Nanoscale Research Letters, 15, 170. 32833066 10.1186/s11671-020-03399-6 PMC7445225

[jev212404-bib-0204] Kaczor‐Urbanowicz, K. E. , Wei, F. , Rao, S. L. , Kim, J. , Shin, H. , Cheng, J. , Tu, M. , Wong, D. T. W. , & Kim, Y. (2019). Clinical validity of saliva and novel technology for cancer detection. Biochimica et Biophysica Acta (BBA) ‐ Reviews on Cancer, 1872, 49–59. 31152821 10.1016/j.bbcan.2019.05.007 PMC6692231

[jev212404-bib-0205] Kahlert, C. , Melo, S. A. , Protopopov, A. , Tang, J. , Seth, S. , Koch, M. , Zhang, J. , Weitz, J. , Chin, L. , Futreal, A. , & Kalluri, R. (2014). Identification of double‐stranded genomic DNA spanning all chromosomes with mutated KRAS and p53 DNA in the serum exosomes of patients with pancreatic cancer. Journal of Biological Chemistry, 289, 3869–3875. 24398677 10.1074/jbc.C113.532267 PMC3924256

[jev212404-bib-0206] Kameli, N. , Becker, H. E. F. , Welbers, T. , Jonkers, D. , Penders, J. , Savelkoul, P. , & Stassen, F. R. (2021). Metagenomic profiling of fecal‐derived bacterial membrane vesicles in Crohn's disease patients. Cells, 10(10), 2795. 34685776 10.3390/cells10102795 PMC8535131

[jev212404-bib-0207] Kang, M. , Jordan, V. , Blenkiron, C. , & Chamley, L. W. (2021). Biodistribution of extracellular vesicles following administration into animals: A systematic review. Journal of Extracellular Vesicles, 10, e12085. 34194679 10.1002/jev2.12085 PMC8224174

[jev212404-bib-0208] Karimi, N. , Cvjetkovic, A. , Jang, S. C. , Crescitelli, R. , Hosseinpour Feizi, M. A. , Nieuwland, R. , Lötvall, J. , & Lässer, C. (2018). Detailed analysis of the plasma extracellular vesicle proteome after separation from lipoproteins. Cellular and Molecular Life Sciences, 75, 2873–2886. 29441425 10.1007/s00018-018-2773-4 PMC6021463

[jev212404-bib-0209] Karimi, N. , Dalirfardouei, R. , Dias, T. , Lötvall, J. , & Lässer, C. (2022). Tetraspanins distinguish separate extracellular vesicle subpopulations in human serum and plasma ‐ Contributions of platelet extracellular vesicles in plasma samples. Journal of Extracellular Vesicles, 11, e12213. 35524458 10.1002/jev2.12213 PMC9077141

[jev212404-bib-0210] Karttunen, J. , Heiskanen, M. , Navarro‐Ferrandis, V. , Gupta, S. D , Lipponen, A. , Puhakka, N. , Rilla, K. , Koistinen, A. , & Pitkänen, A. (2019). Precipitation‐based extracellular vesicle isolation from rat plasma co‐precipitate vesicle‐free microRNAs. Journal of Extracellular Vesicles, 8, 1555410. 30574280 10.1080/20013078.2018.1555410 PMC6300090

[jev212404-bib-0211] Keenan, J. I. , & Allardyce, R. A. (2000). Iron influences the expression of *Helicobacter pylori* outer membrane vesicle‐associated virulence factors. European Journal of Gastroenterology & Hepatology, 12, 1267–1273. 11192314 10.1097/00042737-200012120-00002

[jev212404-bib-0212] Keremi, B. , Beck, A. , Fabian, T. K. , Fabian, G. , Szabo, G. , Nagy, A. , & Varga, G. (2017). Stress and salivary glands. Current Pharmaceutical Design, 23, 4057–4065. 28215154 10.2174/1381612823666170215110648

[jev212404-bib-0213] Kestens, V. , Bozatzidis, V. , De Temmerman, P. J. , Ramaye, Y. , & Roebben, G. (2017). Validation of a particle tracking analysis method for the size determination of nano‐ and microparticles. Journal of Nanoparticle Research, 19, 271. 28824287 10.1007/s11051-017-3966-8 PMC5543194

[jev212404-bib-0214] Khater, I. M. , Nabi, I. R. , & Hamarneh, G. (2020). A review of super‐resolution single‐molecule localization microscopy cluster analysis and quantification methods. Patterns (N Y), 1, 100038. 33205106 10.1016/j.patter.2020.100038 PMC7660399

[jev212404-bib-0215] Khurshid, Z. , Zohaib, S. , Najeeb, S. , Zafar, M. S. , Slowey, P. D. , & Almas, K. (2016). Human saliva collection devices for proteomics: An update. International Journal of Molecular Sciences, 17, 846. 27275816 10.3390/ijms17060846 PMC4926380

[jev212404-bib-0216] Klar, T. A. , & Hell, S. W. (1999). Subdiffraction resolution in far‐field fluorescence microscopy. Optics Letters, 24, 954–956. 18073907 10.1364/ol.24.000954

[jev212404-bib-0217] Klener, J. , Hofbauerová, K. , Bartoš, A. , Ríčný, J. , Rípová, D. , & Kopecký, V. (2014). Instability of cerebrospinal fluid after delayed storage and repeated freezing: A holistic study by drop coating deposition Raman spectroscopy. Clinical Chemistry and Laboratory Medicine, 52, 657–664. 24293450 10.1515/cclm-2013-0800

[jev212404-bib-0218] Klont, F. , Bras, L. , Wolters, J. C. , Ongay, S. , Bischoff, R. , Halmos, G. B. , & Horvatovich, P. (2018). Assessment of sample preparation bias in mass spectrometry‐based proteomics. Analytical Chemistry, 90, 5405–5413. 29608294 10.1021/acs.analchem.8b00600 PMC5906755

[jev212404-bib-0219] Kobayashi‐Sun, J. , Yamamori, S. , Kondo, M. , Kuroda, J. , Ikegame, M. , Suzuki, N. , Kitamura, K. I. , Hattori, A. , Yamaguchi, M. , & Kobayashi, I. (2020). Uptake of osteoblast‐derived extracellular vesicles promotes the differentiation of osteoclasts in the zebrafish scale. Communications Biology, 3, 190. 32327701 10.1038/s42003-020-0925-1 PMC7181839

[jev212404-bib-0220] Koles, K. , Nunnari, J. , Korkut, C. , Barria, R. , Brewer, C. , Li, Y. , Leszyk, J. , Zhang, B. , & Budnik, V. (2012). Mechanism of evenness interrupted (Evi)‐exosome release at synaptic boutons. Journal of Biological Chemistry, 287, 16820–16834. 22437826 10.1074/jbc.M112.342667 PMC3351299

[jev212404-bib-0221] Kolhe, R. , Owens, V. , Sharma, A. , Lee, T. J. , Zhi, W. , Ghilzai, U. , Mondal, A. K. , Liu, Y. , Isales, C. M. , Hamrick, M. W. , Hunter, M. , & Fulzele, S. (2020). Sex‐specific differences in extracellular vesicle protein cargo in synovial fluid of patients with osteoarthritis. Life (Basel), 10(12), 337. 33321751 10.3390/life10120337 PMC7763294

[jev212404-bib-0222] Koliha, N. , Wiencek, Y. , Heider, U. , Jüngst, C. , Kladt, N. , Krauthäuser, S. , Johnston, I. C. , Bosio, A. , Schauss, A. , & Wild, S. (2016). A novel multiplex bead‐based platform highlights the diversity of extracellular vesicles. Journal of Extracellular Vesicles, 5, 29975. 26901056 10.3402/jev.v5.29975 PMC4762227

[jev212404-bib-0223] Korkut, C. , Li, Y. , Koles, K. , Brewer, C. , Ashley, J. , Yoshihara, M. , & Budnik, V. (2013). Regulation of postsynaptic retrograde signaling by presynaptic exosome release. Neuron, 77, 1039–1046. 23522040 10.1016/j.neuron.2013.01.013 PMC3626103

[jev212404-bib-0224] Kowal, J. , Arras, G. , Colombo, M. , Jouve, M. , Morath, J. P. , Primdal‐Bengtson, B. , Dingli, F. , Loew, D. , Tkach, M. , & Théry, C. (2016). Proteomic comparison defines novel markers to characterize heterogeneous populations of extracellular vesicle subtypes. PNAS, 113, E968–E977. 26858453 10.1073/pnas.1521230113 PMC4776515

[jev212404-bib-0225] Kreimer, S. , Belov, A. M. , Ghiran, I. , Murthy, S. K. , Frank, D. A. , & Ivanov, A. R. (2015). Mass‐spectrometry‐based molecular characterization of extracellular vesicles: lipidomics and proteomics. Journal of Proteome Research, 14, 2367–2384. 25927954 10.1021/pr501279t

[jev212404-bib-0226] Krušić Alić, V. , Malenica, M. , Biberić, M. , Zrna, S. , Valenčić, L. , Šuput, A. , Kalagac Fabris, L. , Wechtersbach, K. , Kojc, N. , Kurtjak, M. , Kučić, N. , & Grabušić, K. (2022). Extracellular vesicles from human cerebrospinal fluid are effectively separated by sepharose CL‐6B‐comparison of four gravity‐flow size exclusion chromatography methods. Biomedicines, 10, 785. 35453535 10.3390/biomedicines10040785 PMC9032713

[jev212404-bib-0227] Kuehn, M. J. , & Kesty, N. C. (2005). Bacterial outer membrane vesicles and the host‐pathogen interaction. Genes & Development, 19, 2645–2655. 16291643 10.1101/gad.1299905

[jev212404-bib-0228] Kumeda, N. , Ogawa, Y. , Akimoto, Y. , Kawakami, H. , Tsujimoto, M. , & Yanoshita, R. (2017). Characterization of membrane integrity and morphological stability of human salivary exosomes. Biological & Pharmaceutical Bulletin, 40, 1183–1191. 28768999 10.1248/bpb.b16-00891

[jev212404-bib-0229] Kwizera, E. A. , O'Connor, R. , Vinduska, V. , Williams, M. , Butch, E. R. , Snyder, S. E. , Chen, X. , & Huang, X. (2018). Molecular detection and analysis of exosomes using surface‐enhanced Raman scattering gold nanorods and a miniaturized device. Theranostics, 8, 2722–2738. 29774071 10.7150/thno.21358 PMC5957005

[jev212404-bib-0230] Lacroix, R. , Judicone, C. , Poncelet, P. , Robert, S. , Arnaud, L. , Sampol, J. , & Dignat‐George, F. (2012). Impact of pre‐analytical parameters on the measurement of circulating microparticles: Towards standardization of protocol. Journal of Thrombosis and Haemostasis, 10, 437–446. 22212198 10.1111/j.1538-7836.2011.04610.x

[jev212404-bib-0231] Lai, C. P. , Kim, E. Y. , Badr, C. E. , Weissleder, R. , Mempel, T. R. , Tannous, B. A. , & Breakefield, X. O. (2015). Visualization and tracking of tumour extracellular vesicle delivery and RNA translation using multiplexed reporters. Nature Communications, 6, 7029. 10.1038/ncomms8029 PMC4435621 25967391

[jev212404-bib-0232] Lamparski, H. G. , Metha‐Damani, A. , Yao, J. Y. , Patel, S. , Hsu, D. H. , Ruegg, C. , & Le Pecq, J. B. (2002). Production and characterization of clinical grade exosomes derived from dendritic cells. Journal of Immunological Methods, 270, 211–226. 12379326 10.1016/s0022-1759(02)00330-7

[jev212404-bib-0233] Langer, J. , Jimenez de Aberasturi, D. , Aizpurua, J. , Alvarez‐Puebla, R. A. , Auguie, B. , Baumberg, J. J. , Bazan, G. C. , Bell, S. E. J. , Boisen, A. , Brolo, A. G. , Choo, J. , Cialla‐May, D. , Deckert, V. , Fabris, L. , Faulds, K. , Garcia de Abajo, F. J. , Goodacre, R. , Graham, D. , Haes, A. J. , … Liz‐Marzan, L. M. (2020). Present and future of surface‐enhanced Raman scattering. ACS Nano, 14, 28–117. 31478375 10.1021/acsnano.9b04224 PMC6990571

[jev212404-bib-0234] Lázaro‐Ibáñez, E. , Faruqu, F. N. , Saleh, A. F. , Silva, A. M. , Tzu‐Wen Wang, J. , Rak, J. , Al‐Jamal, K. T. , & Dekker, N. (2021). Selection of fluorescent, bioluminescent, and radioactive tracers to accurately reflect extracellular vesicle biodistribution in vivo. ACS Nano, 15, 3212–3227. 33470092 10.1021/acsnano.0c09873 PMC7905875

[jev212404-bib-0235] Lázaro‐Ibáñez, E. , Lässer, C. , Shelke, G. V. , Crescitelli, R. , Jang, S. C. , Cvjetkovic, A. , García‐Rodríguez, A. , & Lötvall, J. (2019). DNA analysis of low‐ and high‐density fractions defines heterogeneous subpopulations of small extracellular vesicles based on their DNA cargo and topology. Journal of Extracellular Vesicles, 8, 1656993. 31497265 10.1080/20013078.2019.1656993 PMC6719264

[jev212404-bib-0236] Le Saux, S. , Aarrass, H. , Lai‐Kee‐Him, J. , Bron, P. , Armengaud, J. , Miotello, G. , Bertrand‐Michel, J. , Dubois, E. , George, S. , Faklaris, O. , Devoisselle, J. M. , Legrand, P. , Chopineau, J. , & Morille, M. (2020). Post‐production modifications of murine mesenchymal stem cell (mMSC) derived extracellular vesicles (EVs) and impact on their cellular interaction. Biomaterials, 231, 119675. 31838346 10.1016/j.biomaterials.2019.119675

[jev212404-bib-0237] LeClaire, M. , Wohlschlegel, J. A. , Chang, H. C. , Wadehra, M. , Yu, W. , Rao, J. , Elashoff, D. , Gimzewski, J. K. , & Sharma, S. (2021). Nanoscale extracellular vesicles carry the mechanobiology signatures of breast cancer cells. ACS Applied Nano Materials, 4, 9876–9885.

[jev212404-bib-0238] Lee, T. H. , Chennakrishnaiah, S. , Audemard, E. , Montermini, L. , Meehan, B. , & Rak, J. (2014). Oncogenic ras‐driven cancer cell vesiculation leads to emission of double‐stranded DNA capable of interacting with target cells. Biochemical and Biophysical Research Communications, 451, 295–301. 25086355 10.1016/j.bbrc.2014.07.109

[jev212404-bib-0239] Lee, W. , Nanou, A. , Rikkert, L. , Coumans, F. A. W. , Otto, C. , Terstappen, L. , & Offerhaus, H. L. (2018). Label‐free prostate cancer detection by characterization of extracellular vesicles using raman spectroscopy. Analytical Chemistry, 90, 11290–11296. 30157378 10.1021/acs.analchem.8b01831 PMC6170952

[jev212404-bib-0240] Lehrich, B. M. , Liang, Y. , & Fiandaca, M. S. (2021). Foetal bovine serum influence on in vitro extracellular vesicle analyses. Journal of Extracellular Vesicles, 10, e12061. 33532042 10.1002/jev2.12061 PMC7830136

[jev212404-bib-0241] Lehrich, B. M. , Liang, Y. , Khosravi, P. , Federoff, H. J. , & Fiandaca, M. S. (2018). Fetal bovine serum‐derived extracellular vesicles persist within vesicle‐depleted culture media. International Journal of Molecular Sciences, 19, 3538. 30423996 10.3390/ijms19113538 PMC6275013

[jev212404-bib-0242] Lener, T. , Gimona, M. , Aigner, L. , Börger, V. , Buzas, E. , Camussi, G. , Chaput, N. , Chatterjee, D. , Court, F. A. , del Portillo, H. A. , O'Driscoll, L. , Fais, S. , Falcon‐Perez, J. M. , Felderhoff‐Mueser, U. , Fraile, L. , Gho, Y. S. , Görgens, A. , Gupta, R. C. , Hendrix, A. , … Giebel, B. (2015). Applying extracellular vesicles based therapeutics in clinical trials – An ISEV position paper. Journal of Extracellular Vesicles, 4, 30087. 26725829 10.3402/jev.v4.30087 PMC4698466

[jev212404-bib-0243] Lennon, K. M. , Wakefield, D. L. , Maddox, A. L. , Brehove, M. S. , Willner, A. N. , Garcia‐Mansfield, K. , Meechoovet, B. , Reiman, R. , Hutchins, E. , Miller, M. M. , Goel, A. , Pirrotte, P. , Van Keuren‐Jensen, K. , & Jovanovic‐Talisman, T. (2019). Single molecule characterization of individual extracellular vesicles from pancreatic cancer. Journal of Extracellular Vesicles, 8, 1685634. 31741725 10.1080/20013078.2019.1685634 PMC6844376

[jev212404-bib-0244] Lewczuk, P. , Beck, G. , Ganslandt, O. , Esselmann, H. , Deisenhammer, F. , Regeniter, A. , Petereit, H. F. , Tumani, H. , Gerritzen, A. , Oschmann, P. , Schröder, J. , Schönknecht, P. , Zimmermann, K. , Hampel, H. , Bürger, K. , Otto, M. , Haustein, S. , Herzog, K. , Dannenberg, R. , … Wiltfang, J. (2006). International quality control survey of neurochemical dementia diagnostics. Neuroscience Letters, 409, 1–4. 17045397 10.1016/j.neulet.2006.07.009

[jev212404-bib-0245] Li, D. , Shao, L. , Chen, B. C. , Zhang, X. , Zhang, M. , Moses, B. , Milkie, D. E. , Beach, J. R. , Hammer, J. A. 3rd , Pasham, M. , Kirchhausen, T. , Baird, M. A. , Davidson, M. W. , Xu, P. , & Betzig, E. (2015). ADVANCED IMAGING. Extended‐resolution structured illumination imaging of endocytic and cytoskeletal dynamics. Science, 349, aab3500. 26315442 10.1126/science.aab3500 PMC4659358

[jev212404-bib-0246] Li, G. , Shofer, J. B. , Petrie, E. C. , Yu, C. E. , Wilkinson, C. W. , Figlewicz, D. P. , Shutes‐David, A. , Zhang, J. , Montine, T. J. , Raskind, M. A. , Quinn, J. F. , Galasko, D. R. , & Peskind, E. R. (2017). Cerebrospinal fluid biomarkers for Alzheimer's and vascular disease vary by age, gender, and APOE genotype in cognitively normal adults. Alzheimer's Research & Therapy, 9, 48. 10.1186/s13195-017-0271-9 PMC5496132 28673336

[jev212404-bib-0247] Li‐Hui, W. , Chuan‐Quan, L. , Long, Y. , Ru‐Liu, L. , Long‐Hui, C. , & Wei‐Wen, C. (2016). Gender differences in the saliva of young healthy subjects before and after citric acid stimulation. Clinica Chimica Acta, 460, 142–145. 10.1016/j.cca.2016.06.040 27374299

[jev212404-bib-0248] Liangsupree, T. , Multia, E. , & Riekkola, M. L. (2021). Modern isolation and separation techniques for extracellular vesicles. Journal of Chromatography A, 1636, 461773. 33316564 10.1016/j.chroma.2020.461773

[jev212404-bib-0249] Liao, Z. , Jaular, L. M. , Soueidi, E. , Jouve, M. , Muth, D. C. , Schøyen, T. H. , Seale, T. , Haughey, N. J. , Ostrowski, M. , Théry, C. , & Witwer, K. W. (2019). Acetylcholinesterase is not a generic marker of extracellular vesicles. Journal of Extracellular Vesicles, 8, 1628592. 31303981 10.1080/20013078.2019.1628592 PMC6609367

[jev212404-bib-0250] Liebler, D. C. , & Zimmerman, L. J. (2013). Targeted quantitation of proteins by mass spectrometry. Biochemistry, 52, 3797–3806. 23517332 10.1021/bi400110b PMC3674507

[jev212404-bib-0251] Liégeois, S. , Benedetto, A. , Garnier, J. M. , Schwab, Y. , & Labouesse, M. (2006). The V0‐ATPase mediates apical secretion of exosomes containing Hedgehog‐related proteins in Caenorhabditis elegans. Journal of Cell Biology, 173, 949–961. 16785323 10.1083/jcb.200511072 PMC2063919

[jev212404-bib-0252] Ligtenberg, A. J. , Liem, E. H. , Brand, H. S. , & Veerman, E. C. (2016). The effect of exercise on salivary viscosity. Diagnostics (Basel), 6, 40. 27854320 10.3390/diagnostics6040040 PMC5192515

[jev212404-bib-0253] Lim, G. T. , You, D. G. , Han, H. S. , Lee, H. , Shin, S. , Oh, B. H. , Kumar, E. K. P. , Um, W. , Kim, C. H. , Han, S. , Lee, S. , Lim, S. , Yoon, H. Y. , Kim, K. , Kwon, I. C. , Jo, D. G. , Cho, Y. W. , & Park, J. H. (2021). Bioorthogonally surface‐edited extracellular vesicles based on metabolic glycoengineering for CD44‐mediated targeting of inflammatory diseases. Journal of Extracellular Vesicles, 10, e12077. 33738083 10.1002/jev2.12077 PMC7953464

[jev212404-bib-0254] Limbutara, K. , Chou, C. L. , & Knepper, M. A. (2020). Quantitative proteomics of all 14 renal tubule segments in rat. Journal of the American Society of Nephrology, 31, 1255–1266. 32358040 10.1681/ASN.2020010071 PMC7269347

[jev212404-bib-0255] Linares, R. , Tan, S. , Gounou, C. , Arraud, N. , & Brisson, A. R. (2015). High‐speed centrifugation induces aggregation of extracellular vesicles. Journal of Extracellular Vesicles, 4, 29509. 26700615 10.3402/jev.v4.29509 PMC4689953

[jev212404-bib-0256] Lischnig, A. , Bergqvist, M. , Ochiya, T. , & Lässer, C. (2022). Quantitative proteomics identifies proteins enriched in large and small extracellular vesicles. Molecular & Cellular Proteomics, 21, 100273. 35918030 10.1016/j.mcpro.2022.100273 PMC9486130

[jev212404-bib-0257] Liu, D. , Kou, X. , Chen, C. , Liu, S. , Liu, Y. , Yu, W. , Yu, T. , Yang, R. , Wang, R. , Zhou, Y. , & Shi, S. (2018). Circulating apoptotic bodies maintain mesenchymal stem cell homeostasis and ameliorate osteopenia via transferring multiple cellular factors. Cell Research, 28, 918–933. 30030518 10.1038/s41422-018-0070-2 PMC6123409

[jev212404-bib-0258] Liu, H. , Tian, Y. , Xue, C. , Niu, Q. , Chen, C. , & Yan, X. (2022). Analysis of extracellular vesicle DNA at the single‐vesicle level by nano‐flow cytometry. Journal of Extracellular Vesicles, 11, e12206. 35373518 10.1002/jev2.12206 PMC8977970

[jev212404-bib-0259] Liu, Q. , Rojas‐Canales, D. M. , Divito, S. J. , Shufesky, W. J. , Stolz, D. B. , Erdos, G. , Sullivan, M. L. , Gibson, G. A. , Watkins, S. C. , Larregina, A. T. , & Morelli, A. E. (2016). Donor dendritic cell‐derived exosomes promote allograft‐targeting immune response. Journal of Clinical Investigation, 126, 2805–2820. 27348586 10.1172/JCI84577 PMC4966303

[jev212404-bib-0260] Liu, X. , Yang, X. , Sun, W. , Wu, Q. , Song, Y. , Yuan, L. , & Yang, G. (2019). Systematic evolution of ligands by exosome enrichment: A proof‐of‐concept study for exosome‐based targeting peptide screening. Advanced Biosystems, 3, e1800275. 32627374 10.1002/adbi.201800275

[jev212404-bib-0261] Liu, Y. , Li, H. , Wang, J. , Xue, Q. , Yang, X. , Kang, Y. , Li, M. , Xu, J. , Li, G. , Li, C. , Chang, H. C. , Su, K. P. , & Wang, F. (2020). Association of cigarette smoking with cerebrospinal fluid biomarkers of neurodegeneration, neuroinflammation, and oxidation. JAMA Network Open, 3, e2018777. 33006621 10.1001/jamanetworkopen.2020.18777 PMC7532384

[jev212404-bib-0262] Liu, Z. , Cauvi, D. M. , Bernardino, E. M. A. , Lara, B. , Lizardo, R. E. , Hawisher, D. , Bickler, S. , & De Maio, A. (2018). Isolation and characterization of human urine extracellular vesicles. Cell Stress & Chaperones, 23, 943–953. 29796787 10.1007/s12192-018-0902-5 PMC6111092

[jev212404-bib-0263] Lobb, R. J. , Becker, M. , Wen, S. W. , Wong, C. S. , Wiegmans, A. P. , Leimgruber, A. , & Möller, A. (2015). Optimized exosome isolation protocol for cell culture supernatant and human plasma. Journal of Extracellular Vesicles, 4, 27031. 26194179 10.3402/jev.v4.27031 PMC4507751

[jev212404-bib-0264] Long, Q. , Upadhya, D. , Hattiangady, B. , Kim, D. K. , An, S. Y. , Shuai, B. , Prockop, D. J. , & Shetty, A. K. (2017). Intranasal MSC‐derived A1‐exosomes ease inflammation, and prevent abnormal neurogenesis and memory dysfunction after status epilepticus. PNAS, 114, E3536–E3545. 28396435 10.1073/pnas.1703920114 PMC5410779

[jev212404-bib-0265] López‐Guerrero, J. A. , Valés‐Gómez, M. , Borrás, F. E. , Falcón‐Pérez, J. M. , Vicent, M. J. , & Yáñez‐Mó, M. (2023). Standardising the preanalytical reporting of biospecimens to improve reproducibility in extracellular vesicle research – A GEIVEX study. Journal of Extracellular Biology, 2, e76. 10.1002/jex2.76 PMC11080825 38939690

[jev212404-bib-0266] Lőrincz, Á. M. , Timár, C. I. , Marosvári, K. A. , Veres, D. S. , Otrokocsi, L. , Kittel, Á. , & Ligeti, E. (2014). Effect of storage on physical and functional properties of extracellular vesicles derived from neutrophilic granulocytes. Journal of Extracellular Vesicles, 3, 25465. 25536933 10.3402/jev.v3.25465 PMC4275651

[jev212404-bib-0267] Lotvall, J. , Hill, A. F. , Hochberg, F. , Buzas, E. I. , Di Vizio, D. , Gardiner, C. , Gho, Y. S. , Kurochkin, I. V. , Mathivanan, S. , Quesenberry, P. , Sahoo, S. , Tahara, H. , Wauben, M. H. , Witwer, K. W. , & Thery, C. (2014). Minimal experimental requirements for definition of extracellular vesicles and their functions: A position statement from the International Society for Extracellular Vesicles. Journal of Extracellular Vesicles, 3, 26913. 25536934 10.3402/jev.v3.26913 PMC4275645

[jev212404-bib-0268] Lucas, M. , Ryan, J. M. , Watkins, J. , Early, K. , Kruh‐Garcia, N. A. , Mehaffy, C. , & Dobos, K. M. (2021). Extraction and separation of mycobacterial proteins. Methods in Molecular Biology, 2314, 77–107. 34235649 10.1007/978-1-0716-1460-0_3

[jev212404-bib-0269] Lucey, B. P. , Fagan, A. M. , Holtzman, D. M. , Morris, J. C. , & Bateman, R. J. (2017). Diurnal oscillation of CSF Aβ and other AD biomarkers. Molecular Neurodegeneration, 12, 36. 28478762 10.1186/s13024-017-0161-4 PMC5421331

[jev212404-bib-0270] Lucien, F. , Gustafson, D. , Lenassi, M. , Li, B. , Teske, J. J. , Boilard, E. , Clemm von Hohenberg, K. , Falcón‐Perez, J. M. , Gualerzi, A. , Reale, A. , Jones, J. C. , Lässer, C. , Lawson, C. , Nazarenko, I. , O'Driscoll, L. , Pink, R. , Siljander, P. R.‐M. , Soekmadji, C. , Hendrix, A. , … Nieuwland, R. (2023). MIBlood‐EV: Minimal information to enhance the quality and reproducibility of blood extracellular vesicle research. Journal of Extracellular Vesicles, 12(12), e12385. 38063210 10.1002/jev2.12385 PMC10704543

[jev212404-bib-0271] Lunavat, T. R. , Cheng, L. , Einarsdottir, B. O. , Olofsson Bagge, R. , Veppil Muralidharan, S. , Sharples, R. A. , Lässer, C. , Gho, Y. S. , Hill, A. F. , Nilsson, J. A. , & Lötvall, J. (2017). BRAF(V600) inhibition alters the microRNA cargo in the vesicular secretome of malignant melanoma cells. PNAS, 114, E5930–E5939. 28684402 10.1073/pnas.1705206114 PMC5530690

[jev212404-bib-0272] Lundy, S. K. , Klinker, M. W. , & Fox, D. A. (2015). Killer B lymphocytes and their fas ligand positive exosomes as inducers of immune tolerance. Frontiers in Immunology, 6, 122. 25852690 10.3389/fimmu.2015.00122 PMC4367442

[jev212404-bib-0273] Luo, W. , Dai, Y. , Chen, Z. , Yue, X. , Andrade‐Powell, K. C. , & Chang, J. (2020). Spatial and temporal tracking of cardiac exosomes in mouse using a nano‐luciferase‐CD63 fusion protein. Communications Biology, 3, 114. 32157172 10.1038/s42003-020-0830-7 PMC7064570

[jev212404-bib-0274] Ma, D. , Huang, C. , Zheng, J. , Tang, J. , Li, J. , Yang, J. , & Yang, R. (2018). Quantitative detection of exosomal microRNA extracted from human blood based on surface‐enhanced Raman scattering. Biosensors & Bioelectronics, 101, 167–173. 29073517 10.1016/j.bios.2017.08.062

[jev212404-bib-0275] Mackenzie, K. , Foot, N. J. , Anand, S. , Dalton, H. E. , Chaudhary, N. , Collins, B. M. , Mathivanan, S. , & Kumar, S. (2016). Regulation of the divalent metal ion transporter via membrane budding. Cell Discovery, 2, 16011. 27462458 10.1038/celldisc.2016.11 PMC4914834

[jev212404-bib-0276] Maire, C. L. , Fuh, M. M. , Kaulich, K. , Fita, K. D. , Stevic, I. , Heiland, D. H. , Welsh, J. A. , Jones, J. C. , Görgens, A. , Ricklefs, T. , Dührsen, L. , Sauvigny, T. , Joosse, S. A. , Reifenberger, G. , Pantel, K. , Glatzel, M. , Miklosi, A. G. , Felce, J. H. , Caselli, M. , … Ricklefs, F. L. (2021). Genome‐wide methylation profiling of glioblastoma cell‐derived extracellular vesicle DNA allows tumor classification. Neuro‐oncol, 23, 1087–1099. 33508126 10.1093/neuonc/noab012 PMC8673443

[jev212404-bib-0277] Marie, P. P. , Fan, S. J. , Mason, J. , Wells, A. , Mendes, C. C. , Wainwright, S. M. , Scott, S. , Fischer, R. , Harris, A. L. , Wilson, C. , & Goberdhan, D. C. I. (2023). Accessory ESCRT‐III proteins are conserved and selective regulators of Rab11a‐exosome formation. Journal of Extracellular Vesicles, 12, e12311. 36872252 10.1002/jev2.12311 PMC9986085

[jev212404-bib-0278] Martin‐Jaular, L. , Nevo, N. , Schessner, J. P. , Tkach, M. , Jouve, M. , Dingli, F. , Loew, D. , Witwer, K. W. , Ostrowski, M. , Borner, G. H. H. , & Théry, C. (2021). Unbiased proteomic profiling of host cell extracellular vesicle composition and dynamics upon HIV‐1 infection. Embo Journal, 40, e105492. 33709510 10.15252/embj.2020105492 PMC8047442

[jev212404-bib-0279] Martinez‐Bartolome, S. , Deutsch, E. W. , Binz, P. A. , Jones, A. R. , Eisenacher, M. , Mayer, G. , Campos, A. , Canals, F. , Bech‐Serra, J. J. , Carrascal, M. , Gay, M. , Paradela, A. , Navajas, R. , Marcilla, M. , Hernaez, M. L. , Gutierrez‐Blazquez, M. D. , Velarde, L. F. , Aloria, K. , Beaskoetxea, J. , … Albar, J. P. (2013). Guidelines for reporting quantitative mass spectrometry based experiments in proteomics. Journal of Proteomics, 95, 84–88. 23500130 10.1016/j.jprot.2013.02.026

[jev212404-bib-0280] Mateescu, B. , Kowal, E. J. , van Balkom, B. W. , Bartel, S. , Bhattacharyya, S. N. , Buzas, E. I. , Buck, A. H. , de Candia, P. , Chow, F. W. , Das, S. , Driedonks, T. A. , Fernandez‐Messina, L. , Haderk, F. , Hill, A. F. , Jones, J. C. , Van Keuren‐Jensen, K. R. , Lai, C. P. , Lasser, C. , Liegro, I. D. , … Nolte‐’t Hoen, E. N. (2017). Obstacles and opportunities in the functional analysis of extracellular vesicle RNA – An ISEV position paper. Journal of Extracellular Vesicles, 6, 1286095. 28326170 10.1080/20013078.2017.1286095 PMC5345583

[jev212404-bib-0281] Mathieu, M. , Martin‐Jaular, L. , Lavieu, G. , & Théry, C. (2019). Specificities of secretion and uptake of exosomes and other extracellular vesicles for cell‐to‐cell communication. Nature Cell Biology, 21, 9–17. 30602770 10.1038/s41556-018-0250-9

[jev212404-bib-0282] Mathieu, M. , Névo, N. , Jouve, M. , Valenzuela, J. I. , Maurin, M. , Verweij, F. J. , Palmulli, R. , Lankar, D. , Dingli, F. , Loew, D. , Rubinstein, E. , Boncompain, G. , Perez, F. , & Théry, C. (2021). Specificities of exosome versus small ectosome secretion revealed by live intracellular tracking of CD63 and CD9. Nature Communications, 12, 4389. 10.1038/s41467-021-24384-2 PMC8289845 34282141

[jev212404-bib-0283] Mattsson, N. , Andreasson, U. , Persson, S. , Arai, H. , Batish, S. D. , Bernardini, S. , Bocchio‐Chiavetto, L. , Blankenstein, M. A. , Carrillo, M. C. , Chalbot, S. , Coart, E. , Chiasserini, D. , Cutler, N. , Dahlfors, G. , Duller, S. , Fagan, A. M. , Forlenza, O. , Frisoni, G. B. , Galasko, D. , … Blennow, K. (2011). The Alzheimer's Association external quality control program for cerebrospinal fluid biomarkers. Alzheimers Dement, 7, 386–395.e6. 21784349 10.1016/j.jalz.2011.05.2243 PMC3710290

[jev212404-bib-0284] Matusek, T. , Wendler, F. , Polès, S. , Pizette, S. , D'Angelo, G. , Fürthauer, M. , & Thérond, P. P. (2014). The ESCRT machinery regulates the secretion and long‐range activity of Hedgehog. Nature, 516, 99–103. 25471885 10.1038/nature13847

[jev212404-bib-0285] McCann, J. V. , Bischoff, S. R. , Zhang, Y. , Cowley, D. O. , Sanchez‐Gonzalez, V. , Daaboul, G. D. , & Dudley, A. C. (2020). Reporter mice for isolating and auditing cell type‐specific extracellular vesicles in vivo. Genesis (New York, N.Y.: 2000), 58, e23369. 32543746 10.1002/dvg.23369 PMC7405599

[jev212404-bib-0286] McKenzie, A. J. , Hoshino, D. , Hong, N. H. , Cha, D. J. , Franklin, J. L. , Coffey, R. J. , Patton, J. G. , & Weaver, A. M. (2016). KRAS‐MEK signaling controls Ago2 sorting into exosomes. Cell Reports, 15, 978–987. 27117408 10.1016/j.celrep.2016.03.085 PMC4857875

[jev212404-bib-0287] McMillan, H. M. , & Kuehn, M. J. (2023). Proteomic profiling reveals distinct bacterial extracellular vesicle subpopulations with possibly unique functionality. Applied and Environmental Microbiology, 89, e0168622. 36533919 10.1128/aem.01686-22 PMC9888257

[jev212404-bib-0288] Mehanny, M. , Kroniger, T. , Koch, M. , Hoppstädter, J. , Becher, D. , Kiemer, A. K. , Lehr, C. M. , & Fuhrmann, G. (2022). Yields and immunomodulatory effects of pneumococcal membrane vesicles differ with the bacterial growth phase. Advanced Healthcare Materials, 11, e2101151. 34724354 10.1002/adhm.202101151 PMC11469037

[jev212404-bib-0289] Michael, B. N. R. , Kommoju, V. , Ganapathy, C. K , & Negi, V. S. (2019). Characterization of cell‐derived microparticles in synovial fluid and plasma of patients with rheumatoid arthritis. Rheumatology International, 39, 1377–1387. 31201512 10.1007/s00296-019-04337-1

[jev212404-bib-0290] Mihaly, J. , Deak, R. , Szigyarto, I. C. , Bota, A. , Beke‐Somfai, T. , & Varga, Z. (2017). Characterization of extracellular vesicles by IR spectroscopy: Fast and simple classification based on amide and CH stretching vibrations. Biochimica et Biophysica Acta (BBA) ‐ Biomembranes, 1859, 459–466. 27989744 10.1016/j.bbamem.2016.12.005

[jev212404-bib-0291] Mittelbrunn, M. , Gutiérrez‐Vázquez, C. , Villarroya‐Beltri, C. , González, S. , Sánchez‐Cabo, F. , González, M. Á. , Bernad, A. , & Sánchez‐Madrid, F. (2011). Unidirectional transfer of microRNA‐loaded exosomes from T cells to antigen‐presenting cells. Nature Communications, 2, 282. 10.1038/ncomms1285 PMC3104548 21505438

[jev212404-bib-0292] Möhrmann, L. , Huang, H. J. , Hong, D. S. , Tsimberidou, A. M. , Fu, S. , Piha‐Paul, S. A. , Subbiah, V. , Karp, D. D. , Naing, A. , Krug, A. , Enderle, D. , Priewasser, T. , Noerholm, M. , Eitan, E. , Coticchia, C. , Stoll, G. , Jordan, L. M. , Eng, C. , Kopetz, E. S. , … Janku, F. (2018). Liquid biopsies using plasma exosomal nucleic acids and plasma cell‐free DNA compared with clinical outcomes of patients with advanced cancers. Clinical Cancer Research, 24, 181–188. 29051321 10.1158/1078-0432.CCR-17-2007 PMC5754253

[jev212404-bib-0293] Mondal, A. , Ashiq, K. A. , Phulpagar, P. , Singh, D. K. , & Shiras, A. (2019). Effective visualization and easy tracking of extracellular vesicles in glioma cells. Biological Procedures Online, 21, 4. 30918474 10.1186/s12575-019-0092-2 PMC6419365

[jev212404-bib-0294] Montero Llopis, P. , Senft, R. A. , Ross‐Elliott, T. J. , Stephansky, R. , Keeley, D. P. , Koshar, P. , Marqués, G. , Gao, Y. S. , Carlson, B. R. , Pengo, T. , Sanders, M. A. , Cameron, L. A. , & Itano, M. S. (2021). Best practices and tools for reporting reproducible fluorescence microscopy methods. Nature Methods, 18(12), 1463–1476. 34099930 10.1038/s41592-021-01156-w

[jev212404-bib-0295] Morales‐Kastresana, A. , Musich, T. A. , Welsh, J. A. , Telford, W. , Demberg, T. , Wood, J. C. S. , Bigos, M. , Ross, C. D. , Kachynski, A. , Dean, A. , Felton, E. J. , van Dyke, J. , Tigges, J. , Toxavidis, V. , Parks, D. R. , Overton, W. R. , Kesarwala, A. H. , Freeman, G. J. , Rosner, A. , … Jones, J. C. (2019). High‐fidelity detection and sorting of nanoscale vesicles in viral disease and cancer. Journal of Extracellular Vesicles, 8, 1597603. 31258878 10.1080/20013078.2019.1597603 PMC6586126

[jev212404-bib-0296] Morikawa, Y. , Takahashi, N. , Kamiyama, K. , Nishimori, K. , Nishikawa, Y. , Morita, S. , Kobayashi, M. , Fukushima, S. , Yokoi, S. , Mikami, D. , Kimura, H. , Kasuno, K. , Yashiki, T. , Naiki, H. , Hara, M. , & Iwano, M. (2019). Elevated levels of urinary extracellular vesicle fibroblast‐specific protein 1 in patients with active crescentic glomerulonephritis. Nephron, 141, 177–187. 30540988 10.1159/000495217

[jev212404-bib-0297] Mukhopadhya, A. , Santoro, J. , Moran, B. , Useckaite, Z. , & O'Driscoll, L. (2021). Optimisation and comparison of orthogonal methods for separation and characterisation of extracellular vesicles to investigate how representative infant milk formula is of milk. Food Chemistry, 353, 129309. 33725545 10.1016/j.foodchem.2021.129309

[jev212404-bib-0298] Mukhopadhya, A. , Santoro, J. , & O'Driscoll, L. (2021). Extracellular vesicle separation from milk and infant milk formula using acid precipitation and ultracentrifugation. STAR Protocols, 2, 100821. 34568843 10.1016/j.xpro.2021.100821 PMC8449126

[jev212404-bib-0299] Musicò, A. , Zenatelli, R. , Romano, M. , Zendrini, A. , Alacqua, S. , Tassoni, S. , Paolini, L. , Urbinati, C. , Rusnati, M. , Bergese, P. , Pomarico, G. , & Radeghieri, A. (2023). Surface functionalization of extracellular vesicle nanoparticles with antibodies: A first study on the protein corona “variable”. Nanoscale Advances, 5, 4703–4717. 37705771 10.1039/d3na00280b PMC10496878

[jev212404-bib-0300] Mustonen, A. M. , Capra, J. , Rilla, K. , Lehenkari, P. , Oikari, S. , Kääriäinen, T. , Joukainen, A. , Kröger, H. , Paakkonen, T. , Matilainen, J. , & Nieminen, P. (2021). Characterization of hyaluronan‐coated extracellular vesicles in synovial fluid of patients with osteoarthritis and rheumatoid arthritis. BMC Musculoskeletal Disorders [Electronic Resource], 22, 247. 33676459 10.1186/s12891-021-04115-w PMC7937210

[jev212404-bib-0301] Nakai, W. , Yoshida, T. , Diez, D. , Miyatake, Y. , Nishibu, T. , Imawaka, N. , Naruse, K. , Sadamura, Y. , & Hanayama, R. (2016). A novel affinity‐based method for the isolation of highly purified extracellular vesicles. Scientific Reports, 6, 33935. 27659060 10.1038/srep33935 PMC5034288

[jev212404-bib-0302] Nakayasu, E. S. , Gritsenko, M. , Piehowski, P. D. , Gao, Y. , Orton, D. J. , Schepmoes, A. A. , Fillmore, T. L. , Frohnert, B. I. , Rewers, M. , Krischer, J. P. , Ansong, C. , Suchy‐Dicey, A. M. , Evans‐Molina, C. , Qian, W. J. , Webb‐Robertson, B. M. , & Metz, T. O. (2021). Tutorial: Best practices and considerations for mass‐spectrometry‐based protein biomarker discovery and validation. Nature Protocols, 16, 3737–3760. 34244696 10.1038/s41596-021-00566-6 PMC8830262

[jev212404-bib-0303] Navazesh, M. (1993). Methods for collecting saliva. Annals of the New York Academy of Sciences, 694, 72–77. 8215087 10.1111/j.1749-6632.1993.tb18343.x

[jev212404-bib-0304] Neckles, V. N. , Morton, M. C. , Holmberg, J. C. , Sokolov, A. M. , Nottoli, T. , Liu, D. , & Feliciano, D. M. (2019). A transgenic inducible GFP extracellular‐vesicle reporter (TIGER) mouse illuminates neonatal cortical astrocytes as a source of immunomodulatory extracellular vesicles. Scientific Reports, 9, 3094. 30816224 10.1038/s41598-019-39679-0 PMC6395689

[jev212404-bib-0305] Newman, L A. , Useckaite, Z. , & Rowland, A. (2022). Addressing MISEV guidance using targeted LC‐MS/MS: A method for the detection and quantification of extracellular vesicle‐enriched and contaminant protein markers from blood. Journal of Extracellular Biology, 1, e56. 10.1002/jex2.56 PMC11080780 38938773

[jev212404-bib-0306] Ngamchuea, K. , Chaisiwamongkhol, K. , Batchelor‐McAuley, C. , & Compton, R. G. (2017). Chemical analysis in saliva and the search for salivary biomarkers – A tutorial review. Analyst, 143, 81–99. 29149225 10.1039/c7an01571b

[jev212404-bib-0307] Nguyen, V. V. T. , Witwer, K. W. , Verhaar, M. C. , Strunk, D. , & van Balkom, B. W. M. (2020). Functional assays to assess the therapeutic potential of extracellular vesicles. Journal of Extracellular Vesicles, 10, e12033. 33708360 10.1002/jev2.12033 PMC7890556

[jev212404-bib-0308] Nieuwland, R. , Falcon‐Perez, J. M. , Thery, C. , & Witwer, K. W. (2020). Rigor and standardization of extracellular vesicle research: Paving the road towards robustness. Journal of Extracellular Vesicles, 10, e12037. 33343835 10.1002/jev2.12037 PMC7735957

[jev212404-bib-0309] Nikonorova, I. A. , Wang, J. , Cope, A. L. , Tilton, P. E. , Power, K. M. , Walsh, J. D. , Akella, J. S. , Krauchunas, A. R. , Shah, P. , & Barr, M. M. (2022). Isolation, profiling, and tracking of extracellular vesicle cargo in *Caenorhabditis elegans* . Current Biology, 32(9), 1924–1936.e6. 35334227 10.1016/j.cub.2022.03.005 PMC9491618

[jev212404-bib-0310] Nizamudeen, Z. , Markus, R. , Lodge, R. , Parmenter, C. , Platt, M. , Chakrabarti, L. , & Sottile, V. (2018). Rapid and accurate analysis of stem cell‐derived extracellular vesicles with super resolution microscopy and live imaging. Biochimica et Biophysica Acta (BBA) ‐ Molecular Cell Research, 1865, 1891–1900. 30290236 10.1016/j.bbamcr.2018.09.008 PMC6203808

[jev212404-bib-0311] Nonaka, T. , & Wong, D. T. W. (2022). Saliva diagnostics. Annual Review of Analytical Chemistry, 15, 107–121. 10.1146/annurev-anchem-061020-123959 PMC9348814 35696523

[jev212404-bib-0312] Nørgård, M. Ø. , Steffensen, L. B. , Hansen, D. R. , Füchtbauer, E. M. , Engelund, M. B. , Dimke, H. , Andersen, D. C. , & Svenningsen, P. (2022). A new transgene mouse model using an extravesicular EGFP tag enables affinity isolation of cell‐specific extracellular vesicles. Scientific Reports, 12, 496. 35017633 10.1038/s41598-021-04512-0 PMC8752749

[jev212404-bib-0313] Norman, M. , Ter‐Ovanesyan, D. , Trieu, W. , Lazarovits, R. , Kowal, E. J. K. , Lee, J. H. , Chen‐Plotkin, A. S. , Regev, A. , Church, G. M. , & Walt, D. R. (2021). L1CAM is not associated with extracellular vesicles in human cerebrospinal fluid or plasma. Nature Methods, 18, 631–634. 34092791 10.1038/s41592-021-01174-8 PMC9075416

[jev212404-bib-0314] Obeid, S. , Sung, P. S. , Roy, B. L. , Chou, M. L. , Hsieh, S. L. , Elie‐Caille, C. , Burnouf, T. , & Boireau, W. (2019). NanoBioAnalytical characterization of extracellular vesicles in 75‐nm nanofiltered human plasma for transfusion: A tool to improve transfusion safety. Nanomedicine, 20, 101977. 30878658 10.1016/j.nano.2019.02.026

[jev212404-bib-0315] Ogawa, Y. , Kanai‐Azuma, M. , Akimoto, Y. , Kawakami, H. , & Yanoshita, R. (2008). Exosome‐like vesicles with dipeptidyl peptidase IV in human saliva. Biological & Pharmaceutical Bulletin, 31, 1059–1062. 18520029 10.1248/bpb.31.1059

[jev212404-bib-0316] Oleksiuk, O. , Abba, M. , Tezcan, K. C. , Schaufler, W. , Bestvater, F. , Patil, N. , Birk, U. , Hafner, M. , Altevogt, P. , Cremer, C. , & Allgayer, H. (2015). Single‐Molecule Localization Microscopy allows for the analysis of cancer metastasis‐specific miRNA distribution on the nanoscale. Oncotarget, 6, 44745–44757. 26561203 10.18632/oncotarget.6297 PMC4792589

[jev212404-bib-0317] Oliveira, D. L. , Nakayasu, E. S. , Joffe, L. S. , Guimarães, A. J. , Sobreira, T. J. , Nosanchuk, J. D. , Cordero, R. J. , Frases, S. , Casadevall, A. , Almeida, I. C. , Nimrichter, L. , & Rodrigues, M. L. (2010). Characterization of yeast extracellular vesicles: evidence for the participation of different pathways of cellular traffic in vesicle biogenesis. PLoS ONE, 5, e11113. 20559436 10.1371/journal.pone.0011113 PMC2885426

[jev212404-bib-0318] Osteikoetxea, X. , Sodar, B. , Nemeth, A. , Szabo‐Taylor, K. , Paloczi, K. , Vukman, K. V. , Tamasi, V. , Balogh, A. , Kittel, A. , Pallinger, E. , & Buzas, E. I. (2015). Differential detergent sensitivity of extracellular vesicle subpopulations. Organic & Biomolecular Chemistry, 13, 9775–9782. 26264754 10.1039/c5ob01451d

[jev212404-bib-0319] Ostrowski, M. , Carmo, N. B. , Krumeich, S. , Fanget, I. , Raposo, G. , Savina, A. , Moita, C. F. , Schauer, K. , Hume, A. N. , Freitas, R. P. , Goud, B. , Benaroch, P. , Hacohen, N. , Fukuda, M. , Desnos, C. , Seabra, M. C. , Darchen, F. , Amigorena, S. , Moita, L. F. , & Thery, C. (2010). Rab27a and Rab27b control different steps of the exosome secretion pathway. Nature Cell Biology, 12, 19–30, 1–13. 10.1038/ncb2000 19966785

[jev212404-bib-0320] Page, M. J. , Kell, D. B. , & Pretorius, E. (2022). The role of lipopolysaccharide‐induced cell signalling in chronic inflammation. Chronic Stress (Thousand Oaks), 6, 24705470221076390. 35155966 10.1177/24705470221076390 PMC8829728

[jev212404-bib-0321] Palma, J. , Yaddanapudi, S. C. , Pigati, L. , Havens, M. A. , Jeong, S. , Weiner, G. A. , Weimer, K. M. , Stern, B. , Hastings, M. L. , & Duelli, D. M. (2012). MicroRNAs are exported from malignant cells in customized particles. Nucleic Acids Research, 40, 9125–9138. 22772984 10.1093/nar/gks656 PMC3467054

[jev212404-bib-0322] Palmieri, V. , Lucchetti, D. , Gatto, I. , Maiorana, A. , Marcantoni, M. , Maulucci, G. , Papi, M. , Pola, R. , De Spirito, M. , & Sgambato, A. (2014). Dynamic light scattering for the characterization and counting of extracellular vesicles: A powerful noninvasive tool. Journal of Nanoparticle Research, 16, 2583.

[jev212404-bib-0323] Palviainen, M. , Saari, H. , Kärkkäinen, O. , Pekkinen, J. , Auriola, S. , Yliperttula, M. , Puhka, M. , Hanhineva, K. , & Siljander, P. R. (2019). Metabolic signature of extracellular vesicles depends on the cell culture conditions. Journal of Extracellular Vesicles, 8, 1596669. 31007875 10.1080/20013078.2019.1596669 PMC6461113

[jev212404-bib-0324] Palviainen, M. , Saraswat, M. , Varga, Z. , Kitka, D. , Neuvonen, M. , Puhka, M. , Joenväärä, S. , Renkonen, R. , Nieuwland, R. , Takatalo, M. , & Siljander, P. R. M. (2020). Extracellular vesicles from human plasma and serum are carriers of extravesicular cargo‐Implications for biomarker discovery. PLoS ONE, 15, e0236439. 32813744 10.1371/journal.pone.0236439 PMC7446890

[jev212404-bib-0325] Panagopoulou, M. S. , Wark, A. W. , Birch, D. J. S. , & Gregory, C. D. (2020). Phenotypic analysis of extracellular vesicles: A review on the applications of fluorescence. Journal of Extracellular Vesicles, 9, 1710020. 32002172 10.1080/20013078.2019.1710020 PMC6968689

[jev212404-bib-0326] Paolini, L. , Federici, S. , Consoli, G. , Arceri, D. , Radeghieri, A. , Alessandri, I. , & Bergese, P. (2020). Fourier‐transform Infrared (FT‐IR) spectroscopy fingerprints subpopulations of extracellular vesicles of different sizes and cellular origin. Journal of Extracellular Vesicles, 9, 1741174. 32341767 10.1080/20013078.2020.1741174 PMC7170381

[jev212404-bib-0327] Paolini, L. , Zendrini, A. , Di Noto, G. , Busatto, S. , Lottini, E. , Radeghieri, A. , Dossi, A. , Caneschi, A. , Ricotta, D. , & Bergese, P. (2016). Residual matrix from different separation techniques impacts exosome biological activity. Scientific Reports, 6, 23550. 27009329 10.1038/srep23550 PMC4806376

[jev212404-bib-0328] Parisse, P. , Rago, I. , Ulloa Severino, L. , Perissinotto, F. , Ambrosetti, E. , Paoletti, P. , Ricci, M. , Beltrami, A. P. , Cesselli, D. , & Casalis, L. (2017). Atomic force microscopy analysis of extracellular vesicles. European Biophysics Journal, 46, 813–820. 28866771 10.1007/s00249-017-1252-4

[jev212404-bib-0329] Park, J. , Hwang, M. , Choi, B. , Jeong, H. , Jung, J. H. , Kim, H. K. , Hong, S. , Park, J. H. , & Choi, Y. (2017). Exosome classification by pattern analysis of surface‐enhanced raman spectroscopy data for lung cancer diagnosis. Analytical Chemistry, 89, 6695–6701. 28541032 10.1021/acs.analchem.7b00911

[jev212404-bib-0330] "Particle size analysis — Dynamic light scattering (DLS)." . (2017). In ISO.

[jev212404-bib-0331] Peinado, H. , Alečković, M. , Lavotshkin, S. , Matei, I. , Costa‐Silva, B. , Moreno‐Bueno, G. , Hergueta‐Redondo, M. , Williams, C. , García‐Santos, G. , Ghajar, C. , Nitadori‐Hoshino, A. , Hoffman, C. , Badal, K. , Garcia, B. A. , Callahan, M. K. , Yuan, J. , Martins, V. R. , Skog, J. , Kaplan, R. N. , … Lyden, D. (2012). Melanoma exosomes educate bone marrow progenitor cells toward a pro‐metastatic phenotype through MET. Nature Medicine, 18, 883–891. 10.1038/nm.2753 PMC3645291 22635005

[jev212404-bib-0332] Perez‐Gonzalez, R. , Gauthier, S. A. , Kumar, A. , & Levy, E. (2012). The exosome secretory pathway transports amyloid precursor protein carboxyl‐terminal fragments from the cell into the brain extracellular space. Journal of Biological Chemistry, 287, 43108–43115. 23129776 10.1074/jbc.M112.404467 PMC3522305

[jev212404-bib-0333] Perrin, P. , Janssen, L. , Janssen, H. , van den Broek, B. , Voortman, L. M. , van Elsland, D. , Berlin, I. , & Neefjes, J. (2021). Retrofusion of intralumenal MVB membranes parallels viral infection and coexists with exosome release. Current Biology, 31, 3884–3893.e4. 34237268 10.1016/j.cub.2021.06.022 PMC8445322

[jev212404-bib-0334] Pfeiler, S. , Thakur, M. , Grünauer, P. , Megens, R. T. A. , Joshi, U. , Coletti, R. , Samara, V. , Müller‐Stoy, G. , Ishikawa‐Ankerhold, H. , Stark, K. , Klingl, A. , Fröhlich, T. , Arnold, G. J. , Wörmann, S. , Bruns, C. J. , Algül, H. , Weber, C. , Massberg, S. , & Engelmann, B. (2019). CD36‐triggered cell invasion and persistent tissue colonization by tumor microvesicles during metastasis. Faseb Journal, 33, 1860–1872. 30207797 10.1096/fj.201800985R

[jev212404-bib-0335] Pham, T. C. , Jayasinghe, M. K. , Pham, T. T. , Yang, Y. , Wei, L. , Usman, W. M. , Chen, H. , Pirisinu, M. , Gong, J. , Kim, S. , Peng, B. , Wang, W. , Chan, C. , Ma, V. , Nguyen, N. T. H. , Kappei, D. , Nguyen, X. H. , Cho, W. C. , Shi, J. , & Le, M. T. N. (2021). Covalent conjugation of extracellular vesicles with peptides and nanobodies for targeted therapeutic delivery. Journal of Extracellular Vesicles, 10, e12057. 33643546 10.1002/jev2.12057 PMC7886705

[jev212404-bib-0336] Piontek, M. C. , Lira, R. B. , & Roos, W. H. (2021). Active probing of the mechanical properties of biological and synthetic vesicles. Biochimica et Biophysica Acta (BBA) ‐ General Subjects, 1865, 129486. 31734458 10.1016/j.bbagen.2019.129486

[jev212404-bib-0337] Pisitkun, T. , Shen, R. F. , & Knepper, M. A. (2004). Identification and proteomic profiling of exosomes in human urine. PNAS, 101, 13368–13373. 15326289 10.1073/pnas.0403453101 PMC516573

[jev212404-bib-0338] Pleet, M. L. , Cook, S. , Tang, V. A. , Stack, E. , Ford, V. J. , Lannigan, J. , Do, N. , Wenger, E. , Fraikin, J. L. , Jacobson, S. , Jones, J. C. , & Welsh, J. A. (2023). Extracellular vesicle refractive index derivation utilizing orthogonal characterization. Nano Letters, 23(20), 9195–9202. 37788377 10.1021/acs.nanolett.3c00562 PMC10603804

[jev212404-bib-0339] Pocsfalvi, G. , Stanly, C. , Vilasi, A. , Fiume, I. , Capasso, G. , Turiak, L. , Buzas, E. I. , & Vekey, K. (2016). Mass spectrometry of extracellular vesicles. Mass Spectrometry Reviews, 35, 3–21. 25705034 10.1002/mas.21457

[jev212404-bib-0340] Pocsfalvi, G. , Stanly, C. , Fiume, I. , & Vékey, K. (2016). Chromatography and its hyphenation to mass spectrometry for extracellular vesicle analysis. Journal of Chromatography A, 1439, 26–41. 26830636 10.1016/j.chroma.2016.01.017

[jev212404-bib-0341] Polanco, J. C. , Li, C. , Durisic, N. , Sullivan, R. , & Götz, J. (2018). Exosomes taken up by neurons hijack the endosomal pathway to spread to interconnected neurons. Acta Neuropathologica Communications, 6, 10. 29448966 10.1186/s40478-018-0514-4 PMC5815204

[jev212404-bib-0342] Polanco, J. C. , Scicluna, B. J. , Hill, A. F. , & Götz, J. (2016). Extracellular vesicles isolated from the brains of rTg4510 mice seed tau protein aggregation in a threshold‐dependent manner. Journal of Biological Chemistry, 291, 12445–12466. 27030011 10.1074/jbc.M115.709485 PMC4933440

[jev212404-bib-0343] Prados‐Rosales, R. , Baena, A. , Martinez, L. R. , Luque‐Garcia, J. , Kalscheuer, R. , Veeraraghavan, U. , Camara, C. , Nosanchuk, J. D. , Besra, G. S. , Chen, B. , Jimenez, J. , Glatman‐Freedman, A. , Jacobs, W. R., Jr. , Porcelli, S. A. , & Casadevall, A. (2011). Mycobacteria release active membrane vesicles that modulate immune responses in a TLR2‐dependent manner in mice. Journal of Clinical Investigation, 121, 1471–1483. 21364279 10.1172/JCI44261 PMC3069770

[jev212404-bib-0344] Preußer, C. , Stelter, K. , Tertel, T. , Linder, M. , Helmprobst, F. , Szymanski, W. , Graumann, J. , Giebel, B. , Reinartz, S. , Müller, R. , Weber, G. , & von Strandmann, E. P. (2022). Isolation of native EVs from primary biofluids—Free‐flow electrophoresis as a novel approach to purify ascites‐derived EVs. Journal of Extracellular Biology, 1, e71. 10.1002/jex2.71 PMC11080702 38938598

[jev212404-bib-0345] Provencher, S. W. (1982). A constrained regularization method for inverting data represented by linear algebraic or integral equations. Computer Physics Communications, 27, 213–227.

[jev212404-bib-0346] Puca, L. , Chastagner, P. , Meas‐Yedid, V. , Israël, A. , & Brou, C. (2013). Α‐arrestin 1 (ARRDC1) and β‐arrestins cooperate to mediate Notch degradation in mammals. Journal of Cell Science, 126, 4457–4468. 23886940 10.1242/jcs.130500

[jev212404-bib-0347] Pucci, F. , Garris, C. , Lai, C. P. , Newton, A. , Pfirschke, C. , Engblom, C. , Alvarez, D. , Sprachman, M. , Evavold, C. , Magnuson, A. , von Andrian, U. H. , Glatz, K. , Breakefield, X. O. , Mempel, T. R. , Weissleder, R. , & Pittet, M. J. (2016). SCS macrophages suppress melanoma by restricting tumor‐derived vesicle‐B cell interactions. Science, 352, 242–246. 26989197 10.1126/science.aaf1328 PMC4960636

[jev212404-bib-0348] Pužar Dominkuš, P. , Stenovec, M. , Sitar, S. , Lasič, E. , Zorec, R. , Plemenitaš, A. , Žagar, E. , Kreft, M. , & Lenassi, M. (2018). PKH26 labeling of extracellular vesicles: Characterization and cellular internalization of contaminating PKH26 nanoparticles. Biochimica et Biophysica Acta (BBA) ‐ Biomembranes, 1860, 1350–1361. 29551275 10.1016/j.bbamem.2018.03.013

[jev212404-bib-0349] Qu, X. , Li, Q. , Yang, J. , Zhao, H. , Wang, F. , Zhang, F. , Zhang, S. , Zhang, H. , Wang, R. , Wang, Q. , Wang, Q. , Li, G. , Peng, X. , Zhou, X. , Hao, Y. , Zhu, J. , & Xiao, W. (2019). Double‐stranded DNA in exosomes of malignant pleural effusions as a novel DNA source for EGFR mutation detection in lung adenocarcinoma. Frontiers in Oncology, 9, 931. 31608233 10.3389/fonc.2019.00931 PMC6773809

[jev212404-bib-0350] Rad, M. , Kakoie, S. , Brojeni, F. N , & Pourdamghan, N. (2010). Effect of long‐term smoking on whole‐mouth salivary flow rate and oral Health. Journal of Dental Research, Dental Clinics, Dental Prospects, 4, 110–114. 23346336 10.5681/joddd.2010.028 PMC3429961

[jev212404-bib-0351] Radeghieri, A. , Alacqua, S. , Zendrini, A. , Previcini, V. , Todaro, F. , Martini, G. , Ricotta, D. , & Bergese, P. (2022). Active antithrombin glycoforms are selectively physiosorbed on plasma extracellular vesicles. Journal of Extracellular Biology, 1, e57. 10.1002/jex2.57 PMC11080738 38938771

[jev212404-bib-0352] Ragni, E. , Perucca Orfei, C. , De Luca, P. , Lugano, G. , Viganò, M. , Colombini, A. , Valli, F. , Zacchetti, D. , Bollati, V. , & de Girolamo, L. (2019). Interaction with hyaluronan matrix and miRNA cargo as contributors for in vitro potential of mesenchymal stem cell‐derived extracellular vesicles in a model of human osteoarthritic synoviocytes. Stem Cell Research & Therapy, 10, 109. 30922413 10.1186/s13287-019-1215-z PMC6440078

[jev212404-bib-0353] Rahman, M. M. , Shimizu, K. , Yamauchi, M. , Takase, H. , Ugawa, S. , Okada, A. , & Inoshima, Y. (2019). Acidification effects on isolation of extracellular vesicles from bovine milk. PLoS ONE, 14, e0222613. 31525238 10.1371/journal.pone.0222613 PMC6746375

[jev212404-bib-0354] Raj, A. , Kato, C. , Witek, H. A. , & Hamaguchi, H. (2020). Toward standardization of Raman spectroscopy: Accurate wavenumber and intensity calibration using rotational Raman spectra of H‐2, HD, D‐2, and vibration‐rotation spectrum of O‐2. Journal of Raman Spectroscopy, 51, 2066–2082.

[jev212404-bib-0355] Ramirez‐Garrastacho, M. , Bajo‐Santos, C. , Line, A. , Martens‐Uzunova, E. S. , de la Fuente, J. M. , Moros, M. , Soekmadji, C. , Tasken, K. A. , & Llorente, A. (2022). Extracellular vesicles as a source of prostate cancer biomarkers in liquid biopsies: A decade of research. British Journal of Cancer, 126, 331–350. 34811504 10.1038/s41416-021-01610-8 PMC8810769

[jev212404-bib-0356] Rao, P. , Benito, E. , & Fischer, A. (2013). MicroRNAs as biomarkers for CNS disease. Frontiers in Molecular Neuroscience, 6, 39. 24324397 10.3389/fnmol.2013.00039 PMC3840814

[jev212404-bib-0357] Raposo, G. , Nijman, H. W. , Stoorvogel, W. , Liejendekker, R. , Harding, C. V. , Melief, C. J. , & Geuze, H. J. (1996). B lymphocytes secrete antigen‐presenting vesicles. Journal of Experimental Medicine, 183, 1161–1172. 8642258 10.1084/jem.183.3.1161 PMC2192324

[jev212404-bib-0358] Razzauti, A. , & Laurent, P. (2021). Ectocytosis prevents accumulation of ciliary cargo in *C. elegans* sensory neurons. Elife, 10, e67670. 34533135 10.7554/eLife.67670 PMC8492061

[jev212404-bib-0359] Ridder, K. , Keller, S. , Dams, M. , Rupp, A. K. , Schlaudraff, J. , del Turco, D. , Starmann, J. , Macas, J. , Karpova, D. , Devraj, K. , Depboylu, C. , Landfried, B. , Arnold, B. , Plate, K. H. , Höglinger, G. , Sültmann, H. , Altevogt, P. , & Momma, S. (2014). Extracellular vesicle‐mediated transfer of genetic information between the hematopoietic system and the brain in response to inflammation. Plos Biology, 12, e1001874. 24893313 10.1371/journal.pbio.1001874 PMC4043485

[jev212404-bib-0360] Ridolfi, A. , Brucale, M. , Montis, C. , Caselli, L. , Paolini, L. , Borup, A. , Boysen, A. T. , Loria, F. , van Herwijnen, M. J. C. , Kleinjan, M. , Nejsum, P. , Zarovni, N. , Wauben, M. H. M. , Berti, D. , Bergese, P. , & Valle, F. (2020). AFM‐based high‐throughput nanomechanical screening of single extracellular vesicles. Analytical Chemistry, 92, 10274–10282. 32631050 10.1021/acs.analchem.9b05716

[jev212404-bib-0361] Ridolfi, A. , Caselli, L. , Baldoni, M. , Montis, C. , Mercuri, F. , Berti, D. , Valle, F. , & Brucale, M. (2021). Stiffness of fluid and gel phase lipid nanovesicles: Weighting the contributions of membrane bending modulus and luminal pressurization. Langmuir, 37, 12027–12037. 34610740 10.1021/acs.langmuir.1c01660

[jev212404-bib-0362] Riekse, R. G. , Li, G. , Petrie, E. C. , Leverenz, J. B. , Vavrek, D. , Vuletic, S. , Albers, J. J. , Montine, T. J. , Lee, V. M. , Lee, M. , Seubert, P. , Galasko, D. , Schellenberg, G. D. , Hazzard, W. R. , & Peskind, E. R. (2006). Effect of statins on Alzheimer's disease biomarkers in cerebrospinal fluid. Journal of Alzheimer's Disease, 10, 399–406. 10.3233/jad-2006-10408 17183151

[jev212404-bib-0363] Rikkert, L. G. , Nieuwland, R. , Terstappen, L. , & Coumans, F. A. W. (2019). Quality of extracellular vesicle images by transmission electron microscopy is operator and protocol dependent. Journal of Extracellular Vesicles, 8, 1555419. 30651939 10.1080/20013078.2018.1555419 PMC6327933

[jev212404-bib-0364] Roberts‐Dalton, H. D. , Cocks, A. , Falcon‐Perez, J. M. , Sayers, E. J. , Webber, J. P. , Watson, P. , Clayton, A. , & Jones, A. T. (2017). Fluorescence labelling of extracellular vesicles using a novel thiol‐based strategy for quantitative analysis of cellular delivery and intracellular traffic. Nanoscale, 9, 13693–13706. 28880029 10.1039/c7nr04128d

[jev212404-bib-0365] Rodrigues, A. D. , van Dyk, M. , Sorich, M. J. , Fahmy, A. , Useckaite, Z. , Newman, L. A. , Kapetas, A. J. , Mounzer, R. , Wood, L. S. , Johnson, J. G. , & Rowland, A. (2021). Exploring the use of serum‐derived small extracellular vesicles as liquid biopsy to study the induction of hepatic cytochromes P450 and organic anion transporting polypeptides. Clinical Pharmacology & Therapeutics, 110, 248–258. 33792897 10.1002/cpt.2244

[jev212404-bib-0366] Rojalin, T. , Koster, H. J. , Liu, J. , Mizenko, R. R. , Tran, D. , Wachsmann‐Hogiu, S. , & Carney, R. P. (2020). Hybrid nanoplasmonic porous biomaterial scaffold for liquid biopsy diagnostics using extracellular vesicles. ACS Sensors, 5, 2820–2833. 32935542 10.1021/acssensors.0c00953 PMC7522966

[jev212404-bib-0367] Romanò, S. , Di Giacinto, F. , Primiano, A. , Gervasoni, J. , Mazzini, A. , Papi, M. , Urbani, A. , Serafino, A. , De Spirito, M. , Krasnowska, E. K. , & Ciasca, G. (2022). Label‐free spectroscopic characterization of exosomes reveals cancer cell differentiation. Analytica Chimica Acta, 1192, 339359. 35057944 10.1016/j.aca.2021.339359

[jev212404-bib-0368] Rostgaard, N. , Olsen, M. H. , Ottenheijm, M. , Drici, L. , Simonsen, A. H. , Plomgaard, P. , Gredal, H. , Poulsen, H. H. , Zetterberg, H. , Blennow, K. , Hasselbalch, S. G. , MacAulay, N. , & Juhler, M. (2023). Differential proteomic profile of lumbar and ventricular cerebrospinal fluid. Fluids Barriers CNS, 20, 6. 36670437 10.1186/s12987-022-00405-0 PMC9863210

[jev212404-bib-0369] Roux, Q. , Van Deun, J. , Dedeyne, S. , & Hendrix, A. (2020). The EV‐TRACK summary add‐on: Integration of experimental information in databases to ensure comprehensive interpretation of biological knowledge on extracellular vesicles. Journal of Extracellular Vesicles, 9, 1699367. 32002166 10.1080/20013078.2019.1699367 PMC6968489

[jev212404-bib-0370] Royo, F. , Cossío, U. , Ruiz de Angulo, A. , Llop, J. , & Falcon‐Perez, J. M. (2019). Modification of the glycosylation of extracellular vesicles alters their biodistribution in mice. Nanoscale, 11, 1531–1537. 30623961 10.1039/c8nr03900c

[jev212404-bib-0371] Royo, F. , Théry, C. , Falcón‐Pérez, J. M. , Nieuwland, R. , & Witwer, K. W. (2020). Methods for separation and characterization of extracellular vesicles: results of a worldwide survey performed by the ISEV rigor and standardization subcommittee. Cells, 9, 1955. 32854228 10.3390/cells9091955 PMC7563174

[jev212404-bib-0372] Rufino‐Ramos, D. , Lule, S. , Mahjoum, S. , Ughetto, S. , Cristopher Bragg, D. , Pereira de Almeida, L. , Breakefield, X. O. , & Breyne, K. (2022). Using genetically modified extracellular vesicles as a non‐invasive strategy to evaluate brain‐specific cargo. Biomaterials, 281, 121366. 35033904 10.1016/j.biomaterials.2022.121366 PMC8886823

[jev212404-bib-0373] Russell, A. E. , Sneider, A. , Witwer, K. W. , Bergese, P. , Bhattacharyya, S. N. , Cocks, A. , Cocucci, E. , Erdbrügger, U. , Falcon‐Perez, J. M. , Freeman, D. W. , Gallagher, T. M. , Hu, S. , Huang, Y. , Jay, S. M. , Kano, S. I. , Lavieu, G. , Leszczynska, A. , Llorente, A. M. , Lu, Q. , … Vader, P. (2019). Biological membranes in EV biogenesis, stability, uptake, and cargo transfer: an ISEV position paper arising from the ISEV membranes and EVs workshop. Journal of Extracellular Vesicles, 8, 1684862. 31762963 10.1080/20013078.2019.1684862 PMC6853251

[jev212404-bib-0374] Russell, J. C. , Kim, T. K. , Noori, A. , Merrihew, G. E. , Robbins, J. E. , Golubeva, A. , Wang, K. , MacCoss, M. J. , & Kaeberlein, M. (2020). Composition of *Caenorhabditis elegans* extracellular vesicles suggests roles in metabolism, immunity, and aging. Geroscience, 42, 1133–1145. 32578074 10.1007/s11357-020-00204-1 PMC7394990

[jev212404-bib-0375] Rust, M. J. , Bates, M. , & Zhuang, X. W. (2006). Sub‐diffraction‐limit imaging by stochastic optical reconstruction microscopy (STORM). Nature Methods, 3, 793–795. 16896339 10.1038/nmeth929 PMC2700296

[jev212404-bib-0376] Rüwald, J. M. , Randau, T. M. , Hilgers, C. , Masson, W. , Irsen, S. , Eymael, R. L. , Kohlhof, H. , Gravius, S. , Burger, C. , Wirtz, D. C. , & Schildberg, F. A. (2020). Extracellular vesicle isolation and characterization from periprosthetic joint synovial fluid in revision total joint arthroplasty. Journal of Clinical Medicine, 9, 516. 32075029 10.3390/jcm9020516 PMC7074102

[jev212404-bib-0377] Saari, H. , Pusa, R. , Marttila, H. , Yliperttula, M. , & Laitinen, S. (2023). Development of tandem cation exchange chromatography for high purity extracellular vesicle isolation: The effect of ligand steric availability. Journal of Chromatography A, 1707, 464293. 37579702 10.1016/j.chroma.2023.464293

[jev212404-bib-0378] Saari, H. , Turunen, T. , Lõhmus, A. , Turunen, M. , Jalasvuori, M. , Butcher, S. J. , Ylä‐Herttuala, S. , Viitala, T. , Cerullo, V. , Siljander, P. R. M. , & Yliperttula, M. (2020). Extracellular vesicles provide a capsid‐free vector for oncolytic adenoviral DNA delivery. Journal of Extracellular Vesicles, 9, 1747206. 32363012 10.1080/20013078.2020.1747206 PMC7178890

[jev212404-bib-0379] Saludes, J. P. , Morton, L. A. , Ghosh, N. , Beninson, L. A. , Chapman, E. R. , Fleshner, M. , & Yin, H. (2012). Detection of highly curved membrane surfaces using a cyclic peptide derived from synaptotagmin‐I. Acs Chemical Biology, 7, 1629–1635. 22769435 10.1021/cb3002705 PMC3477269

[jev212404-bib-0380] Sanada, T. , Hirata, Y. , Naito, Y. , Yamamoto, N. , Kikkawa, Y. , Ishida, Y. , Yamasaki, C. , Tateno, C. , Ochiya, T. , & Kohara, M. (2017). Transmission of HBV DNA mediated by ceramide‐triggered extracellular vesicles. Cellular and Molecular Gastroenterology and Hepatology, 3, 272–283. 28275693 10.1016/j.jcmgh.2016.10.003 PMC5331779

[jev212404-bib-0381] Sandau, U. S. , Duggan, E. , Shi, X. , Smith, S. J. , Huckans, M. , Schutzer, W. E. , Loftis, J. M. , Janowsky, A. , Nolan, J. P. , & Saugstad, J. A. (2020). Methamphetamine use alters human plasma extracellular vesicles and their microRNA cargo: An exploratory study. Journal of Extracellular Vesicles, 10, e12028. 33613872 10.1002/jev2.12028 PMC7890470

[jev212404-bib-0382] Sansone, P. , Savini, C. , Kurelac, I. , Chang, Q. , Amato, L. B. , Strillacci, A. , Stepanova, A. , Iommarini, L. , Mastroleo, C. , Daly, L. , Galkin, A. , Thakur, B. K. , Soplop, N. , Uryu, K. , Hoshino, A. , Norton, L. , Bonafé, M. , Cricca, M. , Gasparre, G. , … Bromberg, J. (2017). Packaging and transfer of mitochondrial DNA via exosomes regulate escape from dormancy in hormonal therapy‐resistant breast cancer. PNAS, 114, E9066–E9075. 29073103 10.1073/pnas.1704862114 PMC5664494

[jev212404-bib-0383] Santoro, J. , Mukhopadhya, A. , Oliver, C. , Brodkorb, A. , Giblin, L. , & O'Driscoll, L. (2023). An investigation of extracellular vesicles in bovine colostrum, first milk and milk over the lactation curve. Food Chemistry, 401, 134029. 36108387 10.1016/j.foodchem.2022.134029

[jev212404-bib-0384] Schioppo, T. , Ubiali, T. , Ingegnoli, F. , Bollati, V. , & Caporali, R. (2021). The role of extracellular vesicles in rheumatoid arthritis: a systematic review. Clinical Rheumatology, 40, 3481–3497. 33544235 10.1007/s10067-021-05614-w PMC8357675

[jev212404-bib-0385] Scott, A. , Sueiro Ballesteros, L. , Bradshaw, M. , Tsuji, C. , Power, A. , Lorriman, J. , Love, J. , Paul, D. , Herman, A. , Emanueli, C. , & Richardson, R. J. (2021). In vivo characterization of endogenous cardiovascular extracellular vesicles in larval and adult zebrafish. Arteriosclerosis, Thrombosis, and Vascular Biology, 41, 2454–2468. 34261327 10.1161/ATVBAHA.121.316539 PMC8384253

[jev212404-bib-0386] Shah, S. S. , Ebberson, J. , Kestenbaum, L. A. , Hodinka, R. L. , & Zorc, J. J. (2011). Age‐specific reference values for cerebrospinal fluid protein concentration in neonates and young infants. Journal of Hospital Medicine, 6, 22–27. 20629018 10.1002/jhm.711 PMC2978786

[jev212404-bib-0387] Sharma, A. , Mariappan, M. , Appathurai, S. , & Hegde, R. S. (2010). In vitro dissection of protein translocation into the mammalian endoplasmic reticulum. Methods in Molecular Biology, 619, 339–363. 20419420 10.1007/978-1-60327-412-8_20 PMC3122127

[jev212404-bib-0388] Sharma, S. , Gillespie, B. M. , Palanisamy, V. , & Gimzewski, J. K. (2011). Quantitative nanostructural and single‐molecule force spectroscopy biomolecular analysis of human‐saliva‐derived exosomes. Langmuir, 27, 14394–14400. 22017459 10.1021/la2038763 PMC3235036

[jev212404-bib-0389] Sharma, S. , LeClaire, M. , & Gimzewski, J. K. (2018). Ascent of atomic force microscopy as a nanoanalytical tool for exosomes and other extracellular vesicles. Nanotechnology, 29, 132001. 29376505 10.1088/1361-6528/aaab06

[jev212404-bib-0390] Sharma, S. , LeClaire, M. , Wohlschlegel, J. , & Gimzewski, J. (2020). Impact of isolation methods on the biophysical heterogeneity of single extracellular vesicles. Scientific Reports, 10, 13327. 32770003 10.1038/s41598-020-70245-1 PMC7414114

[jev212404-bib-0391] Shekari, F. , Alibhai, F. J. , Baharvand, H. , Börger, V. , Bruno, S. , Davies, O. , Giebel, B. , Gimona, M. , Salekdeh, G. H. , Martin‐Jaular, L. , Mathivanan, S. , Nelissen, I. , Hoen, E. N.‐T. , O'Driscoll, L. , Perut, F. , Pluchino, S. , Pocsfalvi, G. , Salomon, C. , Soekmadji, C. , … Nieuwland, R. (2023). Cell culture‐derived extracellular vesicles: Considerations for reporting cell culturing parameters. Journal of Extracellular Biology, 2, e115. 10.1002/jex2.115 PMC11080896 38939735

[jev212404-bib-0392] Shen, S. , Shen, Z. , Wang, C. , Wu, X. , Wang, L. , Ye, L. , Zhang, S. , & Cheng, X. (2023). Effects of lysate/tissue storage at −80°C on subsequently extracted EVs of epithelial ovarian cancer tissue origins. Iscience, 26, 106521. 37123245 10.1016/j.isci.2023.106521 PMC10139970

[jev212404-bib-0393] Shlomovitz, I. , Erlich, Z. , Arad, G. , Edry‐Botzer, L. , Zargarian, S. , Cohen, H. , Manko, T. , Ofir‐Birin, Y. , Cooks, T. , Regev‐Rudzki, N. , & Gerlic, M. (2021). Proteomic analysis of necroptotic extracellular vesicles. Cell Death & Disease, 12, 1059. 34750357 10.1038/s41419-021-04317-z PMC8575773

[jev212404-bib-0394] Silva, A. M. , Lázaro‐Ibáñez, E. , Gunnarsson, A. , Dhande, A. , Daaboul, G. , Peacock, B. , Osteikoetxea, X. , Salmond, N. , Friis, K. P. , Shatnyeva, O. , & Dekker, N. (2021). Quantification of protein cargo loading into engineered extracellular vesicles at single‐vesicle and single‐molecule resolution. Journal of Extracellular Vesicles, 10, e12130. 34377376 10.1002/jev2.12130 PMC8329990

[jev212404-bib-0395] Simonsen, J. B. (2017). What are we looking at? Extracellular vesicles, lipoproteins, or both? Circulation Research, 121, 920–922. 28963190 10.1161/CIRCRESAHA.117.311767

[jev212404-bib-0396] Simonsen, J. B. (2019). Pitfalls associated with lipophilic fluorophore staining of extracellular vesicles for uptake studies. Journal of Extracellular Vesicles, 8, 1582237. 30815239 10.1080/20013078.2019.1582237 PMC6383605

[jev212404-bib-0397] Singh, P. , Szigyártó, I. C. , Ricci, M. , Zsila, F. , Juhász, T. , Mihály, J. , Bősze, S. , Bulyáki, É. , Kardos, J. , Kitka, D. , Varga, Z. , & Beke‐Somfai, T. (2020). Membrane active peptides remove surface adsorbed protein corona from extracellular vesicles of red blood cells. Frontiers in Chemistry, 8, 703. 32850685 10.3389/fchem.2020.00703 PMC7432246

[jev212404-bib-0398] Sitar, S. , Kejžar, A. , Pahovnik, D. , Kogej, K. , Tušek‐Žnidarič, M. , Lenassi, M. , & Žagar, E. (2015). Size characterization and quantification of exosomes by asymmetrical‐flow field‐flow fractionation. Analytical Chemistry, 87, 9225–9233. 26291637 10.1021/acs.analchem.5b01636

[jev212404-bib-0399] Skliar, M. , Chernyshev, V. S. , Belnap, D. M. , Sergey, G. V. , Al‐Hakami, S. M. , Bernard, P. S. , Stijleman, I. J. , & Rachamadugu, R. (2018). Membrane proteins significantly restrict exosome mobility. Biochemical and Biophysical Research Communications, 501, 1055–1059. 29777705 10.1016/j.bbrc.2018.05.107

[jev212404-bib-0400] Skotland, T. , Iversen, T. G. , Llorente, A. , & Sandvig, K. (2022). Biodistribution, pharmacokinetics and excretion studies of intravenously injected nanoparticles and extracellular vesicles: Possibilities and challenges. Advanced Drug Delivery Reviews, 186, 114326. 35588953 10.1016/j.addr.2022.114326

[jev212404-bib-0401] Smith, E. , & Dent, G. (2005). Modern raman spectroscopy: A practical approach. 1–210.

[jev212404-bib-0402] Smith, Z. J. , Lee, C. , Rojalin, T. , Carney, R. P. , Hazari, S. , Knudson, A. , Lam, K. , Saari, H. , Ibanez, E. L. , Viitala, T. , Laaksonen, T. , Yliperttula, M. , & Wachsmann‐Hogiu, S. (2015). Single exosome study reveals subpopulations distributed among cell lines with variability related to membrane content. Journal of Extracellular Vesicles, 4, 28533. 26649679 10.3402/jev.v4.28533 PMC4673914

[jev212404-bib-0403] Sódar, B. W. , Kittel, Á. , Pálóczi, K. , Vukman, K. V. , Osteikoetxea, X. , Szabó‐Taylor, K. , Németh, A. , Sperlágh, B. , Baranyai, T. , Giricz, Z. , Wiener, Z. , Turiák, L. , Drahos, L. , Pállinger, É. , Vékey, K. , Ferdinandy, P. , Falus, A. , & Buzás, E. I. (2016). Low‐density lipoprotein mimics blood plasma‐derived exosomes and microvesicles during isolation and detection. Scientific Reports, 6, 24316. 27087061 10.1038/srep24316 PMC4834552

[jev212404-bib-0404] Sodar, B. W. , Kovacs, A. , Visnovitz, T. , Pallinger, E. , Vekey, K. , Pocsfalvi, G. , Turiak, L. , & Buzas, E. I. (2017). Best practice of identification and proteomic analysis of extracellular vesicles in human health and disease. Expert Review of Proteomics, 14, 1073–1090. 29025360 10.1080/14789450.2017.1392244

[jev212404-bib-0405] Soekmadji, C. , Hill, A. F. , Wauben, M. H. , Buzás, E. I. , Di Vizio, D. , Gardiner, C. , Lötvall, J. , Sahoo, S. , & Witwer, K. W. (2018). Towards mechanisms and standardization in extracellular vesicle and extracellular RNA studies: Results of a worldwide survey. Journal of Extracellular Vesicles, 7, 1535745. 30370018 10.1080/20013078.2018.1535745 PMC6201809

[jev212404-bib-0406] Somiya, M. , & Kuroda, S. (2021a). Real‐time luminescence assay for cytoplasmic cargo delivery of extracellular vesicles. Analytical Chemistry, 93, 5612–5620. 33759512 10.1021/acs.analchem.1c00339

[jev212404-bib-0407] Somiya, M. , & Kuroda, S. (2021b). Reporter gene assay for membrane fusion of extracellular vesicles. Journal of Extracellular Vesicles, 10, e12171. 34807503 10.1002/jev2.12171 PMC8607979

[jev212404-bib-0408] Somiya, M. , Yoshioka, Y. , & Ochiya, T. (2018). Biocompatibility of highly purified bovine milk‐derived extracellular vesicles. Journal of Extracellular Vesicles, 7, 1440132. 29511463 10.1080/20013078.2018.1440132 PMC5827637

[jev212404-bib-0409] Sorkin, R. , Huisjes, R. , Bošković, F. , Vorselen, D. , Pignatelli, S. , Ofir‐Birin, Y. , Freitas Leal, J. K. , Schiller, J. , Mullick, D. , Roos, W. H. , Bosman, G. , Regev‐Rudzki, N. , Schiffelers, R. M. , & Wuite, G. J. L. (2018). Nanomechanics of extracellular vesicles reveals vesiculation pathways. Small, 14, e1801650. 30160371 10.1002/smll.201801650

[jev212404-bib-0410] Stam, J. , Bartel, S. , Bischoff, R. , & Wolters, J. C. (2021). Isolation of extracellular vesicles with combined enrichment methods. Journal of Chromatography. B, Analytical Technologies in the Biomedical and Life Sciences, 1169, 122604. 33713953 10.1016/j.jchromb.2021.122604

[jev212404-bib-0411] Steenbeek, S. C. , Pham, T. V. , de Ligt, J. , Zomer, A. , Knol, J. C. , Piersma, S. R. , Schelfhorst, T. , Huisjes, R. , Schiffelers, R. M. , Cuppen, E. , Jimenez, C. R. , & van Rheenen, J. (2018). Cancer cells copy migratory behavior and exchange signaling networks via extracellular vesicles. Embo Journal, 37, e98357. 29907695 10.15252/embj.201798357 PMC6068466

[jev212404-bib-0412] Stetefeld, J. , McKenna, S. A. , & Patel, T. R. (2016). Dynamic light scattering: a practical guide and applications in biomedical sciences. Biophysical Reviews, 8, 409–427. 28510011 10.1007/s12551-016-0218-6 PMC5425802

[jev212404-bib-0413] Stoner, S. A. , Duggan, E. , Condello, D. , Guerrero, A. , Turk, J. R. , Narayanan, P. K. , & Nolan, J. P. (2016). High sensitivity flow cytometry of membrane vesicles. Cytometry Part A: the journal of the International Society for Analytical Cytology, 89, 196–206. 26484737 10.1002/cyto.a.22787

[jev212404-bib-0414] Sung, B. H. , Ketova, T. , Hoshino, D. , Zijlstra, A. , & Weaver, A. M. (2015). Directional cell movement through tissues is controlled by exosome secretion. Nature Communications, 6, 7164. 10.1038/ncomms8164 PMC4435734 25968605

[jev212404-bib-0415] Sung, B. H. , & Weaver, A. M. (2017). Exosome secretion promotes chemotaxis of cancer cells. Cell Adhesion & Migration, 11, 187–195. 28129015 10.1080/19336918.2016.1273307 PMC5351719

[jev212404-bib-0416] Suwatthanarak, T. , Thiodorus, I. A. , Tanaka, M. , Shimada, T. , Takeshita, D. , Yasui, T. , Baba, Y. , & Okochi, M. (2021). Microfluidic‐based capture and release of cancer‐derived exosomes via peptide‐nanowire hybrid interface. Lab on A Chip, 21, 597–607. 33367429 10.1039/d0lc00899k

[jev212404-bib-0417] Taha, E. A. , Sogawa, C. , Okusha, Y. , Kawai, H. , Oo, M. W. , Elseoudi, A. , Lu, Y. , Nagatsuka, H. , Kubota, S. , Satoh, A. , Okamoto, K. , & Eguchi, T. (2020). Knockout of MMP3 weakens solid tumor organoids and cancer extracellular vesicles. Cancers (Basel), 12, 1260. 32429403 10.3390/cancers12051260 PMC7281240

[jev212404-bib-0418] Taher, S. , Borja, Y. , Cabanela, L. , Costers, V. J. , Carson‐Marino, M. , Bailes, J. C. , Dhar, B. , Beckworth, M. T. , Rabaglino, M. B. , Post Uiterweer, E. D. , & Conrad, K. P. (2019). Cholecystokinin, gastrin, cholecystokinin/gastrin receptors, and bitter taste receptor TAS2R14: Trophoblast expression and signaling. American Journal of Physiology. Regulatory, Integrative and Comparative Physiology, 316, R628–R639. 30892908 10.1152/ajpregu.00153.2018 PMC6589605

[jev212404-bib-0419] Takov, K. , Yellon, D. M. , & Davidson, S. M. (2017). Confounding factors in vesicle uptake studies using fluorescent lipophilic membrane dyes. Journal of Extracellular Vesicles, 6, 1388731. 29184625 10.1080/20013078.2017.1388731 PMC5699187

[jev212404-bib-0420] Tassetto, M. , Kunitomi, M. , & Andino, R. (2017). Circulating immune cells mediate a systemic RNAi‐based adaptive antiviral response in drosophila. Cell, 169, 314–325.e13. 28388413 10.1016/j.cell.2017.03.033 PMC5730277

[jev212404-bib-0421] Taylor, C. F. , Paton, N. W. , Lilley, K. S. , Binz, P. A. , Julian R. K., Jr , Jones, A. R. , Zhu, W. , Apweiler, R. , Aebersold, R. , Deutsch, E. W. , Dunn, M. J. , Heck, A. J. , Leitner, A. , Macht, M. , Mann, M. , Martens, L. , Neubert, T. A. , Patterson, S. D. , Ping, P. , … Hermjakob, H. (2007). The minimum information about a proteomics experiment (MIAPE). Nature Biotechnology, 25, 887–893. 10.1038/nbt1329 17687369

[jev212404-bib-0422] Ter‐Ovanesyan, D. , Gilboa, T. , Budnik, B. , Nikitina, A. , Whiteman, S. , Lazarovits, R. , Trieu, W. , Kalish, D. , Church, G. M. , & Walt, D. R. (2023). Improved isolation of extracellular vesicles by removal of both free proteins and lipoproteins. Elife, 12, e86394. 37252755 10.7554/eLife.86394 PMC10229111

[jev212404-bib-0423] Ter‐Ovanesyan, D. , Norman, M. , Lazarovits, R. , Trieu, W. , Lee, J. H. , Church, G. M. , & Walt, D. R. (2021). Framework for rapid comparison of extracellular vesicle isolation methods. Elife, 10, e70725. 34783650 10.7554/eLife.70725 PMC8651285

[jev212404-bib-0424] Teunissen, C. E. , Petzold, A. , Bennett, J. L. , Berven, F. S. , Brundin, L. , Comabella, M. , Franciotta, D. , Frederiksen, J. L. , Fleming, J. O. , Furlan, R. , Hintzen, R. Q. , Hughes, S. G. , Johnson, M. H. , Krasulova, E. , Kuhle, J. , Magnone, M. C. , Rajda, C. , Rejdak, K. , Schmidt, H. K. , … Deisenhammer, F. (2009). A consensus protocol for the standardization of cerebrospinal fluid collection and biobanking. Neurology, 73, 1914–1922. 19949037 10.1212/WNL.0b013e3181c47cc2 PMC2839806

[jev212404-bib-0425] Thakur, B. K. , Zhang, H. , Becker, A. , Matei, I. , Huang, Y. , Costa‐Silva, B. , Zheng, Y. , Hoshino, A. , Brazier, H. , Xiang, J. , Williams, C. , Rodriguez‐Barrueco, R. , Silva, J. M. , Zhang, W. , Hearn, S. , Elemento, O. , Paknejad, N. , Manova‐Todorova, K. , Welte, K. , … Lyden, D. (2014). Double‐stranded DNA in exosomes: A novel biomarker in cancer detection. Cell Research, 24, 766–769. 24710597 10.1038/cr.2014.44 PMC4042169

[jev212404-bib-0426] Théry, C. , Amigorena, S. , Raposo, G. , & Clayton, A. (2006). Isolation and characterization of exosomes from cell culture supernatants and biological fluids. Current Protocols in Cell Biology, Chapter 3, Unit 3.22. 10.1002/0471143030.cb0322s30 18228490

[jev212404-bib-0427] Théry, C. , Witwer, K. W. , Aikawa, E. , Alcaraz, M. J. , Anderson, J. D. , Andriantsitohaina, R. , Antoniou, A. , Arab, T. , Archer, F. , Atkin‐Smith, G. K. , Ayre, D. C. , Bach, J. M. , Bachurski, D. , Baharvand, H. , Balaj, L. , Baldacchino, S. , Bauer, N. N. , Baxter, A. A. , Bebawy, M. , … Zuba‐Surma, E. K. (2018). Minimal information for studies of extracellular vesicles 2018 (MISEV2018): A position statement of the International Society for Extracellular Vesicles and update of the MISEV2014 guidelines. Journal of Extracellular Vesicles, 7(1), 1535750. 10.1080/20013078.2018.1535750 30637094 PMC6322352

[jev212404-bib-0428] Thomas, R. E. , Vincow, E. S. , Merrihew, G. E. , MacCoss, M. J. , Davis, M. Y. , & Pallanck, L. J. (2018). Glucocerebrosidase deficiency promotes protein aggregation through dysregulation of extracellular vesicles. Plos Genetics, 14, e1007694. 30256786 10.1371/journal.pgen.1007694 PMC6175534

[jev212404-bib-0429] Tian, T. , Wang, Y. , Wang, H. , Zhu, Z. , & Xiao, Z. (2010). Visualizing of the cellular uptake and intracellular trafficking of exosomes by live‐cell microscopy. Journal of Cellular Biochemistry, 111, 488–496. 20533300 10.1002/jcb.22733

[jev212404-bib-0430] Tian, Y. , Gong, M. , Hu, Y. , Liu, H. , Zhang, W. , Zhang, M. , Hu, X. , Aubert, D. , Zhu, S. , Wu, L. , & Yan, X. (2020). Quality and efficiency assessment of six extracellular vesicle isolation methods by nano‐flow cytometry. Journal of Extracellular Vesicles, 9, 1697028. 31839906 10.1080/20013078.2019.1697028 PMC6896440

[jev212404-bib-0431] Tian, Y. , Ma, L. , Gong, M. , Su, G. , Zhu, S. , Zhang, W. , Wang, S. , Li, Z. , Chen, C. , Li, L. , Wu, L. , & Yan, X. (2018). Protein profiling and sizing of extracellular vesicles from colorectal cancer patients via flow cytometry. ACS Nano, 12, 671–680. 29300458 10.1021/acsnano.7b07782

[jev212404-bib-0432] Toda, H. , Diaz‐Varela, M. , Segui‐Barber, J. , Roobsoong, W. , Baro, B. , Garcia‐Silva, S. , Galiano, A. , Gualdron‐Lopez, M. , Almeida, A. C. G. , Brito, M. A. M. , de Melo, G. C. , Aparici‐Herraiz, I. , Castro‐Cavadia, C. , Monteiro, W. M. , Borras, E. , Sabido, E. , Almeida, I. C. , Chojnacki, J. , Martinez‐Picado, J. , … del Portillo, H. A. (2020). Plasma‐derived extracellular vesicles from Plasmodium vivax patients signal spleen fibroblasts via NF‐kB facilitating parasite cytoadherence. Nature Communications, 11, 2761. 10.1038/s41467-020-16337-y PMC7265481 32487994

[jev212404-bib-0433] Tóth, E. Á. , Turiák, L. , Visnovitz, T. , Cserép, C. , Mázló, A. , Sódar, B. W. , Försönits, A. I. , Petővári, G. , Sebestyén, A. , Komlósi, Z. , Drahos, L. , Kittel, Á. , Nagy, G. , Bácsi, A. , Dénes, Á. , Gho, Y. S. , Szabó‐Taylor, K. É. , & Buzás, E. I. (2021). Formation of a protein corona on the surface of extracellular vesicles in blood plasma. Journal of Extracellular Vesicles, 10, e12140. 34520123 10.1002/jev2.12140 PMC8439280

[jev212404-bib-0434] Toyofuku, M. , Schild, S. , Kaparakis‐Liaskos, M. , & Eberl, L. (2023). Composition and functions of bacterial membrane vesicles. Nature Reviews Microbiology, 21, 415–430. 36932221 10.1038/s41579-023-00875-5

[jev212404-bib-0435] Trajkovic, K. , Hsu, C. , Chiantia, S. , Rajendran, L. , Wenzel, D. , Wieland, F. , Schwille, P. , Brügger, B. , & Simons, M. (2008). Ceramide triggers budding of exosome vesicles into multivesicular endosomes. Science, 319, 1244–1247. 18309083 10.1126/science.1153124

[jev212404-bib-0436] Trenkenschuh, E. , Richter, M. , Heinrich, E. , Koch, M. , Fuhrmann, G. , & Friess, W. (2022). Enhancing the stabilization potential of lyophilization for extracellular vesicles. Advanced Healthcare Materials, 11, e2100538. 34310074 10.1002/adhm.202100538 PMC11468620

[jev212404-bib-0437] Tsai, Y. W. , Sung, H. H. , Li, J. C. , Yeh, C. Y. , Chen, P. Y. , Cheng, Y. J. , Chen, C. H. , Tsai, Y. C. , & Chien, C. T. (2019). Glia‐derived exosomal miR‐274 targets Sprouty in trachea and synaptic boutons to modulate growth and responses to hypoxia. PNAS, 116, 24651–24661. 31666321 10.1073/pnas.1902537116 PMC6900535

[jev212404-bib-0438] Tulkens, J. , Vergauwen, G. , Van Deun, J. , Geeurickx, E. , Dhondt, B. , Lippens, L. , De Scheerder, M. A. , Miinalainen, I. , Rappu, P. , De Geest, B. G. , Vandecasteele, K. , Laukens, D. , Vandekerckhove, L. , Denys, H. , Vandesompele, J. , De Wever, O. , & Hendrix, A. (2020). Increased levels of systemic LPS‐positive bacterial extracellular vesicles in patients with intestinal barrier dysfunction. Gut, 69, 191–193. 30518529 10.1136/gutjnl-2018-317726 PMC6943244

[jev212404-bib-0439] Turner, L. , Bitto, N. J. , Steer, D. L. , Lo, C. , D'Costa, K. , Ramm, G. , Shambrook, M. , Hill, A. F. , Ferrero, R. L. , & Kaparakis‐Liaskos, M. (2018). *Helicobacter pylori* outer membrane vesicle size determines their mechanisms of host cell entry and protein content. Frontiers in Immunology, 9, 1466. 30013553 10.3389/fimmu.2018.01466 PMC6036113

[jev212404-bib-0440] Vagner, T. , Spinelli, C. , Minciacchi, V. R. , Balaj, L. , Zandian, M. , Conley, A. , Zijlstra, A. , Freeman, M. R. , Demichelis, F. , De, S. , Posadas, E. M. , Tanaka, H. , & Di Vizio, D. (2018). Large extracellular vesicles carry most of the tumour DNA circulating in prostate cancer patient plasma. Journal of Extracellular Vesicles, 7, 1505403. 30108686 10.1080/20013078.2018.1505403 PMC6084494

[jev212404-bib-0441] Valcz, G. , Buzas, E. I. , Kittel, A. , Krenacs, T. , Visnovitz, T. , Spisak, S. , Torok, G. , Homolya, L. , Zsigrai, S. , Kiszler, G. , Antalffy, G. , Paloczi, K. , Szallasi, Z. , Szabo, V. , Sebestyen, A. , Solymosi, N. , Kalmar, A. , Dede, K. , Lorincz, P. , … Molnar, B. (2019). En bloc release of MVB‐like small extracellular vesicle clusters by colorectal carcinoma cells. Journal of Extracellular Vesicles, 8(1), 1596668. 31007874 10.1080/20013078.2019.1596668 PMC6461071

[jev212404-bib-0442] Valkov, N. , Das, A. , Tucker, N. R. , Li, G. , Salvador, A. M. , Chaffin, M. D. , Pereira de Oliveira Junior, G. , Kur, I. , Gokulnath, P. , Ziegler, O. , Yeri, A. , Lu, S. , Khamesra, A. , Xiao, C. , Rodosthenous, R. , Srinivasan, S. , Toxavidis, V. , Tigges, J. , Laurent, L. C. , … Das, S. (2021). SnRNA sequencing defines signaling by RBC‐derived extracellular vesicles in the murine heart. Life Science Alliance, 4, e202101048. 34663679 10.26508/lsa.202101048 PMC8548207

[jev212404-bib-0443] van der Pol, E. , Coumans, F. A. , Grootemaat, A. E. , Gardiner, C. , Sargent, I. L. , Harrison, P. , Sturk, A. , van Leeuwen, T. G. , & Nieuwland, R. (2014). Particle size distribution of exosomes and microvesicles determined by transmission electron microscopy, flow cytometry, nanoparticle tracking analysis, and resistive pulse sensing. Journal of Thrombosis and Haemostasis, 12, 1182–1192. 24818656 10.1111/jth.12602

[jev212404-bib-0444] van der Pol, E. , Coumans, F. A. , Sturk, A. , Nieuwland, R. , & van Leeuwen, T. G. (2014). Refractive index determination of nanoparticles in suspension using nanoparticle tracking analysis. Nano Letters, 14, 6195–6201. 25256919 10.1021/nl503371p

[jev212404-bib-0445] van der Pol, E. , de Rond, L. , Coumans, F. A. W. , Gool, E. L. , Boing, A. N. , Sturk, A. , Nieuwland, R. , & van Leeuwen, T. G. (2018). Absolute sizing and label‐free identification of extracellular vesicles by flow cytometry. Nanomedicine, 14, 801–810. 29307842 10.1016/j.nano.2017.12.012

[jev212404-bib-0446] van der Pol, E. , Sturk, A. , van Leeuwen, T. , Nieuwland, R. , Coumans, F. , & Isth‐Ssc‐Vb Working group . (2018). Standardization of extracellular vesicle measurements by flow cytometry through vesicle diameter approximation. Journal of Thrombosis and Haemostasis, 16, 1236–1245. 29575716 10.1111/jth.14009

[jev212404-bib-0447] van der Pol, E. , Welsh, J. A. , & Nieuwland, R. (2022). Minimum information to report about a flow cytometry experiment on extracellular vesicles: Communication from the ISTH SSC subcommittee on vascular biology. Journal of Thrombosis and Haemostasis, 20, 245–251. 34637195 10.1111/jth.15540 PMC8729195

[jev212404-bib-0448] van Deun, J. , Jo, A. , Li, H. , Lin, H. Y. , Weissleder, R. , Im, H. , & Lee, H. (2020). Integrated dual‐mode chromatography to enrich extracellular vesicles from plasma. Advanced Biology, 4, e1900310. 10.1002/adbi.201900310 PMC7606548 32351054

[jev212404-bib-0449] van Royen, M. E. , Soekmadji, C. , Grange, C. , Webber, J. P. , Tertel, T. , Droste, M. , Buescher, A. , Giebel, B. , Jenster, G. W. , Llorente, A. , Blijdorp, C. J. , Burger, D. , Erdbrügger, U. , & Martens‐Uzunova, E. S. (2023). The quick reference card “Storage of urinary EVs” – A practical guideline tool for research and clinical laboratories. Journal of Extracellular Vesicles, 12, e12286. 36916183 10.1002/jev2.12286 PMC10011888

[jev212404-bib-0450] Vella, L. J. , Scicluna, B. J. , Cheng, L. , Bawden, E. G. , Masters, C. L. , Ang, C. S. , Willamson, N. , McLean, C. , Barnham, K. J. , & Hill, A. F. (2017). A rigorous method to enrich for exosomes from brain tissue. Journal of Extracellular Vesicles, 6, 1348885. 28804598 10.1080/20013078.2017.1348885 PMC5533148

[jev212404-bib-0451] Vergauwen, G. , Dhondt, B. , Van Deun, J. , De Smedt, E. , Berx, G. , Timmerman, E. , Gevaert, K. , Miinalainen, I. , Cocquyt, V. , Braems, G. , Van den Broecke, R. , Denys, H. , De Wever, O. , & Hendrix, A. (2017). Confounding factors of ultrafiltration and protein analysis in extracellular vesicle research. Scientific Reports, 7, 2704. 28577337 10.1038/s41598-017-02599-y PMC5457435

[jev212404-bib-0452] Vergauwen, G. , Tulkens, J. , Pinheiro, C. , Avila Cobos, F. , Dedeyne, S. , De Scheerder, M. A. , Vandekerckhove, L. , Impens, F. , Miinalainen, I. , Braems, G. , Gevaert, K. , Mestdagh, P. , Vandesompele, J. , Denys, H. , De Wever, O. , & Hendrix, A. (2021). Robust sequential biophysical fractionation of blood plasma to study variations in the biomolecular landscape of systemically circulating extracellular vesicles across clinical conditions. Journal of Extracellular Vesicles, 10, e12122. 34429857 10.1002/jev2.12122 PMC8363909

[jev212404-bib-0453] Verweij, F. J. , Balaj, L. , Boulanger, C. M. , Carter, D. R. F. , Compeer, E. B. , D'Angelo, G. , El Andaloussi, S. , Goetz, J. G. , Gross, J. C. , Hyenne, V. , Krämer‐Albers, E. M. , Lai, C. P. , Loyer, X. , Marki, A. , Momma, S. , Nolte‐’t Hoen, E. N. M. , Pegtel, D. M. , Peinado, H. , Raposo, G. , … van Niel, G. (2021). The power of imaging to understand extracellular vesicle biology in vivo. Nature Methods, 18(9), 1013–1026. 34446922 10.1038/s41592-021-01206-3 PMC8796660

[jev212404-bib-0454] Verweij, F. J. , Revenu, C. , Arras, G. , Dingli, F. , Loew, D. , Pegtel, D. M. , Follain, G. , Allio, G. , Goetz, J. G. , Zimmermann, P. , Herbomel, P. , Del Bene, F. , Raposo, G. , & van Niel, G. (2019). Live tracking of inter‐organ communication by endogenous exosomes in vivo. Developmental Cell, 48, 573–589.e4. 30745143 10.1016/j.devcel.2019.01.004

[jev212404-bib-0455] Verwilt, J. , Trypsteen, W. , Van Paemel, R. , De Preter, K. , Giraldez, M. D. , Mestdagh, P. , & Vandesompele, J. (2020). When DNA gets in the way: A cautionary note for DNA contamination in extracellular RNA‐seq studies. PNAS, 117, 18934–18936. 32788394 10.1073/pnas.2001675117 PMC7431080

[jev212404-bib-0456] Vestad, B. , Llorente, A. , Neurauter, A. , Phuyal, S. , Kierulf, B. , Kierulf, P. , Skotland, T. , Sandvig, K. , Haug, K. B. F. , & Ovstebo, R. (2017). Size and concentration analyses of extracellular vesicles by nanoparticle tracking analysis: A variation study. Journal of Extracellular Vesicles, 6, 1344087. 28804597 10.1080/20013078.2017.1344087 PMC5533132

[jev212404-bib-0457] Visnovitz, T. , Osteikoetxea, X. , Sodar, B. W. , Mihaly, J. , Lorincz, P. , Vukman, K. V. , Toth, E. A. , Koncz, A. , Szekacs, I. , Horvath, R. , Varga, Z. , & Buzas, E. I. (2019). An improved 96 well plate format lipid quantification assay for standardisation of experiments with extracellular vesicles. Journal of Extracellular Vesicles, 8, 1565263. 30728922 10.1080/20013078.2019.1565263 PMC6352952

[jev212404-bib-0458] Vorselen, D. , MacKintosh, F. C. , Roos, W. H. , & Wuite, G. J. (2017). Competition between bending and internal pressure governs the mechanics of fluid nanovesicles. ACS Nano, 11, 2628–2636. 28273422 10.1021/acsnano.6b07302 PMC5371924

[jev212404-bib-0459] Vorselen, D. , van Dommelen, S. M. , Sorkin, R. , Piontek, M. C. , Schiller, J. , Döpp, S. T. , Kooijmans, S. A. A. , van Oirschot, B. A. , Versluijs, B. A. , Bierings, M. B. , van Wijk, R. , Schiffelers, R. M. , Wuite, G. J. L. , & Roos, W. H. (2018). The fluid membrane determines mechanics of erythrocyte extracellular vesicles and is softened in hereditary spherocytosis. Nature Communications, 9, 4960. 10.1038/s41467-018-07445-x PMC6251882 30470753

[jev212404-bib-0460] Walker, J. G. (2012). Improved nano‐particle tracking analysis. Measurement Science and Technology, 23, 065605.

[jev212404-bib-0461] Walsh, R. B. , Dresselhaus, E. C. , Becalska, A. N. , Zunitch, M. J. , Blanchette, C. R. , Scalera, A. L. , Lemos, T. , Lee, S. M. , Apiki, J. , Wang, S. , Isaac, B. , Yeh, A. , Koles, K. , & Rodal, A. A. (2021). Opposing functions for retromer and Rab11 in extracellular vesicle traffic at presynaptic terminals. Journal of Cell Biology, 220, e202012034. 34019080 10.1083/jcb.202012034 PMC8144913

[jev212404-bib-0462] Wang, C. , Ding, Q. , Plant, P. , Basheer, M. , Yang, C. , Tawedrous, E. , Krizova, A. , Boulos, C. , Farag, M. , Cheng, Y. , & Yousef, G. M. (2019). Droplet digital PCR improves urinary exosomal miRNA detection compared to real‐time PCR. Clinical Biochemistry, 67, 54–59. 30905583 10.1016/j.clinbiochem.2019.03.008

[jev212404-bib-0463] Wang, J. , Kaletsky, R. , Silva, M. , Williams, A. , Haas, L. A. , Androwski, R. J. , Landis, J. N. , Patrick, C. , Rashid, A. , Santiago‐Martinez, D. , Gravato‐Nobre, M. , Hodgkin, J. , Hall, D. H. , Murphy, C. T. , & Barr, M. M. (2015). Cell‐specific transcriptional profiling of ciliated sensory neurons reveals regulators of behavior and extracellular vesicle biogenesis. Current Biology, 25, 3232–3238. 26687621 10.1016/j.cub.2015.10.057 PMC4698341

[jev212404-bib-0464] Wang, J. , Silva, M. , Haas, L. A. , Morsci, N. S. , Nguyen, K. C. , Hall, D. H. , & Barr, M. M. (2014). *C. elegans* ciliated sensory neurons release extracellular vesicles that function in animal communication. Current Biology, 24, 519–525. 24530063 10.1016/j.cub.2014.01.002 PMC4659354

[jev212404-bib-0465] Wang, Q. , & Lu, Q. (2017). Plasma membrane‐derived extracellular microvesicles mediate non‐canonical intercellular NOTCH signaling. Nature Communications, 8, 709. 10.1038/s41467-017-00767-2 PMC5617834 28955033

[jev212404-bib-0466] Wang, X. , Shen, H. , Zhangyuan, G. , Huang, R. , Zhang, W. , He, Q. , Jin, K. , Zhuo, H. , Zhang, Z. , Wang, J. , Sun, B. , & Lu, X. (2018). 14‐3‐3zeta delivered by hepatocellular carcinoma‐derived exosomes impaired anti‐tumor function of tumor‐infiltrating T lymphocytes. Cell Death & Disease, 9, 159. 29415983 10.1038/s41419-017-0180-7 PMC5833352

[jev212404-bib-0467] Wang, Z. T. , Li, K. Y. , Tan, C. C. , Xu, W. , Shen, X. N. , Cao, X. P. , Wang, P. , Bi, Y. L. , Dong, Q. , Tan, L. , & Yu, J. T. (2021). Associations of alcohol consumption with cerebrospinal fluid biomarkers of Alzheimer's disease pathology in cognitively intact older adults: The CABLE Study. Journal of Alzheimer's Disease, 82, 1045–1054. 10.3233/JAD-210140 34151793

[jev212404-bib-0468] Wehman, A. M. , Poggioli, C. , Schweinsberg, P. , Grant, B. D. , & Nance, J. (2011). The P4‐ATPase TAT‐5 inhibits the budding of extracellular vesicles in *C. elegans* embryos. Current Biology, 21, 1951–1959. 22100064 10.1016/j.cub.2011.10.040 PMC3237752

[jev212404-bib-0469] Welsh, J. A. , Killingsworth, B. , Kepley, J. , Traynor, T. , Cook, S. , Savage, J. , Marte, J. , Lee, M. , Maeng, H. M. , Pleet, M. L. , Magana, S. , Gorgens, A. , Maire, C. L. , Lamszus, K. , Ricklefs, F. L. , Merino, M. J. , Linehan, W. M. , Greten, T. , Cooks, T. , … Jones, J. C. (2022). MPAPASS software enables stitched multiplex, multidimensional EV repertoire analysis and a standard framework for reporting bead‐based assays. Cell Reports Methods, 2, 100136. 35474866 10.1016/j.crmeth.2021.100136 PMC9017130

[jev212404-bib-0470] Welsh, J. A. , Arkesteijn, G. J. A. , Bremer, M. , Cimorelli, M. , Dignat‐George, F. , Giebel, B. , Görgens, A. , Hendrix, A. , Kuiper, M. , Lacroix, R. , Lannigan, J. , van Leeuwen, T. G. , Lozano‐Andrés, E. , Rao, S. , Robert, S. , de Rond, L. , Tang, V. A. , Tertel, T. , Yan, X. , … van der Pol, E. (2023). A compendium of single extracellular vesicle flow cytometry. Journal of Extracellular Vesicles, 12, e12299. 36759917 10.1002/jev2.12299 PMC9911638

[jev212404-bib-0471] Welsh, J. A. , Horak, P. , Wilkinson, J. S. , Ford, V. J. , Jones, J. C. , Smith, D. , Holloway, J. A. , & Englyst, N. A. (2020). FCMPASS software aids extracellular vesicle light scatter standardization. Cytometry Part A: The Journal of the International Society for Analytical Cytology, 97, 569–581. 31250561 10.1002/cyto.a.23782 PMC7061335

[jev212404-bib-0472] Welsh, J. A. , Jones, J. C. , & Tang, V. A. (2020). Fluorescence and light scatter calibration allow comparisons of small particle data in standard units across different flow cytometry platforms and detector settings. Cytometry Part A: The Journal of the International Society for Analytical Cytology, 97, 592–601. 32476280 10.1002/cyto.a.24029 PMC8482305

[jev212404-bib-0473] Welsh, J. A. , Killingsworth, B. , Kepley, J. , Traynor, T. , McKinnon, K. , Savage, J. , Appel, D. , Aldape, K. , Camphausen, K. , Berzofsky, J. A. , Ivanov, A. R. , Ghiran, I. H. , & Jones, J. C. (2021). A simple, high‐throughput method of protein and label removal from extracellular vesicle samples. Nanoscale, 13, 3737–3745. 33544111 10.1039/d0nr07830a PMC7941347

[jev212404-bib-0474] Welsh, J. A. , Tang, V. A. , van der Pol, E. , & Görgens, A. (2021). MIFlowCyt‐EV: The next chapter in the reporting and reliability of single extracellular vesicle flow cytometry experiments. Cytometry Part A: The Journal of the International Society for Analytical Cytology, 99, 365–368. 33200505 10.1002/cyto.a.24268

[jev212404-bib-0475] Welsh, J. A. , Van Der Pol, E. , Arkesteijn, G. J. A. , Bremer, M. , Brisson, A. , Coumans, F. , Dignat‐George, F. , Duggan, E. , Ghiran, I. , Giebel, B. , Gorgens, A. , Hendrix, A. , Lacroix, R. , Lannigan, J. , Libregts, S. , Lozano‐Andres, E. , Morales‐Kastresana, A. , Robert, S. , de Rond, L. , … Jones, J. C. (2020). MIFlowCyt‐EV: A framework for standardized reporting of extracellular vesicle flow cytometry experiments. Journal of Extracellular Vesicles, 9, 1713526. 32128070 10.1080/20013078.2020.1713526 PMC7034442

[jev212404-bib-0476] Welsh, J. A. , van der Pol, E. , Bettin, B. A. , Carter, D. R. F. , Hendrix, A. , Lenassi, M. , Langlois, M. A. , Llorente, A. , van de Nes, A. S. , Nieuwland, R. , Tang, V. , Wang, L. , Witwer, K. W. , & Jones, J. C. (2020). Towards defining reference materials for measuring extracellular vesicle refractive index, epitope abundance, size and concentration. Journal of Extracellular Vesicles, 9, 1816641. 33062218 10.1080/20013078.2020.1816641 PMC7534292

[jev212404-bib-0477] Wen, S. W. , Sceneay, J. , Lima, L. G. , Wong, C. S. , Becker, M. , Krumeich, S. , Lobb, R. J. , Castillo, V. , Wong, K. N. , Ellis, S. , Parker, B. S. , & Möller, A. (2016). The biodistribution and immune suppressive effects of breast cancer‐derived exosomes. Cancer Research, 76, 6816–6827. 27760789 10.1158/0008-5472.CAN-16-0868

[jev212404-bib-0478] Whitehead, B. , Wu, L. , Hvam, M. L. , Aslan, H. , Dong, M. , Dyrskjøt, L. , Ostenfeld, M. S. , Moghimi, S. M. , & Howard, K. A. (2015). Tumour exosomes display differential mechanical and complement activation properties dependent on malignant state: Implications in endothelial leakiness. Journal of Extracellular Vesicles, 4, 29685. 26714455 10.3402/jev.v4.29685 PMC4695623

[jev212404-bib-0479] Wiklander, O. P. B. , Bostancioglu, R. B. , Welsh, J. A. , Zickler, A. M. , Murke, F. , Corso, G. , Felldin, U. , Hagey, D. W. , Evertsson, B. , Liang, X. M. , Gustafsson, M. O. , Mohammad, D. K. , Wiek, C. , Hanenberg, H. , Bremer, M. , Gupta, D. , Bjornstedt, M. , Giebel, B. , Nordin, J. Z. , … Gorgens, A. (2018). Systematic methodological evaluation of a multiplex bead‐based flow cytometry assay for detection of extracellular vesicle surface signatures. Frontiers in Immunology, 9, 1326. 29951064 10.3389/fimmu.2018.01326 PMC6008374

[jev212404-bib-0480] Wiklander, O. P. , Nordin, J. Z. , O'Loughlin, A. , Gustafsson, Y. , Corso, G. , Mäger, I. , Vader, P. , Lee, Y. , Sork, H. , Seow, Y. , Heldring, N. , Alvarez‐Erviti, L. , Smith, C. I. , Blanc, K. Le , Macchiarini, P. , Jungebluth, P. , Wood, M. J. , & Andaloussi, S. E. (2015). Extracellular vesicle in vivo biodistribution is determined by cell source, route of administration and targeting. Journal of Extracellular Vesicles, 4, 26316. 25899407 10.3402/jev.v4.26316 PMC4405624

[jev212404-bib-0481] Willy, N. M. , Colombo, F. , Huber, S. , Smith, A. C. , Norton, E. G. , Kural, C. , & Cocucci, E. (2021). CALM supports clathrin‐coated vesicle completion upon membrane tension increase. PNAS, 118, e2010438118. 34155137 10.1073/pnas.2010438118 PMC8237669

[jev212404-bib-0482] Witwer, K. W. , Buzas, E. I. , Bemis, L. T. , Bora, A. , Lasser, C. , Lotvall, J. , Nolte‐’t Hoen, E. N. , Piper, M. G. , Sivaraman, S. , Skog, J. , Thery, C. , Wauben, M. H. , & Hochberg, F. (2013). Standardization of sample collection, isolation and analysis methods in extracellular vesicle research. Journal of Extracellular Vesicles, 2, 20360. 10.3402/jev.v2i0.20360 PMC3760646 24009894

[jev212404-bib-0483] Witwer, K. W. , Goberdhan, D. C. , O'Driscoll, L. , Théry, C. , Welsh, J. A. , Blenkiron, C. , Buzás, E. I. , di Vizio, D. , Erdbrügger, U. , Falcón‐Pérez, J. M. , Fu, Q. L. , Hill, A. F. , Lenassi, M. , Lötvall, J. , Nieuwland, R. , Ochiya, T. , Rome, S. , Sahoo, S. , & Zheng, L. (2021). Updating MISEV: Evolving the minimal requirements for studies of extracellular vesicles. Journal of Extracellular Vesicles, 10, e12182. 34953156 10.1002/jev2.12182 PMC8710080

[jev212404-bib-0484] Wolf, M. , Poupardin, R. W. , Ebner‐Peking, P. , Andrade, A. C. , Blöchl, C. , Obermayer, A. , Gomes, F. G. , Vari, B. , Maeding, N. , Eminger, E. , Binder, H. M. , Raninger, A. M. , Hochmann, S. , Brachtl, G. , Spittler, A. , Heuser, T. , Ofir, R. , Huber, C. G. , Aberman, Z. , … Strunk, D. (2022). A functional corona around extracellular vesicles enhances angiogenesis, skin regeneration and immunomodulation. Journal of Extracellular Vesicles, 11, e12207. 35398993 10.1002/jev2.12207 PMC8994701

[jev212404-bib-0485] Wombacher, R. , Heidbreder, M. , van de Linde, S. , Sheetz, M. P. , Heilemann, M. , Cornish, V. W. , & Sauer, M. (2010). Live‐cell super‐resolution imaging with trimethoprim conjugates. Nature Methods, 7, 717–719. 20693998 10.1038/nmeth.1489

[jev212404-bib-0486] Wong, M. , Schlaggar, B. L. , Buller, R. S. , Storch, G. A. , & Landt, M. (2000). Cerebrospinal fluid protein concentration in pediatric patients: Defining clinically relevant reference values. Archives of Pediatrics & Adolescent Medicine, 154, 827–831. 10922281 10.1001/archpedi.154.8.827

[jev212404-bib-0487] Wong, V. (2007). Medication use as a confounding factor in the use of the cerebrospinal fluid tau/beta‐amyloid42 ratio. Archives of Neurology, 64, 1357–1359. 17846282 10.1001/archneur.64.9.1357-a

[jev212404-bib-0488] Wood, C. R. , Huang, K. , Diener, D. R. , & Rosenbaum, J. L. (2013). The cilium secretes bioactive ectosomes. Current Biology, 23, 906–911. 23623554 10.1016/j.cub.2013.04.019 PMC3850760

[jev212404-bib-0489] Woud, W. W. , Hesselink, D. A. , Hoogduijn, M. J. , Baan, C. C. , & Boer, K. (2022). Direct detection of circulating donor‐derived extracellular vesicles in kidney transplant recipients. Scientific Reports, 12, 21973. 36539446 10.1038/s41598-022-26580-6 PMC9768203

[jev212404-bib-0490] Wu, Y. , Deng, W. , & Klinke, D. J. 2nd (2015). Exosomes: Improved methods to characterize their morphology, RNA content, and surface protein biomarkers. Analyst, 140, 6631–6642. 26332016 10.1039/c5an00688k PMC4986832

[jev212404-bib-0491] Xiang, H. , Jin, S. , Tan, F. , Xu, Y. , Lu, Y. , & Wu, T. (2021). Physiological functions and therapeutic applications of neutral sphingomyelinase and acid sphingomyelinase. Biomedicine & Pharmacotherapy, 139, 111610. 33957567 10.1016/j.biopha.2021.111610

[jev212404-bib-0492] Xu, F. , Laguna, L. , & Sarkar, A. (2019). Aging‐related changes in quantity and quality of saliva: Where do we stand in our understanding? Journal of Texture Studies, 50, 27–35. 30091142 10.1111/jtxs.12356

[jev212404-bib-0493] Yang, C. , Chalasani, G. , Ng, Y. H. , & Robbins, P. D. (2012). Exosomes released from Mycoplasma infected tumor cells activate inhibitory B cells. PLoS ONE, 7, e36138. 22558358 10.1371/journal.pone.0036138 PMC3338602

[jev212404-bib-0494] Yang, K. , Jia, M. , Cheddah, S. , Zhang, Z. , Wang, W. , Li, X. , Wang, Y. , & Yan, C. (2022). Peptide ligand‐SiO(2) microspheres with specific affinity for phosphatidylserine as a new strategy to isolate exosomes and application in proteomics to differentiate hepatic cancer. Bioactive Materials, 15, 343–354. 35356814 10.1016/j.bioactmat.2021.12.017 PMC8935132

[jev212404-bib-0495] Ye, S. , Li, W. , Wang, H. , Zhu, L. , Wang, C. , & Yang, Y. (2021). Quantitative nanomechanical analysis of small extracellular vesicles for tumor malignancy indication. Advanced Science, 8, e2100825. 34338437 10.1002/advs.202100825 PMC8456224

[jev212404-bib-0496] Yelamanchili, S. V. , Lamberty, B. G. , Rennard, D. A. , Morsey, B. M. , Hochfelder, C. G. , Meays, B. M. , Levy, E. , & Fox, H. S. (2015). MiR‐21 in extracellular vesicles leads to neurotoxicity via TLR7 signaling in SIV neurological disease. Plos Pathogens, 11, e1005032. 26154133 10.1371/journal.ppat.1005032 PMC4496044

[jev212404-bib-0497] Yerneni, S. S. , Solomon, T. , Smith, J. , & Campbell, P. G. (2022). Radioiodination of extravesicular surface constituents to study the biocorona, cell trafficking and storage stability of extracellular vesicles. Biochimica et Biophysica Acta (BBA) ‐ General Subjects, 1866, 130069. 34906563 10.1016/j.bbagen.2021.130069

[jev212404-bib-0498] You, J. S. , Gelfanova, V. , Knierman, M. D. , Witzmann, F. A. , Wang, M. , & Hale, J. E. (2005). The impact of blood contamination on the proteome of cerebrospinal fluid. Proteomics, 5, 290–296. 15672452 10.1002/pmic.200400889

[jev212404-bib-0499] Young, G. , Hundt, N. , Cole, D. , Fineberg, A. , Andrecka, J. , Tyler, A. , Olerinyova, A. , Ansari, A. , Marklund, E. G. , Collier, M. P. , Chandler, S. A. , Tkachenko, O. , Allen, J. , Crispin, M. , Billington, N. , Takagi, Y. , Sellers, J. R. , Eichmann, C. , Selenko, P. , … Kukura, P. (2018). Quantitative mass imaging of single biological macromolecules. Science, 360, 423–427. 29700264 10.1126/science.aar5839 PMC6103225

[jev212404-bib-0500] Yuana, Y. , Koning, R. I. , Kuil, M. E. , Rensen, P. C. , Koster, A. J. , Bertina, R. M. , & Osanto, S. (2013). Cryo‐electron microscopy of extracellular vesicles in fresh plasma. Journal of Extracellular Vesicles, 2, 10.3402/jev.v2i0.21494 PMC3895263 24455109

[jev212404-bib-0501] Zavan, L. , Bitto, N. J. , Johnston, E. L. , Greening, D. W. , & Kaparakis‐Liaskos, M. (2019). *Helicobacter pylori* growth stage determines the size, protein composition, and preferential cargo packaging of outer membrane vesicles. Proteomics, 19, e1800209. 30488570 10.1002/pmic.201800209

[jev212404-bib-0502] Zavan, L. , Fang, H. , Johnston, E. L. , Whitchurch, C. , Greening, D. , Hill, A. F. , & KaparakisLiaskos, M. (2023). The mechanism of *Pseudomonas aeruginosa* outer membrane vesicle biogenesis determines their protein composition. Proteomics, e2200464. 36781972 10.1002/pmic.202200464

[jev212404-bib-0503] Zhang, H. , Freitas, D. , Kim, H. S. , Fabijanic, K. , Li, Z. , Chen, H. , Mark, M. T. , Molina, H. , Martin, A. B. , Bojmar, L. , Fang, J. , Rampersaud, S. , Hoshino, A. , Matei, I. , Kenific, C. M. , Nakajima, M. , Mutvei, A. P. , Sansone, P. , Buehring, W. , … Lyden, D. (2018). Identification of distinct nanoparticles and subsets of extracellular vesicles by asymmetric flow field‐flow fractionation. Nature Cell Biology, 20, 332–343. 29459780 10.1038/s41556-018-0040-4 PMC5931706

[jev212404-bib-0504] Zhang, H. , Lu, J. , Liu, J. , Zhang, G. , & Lu, A. (2020). Advances in the discovery of exosome inhibitors in cancer. Journal of Enzyme Inhibition and Medicinal Chemistry, 35, 1322–1330. 32543905 10.1080/14756366.2020.1754814 PMC7717571

[jev212404-bib-0505] Zhang, J. , Goodlett, D. R. , Peskind, E. R. , Quinn, J. F. , Zhou, Y. , Wang, Q. , Pan, C. , Yi, E. , Eng, J. , Aebersold, R. H. , & Montine, T. J. (2005). Quantitative proteomic analysis of age‐related changes in human cerebrospinal fluid. Neurobiology of Aging, 26, 207–227. 15582749 10.1016/j.neurobiolaging.2004.03.012

[jev212404-bib-0506] Zhang, K. , Yue, Y. , Wu, S. , Liu, W. , Shi, J. , & Zhang, Z. (2019). Rapid capture and nondestructive release of extracellular vesicles using aptamer‐based magnetic isolation. ACS Sensors, 4, 1245–1251. 30915846 10.1021/acssensors.9b00060

[jev212404-bib-0507] Zhang, Q. , Higginbotham, J. N. , Jeppesen, D. K. , Yang, Y. P. , Li, W. , McKinley, E. T. , Graves‐Deal, R. , Ping, J. , Britain, C. M. , Dorsett, K. A. , Hartman, C. L. , Ford, D. A. , Allen, R. M. , Vickers, K. C. , Liu, Q. , Franklin, J. L. , Bellis, S. L. , & Coffey, R. J. (2019). Transfer of functional cargo in exomeres. Cell Reports, 27, 940–954.e6. 30956133 10.1016/j.celrep.2019.01.009 PMC6559347

[jev212404-bib-0508] Zhang, Q. , Jeppesen, D. K. , Higginbotham, J. N. , Graves‐Deal, R. , Trinh, V. Q. , Ramirez, M. A. , Sohn, Y. , Neininger, A. C. , Taneja, N. , McKinley, E. T. , Niitsu, H. , Cao, Z. , Evans, R. , Glass, S. E. , Ray, K. C. , Fissell, W. H. , Hill, S. , Rose, K. L. , Huh, W. J. , … Coffey, R. J. (2021). Supermeres are functional extracellular nanoparticles replete with disease biomarkers and therapeutic targets. Nature Cell Biology, 23, 1240–1254. 34887515 10.1038/s41556-021-00805-8 PMC8656144

[jev212404-bib-0509] Zhang, S. , Wear, D. J. , & Lo, S. (2000). Mycoplasmal infections alter gene expression in cultured human prostatic and cervical epithelial cells. Fems Immunology and Medical Microbiology, 27, 43–50. 10617789 10.1111/j.1574-695X.2000.tb01410.x

[jev212404-bib-0510] Zhang, X. , Baht, G. S. , Huang, R. , Chen, Y. H. , Molitoris, K. H. , Miller, S. E. , & Kraus, V. B. (2022). Rejuvenation of neutrophils and their extracellular vesicles is associated with enhanced aged fracture healing. Aging Cell, 21, e13651. 35657721 10.1111/acel.13651 PMC9282841

[jev212404-bib-0511] Zhang, X. , Borg, E. G. F. , Liaci, A. M. , Vos, H. R. , & Stoorvogel, W. (2020). A novel three step protocol to isolate extracellular vesicles from plasma or cell culture medium with both high yield and purity. Journal of Extracellular Vesicles, 9, 1791450. 32944179 10.1080/20013078.2020.1791450 PMC7480457

[jev212404-bib-0512] Zhao, K. , Bleackley, M. , Chisanga, D. , Gangoda, L. , Fonseka, P. , Liem, M. , Kalra, H. , Al Saffar, H. , Keerthikumar, S. , Ang, C. S. , Adda, C. G. , Jiang, L. , Yap, K. , Poon, I. K. , Lock, P. , Bulone, V. , Anderson, M. , & Mathivanan, S. (2019). Extracellular vesicles secreted by *Saccharomyces cerevisiae* are involved in cell wall remodelling. Communications Biology, 2, 305. 31428693 10.1038/s42003-019-0538-8 PMC6688994

[jev212404-bib-0513] Zhu, S. , Ma, L. , Wang, S. , Chen, C. , Zhang, W. , Yang, L. , Hang, W. , Nolan, J. P. , Wu, L. , & Yan, X. (2014). Light‐scattering detection below the level of single fluorescent molecules for high‐resolution characterization of functional nanoparticles. ACS Nano, 8, 10998–11006. 25300001 10.1021/nn505162u PMC4212780

[jev212404-bib-0514] Zomer, A. , Maynard, C. , Verweij, F. J. , Kamermans, A. , Schäfer, R. , Beerling, E. , Schiffelers, R. M. , de Wit, E. , Berenguer, J. , Ellenbroek, S. I. J. , Wurdinger, T. , Pegtel, D. M. , & van Rheenen, J. (2015). In vivo imaging reveals extracellular vesicle‐mediated phenocopying of metastatic behavior. Cell, 161, 1046–1057. 26000481 10.1016/j.cell.2015.04.042 PMC4448148

[jev212404-bib-0515] Zomer, A. , Steenbeek, S. C. , Maynard, C. , & van Rheenen, J. (2016). Studying extracellular vesicle transfer by a Cre‐loxP method. Nature Protocols, 11, 87–101. 26658469 10.1038/nprot.2015.138

[jev212404-bib-0516] Zong, S. , Zong, J. , Chen, C. , Jiang, X. , Zhang, Y. , Wang, Z. , & Cui, Y. (2018). Single molecule localization imaging of exosomes using blinking silicon quantum dots. Nanotechnology, 29, 065705. 29265007 10.1088/1361-6528/aaa375

[jev212404-bib-0517] Zonneveld, M. I. , Brisson, A. R. , van Herwijnen, M. J. , Tan, S. , van de Lest, C. H. , Redegeld, F. A. , Garssen, J. , Wauben, M. H. , & Nolte‐’t Hoen, E. N. (2014). Recovery of extracellular vesicles from human breast milk is influenced by sample collection and vesicle isolation procedures. Journal of Extracellular Vesicles, 3, 10.3402/jev.v3.24215 PMC4139932 25206958

