# Correction to “Minimal information for studies of extracellular vesicles (MISEV2023): From basic to advanced approaches”

**DOI:** 10.1002/jev2.12451

**Published:** 2024-05-10

Welsh, J. A., Goberdhan, D. C. I., O'Driscoll, L., Buzas, E. I., Blenkiron, C., Bussolati, B., Cai, H., Di Vizio, D., Driedonks, T. A. P., Erdbrügger, U., Falcon‐Perez, J. M., Fu, Q.‐L., Hill, A. F., Lenassi, M., Lim, S. K., Mahoney, M. G., Mohanty, S., Möller, A., Nieuwland, R., … Witwer, K. W. (2024). Minimal information for studies of extracellular vesicles (MISEV2023): from basic to advanced approaches. *Journal of Extracellular Vesicles*, 13, e12404. https://doi.org/10.1002/jev2.12404

In the originally published article, Gisela D'Angelo was omitted from the MISEV Consortium. They have been added to the online version of the article. We apologize for this error.

